# Mapping inequalities in exclusive breastfeeding in low- and middle-income countries, 2000–2018

**DOI:** 10.1038/s41562-021-01108-6

**Published:** 2021-06-03

**Authors:** Natalia V. Bhattacharjee, Lauren E. Schaeffer, Simon I. Hay, Local Burden of Disease Exclusive Breastfeeding Collaborators, Dan Lu, Megan F. Schipp, Alice Lazzar-Atwood, Katie M. Donkers, Gdiom Gebreheat Abady, Foad Abd-Allah, Ahmed Abdelalim, Zeleke Hailemariam Abebo, Ayenew Negesse Abejie, Akine Eshete Abosetugn, Lucas Guimarães Abreu, Michael R. M. Abrigo, Eman Abu-Gharbieh, Abdelrahman I. Abushouk, Aishatu L. Adamu, Isaac Akinkunmi Adedeji, Adeyinka Emmanuel Adegbosin, Victor Adekanmbi, Olatunji O. Adetokunboh, Marcela Agudelo-Botero, Budi Aji, Oluwaseun Oladapo Akinyemi, Alehegn Aderaw Alamneh, Fahad Mashhour Alanezi, Turki M. Alanzi, James Albright, Jacqueline Elizabeth Alcalde-Rabanal, Biresaw Wassihun Alemu, Robert Kaba Alhassan, Beriwan Abdulqadir Ali, Saqib Ali, Cyrus Alinia, Vahid Alipour, Arianna Maever L. Amit, Dickson A. Amugsi, Etsay Woldu Anbesu, Robert Ancuceanu, Mina Anjomshoa, Fereshteh Ansari, Carl Abelardo T. Antonio, Davood Anvari, Jalal Arabloo, Amit Arora, Kurnia Dwi Artanti, Mulusew A. Asemahagn, Wondwossen Niguse Asmare, Maha Moh’d Wahbi Atout, Marcel Ausloos, Nefsu Awoke, Beatriz Paulina Ayala Quintanilla, Martin Amogre Ayanore, Yared Asmare Aynalem, Muluken Altaye Ayza, Zelalem Nigussie Azene, B. B. Darshan, Ashish D. Badiye, Atif Amin Baig, Shankar M. Bakkannavar, Maciej Banach, Palash Chandra Banik, Till Winfried Bärnighausen, Huda Basaleem, Mohsen Bayati, Bayisa Abdissa Baye, Neeraj Bedi, Sefealem Assefa Belay, Akshaya Srikanth Bhagavathula, Dinesh Bhandari, Nikha Bhardwaj, Pankaj Bhardwaj, Zulfiqar A. Bhutta, Ali Bijani, Tsegaye Adane Birhan, Binyam Minuye Birihane, Zebenay Workneh Bitew, Somayeh Bohlouli, Mahdi Bohluli, Hunduma Amensisa Bojia, Archith Boloor, Oliver J. Brady, Nicola Luigi Bragazzi, Andre R. Brunoni, Shyam S. Budhathoki, Sharath Burugina Nagaraja, Zahid A. Butt, Rosario Cárdenas, Joao Mauricio Castaldelli-Maia, Franz Castro, Achille Cernigliaro, Jaykaran Charan, Pranab Chatterjee, Souranshu Chatterjee, Vijay Kumar Chattu, Sarika Chaturvedi, Mohiuddin Ahsanul Kabir Chowdhury, Dinh-Toi Chu, Michael L. Collison, Aubrey J. Cook, Michael A. Cork, Rosa A. S. Couto, Baye Dagnew, Haijiang Dai, Lalit Dandona, Rakhi Dandona, Parnaz Daneshpajouhnejad, Aso Mohammad Darwesh, Amira Hamed Darwish, Ahmad Daryani, Jai K. Das, Rajat Das Gupta, Claudio Alberto Dávila-Cervantes, Adrian Charles Davis, Nicole Davis Weaver, Edgar Denova-Gutiérrez, Kebede Deribe, Assefa Desalew, Aniruddha Deshpande, Awrajaw Dessie, Keshab Deuba, Samath Dhamminda Dharmaratne, Meghnath Dhimal, Govinda Prasad Dhungana, Daniel Diaz, Alireza Didarloo, Isaac Oluwafemi Dipeolu, Linh Phuong Doan, Bereket Duko, Andre Rodrigues Duraes, Laura Dwyer-Lindgren, Lucas Earl, Maysaa El Sayed Zaki, Maha El Tantawi, Teshome Bekele Elema, Hala Rashad Elhabashy, Shaimaa I. El-Jaafary, Pawan Sirwan Faris, Andre Faro, Farshad Farzadfar, Valery L. Feigin, Berhanu Elfu Feleke, Tomas Y. Ferede, Florian Fischer, Nataliya A. Foigt, Morenike Oluwatoyin Folayan, Richard Charles Franklin, Mohamed M. Gad, Shilpa Gaidhane, William M. Gardner, Biniyam Sahiledengle Geberemariyam, Birhan Gebresillassie Gebregiorgis, Ketema Bizuwork Gebremedhin, Berhe Gebremichael, Fariborz Ghaffarpasand, Syed Amir Gilani, Themba G. Ginindza, Mustefa Glagn, Mahaveer Golechha, Kebebe Bekele Gonfa, Bárbara Niegia Garcia Goulart, Nachiket Gudi, Davide Guido, Rashid Abdi Guled, Yuming Guo, Samer Hamidi, Demelash Woldeyohannes Handiso, Ahmed I. Hasaballah, Amr Hassan, Khezar Hayat, Mohamed I. Hegazy, Behnam Heidari, Nathaniel J. Henry, Claudiu Herteliu, Hagos Degefa de Hidru, Hung Chak Ho, Chi Linh Hoang, Ramesh Holla, Julia Hon, Mostafa Hosseini, Mehdi Hosseinzadeh, Mowafa Househ, Mohamed Hsairi, Guoqing Hu, Tanvir M. Huda, Bing-Fang Hwang, Segun Emmanuel Ibitoye, Olayinka Stephen Ilesanmi, Irena M. Ilic, Milena D. Ilic, Leeberk Raja Inbaraj, Usman Iqbal, Seyed Sina Naghibi Irvani, M. Mofizul Islam, Chidozie C. D. Iwu, Chinwe Juliana Iwu, Animesh Jain, Manthan Dilipkumar Janodia, Tahereh Javaheri, Yetunde O. John-Akinola, Kimberly B. Johnson, Farahnaz Joukar, Jacek Jerzy Jozwiak, Ali Kabir, Leila R. Kalankesh, Rohollah Kalhor, Ashwin Kamath, Naser Kamyari, Other Tanuj Kanchan, Neeti Kapoor, Behzad Karami Matin, Salah Eddin Karimi, Habtamu Kebebe Kasaye, Getinet Kassahun, Nicholas J. Kassebaum, Gbenga A. Kayode, Ali Kazemi Karyani, Peter Njenga Keiyoro, Bayew Kelkay, Nauman Khalid, Md. Nuruzzaman Khan, Khaled Khatab, Amir M. Khater, Mona M. Khater, Mahalaqua Nazli Khatib, Yun Jin Kim, Ruth W. Kimokoti, Damaris K. Kinyoki, Adnan Kisa, Sezer Kisa, Soewarta Kosen, Kewal Krishan, Vaman Kulkarni, G. Anil Kumar, Manasi Kumar, Nithin Kumar, Pushpendra Kumar, Om P. Kurmi, Dian Kusuma, Carlo La Vecchia, Sheetal D. Lad, Faris Hasan Lami, Iván Landires, Van Charles Lansingh, Savita Lasrado, Paul H. Lee, Kate E. LeGrand, Ian D. Letourneau, Sonia Lewycka, Bingyu Li, Ming-Chieh Li, Shanshan Li, Xuefeng Liu, Rakesh Lodha, Jaifred Christian F. Lopez, Celia Louie, Daiane Borges Machado, Venkatesh Maled, Shokofeh Maleki, Deborah Carvalho Malta, Abdullah A. Mamun, Navid Manafi, Mohammad Ali Mansournia, Chabila Christopher Mapoma, Laurie B. Marczak, Francisco Rogerlândio Martins-Melo, Man Mohan Mehndiratta, Fabiola Mejia-Rodriguez, Tefera Chane Mekonnen, Walter Mendoza, Ritesh G. Menezes, Endalkachew Worku Mengesha, Abera M. Mersha, Ted R. Miller, G. K. Mini, Erkin M. Mirrakhimov, Sanjeev Misra, Masoud Moghadaszadeh, Dara K. Mohammad, Abdollah Mohammadian-Hafshejani, Jemal Abdu Mohammed, Shafiu Mohammed, Ali H. Mokdad, Pablo A. Montero-Zamora, Masoud Moradi, Rahmatollah Moradzadeh, Paula Moraga, Jonathan F. Mosser, Seyyed Meysam Mousavi, Amin Mousavi Khaneghah, Sandra B. Munro, Moses K. Muriithi, Ghulam Mustafa, Saravanan Muthupandian, Ahamarshan Jayaraman Nagarajan, Gurudatta Naik, Mukhammad David Naimzada, Vinay Nangia, Bruno Ramos Nascimento, Vinod C. Nayak, Rawlance Ndejjo, Duduzile Edith Ndwandwe, Ionut Negoi, Georges Nguefack-Tsague, Josephine W. Ngunjiri, Cuong Tat Nguyen, Diep Ngoc Nguyen, Huong Lan Thi Nguyen, Samuel Negash Nigussie, Tadesse T. N. Nigussie, Rajan Nikbakhsh, Chukwudi A. Nnaji, Virginia Nunez-Samudio, Bogdan Oancea, Onome Bright Oghenetega, Andrew T. Olagunju, Bolajoko Olubukunola Olusanya, Jacob Olusegun Olusanya, Muktar Omer Omer, Obinna E. Onwujekwe, Doris V. Ortega-Altamirano, Aaron E. Osgood-Zimmerman, Nikita Otstavnov, Stanislav S. Otstavnov, Mayowa O. Owolabi, P. A. Mahesh, Jagadish Rao Padubidri, Adrian Pana, Anamika Pandey, Seithikurippu R. Pandi-Perumal, Helena Ullyartha Pangaribuan, Shradha S. Parsekar, Deepak Kumar Pasupula, Urvish K. Patel, Ashish Pathak, Mona Pathak, Sanjay M. Pattanshetty, George C. Patton, Kebreab Paulos, Veincent Christian Filipino Pepito, Brandon V. Pickering, Marina Pinheiro, Ellen G. Piwoz, Khem Narayan Pokhrel, Hadi Pourjafar, Sergio I. Prada, Dimas Ria Angga Pribadi, Zahiruddin Quazi Syed, Mohammad Rabiee, Navid Rabiee, Fakher Rahim, Shadi Rahimzadeh, Azizur Rahman, Mohammad Hifz Ur Rahman, Amir Masoud Rahmani, Rajesh Kumar Rai, Chhabi Lal Ranabhat, Sowmya J. Rao, Prateek Rastogi, Priya Rathi, David Laith Rawaf, Salman Rawaf, Reza Rawassizadeh, Rahul Rawat, Ramu Rawat, Lemma Demissie Regassa, Maria Albertina Santiago Rego, Robert C. Reiner, Bhageerathy Reshmi, Aziz Rezapour, Ana Isabel Ribeiro, Jennifer Rickard, Leonardo Roever, Susan Fred Rumisha, Godfrey M. Rwegerera, Rajesh Sagar, S. Mohammad Sajadi, Marwa Rashad Salem, Abdallah M. Samy, Milena M. Santric-Milicevic, Sivan Yegnanarayana Iyer Saraswathy, Abdur Razzaque Sarker, Benn Sartorius, Brijesh Sathian, Deepak Saxena, Alyssa N. Sbarra, Debarka Sengupta, Subramanian Senthilkumaran, Feng Sha, Omid Shafaat, Amira A. Shaheen, Masood Ali Shaikh, Ali S. Shalash, Mohammed Shannawaz, Aziz Sheikh, B. Suresh Kumar Shetty, Ranjitha S. Shetty, Kenji Shibuya, Wondimeneh Shibabaw Shiferaw, Jae Il Shin, Diego Augusto Santos Silva, Narinder Pal Singh, Pushpendra Singh, Surya Singh, Yitagesu Sintayehu, Valentin Yurievich Skryabin, Anna Aleksandrovna Skryabina, Amin Soheili, Shahin Soltani, Muluken Bekele Sorrie, Emma Elizabeth Spurlock, Krista M. Steuben, Agus Sudaryanto, Mu’awiyyah Babale Sufiyan, Scott J. Swartz, Eyayou Girma Tadesse, Animut Tagele Tamiru, Leili Tapak, Md. Ismail Tareque, Ingan Ukur Tarigan, Getayeneh Antehunegn Tesema, Fisaha Haile Tesfay, Abinet Teshome, Zemenu Tadesse Tessema, Kavumpurathu Raman Thankappan, Rekha Thapar, Nihal Thomas, Roman Topor-Madry, Marcos Roberto Tovani-Palone, Eugenio Traini, Bach Xuan Tran, Phuong N. Truong, Berhan Tsegaye B. T. Tsegaye, Irfan Ullah, Chukwuma David Umeokonkwo, Bhaskaran Unnikrishnan, Era Upadhyay, Benjamin S. Chudi Uzochukwu, John David VanderHeide, Francesco S. Violante, Bay Vo, Yohannes Dibaba Wado, Yasir Waheed, Richard G. Wamai, Fang Wang, Yafeng Wang, Yuan-Pang Wang, Nuwan Darshana Wickramasinghe, Kirsten E. Wiens, Charles Shey Wiysonge, Lauren Woyczynski, Ai-Min Wu, Chenkai Wu, Tomohide Yamada, Sanni Yaya, Alex Yeshaneh, Yigizie Yeshaw, Yordanos Gizachew Yeshitila, Mekdes Tigistu Yilma, Paul Yip, Naohiro Yonemoto, Tewodros Yosef, Mustafa Z. Younis, Abdilahi Yousuf Yousuf, Chuanhua Yu, Yong Yu, Deniz Yuce, Shamsa Zafar, Syed Saoud Zaidi, Leila Zaki, Josefina Zakzuk, Maryam Zamanian, Heather J. Zar, Mikhail Sergeevich Zastrozhin, Anasthasia Zastrozhina, Desalege Amare Zelellw, Yunquan Zhang, Zhi-Jiang Zhang, Xiu-Ju George Zhao, Sanjay Zodpey, Yves Miel H. Zuniga, Simon I. Hay

**Affiliations:** 1 Institute for Health Metrics and Evaluation, University of Washington, Seattle, WA USA; 2 Medical Teams International, Seattle, WA USA; 3 Department of Pediatric Newborn Medicine, Brigham and Women’s Hospital, Boston, MA USA; 4 Department of Nursing, Adigrat University, Adigrat, Ethiopia; 5 Department of Neurology, Cairo University, Cairo, Egypt; 6 Department of Public Health, Arba Minch University, Arba Minch, Ethiopia; 7 Debre Markos University, Debre Markos, Ethiopia; 8 Department of Public Health, Debre Berhan University, Debre Brehan, Ethiopia; 9 Department of Pediatric Dentistry, Federal University of Minas Gerais, Belo Horizonte, Brazil; 10 Department of Research, Philippine Institute for Development Studies, Quezon City, Philippines; 11 Department of Clinical Sciences, University of Sharjah, Sharjah, United Arab Emirates; 12 Harvard Medical School, Harvard University, Boston, MA USA; 13 Department of Medicine, Ain Shams University, Cairo, Egypt; 14 Community Medicine Department, Bayero University Kano, Kano, Nigeria; 15 Infectious Diseases Epidemiology, London School of Hygiene & Tropical Medicine, London, UK; 16 Department of Sociology, Olabisi Onabanjo University, Ago-Iwoye, Nigeria; 17 School of Medicine, Griffith University, Gold coast, QLD Australia; 18 Population Health Sciences, King’s College London, London, England; 19 Centre of Excellence for Epidemiological Modelling and Analysis, Stellenbosch University, Stellenbosch, South Africa; 20 Department of Global Health, Stellenbosch University, Cape Town, South Africa; 21 Center for Policy, Population & Health Research, National Autonomous University of Mexico, Mexico City, Mexico; 22 Faculty of Medicine and Public Health, Jenderal Soedirman University, Purwokerto, Indonesia; 23 Department of Health Policy and Management, University of Ibadan, Ibadan, Nigeria; 24 Department of Health Policy and Management, University College Hospital, Ibadan, Ibadan, Nigeria; 25 Department of Human Nutrition and Food Sciences, Debre Markos University, Debre Markos, Ethiopia; 26 Imam Abdulrahman Bin Faisal University, Dammam, Saudi Arabia; 27 Health Information Management and Technology Department, Imam Abdulrahman Bin Faisal University, Dammam, Saudi Arabia; 28 Center for Health System Research, National Institute of Public Health, Cuernavaca, Mexico; 29 College of Medicine and Health Science, Arba Minch University, Arba Minch, Ethiopia; 30 Department of Midwifery, Arba Minch University, Injbara, Ethiopia; 31 Institute of Health Research, University of Health and Allied Sciences, Ho, Ghana; 32 Erbil Technical Health College, Erbil Polytechnic University, Erbil, Iraq; 33 School of Pharmacy, Tishk International University, Erbil, Iraq; 34 Department of Information Systems, College of Economics and Political Science, Sultan Qaboos University, Muscat, Oman; 35 Department of Health Care Management and Economics, Urmia University of Medical Science, Urmia, Iran; 36 Health Management and Economics Research Center, Iran University of Medical Sciences, Tehran, Iran; 37 Health Economics Department, Iran University of Medical Sciences, Tehran, Iran; 38 School of Medicine and Public Health, Ateneo De Manila University, Manila, Philippines; 39 College of Medicine, University of the Philippines Manila, Manila, Philippines; 40 Maternal and Child Wellbeing, African Population and Health Research Center, Nairobi, Kenya; 41 Department of Public Health, Samara University, Samara, Ethiopia; 42 Pharmacy Department, Carol Davila University of Medicine and Pharmacy, Bucharest, Romania; 43 Social Determinants of Health Research Center, Rafsanjan University of Medical Sciences, Rafsanjan, Iran; 44 Research Center for Evidence Based Medicine, Tabriz University of Medical Sciences, Tabriz, Iran; 45 Razi Vaccine and Serum Research Institute, Agricultural Research, Education, and Extension Organization (AREEO), Tehran, Iran; 46 Department of Health Policy and Administration, University of the Philippines Manila, Manila, Philippines; 47 Department of Applied Social Sciences, Hong Kong Polytechnic University, Hong Kong, China; 48 Department of Parasitology, Mazandaran University of Medical Sciences, Sari, Iran; 49 Department of Parasitology, Iranshahr University of Medical Sciences, Iranshahr, Iran; 50 School of Health Sciences, Western Sydney University, Campbelltown, NSW Australia; 51 Disciple of Child and Adolescent Health, University of Sydney, Westmead, NSW Australia; 52 Department of Epidemiology, Airlangga University, Surabaya, Indonesia; 53 School of Public Health, Bahir Dar University, Bahir Dar, Ethiopia; 54 Department of Nursing, Mizan-Tepi University, Mizan Teferi, Ethiopia; 55 Faculty of Nursing, Philadelphia University, Amman, Jordan; 56 School of Business, University of Leicester, Leicester, UK; 57 Department of Statistics and Econometrics, Bucharest University of Economic Studies, Bucharest, Romania; 58 Department of Nursing, Wolaita Sodo University, Wolaita Sodo, Ethiopia; 59 The Judith Lumley Centre, La Trobe University, Melbourne, VIC Australia; 60 Department of Health Policy Planning and Management, University of Health and Allied Sciences, Ho, Ghana; 61 Department of Nursing, Debre Berhan University, Debre Berhan, Ethiopia; 62 Department of Pharmacology and Toxicology, Mekelle University, Mekelle, Ethiopia; 63 Department of Reproductive Health, University of Gondar, Gondar, Ethiopia; 64 Kasturba Medical College, Mangalore, Manipal Academy of Higher Education, Manipal, India; 65 Department of Forensic Science, Government Institute of Forensic Science, Nagpur, India; 66 Unit of Biochemistry, Universiti Sultan Zainal Abidin (Sultan Zainal Abidin University), Kuala Terengganu, Malaysia; 67 Department of Forensic Medicine and Toxicology, Manipal Academy of Higher Education, Manipal, India; 68 Department of Hypertension, Medical University of Lodz, Lodz, Poland; 69 Polish Mothers’ Memorial Hospital Research Institute, Lodz, Poland; 70 Department of Non-communicable Diseases, Bangladesh University of Health Sciences, Dhaka, Bangladesh; 71 Heidelberg Institute of Global Health (HIGH), Heidelberg University, Heidelberg, Germany; 72 T.H. Chan School of Public Health, Harvard University, Boston, MA USA; 73 School of Public Health and Community Medicine, Aden College, Aden, Yemen; 74 Health Human Resources Research Center, Shiraz University of Medical Sciences, Shiraz, Iran; 75 Department of Public Health, Ambo University, Ambo, Ethiopia; 76 Department of Community Medicine, Gandhi Medical College Bhopal, Bhopal, India; 77 Jazan University, Jazan, Saudi Arabia; 78 Department of Biomedical Science, Bahir Dar University, Bahir Dar, Ethiopia; 79 Department of Social and Clinical Pharmacy, Charles University, Hradec Kralova, Czech Republic; 80 Institute of Public Health, United Arab Emirates University, Al Ain, United Arab Emirates; 81 School of Public Health, University of Adelaide, Adelaide, SA Australia; 82 Public Health Research Laboratory, Tribhuvan University, Kathmandu, Nepal; 83 Department of Anatomy, Government Medical College Pali, Pali, India; 84 Department of Community Medicine and Family Medicine, All India Institute of Medical Sciences, Jodhpur, India; 85 School of Public Health, All India Institute of Medical Sciences, Jodhpur, India; 86 Centre for Global Child Health, University of Toronto, Toronto, ON Canada; 87 Centre of Excellence in Women & Child Health, Aga Khan University, Karachi, Pakistan; 88 Social Determinants of Health Research Center, Babol University of Medical Sciences, Babol, Iran; 89 Department of Environmental and Occupational Health and Safety, University of Gondar, Gondar, Ethiopia; 90 Ethiopian Public Health Institute, Addis Ababa, Ethiopia; 91 Department of Nursing, Debre Tabor University, Debretabor, Ethiopia; 92 Nutrition Department, St. Paul’s Hospital Millennium Medical College, Addis Ababa, Ethiopia; 93 St. Paul’s Hospital Millennium Medical College, Addis Ababa, Ethiopia; 94 Department of Veterinary Medicine, Islamic Azad University, Kermanshah, Iran; 95 Department of Computer Science and Information Technology, Institute for Advanced Studies in Basic Sciences, Zanjan, Iran; 96 Department of Research and Innovation, Petanux Research GmBH, Bonn, Germany; 97 School of Pharmacy, Haramaya University, Harar, Ethiopia; 98 Department of Internal Medicine, Manipal Academy of Higher Education, Mangalore, India; 99 Department of Infectious Disease Epidemiology, London School of Hygiene & Tropical Medicine, London, UK; 100 University of Genoa, Genoa, Italy; 101 Department of Internal Medicine, University of São Paulo, São Paulo, Brazil; 102 Department of Psychiatry, University of São Paulo, São Paulo, Brazil; 103 Research Division, Golden Community, Kathmandu, Nepal; 104 Department of Community Medicine, Employee State Insurance Post Graduate Institute of Medical Sciences and Research, Bangalore, India; 105 School of Public Health and Health Systems, University of Waterloo, Waterloo, ON Canada; 106 Al Shifa School of Public Health, Al Shifa Trust Eye Hospital, Rawalpindi, Pakistan; 107 Department of Health Care, Metropolitan Autonomous University, Mexico City, Mexico; 108 Department of Psychiatry, University of São Paulo, Sao Paulo, Brazil; 109 Gorgas Memorial Institute for Health Studies, Panama City, Panama; 110 Regional Epidemiological Observatory Department, Sicilian Regional Health Authority, Palermo, Italy; 111 Department of Pharmacology, All India Institute of Medical Sciences, Jodhpur, India; 112 Department of International Health, Johns Hopkins Bloomberg School of Public Health, Baltimore, MD USA; 113 Department of Microbiology & Infection Control, Medanta Medicity, Gurugram, India; 114 Department of Medicine, University of Toronto, Toronto, ON Canada; 115 Global Institute of Public Health (GIPH), Thiruvananthapuram, India; 116 Research Department, Dr. D. Y. Patil University, Pune, India; 117 Maternal and Child Health Division, International Centre for Diarrhoeal Disease Research, Bangladesh, Dhaka, Bangladesh; 118 Department of Epidemiology and Biostatistics, University of South Carolina, Columbia, SC USA; 119 Center for Biomedicine and Community Health, VNU International School, Hanoi, Vietnam; 120 Department of Chemical Sciences, University of Porto, Porto, Portugal; 121 Department of Human Physiology, University of Gondar, Gondar, Ethiopia; 122 Department of Cardiology, Central South University, Changsha, China; 123 Department of Mathematics and Statistics, York University, Toronto, ON Canada; 124 Public Health Foundation of India, Gurugram, India; 125 Indian Council of Medical Research, New Delhi, India; 126 Department of Health Metrics Sciences, School of Medicine, University of Washington, Seattle, WA USA; 127 Department of Pathology, Johns Hopkins University School of Medicine, Baltimore, MD USA; 128 Department of Pathology, Isfahan University of Medical Sciences, Isfahan, Iran; 129 Department of Information Technology, University of Human Development, Sulaymaniyah, Iraq; 130 Department of Pediatrics, Tanta University, Tanta, Egypt; 131 Toxoplasmosis Research Center, Mazandaran University of Medical Sciences, Sari, Iran; 132 Division of Women and Child Health, Aga Khan University, Karachi, Pakistan; 133 James P Grant School of Public Health, BRAC University, Dhaka, Bangladesh; 134 Department of Population and Development, Latin American Faculty of Social Sciences Mexico, Mexico City, Mexico; 135 Department of Surgery and Cancer, Imperial College London, London, UK; 136 Ear Institute, University College London, London, UK; 137 Center for Nutrition and Health Research, National Institute of Public Health, Cuernavaca, Mexico; 138 Wellcome Trust Brighton and Sussex Centre for Global Health Research, Brighton and Sussex Medical School, Brighton, UK; 139 School of Public Health, Addis Ababa University, Addis Ababa, Ethiopia; 140 School of Nursing and Midwifery, Haramaya University, Harar, Ethiopia; 141 Department of Epidemiology, Emory University, Atlanta, GA USA; 142 National Centre for AIDS and STD Control, Save the Children, Kathmandu, Nepal; 143 Department of Global Public Health, Karolinska Institute, Stockholm, Sweden; 144 Department of Community Medicine, University of Peradeniya, Peradeniya, Sri Lanka; 145 Health Research Section, Nepal Health Research Council, Kathmandu, Nepal; 146 Department of Microbiology, Far Western University, Mahendranagar, Nepal; 147 Center of Complexity Sciences, National Autonomous University of Mexico, Mexico City, Mexico; 148 Faculty of Veterinary Medicine and Zootechnics, Autonomous University of Sinaloa, Culiacán Rosales, Mexico; 149 Department of Public Health, Urmia University of Medical Science, Urmia, Iran; 150 Department of Health Promotion and Education, University of Ibadan, Ibadan, Nigeria; 151 Institute for Global Health Innovations, Duy Tan University, Da Nang, Vietnam; 152 School of Public Health, Hawassa University, Hawassa, Ethiopia; 153 School of Public Health, Curtin University, Perth, WA Australia; 154 School of Medicine, Federal University of Bahia, Salvador, Brazil; 155 Department of Internal Medicine, Escola Bahiana de Medicina e Saúde Pública (Bahiana School of Medicine and Public Health), Salvador, Brazil; 156 Clinical Pathology Department, Mansoura Faculty of Medicine, Mansoura, Egypt; 157 Pediatric Dentistry and Dental Public Health Department, Alexandria University, Alexandria, Egypt; 158 Department of Food Science and Nutrition, Arsi University, Asella, Ethiopia; 159 Center for Food Science and Nutrition, Addis Ababa University, Addis Ababa, Ethiopia; 160 Neurophysiology Department, Cairo University, Cairo, Egypt; 161 Department of Biology and Biotechnology “Lazzaro Spallanzani”, University of Pavia, Pavia, Italy; 162 Department of Biology, Cihan University-Erbil, Erbil, Iraq; 163 Department of Psychology, Federal University of Sergipe, São Cristóvão, Brazil; 164 Non-communicable Diseases Research Center, Tehran University of Medical Sciences, Tehran, Iran; 165 National Institute for Stroke and Applied Neurosciences, Auckland University of Technology, Auckland, New Zealand; 166 Research Center of Neurology, Moscow, Russia; 167 Department of Epidemiology and Biostatistics, Bahir Dar University, Bahir Dar, Ethiopia; 168 School of Nursing, Hawassa University, Hawassa, Ethiopia; 169 Institute of Gerontological Health Services and Nursing Research, Ravensburg-Weingarten University of Applied Sciences, Weingarten, Germany; 170 Institute of Gerontology, National Academy of Medical Sciences of Ukraine, Kyiv, Ukraine; 171 Department of Child Dental Health, Obafemi Awolowo University, Ile-Ife, Nigeria; 172 School of Public Health, Medical, and Veterinary Sciences, James Cook University, Douglas, QLD Australia; 173 Department of Cardiovascular Medicine, Cleveland Clinic, Cleveland, OH USA; 174 Gillings School of Global Public Health, University of North Carolina Chapel Hill, Chapel Hill, NC USA; 175 Department of Medicine, Datta Meghe Institute of Medical Science, Wardha, India; 176 Department of Public Health, Madda Walabu University, Bale Robe, Ethiopia; 177 Department of Nursing and Midwifery, Addis Ababa University, Addis Ababa, Ethiopia; 178 School of Public Health, Haramaya University, Harar, Ethiopia; 179 Department of Neurosurgery, Shiraz University of Medical Sciences, Shiraz, Iran; 180 Faculty of Allied Health Sciences, The University of Lahore, Lahore, Pakistan; 181 Afro-Asian Institute, Lahore, Pakistan; 182 Discipline of Public Health Medicine, University of KwaZulu-Natal, Durban, South Africa; 183 Department of Public Health, Arba Minch University, Arba Minch, Ethiopia; 184 Health Systems and Policy Research, Indian Institute of Public Health Gandhinagar, Gandhinagar, India; 185 Department of Surgery, Madda Walabu University, Bale Robe, Ethiopia; 186 Postgraduate Program in Epidemiology, Federal University of Rio Grande do Sul, Porto Alegre, Brazil; 187 Department of Health Policy, Manipal Academy of Higher Education, Manipal, India; 188 UO Neurologia, Salute Pubblica e Disabilità, Fondazione IRCCS Istituto Neurologico Carlo Besta (Neurology, Public Health and Disability Unit, Carlo Besta Neurological Institute), Milan, Italy; 189 College of Medicine and Health Science, Jigjiga University, Jijiga, Ethiopia; 190 Department of Epidemiology and Preventive Medicine, Monash University, Melbourne, VIC Australia; 191 Department of Epidemiology, Binzhou Medical University, Yantai City, China; 192 School of Health and Environmental Studies, Hamdan Bin Mohammed Smart University, Dubai, United Arab Emirates; 193 Department of Public Health, Wachemo University, Hossana, Ethiopia; 194 Department of Zoology and Entomology, Al Azhar University, Cairo, Egypt; 195 Institute of Pharmaceutical Sciences, University of Veterinary and Animal Sciences, Lahore, Pakistan; 196 Department of Pharmacy Administration and Clinical Pharmacy, Xian Jiaotong University, Xian, China; 197 Endocrinology and Metabolism Research Center, Tehran University of Medical Sciences, Tehran, Iran; 198 Nuffield Department of Clinical Medicine, University of Oxford, Oxford, UK; 199 School of Business, London South Bank University, London, UK; 200 Department of Public Health, Adigrat University, Adigrat, Ethiopia; 201 Department of Urban Planning and Design, University of Hong Kong, Hong Kong, China; 202 Center of Excellence in Behavioral Medicine, Nguyen Tat Thanh University, Ho Chi Minh City, Vietnam; 203 Department of Epidemiology and Biostatistics, Tehran University of Medical Sciences, Tehran, Iran; 204 Pediatric Chronic Kidney Disease Research Center, Tehran University of Medical Sciences, Tehran, Iran; 205 College of Science and Engineering, Hamad Bin Khalifa University, Doha, Qatar; 206 Faculty of Medicine of Tunis, University Tunis El Manar, Tunis, Tunisia; 207 Department of Epidemiology and Health Statistics, Central South University, Changsha, China; 208 School of Public Health, University of Sydney, Sydney, NSW Australia; 209 Department of Occupational Safety and Health, China Medical University, Taichung, Taiwan; 210 Department of Community Medicine, University of Ibadan, Ibadan, Nigeria; 211 Department of Community Medicine, University College Hospital, Ibadan, Ibadan, Nigeria; 212 Faculty of Medicine, University of Belgrade, Belgrade, Serbia; 213 Department of Epidemiology, University of Kragujevac, Kragujevac, Serbia; 214 Division of Community Health and Family Medicine, Bangalore Baptist Hospital, Bangalore, India; 215 College of Public Health, Taipei Medical University, Taipei, Taiwan; 216 Research Institute for Endocrine Sciences, Shahid Beheshti University of Medical Sciences, Tehran, Iran; 217 School of Psychology and Public Health, La Trobe University, Melbourne, VIC Australia; 218 School of Health Systems and Public Health, University of Pretoria, Pretoria, South Africa; 219 South African Medical Research Council, Cape Town, South Africa; 220 Department of Community Medicine, Manipal Academy of Higher Education, Mangalore, India; 221 Manipal College of Pharmaceutical Sciences, Manipal Academy of Higher Education, Manipal, India; 222 Health Informatic Lab, Boston University, Boston, MA USA; 223 Gastrointestinal and Liver Diseases Research Center, Guilan University of Medical Sciences, Rasht, Iran; 224 Caspian Digestive Disease Research Center, Guilan University of Medical Sciences, Rasht, Iran; 225 Department of Family Medicine and Public Health, University of Opole, Opole, Poland; 226 Minimally Invasive Surgery Research Center, Iran University of Medical Sciences, Tehran, Iran; 227 School of Management and Medical Informatics, Tabriz University of Medical Sciences, Tabriz, Iran; 228 Institute for Prevention of Non-communicable Diseases, Qazvin University of Medical Sciences, Qazvin, Iran; 229 Health Services Management Department, Qazvin University of Medical Sciences, Qazvin, Iran; 230 Manipal Academy of Higher Education, Manipal, India; 231 Department of Biostatistics, Hamadan University of Medical Sciences, Hamadan, Iran; 232 Department of Forensic Medicine and Toxicology, All India Institute of Medical Sciences, Jodhpur, India; 233 Research Center for Environmental Determinants of Health, Kermanshah University of Medical Sciences, Kermanshah, Iran; 234 Social Determinants of Health Research Center, Tabriz University of Medical Sciences, Tabriz, Iran; 235 School of Nursing and Midwifery, Wollega University, Nekemte, Ethiopia; 236 School of Midwifery, Hawassa University, Hawassa, Ethiopia; 237 Department of Anesthesiology & Pain Medicine, University of Washington, Seattle, WA USA; 238 International Research Center of Excellence, Institute of Human Virology Nigeria, Abuja, Nigeria; 239 Julius Centre for Health Sciences and Primary Care, Utrecht University, Utrecht, Netherlands; 240 Open, Distance and eLearning Campus, University of Nairobi, Nairobi, Kenya; 241 Department of Midwifery, University of Gondar, Gondar, Ethiopia; 242 School of Food and Agricultural Sciences, University of Management and Technology, Lahore, Pakistan; 243 Department of Population Science, Jatiya Kabi Kazi Nazrul Islam University, Mymensingh, Bangladesh; 244 Faculty of Health and Wellbeing, Sheffield Hallam University, Sheffield, UK; 245 College of Arts and Sciences, Ohio University, Zanesville, OH USA; 246 National Hepatology and Tropical Medicine Research Institute, Cairo University, Cairo, Egypt; 247 Department of Medical Parasitology, Cairo University, Cairo, Egypt; 248 Global Evidence Synthesis Initiative, Datta Meghe Institute of Medical Sciences, Wardha, India; 249 School of Traditional Chinese Medicine, Xiamen University Malaysia, Sepang, Malaysia; 250 Department of Nutrition, Simmons University, Boston, MA USA; 251 School of Health Sciences, Kristiania University College, Oslo, Norway; 252 Global Community Health and Behavioral Sciences, Tulane University, New Orleans, LA USA; 253 Department of Nursing and Health Promotion, Oslo Metropolitan University, Oslo, Norway; 254 Independent Consultant, Jakarta, Indonesia; 255 Department of Anthropology, Panjab University, Chandigarh, India; 256 Department of Psychiatry, University of Nairobi, Nairobi, Kenya; 257 Division of Psychology and Language Sciences, University College London, London, UK; 258 International Institute for Population Sciences, Mumbai, India; 259 Faculty of Health and Life Sciences, Coventry University, Coventry, UK; 260 Department of Medicine, McMaster University, Hamilton, ON Canada; 261 Imperial College Business School, Imperial College London, London, UK; 262 Faculty of Public Health, University of Indonesia, Depok, Indonesia; 263 Department of Clinical Sciences and Community Health, University of Milan, Milan, Italy; 264 Department of Pediatrics, Post Graduate Institute of Medical Education and Research, Chandigarh, India; 265 Department of Community and Family Medicine, University of Baghdad, Baghdad, Iraq; 266 Unit of Genetics and Public Health, Institute of Medical Sciences, Las Tablas, Panama; 267 Ministry of Health, Herrera, Panama; 268 HelpMeSee, New York, NY USA; 269 Mexican Institute of Ophthalmology, Queretaro, Mexico; 270 Department of Otorhinolaryngology, Father Muller Medical College, Mangalore, India; 271 School of Nursing, Hong Kong Polytechnic University, Hong Kong, China; 272 Centre for Tropical Medicine and Global Health, University of Oxford, Oxford, UK; 273 Oxford University Clinical Research Unit, Wellcome Trust Asia Programme, Hanoi, Vietnam; 274 Department of Sociology, Shenzhen University, Shenzhen, China; 275 Department of Public Health, China Medical University, Taichung, Taiwan; 276 School of Public Health and Preventive Medicine, Monash University, Melbourne, VIC Australia; 277 Department of Systems, Populations, and Leadership, University of Michigan, Ann Arbor, MI USA; 278 Department of Paediatrics, All India Institute of Medical Sciences, New Delhi, India; 279 Department of Nutrition, University of the Philippines Manila, Manila, Philippines; 280 Alliance for Improving Health Outcomes, Inc., Quezon City, Philippines; 281 Center for Integration of Data and Health Knowledge, Oswald Cruz Foundation (FIOCRUZ), Salvador, Brazil; 282 Centre for Global Mental Health (CGMH), London School of Hygiene & Tropical Medicine, London, England; 283 Department of Forensic Medicine, Shri Dharmasthala Manjunatheshwara University, Dharwad, India; 284 Department of Forensic Medicine, Rajiv Gandhi University of Health Sciences, Bangalore, India; 285 Clinical Research Development Center, Kermanshah University of Medical Sciences, Kermanshah, Iran; 286 Department of Maternal and Child Nursing and Public Health, Federal University of Minas Gerais, Belo Horizonte, Brazil; 287 Institute for Social Science Research, The University of Queensland, Indooroopilly, QLD Australia; 288 School of Medicine, Iran University of Medical Sciences, Tehran, Iran; 289 School of Medicine, University of Manitoba, Winnipeg, MB Canada; 290 Department of Population Studies, University of Zambia, Lusaka, Zambia; 291 Campus Caucaia, Federal Institute of Education, Science and Technology of Ceará, Caucaia, Brazil; 292 Neurology Department, Janakpuri Super Specialty Hospital Society, New Delhi, India; 293 Department of Neurology, Govind Ballabh Institute of Medical Education and Research, New Delhi, India; 294 Research in Nutrition and Health, National Institute of Public Health, Cuernavaca, Mexico; 295 Department of Public Health, Wollo University, Dessie, Ethiopia; 296 Peru Country Office, United Nations Population Fund (UNFPA), Lima, Peru; 297 Forensic Medicine Division, Imam Abdulrahman Bin Faisal University, Dammam, Saudi Arabia; 298 Department of Reproductive Health and Population Studies, Bahir Dar University, Bahir Dar, Ethiopia; 299 Department of Nursing, Arba Minch University, Arba Minch, Ethiopia; 300 Pacific Institute for Research & Evaluation, Calverton, MD USA; 301 Global Institute of Public Health, Ananthapuri Hospitals and Research Institute, Trivandrum, India; 302 Women’s Social and Health Studies Foundation, Trivandrum, India; 303 Internal Medicine Programme, Kyrgyz State Medical Academy, Bishkek, Kyrgyzstan; 304 Department of Atherosclerosis and Coronary Heart Disease, National Center of Cardiology and Internal Disease, Bishkek, Kyrgyzstan; 305 Department of Surgical Oncology, All India Institute of Medical Sciences, Jodhpur, India; 306 Biotechnology Research Center, Tabriz University of Medical Sciences, Tabriz, Iran; 307 Molecular Medicine Research Center, Tabriz University of Medical Sciences, Tabriz, Iran; 308 Department of Forestry, Salahaddin University-Erbil, Erbil, Iraq; 309 Department of Medicine-Huddinge, Karolinska Institute, Stockholm, Sweden; 310 Department of Epidemiology and Biostatistics, Shahrekord University of Medical Sciences, Shahrekord, Iran; 311 Department of Public Health, Samara University, Semera, Ethiopia; 312 Health Systems and Policy Research Unit, Ahmadu Bello University, Zaria, Nigeria; 313 Department of Public Health Sciences, University of Miami, Miami, FL USA; 314 Center for Health Systems Research, National Institute of Public Health, Cuernavaca, Mexico; 315 Department of Epidemiology, Arak University of Medical Sciences, Arak, Iran; 316 Computer, Electrical, and Mathematical Sciences and Engineering Division, King Abdullah University of Science and Technology, Thuwal, Saudi Arabia; 317 Management and Leadership in Medical Education Research Center, Kerman University of Medical Sciences, Kerman, Iran; 318 Department of Food Science, University of Campinas (Unicamp), Campinas, Brazil; 319 School of Economics, University of Nairobi, Nairobi, Kenya; 320 Department of Pediatric Medicine, The Children’s Hospital & The Institute of Child Health, Multan, Pakistan; 321 Department of Pediatrics & Pediatric Pulmonology, Institute of Mother & Child Care, Multan, Pakistan; 322 Department of Microbiology and Immunology, Mekelle University, Mekelle, Ethiopia; 323 Research and Analytics Department, Initiative for Financing Health and Human Development, Chennai, India; 324 Department of Research and Analytics, Bioinsilico Technologies, Chennai, India; 325 Comprehensive Cancer Center, University of Alabama at Birmingham, Birmingham, AL USA; 326 Laboratory of Public Health Indicators Analysis and Health Digitalization, Moscow Institute of Physics and Technology, Dolgoprudny, Russia; 327 Experimental Surgery and Oncology Laboratory, Kursk State Medical University, Kursk, Russia; 328 Suraj Eye Institute, Nagpur, India; 329 Department of Clinical Medicine, Federal University of Minas Gerais, Belo Horizonte, Brazil; 330 Clinical Hospital, Federal University of Minas Gerais, Belo Horizonte, Brazil; 331 Disease Control and Environmental Health, Makerere University, Kampala, Uganda; 332 Cochrane South Africa, South African Medical Research Council, Cape Town, South Africa; 333 Department of General Surgery, Carol Davila University of Medicine and Pharmacy, Bucharest, Romania; 334 Department of General Surgery, Emergency Hospital of Bucharest, Bucharest, Romania; 335 Department of Public Health, University of Yaoundé I, Yaoundé, Cameroon; 336 Department of Biological Sciences, University of Embu, Embu, Kenya; 337 Institute for Global Health Innovations, Duy Tan University, Hanoi, Vietnam; 338 Faculty of Pharmacy, Duy Tan University, Da Nang, Vietnam; 339 Department of Public Health, Mizan-Tepi University, Mizan Teferi, Ethiopia; 340 Obesity Research Center, Shahid Beheshti University of Medical Sciences, Tehran, Iran; 341 School of Public Health and Family Medicine, University of Cape Town, Cape Town, South Africa; 342 Unit of Microbiology and Public Health, Institute of Medical Sciences, Las Tablas, Panama; 343 Department of Public Health, Ministry of Health, Herrera, Panama; 344 Administrative and Economic Sciences Department, University of Bucharest, Bucharest, Romania; 345 Department of Obstetrics and Gynecology, University of Ibadan, Ibadan, Nigeria; 346 Department of Psychiatry and Behavioural Neurosciences, McMaster University, Hamilton, ON Canada; 347 Department of Psychiatry, University of Lagos, Lagos, Nigeria; 348 Centre for Healthy Start Initiative, Lagos, Nigeria; 349 Department of Public Health, Jigjiga University, Jijiga, Ethiopia; 350 Department of Pharmacology and Therapeutics, University of Nigeria Nsukka, Enugu, Nigeria; 351 Health Systems Research Center, National Institute of Public Health, Cuernavaca, Mexico; 352 Department of Project Management, National Research University Higher School of Economics, Moscow, Russia; 353 Department of Medicine, University of Ibadan, Ibadan, Nigeria; 354 Department of Medicine, University College Hospital, Ibadan, Ibadan, Nigeria; 355 Department of Respiratory Medicine, Jagadguru Sri Shivarathreeswara Academy of Health Education and Research, Mysore, India; 356 Department of Health Metrics, Center for Health Outcomes & Evaluation, Bucharest, Romania; 357 Department of Research, Public Health Foundation of India, Gurugram, India; 358 Corporate, Somnogen Canada Inc, Toronto, ON Canada; 359 National Institute of Health Research and Development, Ministry of Health, Jakarta, Indonesia; 360 Public Health Evidence South Asia, Manipal Academy of Higher Education, Manipal, India; 361 Division of General Internal Medicine, University of Pittsburgh Medical Center, Pittsburgh, PA USA; 362 Department of Neurology and Public Health, Icahn School of Medicine at Mount Sinai, New York, NY USA; 363 Department of Pediatrics, RD Gardi Medical College, Ujjain, India; 364 Global Public Health-Health Systems and Policy (HSP): Medicines Focusing Antibiotics, Karolinska Institute, Stockholm, Sweden; 365 Research & Development Department, Kalinga Institute of Medical Sciences, Bhubaneswar, India; 366 Department of Pediatrics, University of Melbourne, Melbourne, VIC Australia; 367 Population Health Theme, Murdoch Childrens Research Institute, Melbourne, VIC Australia; 368 Department of Midwifery, Wolaita Sodo University, Wolaita Sodo, Ethiopia; 369 Center for Research and Innovation, Ateneo De Manila University, Pasig City, Philippines; 370 Department of Chemistry, University of Porto, Porto, Portugal; 371 Global Development Program, Bill & Melinda Gates Foundation, Seattle, WA USA; 372 HIV and Mental Health Department, Integrated Development Foundation Nepal, Kathmandu, Nepal; 373 Department of Nutrition and Food Sciences, Maragheh University of Medical Sciences, Maragheh, Iran; 374 Dietary Supplements and Probiotic Research Center, Alborz University of Medical Sciences, Karaj, Iran; 375 Centro de Investigaciones Clinicas, Fundación Valle del Lili, (Clinical Research Center, Valle del Lili Foundation), Cali, Colombia; 376 Centro PROESA, Universidad ICESI, (PROESA, ICESI University), Cali, Colombia; 377 Health Sciences Department, Muhammadiyah University of Surakarta, Sukoharjo, Indonesia; 378 Department of Community Medicine, Datta Meghe Institute of Medical Sciences, Wardha, India; 379 Biomedical Engineering Department, Amirkabir University of Technology, Tehran, Iran; 380 Department of Chemistry, Sharif University of Technology, Tehran, Iran; 381 Thalassemia and Hemoglobinopathy Research Center, Ahvaz Jundishapur University of Medical Sciences, Ahvaz, Iran; 382 Metabolomics and Genomics Research Center, Tehran University of Medical Sciences, Tehran, Iran; 383 Department of Natural Science, Middlesex University, London, UK; 384 Data Mining Research Unit (DaMRA), Charles Sturt University, Wagga Wagga, NSW Australia; 385 Department of Community Medicine, Maharishi Markandeshwar Medical College & Hospital, Solan, India; 386 Future Technology Research Center, National Yunlin University of Science and Technology, Yunlin, Taiwan; 387 Institute of Research and Development, Duy Tan University, Da Nang, Vietnam; 388 Society for Health and Demographic Surveillance, Suri, India; 389 Department of Economics, University of Göttingen, Göttingen, Germany; 390 Research Department, Policy Research Institute, Kathmandu, Nepal; 391 Health and Public Policy Department, Global Center for Research and Development, Kathmandu, Nepal; 392 Department of Oral Pathology, Srinivas Institute of Dental Sciences, Mangalore, India; 393 Department of Forensic Medicine and Toxicology, Manipal Academy of Higher Education, Mangalore, India; 394 WHO Collaborating Centre for Public Health Education and Training, Imperial College London, London, UK; 395 University College London Hospitals, London, UK; 396 Department of Primary Care and Public Health, Imperial College London, London, UK; 397 Academic Public Health England, Public Health England, London, UK; 398 Department of Computer Science, Boston University, Boston, MA USA; 399 Maternal, Newborn, and Child Health Program, Bill & Melinda Gates Foundation, Seattle, WA USA; 400 Department of Mathematical Demography & Statistics, International Institute for Population Sciences, Mumbai, India; 401 Department of Pediatrics, Federal University of Minas Gerais, Belo Horizonte, Brazil; 402 Department of Health Information Management, Manipal Academy of Higher Education, Manipal, India; 403 Manipal Academy of Higher Education, Manipal, India; 404 Epidemiology Research Unit Institute of Public Health (EPIUnit-ISPUP), University of Porto, Porto, Portugal; 405 Department of Surgery, University of Minnesota, Minneapolis, MN USA; 406 Department of Surgery, University Teaching Hospital of Kigali, Kigali, Rwanda; 407 Department of Clinical Research, Federal University of Uberlândia, Uberlândia, Brazil; 408 Malaria Atlas Project, University of Oxford, Oxford, UK; 409 Department of Health Statistics, National Institute for Medical Research, Dar es Salaam, Tanzania; 410 Department of Internal Medicine, University of Botswana, Gaborone, Botswana; 411 Department of Psychiatry, All India Institute of Medical Sciences, New Delhi, India; 412 Department of Phytochemistry, Soran University, Soran, Iraq; 413 Department of Nutrition, Cihan University-Erbil, Erbil, Iraq; 414 Public Health and Community Medicine Department, Cairo University, Giza, Egypt; 415 Department of Entomology, Ain Shams University, Cairo, Egypt; 416 School of Public Health and Health Management, University of Belgrade, Belgrade, Serbia; 417 Department of Community Medicine, PSG Institute of Medical Sciences and Research, Coimbatore, India; 418 PSG-FAIMER South Asia Regional Institute, Coimbatore, India; 419 Health Economics Department, Bangladesh Institute of Development Studies (BIDS), Dhaka, Bangladesh; 420 Centre for Tropical Medicine and Global Health, University of Oxford, Oxford, UK; 421 Nuffield Department of Medicine, University of Oxford, Oxford, UK; 422 Department of Geriatrics and Long Term Care, Hamad Medical Corporation, Doha, Qatar; 423 Faculty of Health & Social Sciences, Bournemouth University, Bournemouth, UK; 424 Department of Epidemiology, Indian Institute of Public Health, Gandhinagar, India; 425 Department of Computational Biology, Indraprastha Institute of Information Technology, Delhi, India; 426 Emergency Department, Manian Medical Centre, Erode, India; 427 Center for Biomedical Information Technology, Shenzhen Institutes of Advanced Technology, Shenzhen, China; 428 Department of Radiology and Radiological Science, Johns Hopkins University, Baltimore, MD USA; 429 Department of Radiology and Interventional Neuroradiology, Isfahan University of Medical Sciences, Isfahan, Iran; 430 Public Health Division, An-Najah National University, Nablus, Palestine; 431 Independent consultant, Karachi, Pakistan; 432 Neurology Department, Ain Shams University, Cairo, Egypt; 433 Department of Community Medicine, BLDE University, Vijayapur, India; 434 Centre for Medical Informatics, University of Edinburgh, Edinburgh, UK; 435 Division of General Internal Medicine, Harvard University, Boston, MA USA; 436 Department of Community Medicine, Manipal Academy of Higher Education, Manipal, India; 437 Institute for Population Health, King’s College London, London, UK; 438 College of Medicine, Yonsei University, Seoul, South Korea; 439 Department of Physical Education, Federal University of Santa Catarina, Florianópolis, Brazil; 440 Faculty of Medicine and Health Sciences, Shree Guru Gobind Singh Tricentenary University, Gurugram, India; 441 Department of Humanities and Social Sciences, Indian Institute of Technology, Roorkee, Roorkee, India; 442 Division of Environmental Monitoring & Exposure Assessment (Water & Soil), National Institute for Research in Environmental Health, Bhopal, India; 443 Department of Midwifery, Haramaya University, Harar, Ethiopia; 444 Department No.16, Moscow Research and Practical Centre on Addictions, Moscow, Russia; 445 Therapeutic Department, Balashiha Central Hospital, Balashikha, Russia; 446 Nursing Care Research Center, Semnan University of Medical Sciences, Semnan, Iran; 447 Department of Nursing, Muhammadiyah University of Surakarta, Surakarta, Indonesia; 448 Department of Community Medicine, Ahmadu Bello University, Zaria, Nigeria; 449 School of Medicine, University of California San Francisco, San Francisco, CA USA; 450 Joint Medical Program, University of California Berkeley, Berkeley, CA USA; 451 Department of Biomedical Sciences, Arba Minch University, Arba Minch, Ethiopia; 452 Non-communicable Diseases Research Center, Hamadan University of Medical Sciences, Hamadan, Iran; 453 Department of Population Science and Human Resource Development, University of Rajshahi, Rajshahi, Bangladesh; 454 Research and Development Center for Humanities and Health Management, National Institute of Health Research & Development, Jakarta, Indonesia; 455 Department of Epidemiology and Biostatistsics, University of Gondar, Gondar, Ethiopia; 456 School of Public Health, Mekelle University, Mekelle, Ethiopia; 457 Southgate Institute for Health and Society, Flinders University, Adelaide, SA Australia; 458 Department of Epidemiology and Biostatistics, University of Gondar, Gondar, Ethiopia; 459 Department of Public Health and Community Medicine, Central University of Kerala, Kasaragod, India; 460 Department of Endocrinology, Diabetes and Metabolism, Christian Medical College and Hospital (CMC), Vellore, India; 461 Institute of Public Health, Jagiellonian University Medical College, Kraków, Poland; 462 Agency for Health Technology Assessment and Tariff System, Warsaw, Poland; 463 Department of Pathology and Legal Medicine, University of São Paulo, Ribeirão Preto, Brazil; 464 Modestum LTD, London, UK; 465 Institute for Risk Assessment Sciences (IRAS), Utrecht University, Utrecht, Netherlands; 466 Department of Health Economics, Hanoi Medical University, Hanoi, Vietnam; 467 Faculty of Geo-Information Science and Earth Observation, University of Twente, Enschede, Netherlands; 468 Department of Allied Health Sciences, Iqra National University, Peshawar, Pakistan; 469 Department of Community Medicine, Alex Ekwueme Federal University Teaching Hospital Abakaliki, Abakaliki, Nigeria; 470 Kasturba Medical College, Manipal Academy of Higher Education, Mangalore, India; 471 Amity Institute of Biotechnology, Amity University Rajasthan, Jaipur, India; 472 Department of Community Medicine, University of Nigeria Nsukka, Enugu, Nigeria; 473 Insights Program, Bill & Melinda Gates Foundation, Seattle, WA USA; 474 Department of Medical and Surgical Sciences, University of Bologna, Bologna, Italy; 475 Occupational Health Unit, Sant’Orsola Malpighi Hospital, Bologna, Italy; 476 Faculty of Information Technology, Ho Chi Minh City University of Technology (HUTECH), Ho Chi Minh City, Vietnam; 477 Population Dynamics and Sexual and Reproductive Health, African Population and Health Research Center, Nairobi, Kenya; 478 Foundation University Medical College, Foundation University Islamabad, Islamabad, Pakistan; 479 Cultures, Societies and Global Studies, & Integrated Initiative for Global Health, Northeastern University, Boston, MA USA; 480 School of Public Health, University of Nairobi, Nairobi, Kenya; 481 School of Health Sciences, Wuhan University, Wuhan, China; 482 Department of Epidemiology and Biostatistics, Wuhan University, Wuhan, China; 483 Department of Community Medicine, Rajarata University of Sri Lanka, Anuradhapura, Sri Lanka; 484 Department of Epidemiology, Johns Hopkins University, Baltimore, MD USA; 485 Department of Orthopaedics, Wenzhou Medical University, Wenzhou, China; 486 Global Health Research Center, Duke Kunshan University, Kunshan, China; 487 Duke Global Health Institute, Duke University, Durham, NC USA; 488 Department of Diabetes and Metabolic Diseases, University of Tokyo, Tokyo, Japan; 489 School of International Development and Global Studies, University of Ottawa, Ottawa, ON Canada; 490 The George Institute for Global Health, University of Oxford, Oxford, UK; 491 Department of Midwifery, Wolkite University, Wolkite, Ethiopia; 492 Department of Public Health, Wollega University, Nekemte, Ethiopia; 493 Centre for Suicide Research and Prevention, University of Hong Kong, Hong Kong, China; 494 Department of Social Work and Social Administration, University of Hong Kong, Hong Kong, China; 495 Department of Neuropsychopharmacology, National Center of Neurology and Psychiatry, Kodaira, Japan; 496 Department of Public Health, Juntendo University, Tokyo, Japan; 497 Department of Health Policy and Management, Jackson State University, Jackson, MS USA; 498 School of Medicine, Tsinghua University, Beijing, China; 499 School of Public Health and Management, Hubei University of Medicine, Shiyan, China; 500 Cancer Institute, Hacettepe University, Ankara, Turkey; 501 Department of Obstetrics and Gynaecology, Fazaia Medical College, Islamabad, Pakistan; 502 Department of Obstetrics and Gynaecology, Air University, Islamabad, Pakistan; 503 Department of Pharmaceutics, Dow University of Health Sciences, Karachi, Pakistan; 504 Department of Parasitology and Entomology, Tarbiat Modares University, Tehran, Iran; 505 Institute for Immunological Research, University of Cartagena, Cartagena, Colombia; 506 Department of Paediatrics & Child Health, University of Cape Town, Cape Town, South Africa; 507 Unit on Child & Adolescent Health, Medical Research Council South Africa, Cape Town, South Africa; 508 Laboratory of Genetics and Genomics, Moscow Research and Practical Centre on Addictions, Moscow, Russia; 509 Addictology Department, Russian Medical Academy of Continuous Professional Education, Moscow, Russia; 510 Pediatrics Department, Russian Medical Academy of Continuous Professional Education, Moscow, Russia; 511 Department of Pediatrics and Child Health Nursing, Bahir Dar University, Bahir Dar, Ethiopia; 512 School of Public Health, Wuhan University of Science and Technology, Wuhan, China; 513 Hubei Province Key Laboratory of Occupational Hazard Identification and Control, Wuhan University of Science and Technology, Wuhan, China; 514 School of Medicine, Wuhan University, Wuhan, China; 515 School of Biology and Pharmaceutical Engineering, Wuhan Polytechnic University, Wuhan, China; 516 Indian Institute of Public Health, Public Health Foundation of India, Gurugram, India; 517 Health Technology Assessment Unit, Department of Health Philippines, Manila, Philippines; 518 #MentalHealthPH, Inc., Quezon City, Philippines

**Keywords:** Nutrition disorders, Risk factors, Developing world

## Abstract

Exclusive breastfeeding (EBF)—giving infants only breast-milk for the first 6 months of life—is a component of optimal breastfeeding practices effective in preventing child morbidity and mortality. EBF practices are known to vary by population and comparable subnational estimates of prevalence and progress across low- and middle-income countries (LMICs) are required for planning policy and interventions. Here we present a geospatial analysis of EBF prevalence estimates from 2000 to 2018 across 94 LMICs mapped to policy-relevant administrative units (for example, districts), quantify subnational inequalities and their changes over time, and estimate probabilities of meeting the World Health Organization’s Global Nutrition Target (WHO GNT) of ≥70% EBF prevalence by 2030. While six LMICs are projected to meet the WHO GNT of ≥70% EBF prevalence at a national scale, only three are predicted to meet the target in all their district-level units by 2030.

## Abstract (web summary)

Bhattacharjee and Schaeffer et al. map exclusive breastfeeding (EBF) in 94 low- and middle-income countries (LMICs), finding increased EBF practice and reduced subnational variation across the majority of LMICs from 2000 to 2018. However, only six LMICs will meet WHO’s target of ≥70% EBF by 2030 nationally, and only three will achieve this in all districts.

## Main

Exclusive breastfeeding (EBF)—giving infants only breast-milk (and medications, vitamins or oral rehydration solution (ORS) as needed) for the first 6 months of life—is effective in preventing deaths from diarrhoea, pneumonia and other leading causes of child mortality^1–4^. Breast-milk has been characterized as a ‘personalized medicine’ for infants^2^ due to its nutritional properties, natural growth stimulators and tailored immune-protective properties, which collectively contribute to infant growth, development and survival^5–8^. Furthermore, evidence suggests long-term health benefits of breastfeeding, including reduced risks of cardiovascular diseases and increased benefits to human capital in adulthood^2,9,10^. The introduction of supplementary food and water during the first 6 months of life, particularly in settings lacking reliable access to clean water, can expose infants to infections from a range of pathogens^1,3^. Along with the initiation of breastfeeding within the first hour after birth and continued breastfeeding to 2 years, the World Health Organization (WHO) considers EBF to be an optimal breastfeeding practice^11^ and included it as a proven protective intervention in the Global Action Plan for Pneumonia and Diarrhoea (GAPPD)^1^. Despite the benefits, the proportion of exclusively breastfed children remains low in many low- and middle-income countries (LMICs), where most child deaths attributed to suboptimal breastfeeding occur^12^. Accelerated uptake in EBF is required to successfully achieve the World Health Organization’s Global Nutrition Target (WHO GNT) of at least 50% EBF prevalence by 2025^11^ and the recently updated WHO GNT of at least 70% EBF prevalence by 2030^13^.

This study is a part of a body of work mapping high-spatial-resolution estimates to track progress toward the WHO GNTs^14–17^. Building on our previous geospatial analysis of EBF prevalence in sub-Saharan Africa^14^, we synthesized data from 349 geo-referenced household surveys from years 1998 to 2018 representing 302,435 infants under 6 months to produce annual 2000–2018 subnational estimates for the proportion and absolute number of exclusively breastfed infants for 94 LMICs. We used 14 geographically distinct modelling regions which were determined on the basis of epidemiological homogeneity and geographical contiguity by the Global Burden of Disease (GBD) study^18^ (Supplementary Table 4 and Supplementary Fig. 7). We first mapped estimates on a 5 × 5-km grid to align with the resolution of many of the covariates used in this study and then aggregated to more policy-relevant second- and first-administrative-level units for each country in our analysis. Here we provide mapped annual estimates of EBF prevalence and trends at policy-relevant administrative and national levels from 2000 to 2018, as well as the estimated number of infants not receiving EBF. On the basis of trends in the most recent years, we project these estimates to the years 2025 and 2030, and determine the probability of meeting the WHO GNTs of ≥50% and ≥70% EBF prevalence in the respective target years. Furthermore, we examine relative and absolute subnational inequalities of EBF prevalence within LMICs and compare areas with low EBF prevalence to areas with high disease burden and low coverage of mitigating interventions. The full array of our model outputs—at various spatial levels and aggregations—is available through an online visualization tool (https://vizhub.healthdata.org/lbd/ebf), with additional results in the Supplementary Information.

## Results

### Regional, national and subnational trends in EBF prevalence

**Fig. 1:**
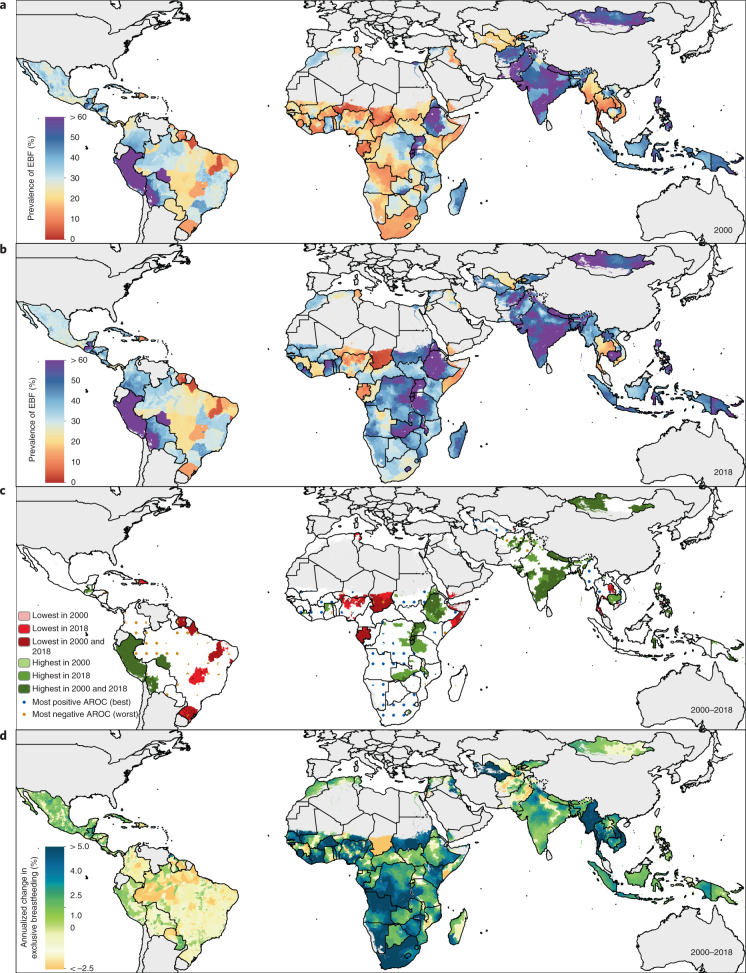
EBF prevalence and progress (2000–2018) among infants under 6 months across LMICs. **a**,**b**, Prevalence of EBF practices at the district level in 2000 (**a**) and 2018 (**b**). **c**, Overlapping population-weighted highest and lowest deciles of prevalence and weighted AROC between 2000 and 2018, at the district level. **d**, Weighted annualized percentage change in EBF prevalence between 2000 and 2018. Maps reflect administrative boundaries, land cover, lakes and population; grey-coloured grid cells had fewer than ten people per 1 × 1-km grid cell and were classified as ‘barren or sparsely vegetated’ or were not included in this analysis^50–55^.

EBF prevalence varied widely between and within LMICs from 2000 to 2018 (Fig. 1a,b). General increases in mean EBF prevalence occurred across LMICs over the study period, from 28.6% (95% uncertainty interval: 22.9–35.4%) in 2000 to 38.7% (28.3–49.9%) in 2018. Regionally, most LMICs in Andean South America, South Asia and East Asia had relatively high EBF levels throughout the study period; for example, Peru (63.6% (60.9–66.4%) in 2000; 69.2% (57.6–79.1%) in 2018), Nepal (64.2% (49.1–76.9%) in 2000; 64.5% (53.6–74.3%) in 2018) and Mongolia (51.9% (49.3–54.4%) in 2000; 55.1% (52.1–58.1%) in 2018) all maintained high national EBF prevalence. Several countries in other regions maintained low EBF prevalence throughout the study, including the Dominican Republic (13.2% (9.7–17.8%) in 2000; 8.2% (4.7–14.3%) in 2018), Suriname (6.4% (4.3–9.2%); 5.7% (3.2–9.6%)), Tunisia (10.9% (6.7–17.0%); 12.2% (7.7–18.0%)), Yemen (11.7% (4.7–22.3%); 12.5% (7.3–20.2%)) and Thailand (7.5% (4.9–11.1%); 13.9% (9.8–19.0%)). National 2018 EBF levels varied broadly between countries in the regions of Central America and the Caribbean (8.2% (4.7–14.3%) in the Dominican Republic; 50.7% (40.3–61.7%) in Guatemala), Tropical South America (5.7% (3.2–9.6%) in Suriname; 32.4% (29.1–35.8%) in Paraguay), Central Asia (18.7% (13.9–24.7%) in Uzbekistan; 51.8% (44.9–58.8%) in Afghanistan), Southeast Asia (13.9% (9.8–19.0%) in Thailand; 62.0% (50.4–72.9%) in Cambodia), North Africa (12.2% (7.7–18.0%) in Tunisia; 51.3% (44.7–57.6%) in Sudan) and throughout sub-Saharan Africa. Overall, in 2018, national EBF prevalence varied by as much as 39.2 times across all LMICs, ranging from 2.2% (1.1–4.0%) in Chad (Western sub-Saharan Africa) to 87.7% (76.9–94.2%) in Rwanda (Eastern sub-Saharan Africa).

Select LMICs made notable progress in the study period. In 2000, 57 LMICs had <30% estimated mean EBF prevalence in at least half of their first-administrative-level units (henceforth ‘provinces’); by 2018, eight of these countries had increased mean EBF prevalence to come close to the original WHO GNT of 50% EBF prevalence, with at least 45% EBF in most provinces: Cambodia (88.2%; 30 of 34 provinces), Democratic Republic of the Congo (DRC; 69.2%; 18 of 26), Guinea-Bissau (77.8%; 7 of 9), Lesotho (100.0%; 10 of 10), Liberia (80.0%; 12 of 15), Sudan (88.9%; 16 of 18) and Turkmenistan (66.7%; 4 of 6). For example, Kâmpóng Chhnang province in central Cambodia (19.5% (14.0–26.3%) in 2000; 63.4% (47.5–77.8%) in 2018) and West Kurdufan state in southern Sudan (13.4% (10.4–17.2%) in 2000; 51.9% (40.6–63.0%) in 2018) both experienced large gains. Overall, 34 LMICs had at least one province that made similar gains from <30% to ≥45% EBF prevalence (45.1%; 296 of 656 provinces across these 34 LMICs).

To compare trends and prevalence levels, we overlaid the highest and lowest population-weighted deciles of EBF at the second-administrative level (henceforth ‘district’) to the highest and lowest deciles of annualized rates of change (AROC) (Fig. 1c). Along with having some of the lowest levels of EBF practice in 2000 and 2018, Chad, Suriname, Somalia and Brazil also had among the highest rates of annualized decline in EBF during the study period. Districts in Niger, Nigeria, Gabon, Yemen, Tunisia, the Dominican Republic, southern Thailand and central Philippines also had among the lowest EBF prevalence levels in both 2000 and 2018; even despite some of the highest rates of EBF increase in southern Vietnam and northeastern Thailand, EBF remained among the lowest levels in these districts in both years (Fig. 1c). Districts throughout much of Peru, southeastern Bolivia, eastern Brazil, Ethiopia, Uganda, Rwanda, Burundi, India, Nepal, Mongolia and the Philippines had among the highest prevalence levels in both years; as did select districts in Guatemala, Zambia, Malawi, Eritrea, Afghanistan, Pakistan and Indonesia. Districts scattered throughout Guatemala, the DRC, northern Liberia, northern Ghana, Eritrea, western Tanzania, Zambia, Malawi, Lesotho, Bangladesh and Cambodia had among the highest levels for the year 2018, as did select districts in western Honduras, eastern and western Sudan and northern Laos. Districts with some of the highest rates of annualized increase in EBF were located in southern Sierra Leone, central Côte d’Ivoire, southern Burkina Faso, central Niger, central Nigeria, Sudan, eastern Ethiopia, DRC, Angola, Namibia, South Africa, northern Mozambique, central Kenya, Turkmenistan, western Kyrgyzstan, Myanmar, northern Thailand, southern Laos and southern Vietnam. In contrast, the highest rates of annualized decline in EBF were seen in eastern Honduras, Colombia, Brazil, eastern Bolivia, eastern Zambia, eastern Ghana, eastern Niger, central Nigeria, central Mozambique, central Madagascar, central Afghanistan and Pakistan. The Philippines and Brazil both had among the best-performing and worst-performing districts for both years in regard to prevalence and Niger, Nigeria and Mozambique had districts among the highest and lowest rates of annualized change.

By mapping AROC from 2000 to 2018, we show where and to what degree EBF practices have increased or decreased on average over the study period (Fig. 1d). Most district-level units across LMICs experienced increases in estimated mean prevalence of EBF over the study period (62.6%; 15,379 of 24,556 districts), while over a third experienced decreases 37.2% (9,137 of 24,556 districts). Overall, 28 LMICs experienced annualized increases in mean EBF prevalence in all districts; 25 LMICs had >2.5% annualized increase in all districts, including Bangladesh, Cambodia, Botswana, Liberia and Lesotho (Supplementary Table 8a). Sudan, Zimbabwe, South Africa, Kenya, Myanmar and Turkmenistan were among 14 LMICs that experienced among the highest annualized EBF increases (>5% AROC) in all of their districts’ mean estimates (Supplementary Table 8a). In 13 LMICs, most districts had decreasing annualized trends in EBF practice (<0% AROC); Chad was the only LMIC that experienced EBF declines in all of its districts (Supplementary Table 8b). A large proportion (69.1%; 65 of 94) of LMICs had both annualized increases and decreases in EBF across their districts; 7 (7.4%) LMICs had districts that had experienced both extremes of the mapped annualized increases (>5%) and decreases (≤−2.5%): Nigeria, Somalia, Mozambique, Niger, Thailand, the Philippines and India (Supplementary Table 8c).

### Comparison of units with low EBF and other health conditions

To identify some of the highest-need provinces across LMICs, we compared the lowest decile of EBF prevalence in this study to the highest decile levels of our previously published geospatial estimates of stunting^16^, childhood diarrhoea^19^ and under-5-yr mortality^20^ and the lowest decile of coverage of ORS^21^ and access to piped water^22^ (Supplementary Information section 5.5 and Supplementary Figs. 19–23). Several provinces in Chad had among the lowest levels of EBF, as well as some of the highest levels of under-5-yr mortality, stunting and diarrhoea and some of the lowest coverage levels of ORS and access to piped water. Also among the lowest levels of EBF, select provinces in Nigeria had among the lowest ORS coverage and both Niger and Nigeria had provinces with low EBF prevalence and some of the highest child stunting and mortality rates. Yemen had provinces with codistribution of low EBF prevalence and high levels of child diarrhoea and stunting. Somalia had several provinces with low EBF and high under-5-yr mortality rates, while Gabon had one province with among the highest childhood diarrhoea rates and lowest EBF prevalence. One province in Comoros and several provinces in Thailand had among the lowest levels of EBF as well as access to piped water (Supplementary Table 12).

### Geographic inequalities in EBF prevalence

We calculated Gini coefficients as a measure of geographic inequality at the country level^23^. Our results suggest that geographic inequality in EBF prevalence decreased in most of the countries from 2000 to 2018 (77 of 94) on the basis of Gini coefficients; while there were 11 countries in 2000 whose Gini coefficient was >0.25, only Nigeria and the Philippines had coefficients above 0.25 in 2018.

**Fig. 2:**
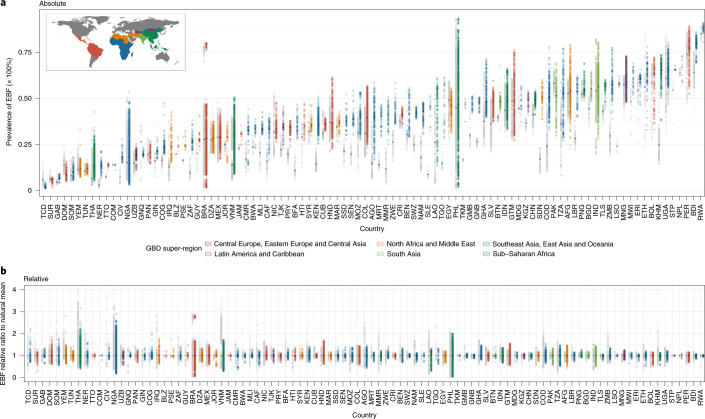
Geographic inequalities in EBF prevalence across 94 countries for 2000 and 2018. **a**, Absolute inequalities: range of EBF estimates in district-level units within 94 LMICs. **b**, Relative inequalities: range of ratios of EBF estimates in district-level units relative to country means. Each dot represents a district-level unit. The lower bound of each bar represents the district-level unit with the lowest EBF in each country. The upper end of each bar represents the district-level unit with the highest EBF in each country. Thus, each bar represents the extent of geographic inequality in EBF estimated for each country. Bars indicating the range in 2018 are coloured according to their GBD super-region. Grey bars indicate the range in EBF in 2000. The black diamond in each bar represents the median EBF estimated across district-level units in each country and year. A coloured bar that is shorter than its grey counterpart indicates that geographic inequality has narrowed. Countries are labeled by their ISO 3 codes (full country names are listed in Supplementary Table 4).

We quantified absolute geographic inequalities in EBF prevalence by calculating the absolute differences between district-level units with the lowest and highest prevalence in each country (method details in Supplementary Information section 4.4.3). Between 2000 and 2018, absolute geographic inequalities had increased in over a third (38.3%; 36 of 94) of LMICs, at least doubling in eight countries, including Afghanistan, Jamaica, Jordan, Nepal, Niger, Republic of the Congo, Sierra Leone and Turkmenistan (Fig. 2). Of the 92.6% (88 of 94) of LMICs which had increased in EBF national prevalence, almost half (42.1% (37 of 88)) had also increased in absolute inequalities—including in Afghanistan and Republic of the Congo—indicating areas left behind in overall national progress. While 39.3% (37 of 94) of LMICs had increased absolute inequalities between districts, 12.6% (12 of 94) of LMICs decreased their absolute inequalities; absolute inequalities in the other 45 LMICs in the analysis remained relatively the same. Several countries had reduced absolute inequalities by at least one-third while also increasing their EBF prevalence, including Burundi, Cuba, Eritrea, Gabon, Guinea, Malawi, Mali, Rwanda, Trinidad and Tobago and Uganda. Absolute inequalities in EBF were at least halved in eight LMICs: Burundi, Chad, Cuba, Eritrea, Gambia, Guinea, Mali and Rwanda. Along with substantial reductions in absolute inequalities, Gambia also substantially increased its national EBF prevalence, while Guinea, Mali and Rwanda experienced marginal increases in national prevalence; Chad, however, had decreased EBF prevalence across all its district-level units. In 2018, absolute differences in EBF between the highest- and lowest-prevalent districts within countries ranged from 1.1 to 45.3 times; São Tomé and Príncipe had the least variation, ranging from 66.0% (29.8–90.9%) in Me-Zochi (São Tomé) to 67.8% (31.3–93.0%) in Pague (Príncipe), while the Philippines ranged from 1.5% (0.9–2.3%) in San Jose (Antique) to 92.8% (88.6–95.9%) in Bagamanoc (Catanduanes). Most LMICs (60.0% (57 of 94)) had twofold or more difference in EBF between districts in 2000; 36.8% (35 of 94) had this difference in 2018. A threefold or greater difference between units was experienced in 34 (35.8%) and 15 (15.8%) LMICs in 2000 and 2018, respectively. A sixfold or greater difference was experienced by 14 (14.7%) LMICs in 2000 and 4 (4.2%) LMICs in 2018—Brazil, Nigeria, the Philippines and Thailand.

**Fig. 3:**
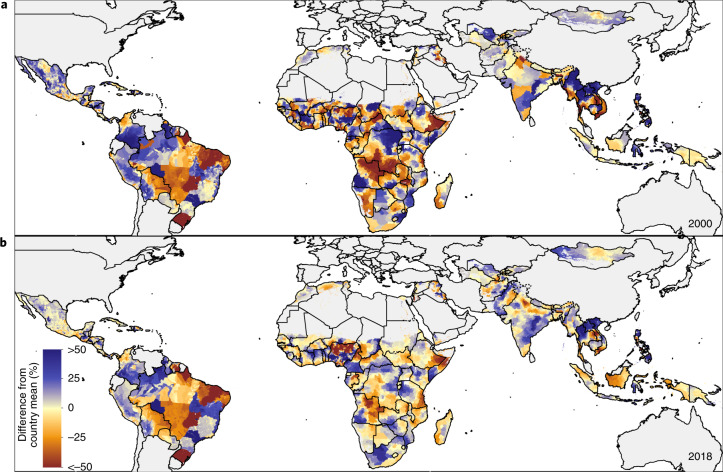
Relative geographic inequalities within countries in EBF prevalence in 2000 and 2018: comparing district-level units to the country-level means. **a**,**b**, Relative deviation of EBF prevalence in district-level units from the country-level EBF mean in 2000 (**a**) and 2018 (**b**). Blue indicates a positive deviation from the EBF country-level mean, indicating a higher EBF prevalence level. Red indicates a negative deviation from the EBF country-level mean, indicating a lower EBF prevalence level. Maps reflect administrative boundaries, land cover, lakes and population; grey-coloured grid cells had fewer than ten people per 1 × 1-km grid cell and were classified as ‘barren or sparsely vegetated’ or were not included in this analysis^50–55^.

We quantified relative inequalities by calculating the relative differences between each district-level unit and its country’s average for 2000 and 2018 (Supplementary Information section 4.4.3). Overall, within-country relative inequalities in EBF coverage declined; 48 LMICs in 2000 and 25 LMICs in 2018 had district-level units that deviated by >50% from the country mean (Fig. 2). Throughout the study period, Belize, Egypt, Eritrea and Papua New Guinea demonstrated low within-country relative differences in EBF, whereas Myanmar, Cambodia, Laos, Ghana and Peru had reduced relative geographic inequalities over time (Fig. 3). As an example, northern districts of Myanmar positively deviated and southwestern districts negatively deviated by ≥30% from the national mean in 2000 but these within-country relative differences decreased to <10% from the national mean in either direction by 2018. Within-country relative inequalities remained high, however, in Comoros, Brazil, the Philippines and Guyana in both 2000 and 2018. In 2018, the largest relative inequalities were in Nigeria, Brazil, Thailand and the Philippines. In particular, Nigeria’s most negatively deviating district-level units were concentrated in the north and southeast, while central districts loomed largely above the mean in 2018 (20.1% (18.8–21.4%) national mean; 3.4% (1.7–5.9%) in Baure (Osun); 53.7% (41.3–62.7%) in Ife Central (Osun)). Additionally, in Brazil, deviating patterns were scattered, with districts throughout much of the Amazon Basin in the west positively deviating from the national mean (for example, 80.1% (80.8–83.7) in Machadinho municipality (Rondônia) in 2018) and many districts in the Brazilian Highlands negatively deviating from the mean (for example, 10.7% (9.9–11.7%) in Abadia de Goiás municipality (Goiás); national mean 27.1% (25.6–28.6%)).

### Absolute number of children not exclusively breastfed

**Fig. 4:**
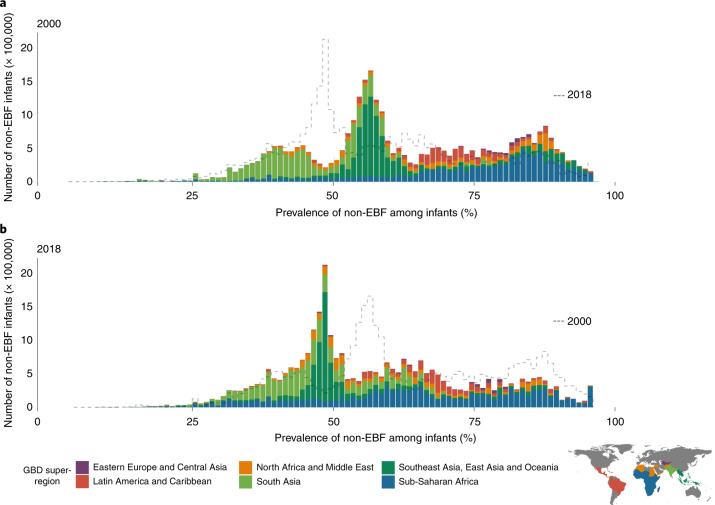
Number of infants under 6 months who are not being exclusively breastfed, distributed across non-EBF prevalence in 2000 and 2018, across 94 countries. **a**, Non-EBF infants under 6 months in 2000. **b**, Non-EBF infants under 6 months in 2018. The dotted line in the 2000 plot is the shape of the distribution in 2018 and the dotted line in the 2018 plot represents the distribution in 2000. Bar heights represent the total number of infants under 6 months who were not exclusively breastfed by district-level units, with corresponding non-EBF prevalence. Bins are a width of one non-EBF infant per 100 infants. The colour of each bar represents the global region as defined by the subset legend map. As such, the sum of heights of all bars represents the total number of non-EBF infants across the 94 countries.

In 2000, of the ~56,039,700 (51,145,700–60,940,400) infants under 6 months in the populations across the 94 countries in our analysis (according to the 2019 GBD Study^24^), an estimated 33,489,000 (31,867,900–35,031,200) infants were not exclusively breastfed. In 2018, among a population of ~57,787,200 (51,016,200–64,661,000) infants under 6 months in 94 LMICs^24^, an estimated 31,878,600 (28,721,500–34,999,000) children were not exclusively breastfed, representing a 4.8% (0.1–9.9%) decrease since 2000. A comparison of shifts in prevalence and numbers of non-EBF children over the past two decades suggests that, despite some of the largest increases in EBF prevalence in Asia and Oceania, the bulk of the total number of infants not benefiting from EBF still comes from these regions (Fig. 4).

**Fig. 5:**
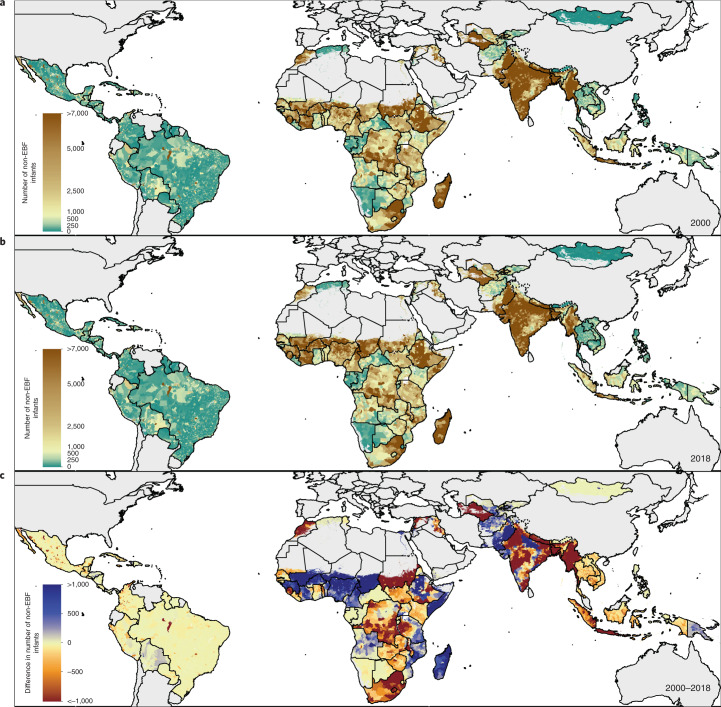
Number of infants under 6 months who are not being exclusively breastfed at the district level, 2000 and 2018. **a**,**b**, Number of infants under 6 months who are not being exclusively breastfed, aggregated to district-level units in 2000 (**a**) and 2018 (**b**). **c**, Difference in number of infants under 6 months who are not being exclusively breastfed between 2018 and 2000, aggregated to district-level units. Maps reflect administrative boundaries, land cover, lakes and population; grey-coloured grid cells had fewer than ten people per 1 × 1-km grid cell and were classified as ‘barren or sparsely vegetated’ or were not included in this analysis^50–55^.

Four countries have more than an estimated million infants each that were not exclusively breastfed in 2018 (Fig. 5), accounting for 39.9% of the total: India (5,351,900 (4,825,700–5,904,700); 19.1% of the total non-EBF infants), Nigeria (2,899,100 (2,850,500–2,945,200); 10.4%), Pakistan (1,770,300 (1,653,400–1,889,400); 6.3%) and Brazil (1,157,000 (1,116,400–1,200,800); 4.1%). Eight additional countries each had an estimated half-million children or more that were not exclusively breastfed in 2018, accounting for 17.4% of the total non-EBF infants: Indonesia (982,200 (897,700–1,063,100); 3.5% of the total), the Philippines (782,100 (658,100–893,800); 2.8%), Mexico (760,500 (699,500–813,800); 2.7%), DRC (741,200 (623,700–848,700); 2.7%), Ethiopia (626,800 (427,600–851,000); 2.2%), Bangladesh (609,900 (402,100–832,000); 2.1%), Egypt (574,100 (480,700–670,500); 2.0%) and Vietnam (515,500 (455,800–564,800); 1.8%). Although some of these countries were close to achieving the original WHO GNT of 50% prevalence by 2018, with >45% mean national prevalence, Mexico has had low EBF prevalence scattered throughout its units and the Philippines has consistently had some of the largest subnational inequalities. Nigeria, Brazil and Vietnam have the dual complications of high geographic inequalities and relatively low national EBF prevalence (<30%).

### Projected EBF prevalence in 2025 and 2030

**Fig. 6:**
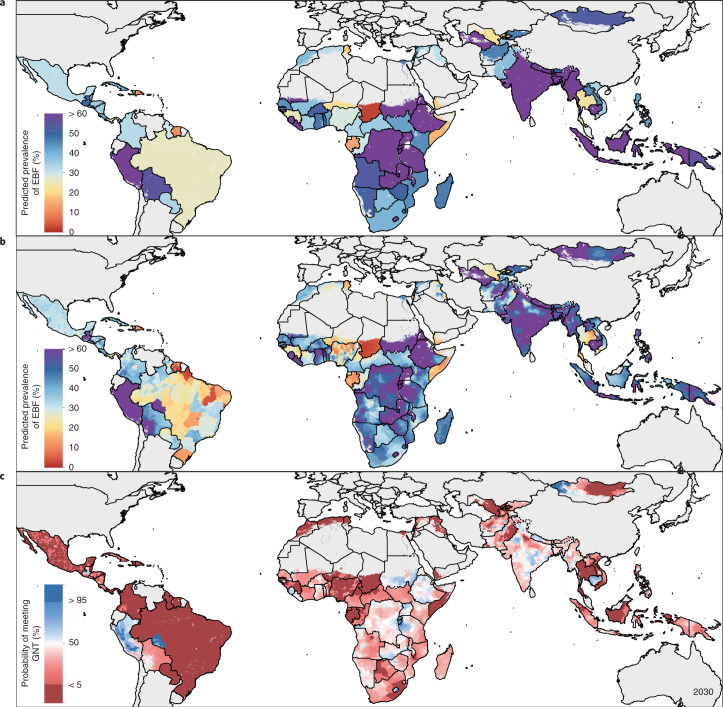
Projected prevalence for EBF for 2030 and probability of meeting the ≥70% WHO GNT by 2030. **a**,**b**, Projected EBF prevalence for 2030 at the national (**a**) and district (**b**) levels. **c**, Probability of meeting the WHO GNT of at least 70% EBF prevalence by 2030 at the district level. Dark blue indicates a high probability (>95% posterior probability) and dark red indicates a low probability (<5% posterior probability) of meeting the WHO GNT by 2030. Maps reflect administrative boundaries, land cover, lakes and population; grey-coloured grid cells had fewer than ten people per 1 × 1-km grid cell and were classified as ‘barren or sparsely vegetated’ or were not included in this analysis^50–55^.

On the basis of previous spatiotemporal historical trends and the assumption that recent trends will continue, we projected EBF estimates for the year 2025 (Supplementary Fig. 17a,b) and 2030 (Fig. 6a,b). Overall, EBF prevalence across LMICs is expected to increase from 38.7% (28.3–49.9%) in 2018 to 42.6% (25.6–60.5%) in 2025 and to reach 45.2% (23.9–67.2%) by 2030. National EBF prevalence is expected to vary by as much as 56.6 times across all LMICs (1.6% (0.5–3.8%) in Chad; 87.9% (67.4–97.0%) in Rwanda) in 2025, while within-country differences are expected to range from 1.1 to 62.9 times, with the most variation in the Philippines, Brazil and Nigeria (ninefold or more difference). By 2030, national-level prevalence is projected to vary by 71.3 times across LMICs (1.2 (0.3–3.7%) in Chad; 87.7% (59.9–98.1%) in Rwanda), with subnational variation ranging from 1.1 to 80.4 times; Brazil, the Philippines and Nigeria are expected to maintain a ninefold or greater difference between districts.

Our predictions for 2025 and 2030 show similar levels of EBF and patterns of subnational inequalities throughout LMICs as in 2018, with a few notable exceptions. On the basis of current trajectories, some of the largest projected gains are expected throughout sub-Saharan Africa. In Guinea-Bissau, Mauritania, Sierra Leone, Namibia, Zimbabwe and Gambia, most districts had <50% mean prevalence in 2018, but these countries are estimated to meet or exceed the original 50% EBF target in most districts in 2025. Outside of sub-Saharan Africa, Turkmenistan, Myanmar, Indonesia and Kyrgyzstan are also expected to exceed the 50% EBF mean prevalence target in most of their districts by 2025. Projected declines are expected to lead to districts in 15 LMICs that had mean estimates of EBF of ≥50% in 2018 to drop below this threshold by 2025; for example, Argo (Badakshan) in northeastern Afghanistan is expected to decrease from 52.4% (32.6–71.9%) in 2018 to 48.9% (17.8–60.45%) in 2025. By 2025, 33 LMICs are projected to have national mean EBF prevalence that meet the original WHO GNT of ≥50%, while 16 LMICs are predicted to have mean EBF prevalence meeting this target in all of their province-level units; 11 LMICs are expected to meet this target in all of their district-level units by 2025.

By 2030, six LMICs (Burundi, Cambodia, Lesotho, Peru, Rwanda and Sierra Leone) are projected to have mean national EBF prevalence that meet the updated WHO GNT of ≥70%, while three LMICs (Burundi, Lesotho and Rwanda) are predicted to meet this target in all their province-level and district-level units. Five LMICs (the Philippines, India, Peru, Ghana and Bolivia) had districts that met the ≥70% WHO GNT in 2018 which are expected to fall below this threshold in 2030, such as in Sandia (Puno), Peru (70.6% (51.2–88.4%) in 2018; 64.0% (33.8–87.1%) in 2030) and Mallig (Isabella), the Philippines (70.5% (54.5–83.2%) in 2018; 69.1% (38.2–90.0%) in 2030).

### Progress towards the 2030 WHO GNT of ≥70% EBF

We mapped the probabilities of meeting the updated WHO GNT of ≥70% EBF by 2018 and 2030 at various scales (Supplementary Fig. 18 and Fig. 6c). Across LMICs, 86.2% (81 of 94), 63.8% (60 of 94) and 52.1% (49 of 94) had a low probability (<5%) of having achieved the updated WHO GNT of ≥70% EBF at the national level, in all provinces, or in all districts, respectively, by 2018 (Supplementary Table 10a). Rwanda was the only LMIC that had a high probability (>95%) of having already achieved the 70% target in 2018 at the national level, as well as the only LMIC to have had a high probability of meeting the target in all province-level units. No LMIC, however, had a high probability of meeting WHO GNT of 70% in all their district-level units in 2018. Across LMICs, 84.4% (20,717 of 24,556) of districts located in 88 LMICs had a low probability, while only 1.0% (256 of 24,556) of districts in five LMICs had a high probability of having achieved the updated target of 70% by 2018. Three LMICs had districts with both high and low probability of having met the new 70% target by 2018: Brazil, Peru and the Philippines.

In analysing probabilities of meeting the updated WHO GNT of ≥70% EBF by the year 2030, most LMICs (56.4% (53 of 94)) are expected to have a low probability (<5%) of nationally achieving this goal; 23.4% (22 of 94) and 13.8% (13 of 94) of LMICs have a low probability of meeting this goal in all of their province- and district-level units, respectively (Supplementary Table 10b). No LMIC has a high probability (>95%) of meeting the ≥70% target by 2030 at the national level or in all their province- or district-level units. Across LMICs, only 0.7% (177 of 24,556) of districts located in seven LMICs have a high probability, while 59.1% (14,518 of 24,556) of districts in 56 LMICs have a low probability of meeting the ≥70% target by 2030. Extreme subnational inequalities in probabilities (both <5% and >95% probability) of achieving the 70% EBF target by 2030 are expected to occur in 3.2% (3 of 94) of LMICs: Brazil, the Philippines and Mongolia. See Supplementary Table 9 and Supplementary Fig. 17 for probabilities of meeting the original WHO GNT of ≥50% by 2025.

## Discussion

EBF practice has been known to vary by region, country and population^25–27^ but an understanding of the subnational distribution of this heterogeneity is hampered by several limitations in the previously available estimates. Previous studies have estimated EBF prevalence and interest groups such as UNICEF^26^ and Countdown to 2030^27^ have compiled EBF datasets and country profiles; some of these results have been stratified by urban–rural status or wealth quintiles or mapped at the first-administrative level (for example, states, provinces). These maps and datasets, however, are limited to select countries or years and do not allow for comparisons across countries for each year or within countries at more detailed geographic scales. Understanding subnational variation in EBF is critical to determining where increased breastfeeding support efforts are needed to lead to the most improvement. This study maps comparable subnational estimates of EBF prevalence across most LMICs over an almost 20-yr period, projects these estimates to WHO GNT target years and quantifies within-country inequalities. Not only can these estimates aid tracking progress toward WHO GNTs but also toward the United Nation’s Sustainable Development Goal (SDG)^28^ to reduce national inequalities in health opportunities and outcomes, both between and within countries, by 2030.

Although EBF is considered a cost-effective intervention, it is not free; it requires investment of time and energy from mothers and support from wider networks, including their families, communities, workplaces, health systems and government leadership. Manipulative marketing of breast-milk substitutes^29,30^, inadequate workplace support^31^, late or lack of attendance at antenatal care^32^, lack of skilled lactation support or breastfeeding counselling in health facilities^11,32^ and societal beliefs favouring mixed feeding^11,32–34^ all contribute to low rates of EBF^25,26,35^. The WHO-UNICEF Global Breastfeeding Collective (GBC) initiative stresses the need for advocacy at global, national and subnational levels to improve breastfeeding rates for the betterment of maternal and child health and wellbeing^36^. The GBC’s Breastfeeding Advocacy Toolkit outlines seven key policy actions to increase breastfeeding practices, which are: increasing funding to support EBF and continued breastfeeding to 2 years; fully adopting and monitoring the International Code of Marketing of Breast-Milk Substitutes (‘the Code’); enacting workplace breastfeeding policies and paid family leave; implementing the ‘baby-friendly’ hospital’s ‘ten steps to successful breastfeeding’; improving access to skilled breastfeeding counselling in health facilities; strengthening links between health facilities and communities to support breastfeeding; and strengthening monitoring systems to track progress^36^. Inconsistent implementation of these policies could contribute to the between- and within-country variation we see in EBF practice across LMICs. Combined with information on breastfeeding interventions, our mapped estimates can aid policy-makers in monitoring the success of breastfeeding policy and programme investments.

The World Bank estimates that an investment of US$4.70 per live newborn is needed to meet the WHO GNT for EBF by 2025^37,38^. According to the Global Breastfeeding Scorecard, however, only five LMICs in this analysis meet or exceed estimated funding needs (Guinea-Bissau, Haiti, Nepal, Somalia and Timor-Leste), while 50 spend <US$1 per live birth on breastfeeding support programmes, as of 2018^39^. Aggressive marketing of breast-milk substitutes (BMS) disrupts mothers’ informed choices by providing misleading information. In response to controversial marketing strategies, the World Health Assembly established the Code in 1981 to regulate the promotion and safety of BMS and ensure the adequate nutrition of infants^40^. The Code bans point-of-sale promotion of BMS or bottles, distribution of free samples and misleading promotional materials suggesting a product’s superiority over mother’s natural milk^40^. In 2018, however, only 24 of the 94 LMICs in this study had comprehensive Code legislation in place and 25 had no legal measures protecting consumers from aggressive BMS marketing tactics^39^. A study on global infant formulas sales showed that the steepest market increases were in Asia Pacific (18% increase) and Middle East and Africa (14% increase) regions within just 1 year (2012–2013)^30^; by 2025, the infant formula industry is expected to surpass US$98 billion in sales^41^, and increase in marketing and sales will likely negatively affect breastfeeding^42,43^. Additionally, few LMICs have national policies that satisfy the International Labour Organization’s Convention minimum recommendations for 14 weeks of paid maternity leave and appropriate workplace nursing areas; Colombia, Cuba, India, Paraguay, Tajikistan and Vietnam are the only six LMICs in our analysis that fully met these recommendations in 2018^39^. Individual breastfeeding counselling was reported to be implemented in all primary healthcare facilities in just 28 LMICs^39^. Of the LMICs with available data, at least half of births were in baby-friendly hospitals and maternities in only six countries (Costa Rica, Cuba, Eswatini, Tajikistan, Thailand and Turkmenistan)^39^. By subnational reporting, 29 LMICs in the analysis had implemented community programmes in all districts in 2018^39^. Our estimates, combined with the WHO’s Breastfeeding Scorecard Data, can be used to decide where additional resources to support breastfeeding are most needed (Supplementary Information section 5.4).

Positive exemplars in EBF uptake due to policy implementation and financial investments could provide lessons learned for policy-makers to apply towards their countries. The 2018 Global Nutrition Report spotlighted Burkina Faso’s strong commitment to supporting breastfeeding through the rapid roll-out of a national infant and young feeding programme that led to all primary healthcare facilities providing counselling and 70% of districts with community programmes for breastfeeding support^35^. Furthermore, Burkina Faso passed legislation providing 14 weeks of state-funded maternity leave and laws prohibiting advertising breast-milk substitutes^35^; by our estimates, most districts experienced >5% annualized increase in EBF over the modelled study period. In Nepal, USAID’s integrated nutrition programme combined water and sanitation, family planning and agricultural activities along with essential nutrition and breastfeeding counselling to children and caregivers in 42 of 77 districts and the recommended minimum US$4.70 per live-birth investment was met in 2018^35^; by our estimates, all districts in Nepal had annualized EBF increases between 2000 and 2018. The USAID’s programming in Malawi worked with the Ministry of Health to achieve ‘baby-friendly’ status in hospitals, develop a nutrition training for nurses and midwives and provide deworming and vitamin A supplementation^35^; these combined efforts may have contributed to many of Malawi’s districts being >50% of mean EBF prevalence in 2018. Turkmenistan’s success in achieving >5% annualized increase in all of its districts by 2018 may be attributed to the high proportion of births in baby-friendly hospitals (86.9%) and community breastfeeding programmes implemented in all its districts^39^. Gambia and Côte d’Ivoire, which had reduced absolute inequalities by at least a third, fared well on the Breastfeeding Scorecard; basic maternity provisions, as well as community programmes in all districts and counselling in all facilities were reported for Côte d’Ivoire and Gambia had full legal status of the Code, met recommended maternity leave length and all facilities offered counselling. Although we identified Cambodia as having among the highest EBF prevalence levels in 2018, and Myanmar as having among the highest annualized increases, and both countries experienced large reductions in relative inequalities, they did not have widespread supportive breastfeeding policies implemented, according to their 2018 national scorecard^39^. Additional local investigations are needed to document subnational policy implementation and determine associations between breastfeeding policies and interventions and EBF progress.

This study provides a comprehensive picture of the unmet need for EBF by mapping both prevalence of EBF and the absolute number of children not exclusively breastfed for their first 6 months of life. Our mapped estimates provide a tool to visualize subnational inequalities otherwise masked by national-level estimates and areas left behind in EBF uptake. These subnational EBF estimates can aid policy- and decision-makers in tracking progress towards the international target and in identifying where additional breastfeeding support efforts are needed to improve child health and survival. Comparisons against additional health indicators could inform the development of more comprehensive approaches to improve health in populations most in need. Future research could compare these estimates with breastfeeding policies and interventions, or lack thereof, to determine which were most successful in achieving increased practice of EBF and what barriers still need to be addressed.

## Methods

### Overview

For this study, we used a similar methodology to that of our previous work on mapping EBF prevalence in Africa^14^ and extended our scope to include all LMICs with available relevant data. LMIC status was determined by sociodemographic index (SDI), which indicates a country’s level of development on the basis of poverty, education and fertility as defined in the GBD study. Here we map estimates of countries that have low, low-middle or middle SDI status (Supplementary Table 4). We excluded several countries from our analysis despite low, low-middle or middle status due to lack of relevant input data (Cape Verde, Dominica, Djibouti, Ecuador, Grenada, Iran, Libya, Malaysia, Seychelles, Sri Lanka and Venezuela). This study complies with the Guidelines for Accurate and Transparent Health Estimates Reporting (GATHER; http://gather-statement.org; Supplementary Information section 1.0).

### Data

#### Surveys and EBF indicator data

When searching the Global Health Data Exchange (GHDx; http://ghdx.healthdata.org) for the keyword ‘breastfeeding’, we compiled an extensive geo-located dataset that includes 345 household surveys (including the Demographic and Health Surveys (DHS), Multiple Indicator Cluster Surveys (MICS) and other country-specific or multinational surveys) conducted in years 1998 to 2018 in LMICs. Of these, we assigned data from 21 surveys conducted in years 1998 or 1999 to the year 2000 to address data scarcity. This dataset represents 302,435 infants aged 0–5 months (infants up to the age of 6 months) across 94 LMICs and was geocoded to 69,179 coordinates corresponding to cluster-level boundaries and 67,750 subnational polygon boundaries. Across the 94 countries in the analysis, there were 1,727 first-administrative-level boundaries (for example, provinces) and 24,556 second-administrative-level boundaries (for example, districts). Overall inclusion criteria for surveys included: conducted in an LMIC between 1998 and 2018; responses available at the individual level; contains subnational geographic identifiers (that is, GPS coordinates, cluster or administrative units) with survey weights for each observation; and contains questions and responses about child’s age and breastfeeding status. A survey was included if it contained questions and responses regarding whether the child had consumed other food or liquids besides breast-milk. We only included observations of children who were under 6 months at the time of the survey (0–5 months). See the Supplementary Information sections 2.1 and 2.2 for further details on exclusion and inclusion criteria.

#### Spatial covariates

In these analyses, we included 11 socioeconomic and health-related covariates identified as conceivably associated with breastfeeding practices: (1) travel time to the nearest settlement >50,000 inhabitants, (2) nighttime lights^TV^, (3) population^TV^, (4) number of children under 5 yr per woman of childbearing age^TV^, (5) urban proportion of the location^TV^, (6) number of people whose daily vitamin A needs could be met, (7) educational attainment in women of reproductive age (15–49-years-old)^TV^, (8) human development index (HDI)^TV^, (9) human immunodeficiency virus (HIV) prevalence^TV^, (10) healthcare access and quality index^TV^ and (11) proportion of pregnant women who received four or more antenatal care visits^TV^ (where superscript TV indicates time-varying covariates). Of these, the covariates for the Healthcare Access and Quality Index^44^ and the proportion of pregnant women who received four or more antenatal care visits^45^ were indexed at the national level, while all others were indexed at the subnational level. The spatial covariates were selected because they are factors or proxies for factors that previous literature has identified to be associated (not necessarily causally) with EBF prevalence.

Variance inflation factor^46^ (VIF) analysis was used to filter covariates for multicollinearity. We performed temporal processing for covariates that did not have information for every year of the modelled study period and filled in intervening years with the value from the nearest neighbouring year or used an exponential growth rate model. Detailed information on covariates can be found in Supplementary Table 5 and Supplementary Fig. 8.

### Analysis

The technical descriptions of methods for the underlying geostatistical model, model validation and postestimation are consistent with those previously used in the geospatial modelling of EBF across Africa^14^.

#### Geostatistical model

**Extended Data Fig. 1:**
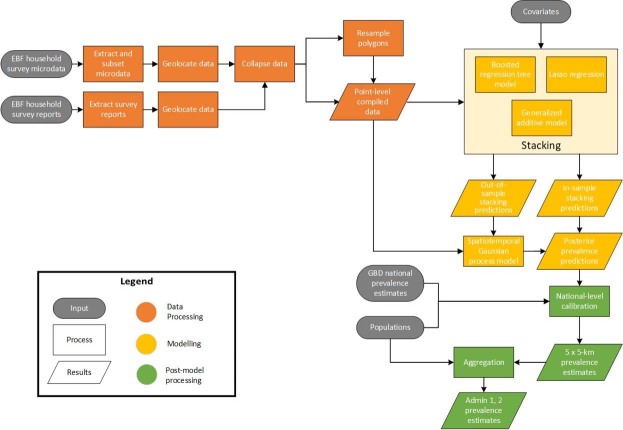
Analytic process overview. The process used to produce EBF prevalence estimates across LMICs involved three main parts. In the data-processing steps (orange), data were identified, extracted, and prepared for use in the models. In the modelling phase (yellow), we used these data and covariates in stacked generalization ensemble models and spatiotemporal Gaussian process models for each EBF indicator. In post-processing (green), we calibrated the prevalence estimates to match the GBD 2019^12^ study estimates and aggregated the estimates to the first- and second-administrative levels in each country.

EBF was modelled using a Bayesian geostatistical approach. This approach uses a hierarchical logistic regression model that is spatially and temporally explicit, and assumes points close in space and time and with similar covariate patterns will have similar levels of EBF. Using a stacked generalization technique, we also incorporated potential nonlinear relationships between covariates and EBF input data. For all model parameters and hyper-parameters, we used the R-INLA statistical package to approximate posterior distributions^47^. We used 1,000 draws from these approximate joint posterior distributions to calculate uncertainty intervals (UI), determining and reporting the 2.5th and 97.5th percentiles of those 1,000 draws. Further details on methodology can be found in Supplementary Information section 4.0. Extended Data Fig. 1 provides an overview of analytical processing steps involved in the analysis.

#### Model validation

We used fivefold cross validation to validate models, as summarized below. Complete methods used for validation and related results are available in the Supplementary Information. First, we combined randomized sets of cluster-level data points at the first-administrative level to create holdout sets. Afterwards, we fit the geostatistical model five times, sequentially excluding each of the five groups of data, and provided out-of-sample predictions that correspond to all included surveys in the analysis. We summarized the performance of the model using 95% data coverage within prediction intervals, correlation between predictions and observed data and the mean error (a measure of bias) and root-mean-square error (a measure of total variance). Model estimates were also compared with other existing estimates, as possible.

#### Postestimation

**Extended Data Fig. 2:**
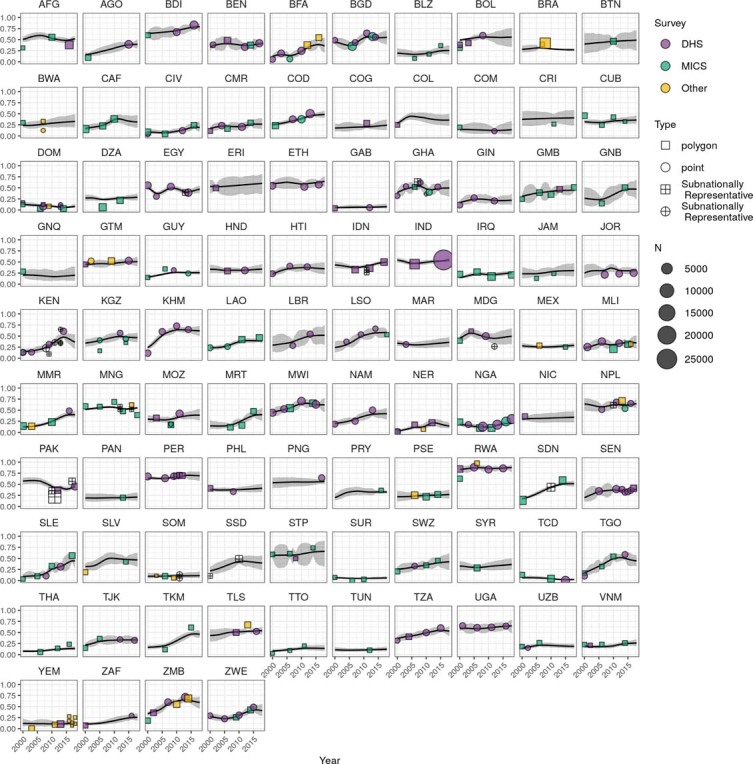
National time series plots and aggregated input data. National time series plots of the post-GBD calibration final estimates by country during 2000–2018. Uncertainty ranges are presented in grey, and aggregated input data are classified by survey series (purple for country-specific, green for DHS, and yellow for MICS surveys), data type (square for polygon, circle for point data), and whether the survey is nationally or subnationally representative).

To estimate EBF prevalence at various levels (province, district and country), we aggregated each of the 1,000 draws of coverage at the 5 × 5-km grid-cell level, weighted by population. We preformed posthoc calibration of our estimates to the GBD 2019 estimates^12^. This allowed us to include data sources outside of the scope of our geospatial modelling framework. On the basis of the estimates, we calculated absolute differences between lowest and highest administrative units and relative differences between a country’s average and each administrative unit in that country to quantify geographic inequality. We performed a simple projection calculation by comparing the estimated rates of EBF improvement between 2000 and 2018 with the improvements needed between 2018 and 2030 to meet the WHO GNT (Supplementary Information section 4.4.4). The national time series and aggregated input data in our estimates can be found in Extended Data Fig. 2.

#### Modelling limitations

The modelling limitations in this work are consistent with those previously described in the geospatial modelling of EBF across Africa^14^.

While we have attempted to propagate uncertainty from various sources through the different modelling stages, there are some sources of uncertainty that have not been propagated. In particular, it was not computationally feasible to propagate uncertainty from the submodels in stacking through the geostatistical model. Similarly, although the WorldPop population raster is also composed of estimates associated with some uncertainty, this uncertainty is difficult to quantify and not currently reported and we were unable to propagate this uncertainty into our estimates of EBF prevalence for administrative units that were created using population-weighted averages of grid-cell estimates. Model fitting was carried out using an integrated nested Laplace approximation to the posterior distribution, as implemented in the R-INLA package^47^. Prediction from fitted models was subsequently carried out using the inla.posterior.sample() function, which generates samples from the approximated posterior of the fitted model. Both model fitting and prediction thus require approximations, and these approximations may introduce error.

**Extended Data Fig. 3:**
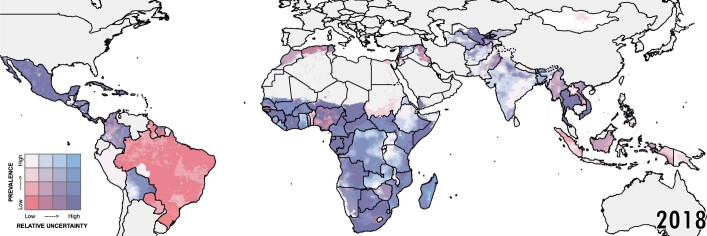
Relative uncertainty in EBF estimates for 2018. Relative uncertainty in second-administrative-level estimates compared with mean estimated EBF prevalence in each second-administrative-level unit for 2018. Mean prevalence and relative uncertainty are split into population-weighted quartiles. These cut-off points for relative uncertainty (calculated as the absolute range of the uncertainty intervals divided by the estimate) are 0.684 (25th percentile), 0.916 (50th percentile), and 1.271 (75th percentile), respectively. The cut-off points for EBF prevalence are 25.8% (25th percentile), 35.4% (50th percentile), and 49.4% (75th percentile), respectively. Units in which our estimates are more uncertain are coloured with a scale of increasing blue hue, whereas areas in which the mean estimates of EBF are low are coloured with a scale of increasing red hue. Purple areas have low, but uncertain, estimates of EBF. White areas have high EBF estimates that are fairly certain. Relative uncertainty is defined as the ratio of the width of the 95% uncertainty interval to mean estimate. Maps reflect administrative boundaries, land cover, lakes, and population; grey-coloured grid cells had fewer than ten people per 1 × 1-km grid cell and were classified as ‘barren or sparsely vegetated’, or were not included in this analysis^50–55^.

To estimate projections of EBF prevalence levels in 2025 and 2030, we used previous historical trends and the assumption that recent trends will continue. These assumptions in turn lend to modelling limitations, as we were not able to project underlying drivers of changes in EBF, such as increasing urbanization or changes in population^35,48,49^, and the certainty of our estimates and projections were critically dependent on data quality and availability. Availability of relevant data varies both spatially and temporally across LMICs (Supplementary Figs. 1–5) and lack of relevant data is one of the main sources of uncertainty around our estimates (as seen in Extended Data Fig. 3). We have mapped EBF prevalence levels against the relative uncertainty of our estimates in Extended Data Fig. 3.

### Reporting Summary

Further information on research design is available in the Nature Research Reporting Summary linked to this article.

## Supplementary information

Supplementary Information Supplementary Figs. 1–23, Supplementary Tables 1–12, Supplementary Methods, Supplementary Results, Supplementary Limitations, Supplementary Discussion, Supplementary References; Author Contributions.

Reporting Summary

Peer review information

## Data Availability

The information given here is mostly consistent with our previous study modelling EBF across Africa^14^. The findings of this study are supported by data that are available in public online repositories, data that are publicly available on request from the data provider and data that are not publicly available due to restrictions by the data provider and which were used under license for the current study. Details on data sources can be found on the GHDx website (http://ghdx.healthdata.org/lbd-publication-data-input-sources?field_rec_ihme_publication_tid=29093), including information about the data provider and links to where the data can be accessed or requested (where available). We have also provided maps of the data included in our models in Supplementary Figs. 1–5. Outputs of these EBF analyses can be explored at various spatial levels (national, administrative and 5 × 5-km levels) through our customized visualization tool (https://vizhub.healthdata.org/lbd/ebf) and are publicly available at the GHDx (http://ghdx.healthdata.org/record/ihme-data/global-exclusive-breastfeeding-prevalence-geospatial-estimates-2000-2019). Administrative boundaries were retrieved from the Database of Global Administrative Areas (GADM)^50^. Land cover was retrieved from the online Data Pool, courtesy of the NASA EOSDIS Land Processes Distributed Active Archive Center, USGS/Earth Resources Observation and Science Center, Sioux Falls, South Dakota^51^. Lakes were retrieved from the Global Lakes and Wetlands Database, courtesy of the World Wildlife Fund and the Center for Environmental Systems Research, University of Kassel^52^. Populations were retrieved from WorldPop^53^. All maps in this study were produced using ArcGIS Desktop 10.6.

## Code availability

All codes used for these analyses are publicly available online at https://github.com/ihmeuw/lbd/tree/ebf-lmic-2021.
