# Supplementary material for: Mapping inequalities in exclusive breastfeeding in low- and middle-income countries, 2000–2018
Source: Nat Hum Behav. 2021 Jun 3;5(8):1027–45. doi: 10.1038/s41562-021-01108-6 (PMC8373614; doi:10.1038/s41562-021-01108-6)
Supplement: Supplementary file 1 — Supplementary Figs. 1–23, Supplementary Tables 1–12, Supplementary Methods, Supplementary Results, Supplementary Limitations, Supplementary Discussion, Supplementary References; Author Contributions. [file 41562_2021_1108_MOESM1_ESM.pdf]

nature portfolio

https://doi.org/10.1038/s41562-021-01108-6

Supplementary information

Mapping inequalities in exclusive breastfeeding in low- and middle-income countries, 2000–2018

In the format provided by the authors and unedited

Contents

Supplementary Figures 2

Supplementary Tables 3

1.0. Compliance with the Guidelines for Accurate and Transparent Health Estimates Reporting (GATHER) 4

2.0. Data Sources and Processing 6

2.1. Data excluded from model 26
2.2. Data processing 26
2.3. Geographic inclusion 30

3.0. Covariates 33

4.0. Statistical model 40

4.1. Ensemble covariate modelling process 40
4.2. Geostatistical model 41
4.3. Model validation 47
4.4. Post-estimation 52
4.4.1. Calibration to Global Burden of Disease 2019 52
4.4.2. Aggregation to first- and second-level administrative units 53
4.4.3. Geographic Inequality 53
4.4.4. Projections 53

5.0. Supplementary Results 55

5.1. National differences in rates of change from 2000 to 2018 55
5.2. Achievement of the original WHO GNT (50% EBF) by 2018 and 2025 56
5.3. Achievement of the updated WHO GNT (70% EBF) by 2030 62
5.4. Global Breastfeeding Scorecard (GBS) Exemplars 67
5.5. Comparison of EBF with respect to other key indicators 69

6.0. Limitations 76

7.0. Supplementary Discussion 78

8.0. Collaborators and Affiliations 79

9.0. Author Contributions 93

10.0. Supplementary References 98

Supplementary Figures

Supplementary Figure 1. Data availability in Africa (with Yemen) for EBF among infants under 6 months by type and country, 1998–2018 7
Supplementary Figure 2. Data availability in Central Asia and Middle East for EBF among infants under 6 months by type and country, 1998–2018 8
Supplementary Figure 3. Data availability in Southeast Asia and Oceania for EBF among infants under 6 months by type and country, 1998–2018 9
Supplementary Figure 4. Data availability in South Asia for EBF among infants under 6 months by type and country, 1998–2018 10
Supplementary Figure 5. Data availability in Latin America for EBF among infants under 6 months by type and country, 1998–2018 11
Supplementary Figure 6. Flowchart for data extraction (a) and data cleaning (b) processes 28
Supplementary Figure 7. Countries included in this analysis and modeling regions 30
Supplementary Figure 8. Map of spatial covariates 38
Supplementary Figure 9. Example of finite elements mesh for geostatistical models 43
Supplementary Figure 10. Posterior means and 95% uncertainty intervals for EBF prevalence by 5 × 5-km level in 2018. 44
Supplementary Figure 11. Posterior means and 95% uncertainty intervals for EBF prevalence by the first administrative level in 2018. 45
Supplementary Figure 12. Posterior means and 95% uncertainty intervals for EBF prevalence by the second administrative level in 2018. 46
Supplementary Figure 13. In-sample comparison of data and estimates, aggregated to the national level and year 48
Supplementary Figure 14. In-sample comparison of data and estimates, aggregated to the first administrative level and year 49
Supplementary Figure 15. In-sample comparison of data and estimates, aggregated to the second administrative level and year 50
Supplementary Figure 16. Probability of meeting the ≥50% WHO GNT for EBF in 2018 57
Supplementary Figure 17. Projected prevalence for exclusive breastfeeding for 2025 and probability of meeting the WHO GNT by 2025 58
Supplementary Figure 18. Probability of meeting the ≥70% WHO GNT for EBF in 2018 62
Supplementary Figure 19. Comparison of ORS (oral rehydration solution) prevalence among children under 5 years and EBF prevalence by area 69
Supplementary Figure 20. Comparison of access to piped water and EBF prevalence by area ... 70
Supplementary Figure 21. Comparison of diarrhea prevalence among children under 5 years and EBF prevalence by area 71
Supplementary Figure 22. Comparison of stunting prevalence among children under 5 years and EBF prevalence by area 72
Supplementary Figure 23. Comparison of mortality rate of children under 5 years (U5MR) and EBF prevalence by area 73

Supplementary Tables

Supplementary Table 1. Data excluded from both the geostatistical model and GBD estimates 12
Supplementary Table 2. Data excluded from GBD estimates but included in geostatistical model 14
Supplementary Table 3. Data excluded from geostatistical model but included in GBD estimates 19
Supplementary Table 4. Countries included in the analysis (94) grouped by modelling regions. 31
Supplementary Table 5. Sources for covariates used in mapping. 35
Supplementary Table 6. Covariates used in ensemble covariate modelling via stacked generalization, stratified by modeling region 39
Supplementary Table 7. Validation metrics by level of aggregation........ 51
Supplementary Table 8. Countries with annualized increases and decreases in all districts........ 55
Supplementary Table 9. Countries and administrative units achieving the original WHO GNT of 50% prevalence of EBF with high and low probabilities 59
Supplementary Table 10. Countries and administrative units achieving the updated WHO GNT of 70% prevalence of EBF with high and low probabilities........ 63
Supplementary Table 11. Countries meeting and not meeting GBS[44] criteria 67
Supplementary Table 12. First administrative-level units with the lowest decile of EBF prevalence, as well as either the lowest decile of oral rehydration solution (ORS) coverage, highest prevalence of child diarrheal disease, highest decile of child stunting prevalence, or highest under-5 mortality rates, for year 2017........ 74

1.0. Compliance with the Guidelines for Accurate and Transparent Health Estimates Reporting (GATHER)

Item #	Checklist item	Description of Compliance
**Objectives and funding**		
**1**	Define the indicator(s), populations (including age, sex, and geographic entities), and time period(s) for which estimates were made.	Summary; Introduction
**2**	List the funding sources for the work.	End Notes
**Data Inputs**		
For all data inputs from multiple sources that are synthesized as part of the study:		
**3**	Describe how the data were identified and how the data were accessed.	Methods (Data); SI section 2
**4**	Specify the inclusion and exclusion criteria. Identify all ad-hoc exclusions.	SI section 2; Supplementary Tables 1–3
**5**	Provide information on all included data sources and their main characteristics. For each data source used, report reference information or contact name/institution, population represented, data collection method, year(s) of data collection, sex and age range, diagnostic criteria or measurement method, and sample size, as relevant.	Supplementary Figures 1–5; List of included data sources provided through http://ghdx.healthdata.org/lbd-publication-data-input-sources?field_rec_ihme_publication_tid=29093
**6**	Identify and describe any categories of input data that have potentially important biases (e.g., based on characteristics listed in item 5).	SI section 2.2
For data inputs that contribute to the analysis but were not synthesized as part of the study:		
**7**	Describe and give sources for any other data inputs.	SI section 3, Supplementary Table 5
For all data inputs:		
**8**	Provide all data inputs in a file format from which data can be efficiently extracted (e.g., a spreadsheet rather than a PDF), including all relevant meta-data listed in item 5. For any data inputs that cannot be shared because of ethical or legal reasons, such as third-party ownership, provide a contact name or the name of the institution that retains the right to the data.	Available through http://ghdx.healthdata.org/lbd-publication-data-input-sources?field_rec_ihme_publication_tid=29093

Data analysis		
**9**	Provide a conceptual overview of the data analysis method. A diagram may be helpful.	Methods (Analysis), Extended Data Figure 1; SI section 2; Supplementary Figure 6
**10**	Provide a detailed description of all steps of the analysis, including mathematical formulae. This description should cover, as relevant, data cleaning, data pre-processing, data adjustments and weighting of data sources, and mathematical or statistical model(s).	Methods; SI sections 4
**11**	Describe how candidate models were evaluated and how the final model(s) were selected.	SI section 4.3; Supplementary Figures 13–15, Supplementary Table 7
**12**	Provide the results of an evaluation of model performance, if done, as well as the results of any relevant sensitivity analysis.	SI section 4.3; Supplementary Table 7
**13**	Describe methods for calculating uncertainty of the estimates. State which sources of uncertainty were, and were not, accounted for in the uncertainty analysis.	Methods (Geostatistical model); SI sections 4 and 6
**14**	State how analytic or statistical source code used to generate estimates can be accessed.	Available through https://github.com/ihmeuw/lbd/tree/ebf-lmic-2021
**Results and Discussion**		
**15**	Provide published estimates in a file format from which data can be efficiently extracted.	Available through http://ghdx.healthdata.org/record/ihme-data/global-exclusive-breastfeeding-prevalence-geospatial-estimates-2000-2019
**16**	Report a quantitative measure of the uncertainty of the estimates (e.g., uncertainty intervals).	Supplementary Figures 10–12, Extended Data Figure 3
**17**	Interpret results in light of existing evidence. If updating a previous set of estimates, describe the reasons for changes in estimates.	Discussion
**18**	Discuss limitations of the estimates. Include a discussion of any modelling assumptions or data limitations that affect interpretation of the estimates.	Methods (Limitations); SI section 6

2.0. Data Sources and Processing

The data sources used to model EBF are described below. Information on geographic detail, the citation(s) and name(s) of the survey(s) used in the mapping of EBF prevalence among infants under 6 months in low- and middle- income countries (LMICs) can be downloaded through the GHDx website (http://ghdx.healthdata.org/lbd-publication-data-input-sources?field_rec_ihme_publication_tid=29093).

Out of 349 surveys, 162 were from the Demographic and Health (DHS) series, 156 from the UNICEF Multiple Indicator Cluster Survey (MICS) series, and 31 from other sources. Supplementary Figures 1–5 show the spatial and temporal extent of data availability by country.

Supplementary Information (SI) Section 2.1 provides detailed information on surveys that were not included in the modelling (Supplementary Tables 1–3). Although they are categorized as LMICs, we do not estimate for Libya, Djibouti, Ecuador, Venezuela, Malaysia, Sri Lanka, Iran, or Dominica for which no data were identified meeting the inclusion and exclusion criteria described below (Sections 2.1 and 2.2), nor do we estimate for island nations where survey data were not available (Mauritius, Seychelles, and Cape Verde). Supplementary Figure 6 describes the detailed steps performed during data extraction and data processing workflow.

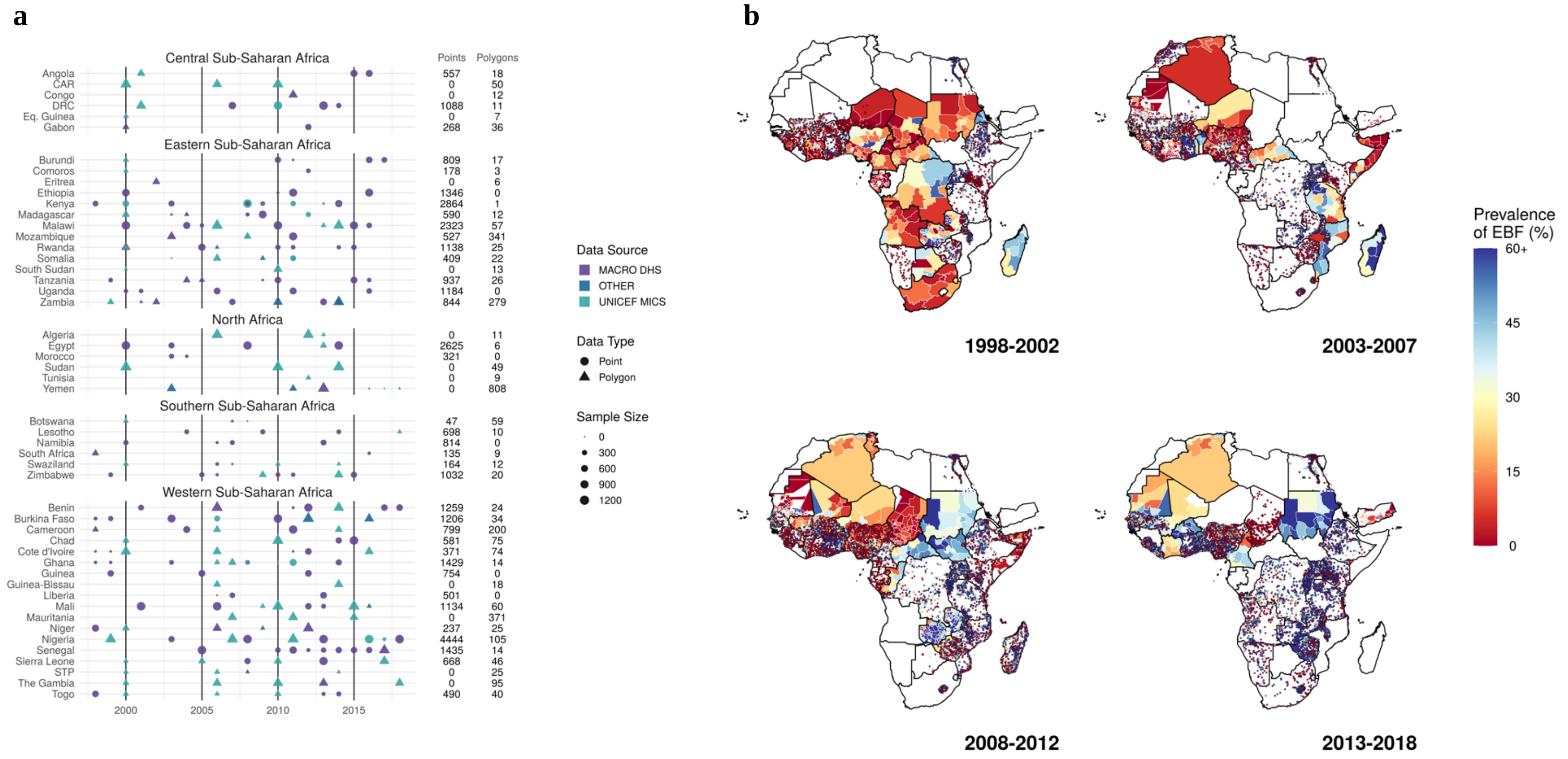

Supplementary Figure 1. Data availability in Africa (with Yemen) for EBF among infants under 6 months by type and country, 1998–2018

a, EBF data used in this study for Africa by country. Color indicates the data source: DHS; MICS; or other survey type. Shape type indicates whether a data source has point (GPS) or polygon (for example, aggregated to an administrative level) location information. Size indicates the relative effective sample size for each source. A full list of data sources, with additional details about data type (such as survey microdata and survey reports) and geographic details, is provided through the GHDx website (http://ghdx.healthdata.org/lbd-publication-data-input-sources?field_rec_ihme_publication_tid=29093). **b,** Maps of EBF data coverage displayed at 5-year intervals. Maps show the spatial resolution of the underlying data in our models, and the color indicates the EBF prevalence as estimated from the data sources. Countries in white have no available survey data in the given time range.

a

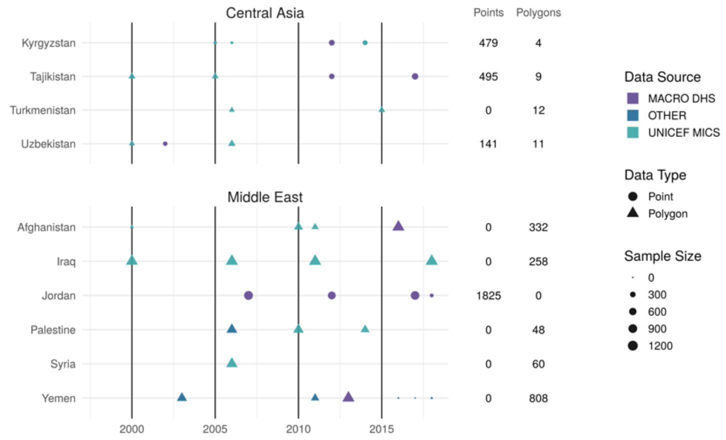

b

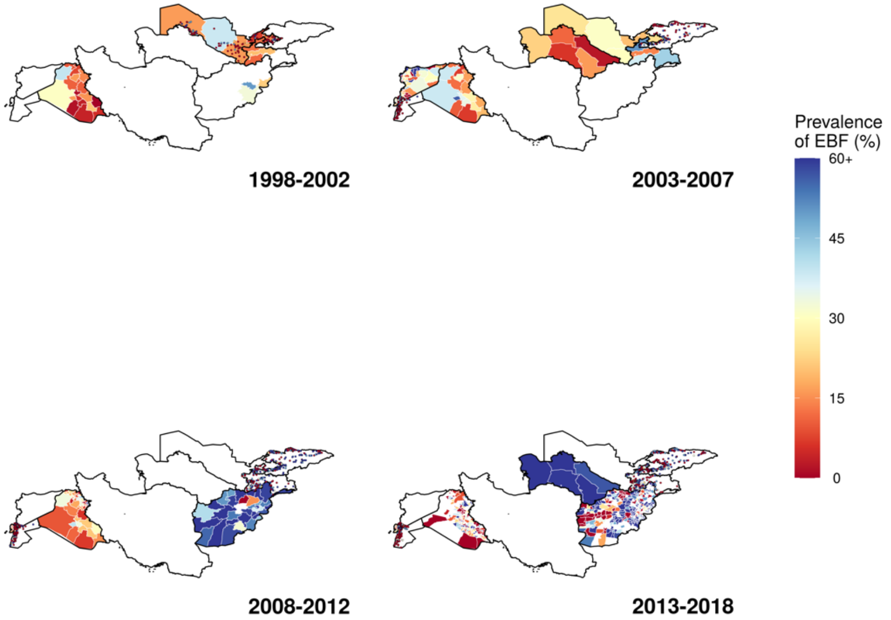

Supplementary Figure 2. Data availability in Central Asia and Middle East for EBF among infants under 6 months by type and country, 1998–2018

a, EBF data used in this study for Central Asia and Middle East by country. Color indicates the data source: DHS; MICS; or other survey type. Shape type indicates whether a data source has point (GPS) or polygon (for example, aggregated to an administrative level) location information. Size indicates the relative effective sample size for each source. A full list of data sources, with additional details about data type (such as survey microdata and survey reports) and geographic details, is provided through the GHDx website (http://ghdx.healthdata.org/lbd-publication-data-input-sources?field_rec_ihme_publication_tid=29093). **b,** Maps of EBF data coverage displayed at 5-year intervals. Maps show the spatial resolution of the underlying data in our models, and the color indicates the EBF prevalence as estimated from the data sources. Countries in white have no available survey data in the given time range.

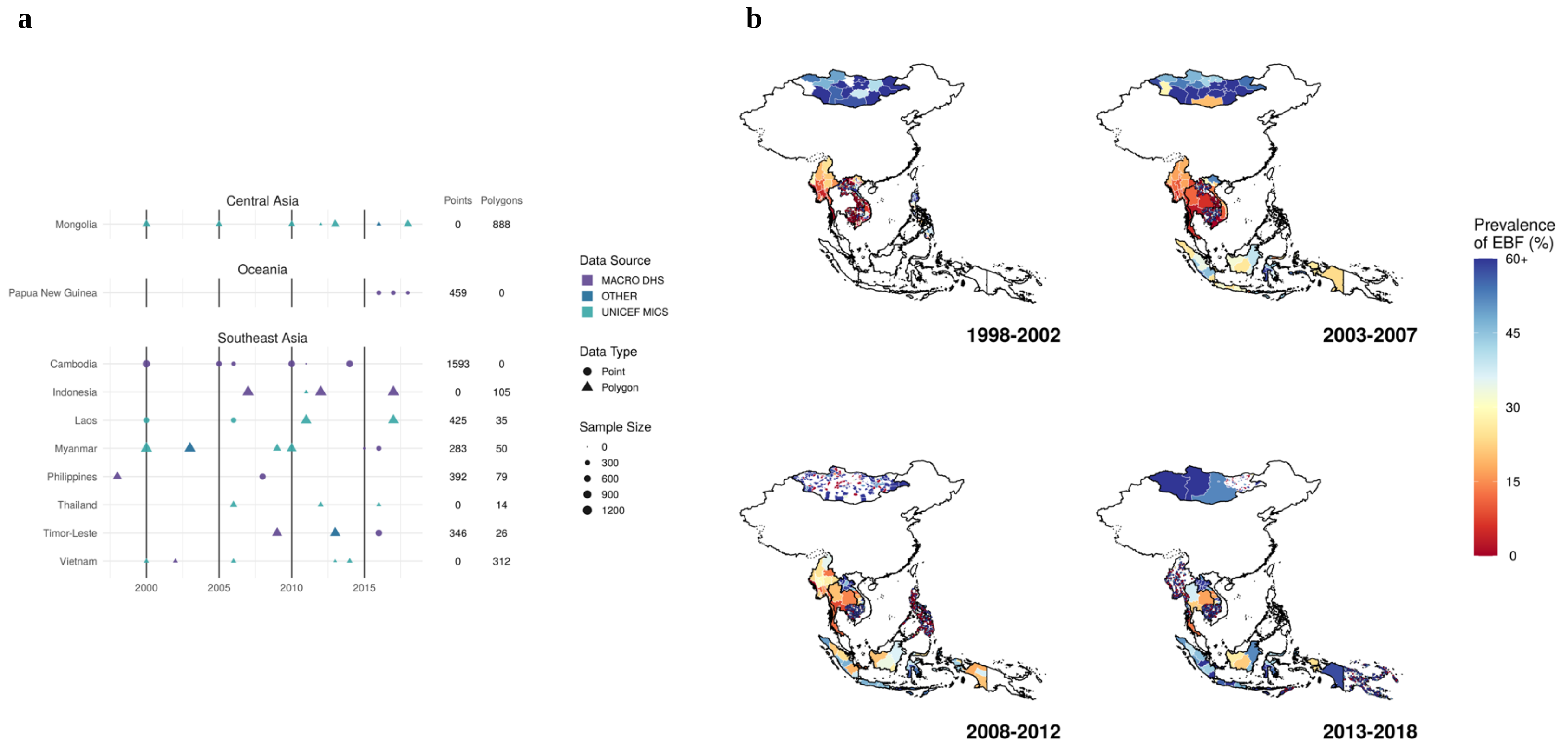

Supplementary Figure 3. Data availability in Southeast Asia and Oceania for EBF among infants under 6 months by type and country, 1998–2018

a, EBF data used in this study for Southeast Asia and Oceania by country. Color indicates the data source: DHS; MICS; or other survey type. Shape type indicates whether a data source has point (GPS) or polygon (for example, aggregated to an administrative level) location information. Size indicates the relative effective sample size for each source. A full list of data sources, with additional details about data type (such as survey microdata and survey reports) and geographic details, is provided through the GHDx website (http://ghdx.healthdata.org/lbd-publication-data-input-sources?field_rec_ihme_publication_tid=29093). **b,** Maps of EBF data coverage displayed at 5-year intervals. Maps show the spatial resolution of the underlying data in our models, and the color indicates the EBF prevalence as estimated from the data sources. Countries in white have no available survey data in the given time range.

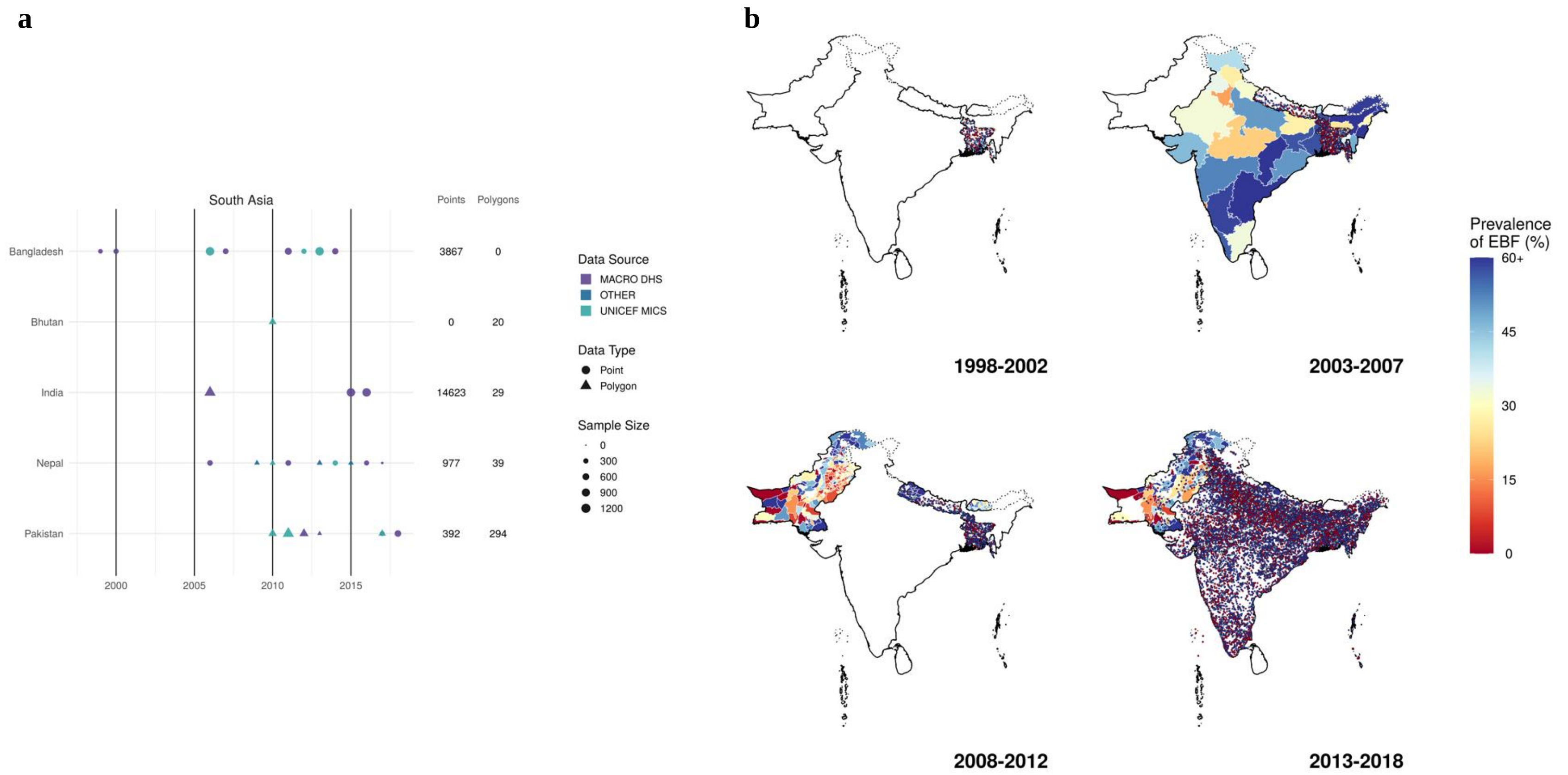

Supplementary Figure 4. Data availability in South Asia for EBF among infants under 6 months by type and country, 1998–2018

a, EBF data used in this study for South Asia by country. Color indicates the data source: DHS; MICS; or other survey type. Shape type indicates whether a data source has point (GPS) or polygon (for example, aggregated to an administrative level) location information. Size indicates the relative effective sample size for each source. A full list of data sources, with additional details about data type (such as survey microdata and survey reports) and geographic details, is provided through the GHDx website (http://ghdx.healthdata.org/lbd-publication-data-input-sources?field_rec_ihme_publication_tid=29093). **b**, Maps of EBF data coverage displayed at 5-year intervals. Maps show the spatial resolution of the underlying data in our models, and the color indicates the EBF prevalence as estimated from the data sources. Countries in white have no available survey data in the given time range.

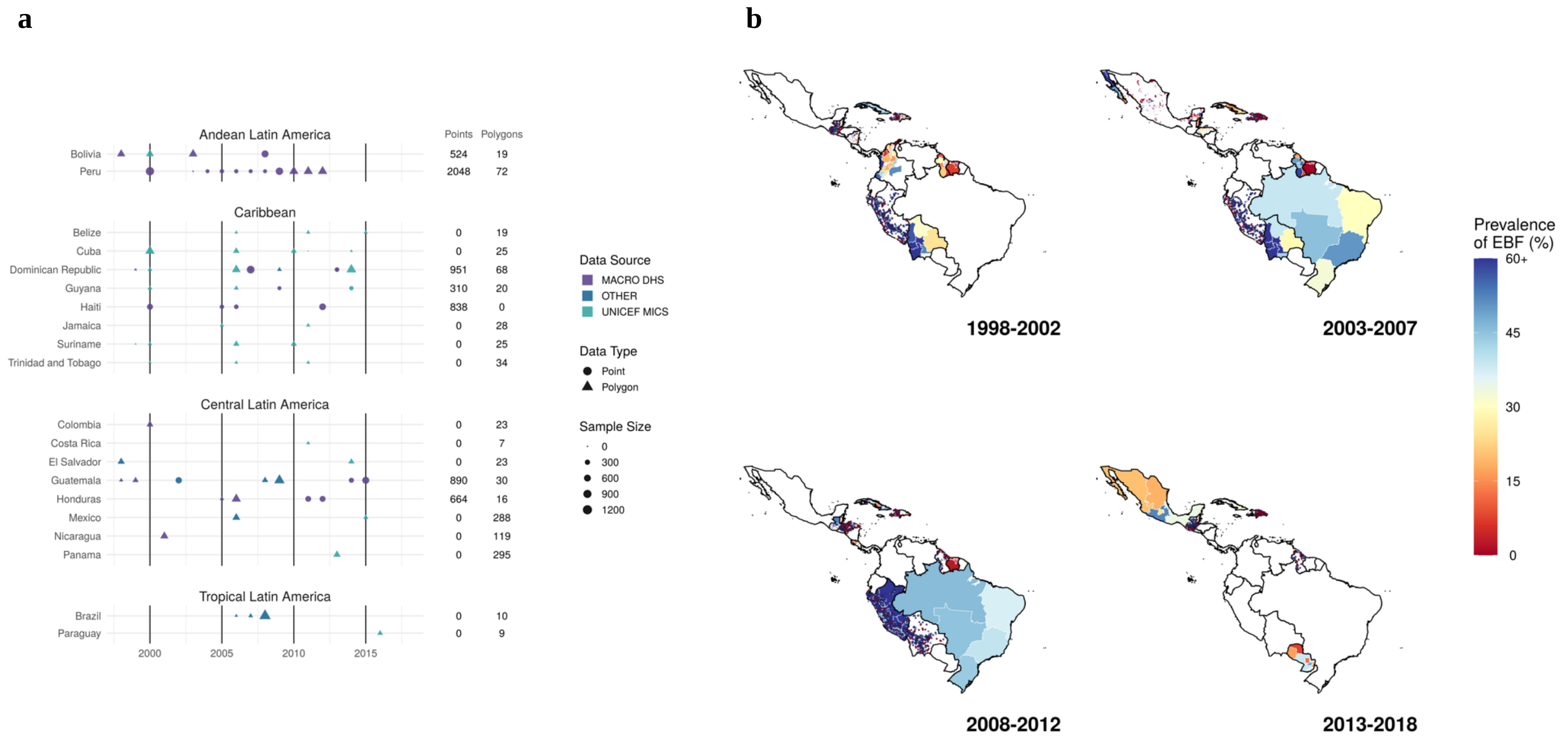

Supplementary Figure 5. Data availability in Latin America for EBF among infants under 6 months by type and country, 1998–2018

a, EBF data used in this study for Latin America by country. Color indicates the data source: DHS; MICS; or other survey type. Shape type indicates whether a data source has point (GPS) or polygon (for example, aggregated to an administrative level) location information. Size indicates the relative effective sample size for each source. A full list of data sources, with additional details about data type (such as survey microdata and survey reports) and geographic details, is provided through the GHDx website (http://ghdx.healthdata.org/lbd-publication-data-input-sources?field_rec_ihme_publication_tid=29093). **b,** Maps of EBF data coverage displayed at 5-year intervals. Maps show the spatial resolution of the underlying data in our models, and the color indicates the EBF prevalence as estimated from the data sources. Countries in white have no available survey data in the given time range.

Supplementary Table 1. Data excluded from both the geostatistical model and GBD estimates

Country	Series	Year(s)	Citation	NID[♦]	Rationale for exclusion
Ethiopia	LSMS	2015–2016	Central Statistical Agency (Ethiopia), World Bank. Ethiopia Socioeconomic Survey 2015–2016. Washington DC, United States: World Bank, 2015.	286657	Estimates considered implausible (zero values)
Mali	Multiple Indicator Cluster Survey (MICS)	2009–2010	Ministry of Health (Mali), National Institute of Statistics (INSTAT) (Mali), United Nations Children's Fund (UNICEF). Mali Multiple Indicator Cluster Survey 2009–2010. New York, United States: United Nations Children's Fund (UNICEF), 2017.	270627	Survey estimates are systematically low compared to estimates from other established survey series (2006 DHS, 2012 DHS)
Mali	LSMS	2014–2015	Ministry of Rural Development (Mali), National Institute of Statistics (INSTAT) (Mali), World Bank. Mali Agricultural Integrated Economic Survey 2014–2015. Washington DC, United States: World Bank.	260407	Survey estimates are implausibly high compared to estimates from other established survey series (2012 DHS)
Nigeria	Core Welfare Indicators Questionnaire Survey (CWIQ)	2006–2007	National Bureau of Statistics (Nigeria). Nigeria Core Welfare Indicators Questionnaire Survey 2006. Abuja, Nigeria: National Bureau of Statistics (Nigeria).	9522	Survey estimates are systematically high compared to administrative-level estimates and estimates from other established survey series (2008 DHS, 2007 MICS).
Senegal	Multiple Indicator Cluster Survey (MICS)	2015–2016	National Agency of Statistics and Demography (Senegal), United Nations Children's Fund (UNICEF). Senegal — Dakar Urban Multiple Indicator Cluster Survey 2015–2016. New York, United States: United Nations Children's Fund (UNICEF), 2018.	287639	Estimates considered implausible (zero values).
Uganda	LSMS	2010–2011	Uganda Bureau of Statistics. Uganda Living Standards Measurement Survey — Integrated Survey on Agriculture 2010–2011. Washington DC, United States: World Bank.	142934	Survey estimates are systematically high compared to estimates from other

Country	Series	Year(s)	Citation	NID♦	Rationale for exclusion
					established survey series (2006 DHS, 2011 DHS, 2016 DHS)
Uganda	LSMS	2011–2012	Uganda Bureau of Statistics. Uganda Living Standards Measurement Survey — Integrated Survey on Agriculture 2010–2011. Washington DC, United States: World Bank.	142935	Survey estimates are systematically high compared to estimates from other established survey series (2006 DHS, 2011 DHS, 2016 DHS)
Zambia	LSMS	1998	Central Statistical Office (Zambia), London School of Hygiene and Tropical Medicine. Zambia Living Conditions Monitoring Survey 1998. Lusaka, Zambia: Central Statistical Office (Zambia).	14015	Estimates considered implausible (zero values).
Zambia	Zambia Living Conditions Monitoring Survey	2002–2003	Central Statistical Office (Zambia). Zambia Living Conditions Monitoring Survey 2002–2003. Lusaka, Zambia: Central Statistical Office (Zambia).	14027	Estimates considered implausible (zero values).

♦NID = Data source unique identifier in the Global Health Data Exchange (GHDx) (http://ghdx.healthdata.org/). Additional information about each data source is available via the GHDx, including information about the data provider and links to where the data can be accessed or requested (where available). NIDs can be entered in the search bar to retrieve the record for a particular source.

Supplementary Table 2. Data excluded from GBD estimates but included in geostatistical model

Country	Series	Year(s)	Citation	NID♦	Rationale for exclusion
Brazil	Brazil Survey of Prevalence of Breastfeeding in Capitals and the Federal District	2008	Ministry of Health (Brazil). Brazil Survey of Prevalence of Breastfeeding in Capitals and the Federal District 2008.	233960	Not extracted
Dominican Republic	Multiple Indicator Cluster Survey (MICS)	2006	National Statistics Office (Dominican Republic), United Nations Children's Fund (UNICEF). Dominican Republic Multiple Indicator Cluster Survey 2006.	3465	Not extracted
Dominican Republic	Dominican Republic National Multipurpose Household Survey	2009	International Labour Organization (ILO), National Statistics Office (Dominican Republic), United Nations Children's Fund (UNICEF). Dominican Republic National Multipurpose Household Survey 2009–2010. 2011.	65416	Not extracted
India	India National Health Profile	2005	Central Bureau of Health Intelligence (India). India National Health Profile 2011. New Delhi, India: Central Bureau of Health Intelligence (India), 2012.	59322	Not extracted
Iraq	Multiple Indicator Cluster Survey (MICS)	2018	Central Statistical Organization (Iraq), United Nations Children's Fund (UNICEF). Iraq Multiple Indicator Cluster Survey 2018. New York, United States of America: United Nations Children's Fund (UNICEF), 2019.	385708	Released after GBD 2019 data addition deadline
Laos	Multiple Indicator Cluster Survey (MICS), DHS Standard Demographic and Health Survey (DHS), Lao Social Indicator Survey	2017	Lao Statistics Bureau, Ministry of Education and Sports (Laos), Ministry of Health (Laos), United Nations Children's Fund (UNICEF). Laos Multiple Indicator Cluster Survey 2017. New York, United States of America: United Nations Children's Fund (UNICEF), 2018.	375362	Not extracted

	(LSIS), DHS Program Surveys				
Madagascar	Multiple Indicator Cluster Survey (MICS)	2012	National Institute of Statistics (Madagascar), United Nations Children's Fund (UNICEF). Madagascar — South Multiple Indicator Cluster Survey 2012. New York, United States of America: United Nations Children's Fund (UNICEF), 2015.	125594	Not nationally representative. Only sampled the south of Madagascar.
Mali	Multiple Indicator Cluster Survey (MICS)	2015	Ministry of Health (Mali), Ministry of Planning (Mali), National Institute of Statistics (INSTAT) (Mali), United Nations Children's Fund (UNICEF). Mali Multiple Indicator Cluster Survey 2015. New York, United States of America: United Nations Children's Fund (UNICEF), 2017.	248224	Not extracted
Mali	Multiple Indicator Cluster Survey (MICS)	2010	Ministry of Health (Mali), National Institute of Statistics (INSTAT) (Mali), United Nations Children's Fund (UNICEF). Mali Multiple Indicator Cluster Survey 2009–2010. New York, United States of America: United Nations Children's Fund (UNICEF), 2017.	270627	Not extracted
Mali	Mali National Anthropometric Nutrition Survey and Mortality Retrospective	2016	National Directorate of Health (Mali), National Institute of Statistics (INSTAT) (Mali). Mali National Anthropometric Nutrition Survey and Mortality Retrospective June–August 2016.	297069	Not extracted
Mexico	Mexico National Survey of Health and Nutrition (ENSANUT)	2006	National Institute of Public Health (Mexico). Mexico National Survey of Health and Nutrition 2005–2006. Cuernavaca, Mexico: National Institute of Public Health (Mexico).	8618	Not extracted
Mongolia	Multiple Indicator Cluster Survey (MICS)	2016	National Statistical Office of Mongolia, United Nations Children's Fund (UNICEF). Mongolia — Nalaikh District Multiple Indicator Cluster Survey 2016. New York, United States of America: United Nations Children's Fund (UNICEF), 2018.	336042	Not nationally representative. Only sampled Nalaikh district in the municipality of Ulaanbaatar.

Mongolia	Mongolia National Nutrition Survey	2016	Ministry of Health (Mongolia), National Center for Public Health (Mongolia), United Nations Children's Fund (UNICEF). Mongolia National Nutrition Survey 2016.	340363	Not extracted
Nepal	NA	2009	Renzaho AMN. Child Grant Programme and the Health and Nutritional Well-being of Under-Five Children in the Karnali Zone of Nepal: Assessing the Impact of Integrated Social Protection Services and Trend Analysis in Five Districts-Re-analysis of secondary data. New York, United States: United Nations Children's Fund (UNICEF), 2017.	330625	Not extracted
Nepal	NA	2013	Renzaho AMN. Child Grant Programme and the Health and Nutritional Well-being of Under-Five Children in the Karnali Zone of Nepal: Assessing the Impact of Integrated Social Protection Services and Trend Analysis in Five Districts-Re-analysis of secondary data. New York, United States: United Nations Children's Fund (UNICEF), 2017.	330625	Not extracted
Nepal	NA	2015	Renzaho AMN. Child Grant Programme and the Health and Nutritional Well-being of Under-Five Children in the Karnali Zone of Nepal: Assessing the Impact of Integrated Social Protection Services and Trend Analysis in Five Districts-Re-analysis of secondary data. New York, United States: United Nations Children's Fund (UNICEF), 2017.	330625	Not extracted
Niger	Niger Nutrition and Child Survival Survey	2009	Ministry of Public Health (Niger), National Institute of Statistics (Niger). Niger Nutrition and Child Survival Survey 2009.	160053	Not extracted
Pakistan	DHS Standard Demographic and Health Survey	2018	ICF International, Ministry of National Health Services, Regulations & Coordination (Pakistan), National Institute of Population Studies (Pakistan). Pakistan Demographic and Health Survey 2017–	286783	Released after GBD 2019 data addition deadline

	(DHS), DHS Program Surveys		2018. Fairfax, United States of America: ICF International, 2018.		
Pakistan	Multiple Indicator Cluster Survey (MICS)	2017	Pakistan Bureau of Statistics, Planning and Development Department, Government of Gilgit-Baltistan (Pakistan), United Nations Children's Fund (UNICEF). Pakistan — Gilgit-Baltistan Multiple Indicator Cluster Survey 2016–2017. New York, United States of America: United Nations Children's Fund (UNICEF), 2020.	308316	Not nationally representative. Only sampled Gilgit-Baltistan in Pakistan.
Palestine	Pan Arab Project for Family Health (PAPFAM)	2006	League of Arab States, Palestinian Central Bureau of Statistics, United Nations Children's Fund (UNICEF). Palestine Family Health Survey 2006–2007.	9999	Not extracted
Rwanda	NA	2006	Concern Worldwide. Rwanda — Gisagara Knowledge, Practices, and Coverage of Services Survey 2006.	24148	Not extracted
Somalia	Somalia District Nutrition Survey	2003	International Federation of Red Cross and Red Crescent Societies, Muslim Aid, United Nations Children's Fund (UNICEF). Somalia — Jubbada Hoose Nutrition Survey in Kismayo District 2003.	142166	Not extracted
Thailand	Multiple Indicator Cluster Survey (MICS)	2016	National Health Security Office (Thailand), National Statistical Office (Thailand), United Nations Children's Fund (UNICEF). Thailand Multiple Indicator Cluster Survey 2015–2016. New York, United States of America: United Nations Children's Fund (UNICEF), 2018.	296646	Not extracted
Timor-Leste	NA	2013	Ministry of Health (Timor-Leste). Timor-Leste Food and Nutrition Survey 2013.	286211	Not extracted
Yemen	NA	2011	Ministry of Public Health and Population (Yemen), United Nations Children's Fund (UNICEF). Yemen — Al Hudaydah Nutrition Survey Among Under 5 Children 2011.	291261	Not extracted

Yemen	Yemen Nutritional Status and Mortality Survey	2016	Ministry of Public Health and Population (Yemen), United Nations Children's Fund (UNICEF). Yemen — Ad Dali Nutritional Status and Mortality Survey 2016.	292315	Not extracted
Yemen	Yemen Nutritional Status and Mortality Survey	2017	Ministry of Public Health and Population (Yemen), United Nations Children's Fund (UNICEF). Yemen — Shabwah Nutritional Status and Mortality Survey 2017.	292489	Not extracted
Yemen	Yemen Nutritional Status and Mortality Survey	2016	Ministry of Public Health and Population (Yemen), United Nations Children's Fund (UNICEF). Yemen — San,Äòa Nutrition Survey 2016.	292491	Not extracted
Yemen	Yemen Nutritional Status and Mortality Survey	2018	Action Against Hunger (ACF), Ministry of Public Health and Population (Yemen), United Nations Children's Fund (UNICEF). Yemen — Hajjah Nutrition and Retrospective Mortality Survey 2018.	373856	Not extracted
Yemen	Yemen Nutritional Status and Mortality Survey	2018	Abyan Governmental Health Office, Action Against Hunger (ACF), Ministry of Public Health and Population (Yemen). Yemen — Abyan Nutrition and Retrospective Mortality Survey 2018.	373862	Not extracted
Yemen	Yemen Nutritional Status and Mortality Survey	2017	Ministry of Public Health and Population (Yemen), United Nations Children's Fund (UNICEF). Yemen — Ibb Nutrition and Mortality Survey 2017.	373869	Not extracted
Zambia	Zambia Living Conditions Monitoring Survey	2010	Central Statistical Office (Zambia). Zambia Living Conditions Monitoring Survey 2010.	58660	Not extracted
Zambia	NA	2014	European Union (EU), Government of Zambia, Liverpool School of Tropical Medicine, United Nations Children's Fund (UNICEF). Zambia Lot Assurance Quality Sampling Survey 2014.	281731	Not extracted

♦NID = Data source unique identifier in the Global Health Data Exchange (GHDx) (http://ghdx.healthdata.org/). Additional information about each data source is available via the GHDx, including information about the data provider and links to where the data can be accessed or requested (where available). NIDs can be entered in the search bar to retrieve the record for a particular source.

Supplementary Table 3. Data excluded from geostatistical model but included in GBD estimates

Country	Series	Year(s)	Citation	NID♦	Rationale for exclusion
Afghanistan	Multiple Indicator Cluster Survey (MICS)	2003	Central Statistics Organization (Afghanistan), United Nations Children's Fund (UNICEF). Afghanistan Multiple Indicator Cluster Survey 2003.	561	The data is not subnationally representative.
Afghanistan	Afghanistan Health Survey (AHS)	2006	Indian Institute of Health Management Research (IIHMR), Johns Hopkins University, Ministry of Public Health (Afghanistan). Afghanistan Health Survey 2006.	18468	The data is not subnationally representative.
Algeria	Multiple Indicator Cluster Survey (MICS)	2000	Ministry of Health and Population (Algeria), National Institute of Public Health (Algeria), National Office of Statistics (Algeria), United Nations Children's Fund (UNICEF). Algeria Multiple Indicator Cluster Survey 2000.	26449	There is no sample size for each of the subnational location.
Algeria	Pan Arab Project for Family Health (PAPFAM)	2002	National Office of Statistics (Algeria), Ministry of Health, Population and Hospital Reform (Algeria), League of Arab States. Algeria Family Health Survey 2002–2003.	627	Missing age in months.
Colombia	DHS Standard Demographic and Health Survey (DHS), DHS Program Surveys	2004	Macro International, Inc, Profamilia (Colombia). Colombia Demographic and Health Survey 2004–2005. Fairfax, United States of America: ICF International, 2005.	19324	Estimates considered implausible (zero or extreme values).
Colombia	DHS Standard Demographic and Health Survey (DHS), DHS Program Surveys	2009	ICF Macro, Profamilia (Colombia). Colombia Demographic and Health Survey 2009–2010. Fairfax, United States of America: ICF International, 2011.	21281	Estimates considered implausible (zero or extreme values).
Dominican Republic	DHS Standard Demographic and Health Survey (DHS), DHS Program Surveys	2002	Center for Social and Demographic Studies (Dominican Republic) (CESDEM), Macro International, Inc. Dominican Republic Demographic and Health Survey 2002.	19444	Estimates considered implausible (zero or extreme values).

			Fairfax, United States of America: ICF International.		
El Salvador	Reproductive Health Survey (RHS)	2002	Asociación Demográfica Salvadoreña (ADS), Division of Reproductive Health — Centers for Disease Control and Prevention (CDC). (2004) El Salvador Reproductive Health Survey 2002–2003. San Salvador, El Salvador: ADS.	27599	Missing relevant breastfeeding indicators.
El Salvador	Reproductive Health Survey (RHS)	2008	Asociación Demográfica Salvadoreña (ADS), Division of Reproductive Health — Centers for Disease Control and Prevention (CDC). (2009) El Salvador Reproductive Health Survey 2008. San Salvador, El Salvador: ADS.	27606	Missing relevant breastfeeding indicators.
Equatorial Guinea	DHS Standard Demographic and Health Survey (DHS), DHS Program Surveys	2011	ICF International, Ministry of Health and Social Welfare (Equatorial Guinea), Ministry of Planning, Economic Development and Public Investment (Equatorial Guinea). Equatorial Guinea Demographic and Health Survey 2011. Fairfax, United States of America: ICF International, 2012.	76884	The data is not subnationally representative.
Guinea-Bissau	Multiple Indicator Cluster Survey (MICS)	2000	Secretary State of Planning, National Institute of Statistics and Census (INEC), United Nations Children's Fund (UNICEF). Guinea-Bissau Multiple Indicator Cluster Survey 2000. New York, United States: United Nations Children's Fund (UNICEF).	4808	Estimates considered implausible (zero or extreme values).
Guinea-Bissau	Multiple Indicator Cluster Survey (MICS)	2010	Centers for Disease Control and Prevention (CDC), National Statistics Institute (Guinea-Bissau), United Nations Children's Fund (UNICEF). Guinea-Bissau Multiple Indicator Cluster Survey 2010. New York, United	27215	Missing relevant breastfeeding indicators.

			States: United Nations Children's Fund (UNICEF), 2018.		
Honduras	Reproductive Health Survey (RHS)	2001	Honduras Family Planning Association (ASHONPLAFA), Ministry of Health (Honduras), and Division of Reproductive Health — Centers for Disease Control and Prevention (CDC). Honduras Reproductive Health Survey 2001. Tegucigalpa, Honduras: Honduras Family Planning Association (ASHONPLAFA).	27551	Missing relevant breastfeeding indicators.
India	Multiple Indicator Cluster Survey (MICS)	2000	United Nations Statistical Division, World Health Organization (WHO), United Nations Educational, Scientific, and Cultural Organization (UNESCO), United Nations Population Fund (UNFPA), World Bank (WB), London School of Hygiene and Tropical Medicine, United Nations Children's Fund (UNICEF). India Multiple Indicator Cluster Survey 2000. New York, United States: United Nations Children's Fund (UNICEF).	5127	There is no sample size for each of the subnational location.
Indonesia	DHS Standard Demographic and Health Survey (DHS), DHS Program Surveys	2002	Macro International, Inc, Ministry of Health (Indonesia), National Family Planning Coordinating Board (Indonesia), Statistics Indonesia. Indonesia Demographic and Health Survey 2002–2003. Fairfax, United States of America: ICF International.	20011	Estimates considered implausible (zero or extreme values).
Jordan	DHS Standard Demographic and Health Survey (DHS), DHS Program Surveys	2002	Department of Statistics (Jordan), Macro International, Inc. Jordan Demographic and Health Survey 2002. Fairfax, United States of America: ICF International.	20073	Estimates considered implausible (zero or extreme values).

Kenya	Multiple Indicator Cluster Survey (MICS)	2007	Kenya National Bureau of Statistics, United Nations Children's Fund (UNICEF). Kenya — North Eastern Province Multiple Indicator Cluster Survey 2007. Nairobi, Kenya: Kenya National Bureau of Statistics.	155335	Estimates considered implausible (zero or extreme values).
Laos	Laos Reproductive Health Survey	2005	National Statistical Center (Laos). Laos Reproductive Health Survey 2005.	43045	The survey does not specify a 24-hour recall period.
Lesotho	Multiple Indicator Cluster Survey (MICS)	2000	Bureau of Statistics (Lesotho), United Nations Children's Fund (UNICEF). Lesotho Multiple Indicator Cluster Survey 2000. New York, United States of America: United Nations Children's Fund (UNICEF).	7721	Missing survey weights.
Morocco	Multiple Indicator Cluster Survey (MICS)	2006	Ministry of Health (Morocco), United Nations Children's Fund (UNICEF). Morocco Multiple Indicator Cluster Survey 2006.	8852	The data is not subnationally representative.
Nepal	DHS Standard Demographic and Health Survey (DHS), DHS Program Surveys	2001	Macro International, Inc, Ministry of Health and Population (Nepal), New ERA. Nepal Demographic and Health Survey 2001. Fairfax, United States of America: ICF International.	20450	Estimates considered implausible (zero or extreme values).
Nicaragua	Reproductive Health Survey (RHS)	2006	Division of Reproductive Health, Centers for Disease Control and Prevention (CDC), National Institute for Development Information (Nicaragua). Nicaragua Reproductive Health Survey 2006–2007. Managua, Nicaragua: National Institute for Development Information (Nicaragua).	9270	Missing relevant breastfeeding indicators.
Nigeria	Nigeria General Household Survey	2008	Central Bank of Nigeria, National Bureau of Statistics (Nigeria), Nigerian Communications Commission (NCC). Nigeria General Household Survey 2008.	24915	Estimates considered implausible (zero or extreme values).

Pakistan	Multiple Indicator Cluster Survey (MICS)	2014	Bureau of Statistics Punjab (Pakistan), United Nations Children's Fund (UNICEF). Pakistan — Punjab Multiple Indicator Cluster Survey 2014. New York, United States of America: United Nations Children's Fund (UNICEF), 2015.	236266	Estimates considered implausible (zero or extreme values).
Paraguay	Reproductive Health Survey (RHS)	2004	Division of Reproductive Health — Centers for Disease Control and Prevention (CDC). (2005): Paraguay Reproductive Health Survey 2004. Asunción, Paraguay, Paraguayan Center for Population Studies (CEPEP).	10370	Missing relevant breastfeeding indicators.
Paraguay	Reproductive Health Survey (RHS)	2008	Paraguay Center for Population Studies (CEPEP). Paraguay Reproductive Health Survey 2008. Asunción, Paraguay: Paraguayan Center for Population Studies (CEPEP).	27525	Missing relevant breastfeeding indicators.
The Philippines	DHS Standard Demographic and Health Survey (DHS), DHS Program Surveys	2003	Macro International, Inc, National Statistics Office (Philippines). Philippines Demographic and Health Survey 2003. Fairfax, United States of America: ICF International.	20699	Estimates considered implausible (zero or extreme values).
Republic of the Congo	DHS Standard Demographic and Health Survey (DHS), DHS Program Surveys	2005	Macro International, Inc, National Center for Statistics and Economic Studies (Congo, Rep.). Congo Demographic and Health Survey 2005. Fairfax, United States of America: ICF International.	19391	Missing relevant breastfeeding indicators.
Sudan	Multiple Indicator Cluster Survey (MICS)	2010	Federal Ministry of Health and Central Bureau of Statistics, Sudan Household and Health Survey — 2, 2012, National report. Khartoum, Republic of Sudan: Federal Ministry of Health and Central Bureau of Statistics.	153563	Missing age in months.

Syria	Pan Arab Project for Family Health (PAPFAM)	2001	Central Bureau of Statistics (Syria), League of Arab States. Syria Family Health Survey 2001.	12379	Missing relevant breastfeeding indicators.
Syria	Multiple Indicator Cluster Survey (MICS)	2006	General Administration for Palestine Arab Refugees (GAPAR), Palestinian Central Bureau of Statistics, Pan Arab Project for Family Health (PAPFAM), United Nations Children's Fund (UNICEF). Palestinians in Syria Multiple Indicator Cluster Survey 2006.	10023	The data is not representative of Syrian population.
Tunisia	Multiple Indicator Cluster Survey (MICS)	2006	Ministry of Public Health (Tunisia), National Office for Family and Population, Ministry of Public Health (Tunisia), United Nations Children's Fund (UNICEF). Tunisia Multiple Indicator Cluster Survey 2006.	12985	Lacking geographic information.
Turkmenistan	DHS Standard Demographic and Health Survey (DHS), DHS Program Surveys	2000	Gurbansoltan Eje Clinical Research Center for Maternal and Child Health (GECRCMCH), Macro International, Inc, Ministry of Health and Medical Industry (Turkmenistan). Turkmenistan Demographic and Health Survey 2000.	20956	The report is in Turkmen and has not been translated for this round of modeling.
Uganda	Child Verbal Autopsy Study (CVAS)	2007	MEASURE Evaluation Project, Carolina Population Center, University of North Carolina, Macro International, Inc, Ministry of Health (Uganda), Uganda Bureau of Statistics. Uganda Child Verbal Autopsy Study 2007. Calverton, United States: Macro International, Inc.	23289	Lacking geographic information.
Vietnam	Multiple Indicator Cluster Survey (MICS)	2010	General Statistics Office (Vietnam), United Nations Children's Fund (UNICEF). Vietnam Multiple Indicator Cluster Survey 2010–2011. New York, United States of America: United Nations Children's Fund (UNICEF).	57999	The data can not be geomatched to the correct subnational locations.

Zambia	Zambia Living Conditions Monitoring Survey	2004	Central Statistical Office (Zambia). Zambia Living Conditions Monitoring Survey 2004–2005. Lusaka, Zambia: Central Statistical Office (Zambia).	14063	Estimates considered implausible (zero or extreme values).

♦NID = Data source unique identifier in the Global Health Data Exchange (GHDx) (http://ghdx.healthdata.org/). Additional information about each data source is available via the GHDx, including information about the data provider and links to where the data can be accessed or requested (where available). NIDs can be entered in the search bar to retrieve the record for a particular source.

2.1. Data excluded from model

To identify potential survey biases, we reviewed national-level survey estimates for each country and compared with national-level estimates from DHS, GBD, and the geospatial model. In cases where a survey's estimates appeared implausible in comparison with other existing survey-based data sources, we inspected differences in definitions, data collection, or other methodological explanations. Supplementary Table 1 provides a list of surveys that were excluded from both geostatistical model and GBD 2019 estimates[1]. Supplementary Table 2 provides a list of surveys that were included in the geostatistical model but excluded from GBD estimates (in cases where surveys were non-nationally representative but could provide spatial information for the geostatistical model). Additionally, a number of surveys were included in GBD estimates but excluded from the geostatistical model (Supplementary Table 3). For each case, we specified reasons for exclusion in the tables.

2.2. Data processing

The technical descriptions of data processing and methods for resampling are consistent with those previously used in the geospatial modelling of EBF across Africa[2].

The scope of our data extraction included Demographic and Health Surveys (DHS), Multiple Indicator Cluster Surveys (MICS) and country-specific surveys collected from 1998 to 2018 in LMICs. As a first step, we completed the following data extraction process:

- Searched the Global Health Data Exchange (GHDx: http://ghdx.healthdata.org/) for all surveys in LMICs tagged as containing exclusive breastfeeding indicators of interest;
- Designed and tested a codebook, or survey data extraction framework, for breastfeeding variables present in the household surveys;
- Extracted and geo-matched (either to GPS data or administrative units) all surveys available for LMICs;
- Refreshed our query of the GHDx for surveys performed in LMICs.

Some surveys directly ask the question: "did you exclusively breastfeed?". However, in our preliminary analysis we found that responses to this question were widely inconsistent across surveys. This is likely because the respondent may not understand the meaning of "exclusively breastfeed" or the question may be misinterpreted with translation. Instead, we used the following survey response information to determine exclusive breastfeeding for children under six months:

- Whether the child is still being breastfed;
- Food and liquid items given to a child in the past 24 hours.

Surveys were excluded from this analysis if they lacked subnational geographic identifiers, were not available at the individual level, or did not contain sufficient information to generate the exclusive breastfeeding indicator. Specifically, our inclusion criteria for survey microdata with complete records were the following:

- "Survey responses must be available at the individual level;
- Survey must contain subnational geographic identifiers, which could include either subnational areal units (typically administrative units) or GPS coordinates. Data

referenced to subnational areal units must also contain survey weights for each observation;

- Survey must have been conducted between 1998 and 2018;
- Survey must contain questions about the age of the child, whether the child is still being breastfed, and whether the child has consumed other food or liquid items. Typically, consumption during the past 24 hours is recorded. In 22 out of 349 household surveys, the question about food or liquid items did not specify a particular recall period. After performing sensitivity analysis, we decided to keep those surveys in our model.

In cases where survey microdata were not available, we were instead able to include estimates of EBF prevalence from survey reports. Survey reports were excluded from this analysis if they lacked subnational geographic identifiers, did not include a sample size or confidence interval, or the estimates reported appeared implausible. Specifically, our inclusion criteria for these surveys were the following:

- Survey must contain subnational identifiers, which could include subnational areal units (typically administrative units);
- Survey must have been conducted between 1998 and 2018;
- Survey must contain the prevalence of exclusive breastfeeding with a sample size or the lower and upper bounds for the 95% confidence interval.

After data extraction, the following steps were performed prior to using these data in our models:

- Aggregated the individual-level responses from survey microdata to calculate EBF prevalence and the effective sample size at the finest possible spatial resolution available, incorporating individual-level sample weights and using the Kish approximation[3] for the effective sample size.
  - For surveys where a latitude and longitude pair representing the location of each survey cluster were available ("point data"), data were aggregated to these specific coordinates.
  - For surveys where cluster-specific latitude and longitude pairs were not available, the smallest geographic area was used instead ("polygon data"). Typically, these polygons correspond to administrative units.
- Resampled data matched to polygons to generate pseudo-point data based on the underlying population distribution within the polygon.

The methods for the resampling are consistent with those previously used in geospatial modelling of under-5 mortality[4]. Specifically, for each polygon-level observation, we randomly sampled 10,000 locations among 5 × 5-km grid cells in the given polygon with probability proportional to grid-cell population. Grid cells were defined to be contained within the polygon if their centroid fell within the geographic boundary. We performed k-means clustering (with k set to 1 per 40 grid cells) on the sampled points to generate a reduced set of locations to be used in modelling based on the k-means cluster centroids. Weights were assigned to each pseudo-point proportional to the number of sampled points contained in each of the k-means clusters (i.e., the number of sampled points divided by 10,000). Each pseudo-point generated by this process was assigned the EBF prevalence and sample size observed for the polygon as a whole, and the weights associated with each pseudo-point were applied during all stages of model fitting.

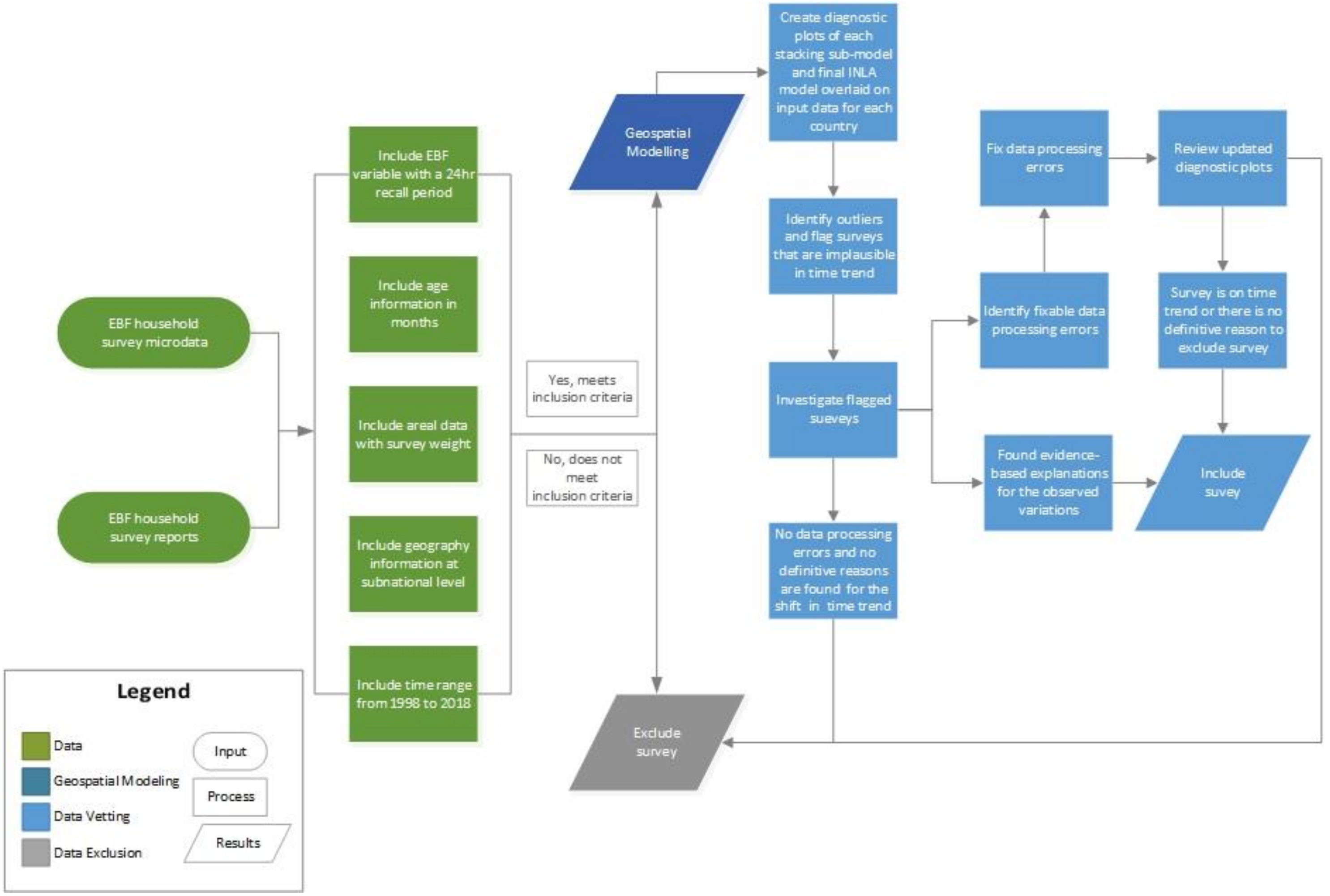

Supplementary Figure 6. Flowchart for data extraction (a) and data cleaning (b) processes

(a) Data extraction refers to the manual extraction process where a survey will be excluded if it does not contain geography variables that we can match to, specific questions for exclusive breastfeeding, and field for age information; **(b)** Data cleaning refers to the process where the extractions are collapsed in our post-processing code and we exclude a survey if it does not contain survey weights, sufficient geographic information, age

information that can be converted to months, observations in our time study period, observations for children 0–5 months, or valid responses for specific breastfeeding questions. We also exclude surveys that contain outlier observations at this stage.

2.3. Geographic inclusion

We included 94 low- and middle- income countries (LMIC) in this analysis. LMIC status was determined by the Global Burden of Disease's (GBD) Socio-demographic Index (SDI), which is a composite variable of poverty, education, and fertility and which indicates a country's level of development. The countries of Ecuador, Venezuela, Malaysia, Sri Lanka, Iran, Djibouti, Libya, Cape Verde, Dominica, Grenada, and Seychelles were excluded despite Low, Low-Middle, or Middle status due to insufficient data. Supplementary Figure 7 represents a map of the countries included in this study, and Supplementary Table 4 provides the list of 94 countries along with region name, SDI and ISO3 code for each country.

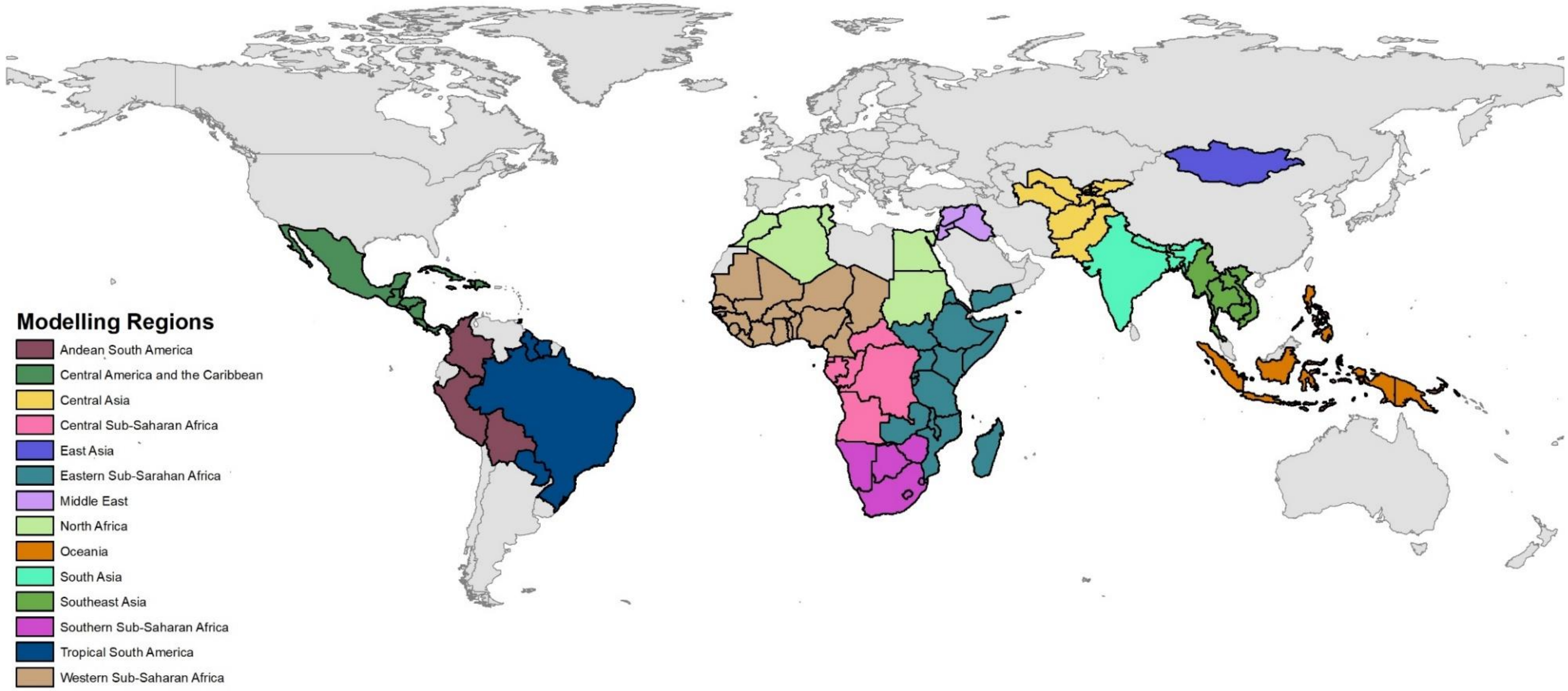

Supplementary Figure 7. Countries included in this analysis and modeling regions

Supplementary Table 4. Countries included in the analysis (94) grouped by modelling regions.

Region name	Country name	ISO3 Code	Socio-demographic Index
Andean South America	Bolivia	BOL	Low-Middle SDI
	Colombia	COL	Middle SDI
	Peru	PER	Middle SDI
	Trinidad and Tobago	TTO	Middle SDI
Central America and the Caribbean	Belize	BLZ	Low-Middle SDI
	Costa Rica	CRI	Middle SDI
	Cuba	CUB	Middle SDI
	Dominican Republic	DOM	Low-Middle SDI
	El Salvador	SLV	Low-Middle SDI
	Guatemala	GTM	Low-Middle SDI
	Haiti	HTI	Low SDI
	Honduras	HND	Low-Middle SDI
	Jamaica	JAM	Middle SDI
	Mexico	MEX	Middle SDI
	Nicaragua	NIC	Low-Middle SDI
	Panama	PAN	Middle SDI
Central Asia	Afghanistan	AFG	Low SDI
	Kyrgyzstan	KGZ	Low-Middle SDI
	Pakistan	PAK	Low-Middle SDI
	Tajikistan	TJK	Low-Middle SDI
	Turkmenistan	TKM	Middle SDI
	Uzbekistan	UZB	Middle SDI
Central sub-Saharan Africa	Angola	AGO	Low-Middle SDI
	Central African Republic	CAF	Low SDI
	Democratic Republic of the Congo	COD	Low SDI
	Equatorial Guinea	GNQ	Middle SDI
	Gabon	GAB	Middle SDI
	Republic of the Congo	COG	Low-Middle SDI
East Asia	Mongolia	MNG	Middle SDI
Eastern sub-Saharan Africa	Burundi	BDI	Low SDI
	Comoros	COM	Low SDI
	Eritrea	ERI	Low SDI
	Ethiopia	ETH	Low SDI
	Kenya	KEN	Low-Middle SDI
	Madagascar	MDG	Low SDI
	Malawi	MWI	Low SDI
	Mozambique	MOZ	Low SDI
	Rwanda	RWA	Low SDI
	Somalia	SOM	Low SDI
	South Sudan	SSD	Low SDI

	Tanzania	TZA	Low SDI
	Uganda	UGA	Low SDI
	Yemen	YEM	Low SDI
	Zambia	ZMB	Low-Middle SDI
Middle East	Iraq	IRQ	Low-Middle SDI
	Jordan	JOR	Middle SDI
	Palestine	PSE	Low-Middle SDI
	Syria	SYR	Middle SDI
North Africa	Algeria	DZA	Middle SDI
	Egypt	EGY	Low-Middle SDI
	Morocco	MAR	Low-Middle SDI
	Sudan	SDN	Low-Middle SDI
	Tunisia	TUN	Middle SDI
Oceania	Indonesia	IDN	Middle SDI
	Papua New Guinea	PNG	Low SDI
	The Philippines	PHL	Middle SDI
	Timor-Leste	TLS	Low-Middle SDI
South Asia	Bangladesh	BGD	Low SDI
	Bhutan	BTN	Low-Middle SDI
	India	IND	Low-Middle SDI
	Nepal	NPL	Low SDI
Southeast Asia	Cambodia	KHM	Low-Middle SDI
	Laos	LAO	Low-Middle SDI
	Myanmar	MMR	Low-Middle SDI
	Thailand	THA	Middle SDI
	Vietnam	VNM	Middle SDI
Southern sub-Saharan Africa	Botswana	BWA	Middle SDI
	Eswatini	SWZ	Low-Middle SDI
	Lesotho	LSO	Low-Middle SDI
	Namibia	NAM	Middle SDI
	South Africa	ZAF	Middle SDI
	Zimbabwe	ZWE	Low-Middle SDI
Tropical South America	Brazil	BRA	Middle SDI
	Guyana	GUY	Low-Middle SDI
	Paraguay	PRY	Middle SDI
	Suriname	SUR	Middle SDI
Western sub-Saharan Africa	Benin	BEN	Low SDI
	Burkina Faso	BFA	Low SDI
	Cameroon	CMR	Low-Middle SDI
	Chad	TCD	Low SDI
	Côte d'Ivoire	CIV	Low SDI
	Gambia	GMB	Low SDI
	Ghana	GHA	Low-Middle SDI
	Guinea	GIN	Low SDI
	Guinea-Bissau	GNB	Low SDI

	Liberia	LBR	Low SDI
	Mali	MLI	Low SDI
	Mauritania	MRT	Low-Middle SDI
	Niger	NER	Low SDI
	Nigeria	NGA	Low-Middle SDI
	São Tomé and Príncipe	STP	Low-Middle SDI
	Senegal	SEN	Low SDI
	Sierra Leone	SLE	Low SDI
	Togo	TGO	Low SDI

3.0. Covariates

The descriptions of covariates for the underlying geostatistical model are consistent with those previously used in the geospatial modelling of EBF across Africa[2].

In these analyses, we included the following socioeconomic, environmental, and health-related covariates to improve the predictions of exclusive breastfeeding: urban proportion of the location [TV], night-time lights [TV], travel time to the nearest settlement >50,000 inhabitants, population [TV], Human Development Index (HDI) [TV], educational attainment in women of reproductive age (15–49 years old) [TV], number of people whose daily vitamin A needs could be met, number of children under 5 per woman of childbearing age [TV], Healthcare Access and Quality Index (HAQI) [TV], proportion of pregnant women who received four or more antenatal care visits [TV], and human immunodeficiency virus (HIV) prevalence [TV] ([TV]=time-varying covariates). Of these, the covariates for the Healthcare Access and Quality Index[5] and the proportion of pregnant women who received four or more antenatal care visits[6] were indexed at the national level, while all others were indexed at the subnational level.

These covariates were selected because they are factors or proxies for factors that previous literature has identified to be associated (not necessarily causally) with exclusive breastfeeding prevalence. The first four covariates were included as measures or proxies for connectedness and urbanicity as EBF is typically found to be different in urban areas compared to rural locations[7–11]. Human Development Index (HDI; a composite indicator of key aspects of development: namely, education, economy, and health) was chosen based on prior studies relating country development to EBF[12]. Educational attainment in women of reproductive age (15–49 years old) was included because previous studies highlight education as a maternal factor influencing the decision to initiate and continue EBF[13,14]. Number of people whose daily vitamin A needs could be met was chosen as a proxy of maternal nutrition while breastfeeding[15,16]. Number of children under 5 per woman of childbearing age was selected as a previous study suggest that EBF rates are higher among women with more than 4 children[17]. Healthcare Access and Quality Index was chosen because maternal care practices that promote breastfeeding are influenced by access to high-quality health care[18,19]. Proportion of pregnant women who received four or more antenatal care visits due to positive association between EBF and antenatal care[20]. Human immunodeficiency virus (HIV) was included given the known risks of mother-to-child transmission of HIV and consequent potential avoidance of breastfeeding in hyperendemic settings over the study period[21–24]. These covariates underwent spatial and temporal processing in preparation for their inclusion in analysis.

Spatial processing involved resampling the input covariate raster to align the spatial resolution of the covariate to the 5 × 5-km resolution used in modelling. For covariates that were originally at a finer resolution, we resampled the raster by taking the neighbourhood average (travel time to the nearest settlement of more than 50,000 inhabitants, night-time lights) or using the nearest neighbour (urbanicity) or sum (total population) of the finer covariate raster to produce one at a 5 × 5-km resolution. Educational attainment in women of reproductive age and HIV covariates were natively at a 5 × 5-km resolution and thus did not require additional spatial processing. For covariates that were originally at lower resolution (HDI and nutritional yield for vitamin A), we resampled the raster using bilinear interpolation, with the effect of smoothing some of the hard grid-cell boundaries in the raw data to make for a 5 × 5-km resolution raster.

Temporal processing was required in instances where the original temporal resolution of the covariate was anything other than annual. To resolve from a coarser time period to an annual time period, we filled the intervening years with the value from the nearest neighbouring year (urbanicity) or utilizing an exponential growth rate model (total population). Night-time lights, educational attainment, and HIV prevalence were available at a one-year temporal resolution and did not require interpolation. As travel time to the nearest settlement of more than 50,000 inhabitants and nutritional yield for vitamin A covariates were available only for a single representative year (2015 and 2005, respectively) these covariates were set to be unchanged over time. After interpolation, night-time lights, human development index and urbanicity were still missing the most recent years of the 2000 to 2018 analysis period, and in these instances we filled out the end of the time-series carrying forward the most recent year without modification.

We filtered these covariates for multi-collinearity within each modeling region (see Supplementary Figure 7) using variance inflation factor[25] (VIF) analysis based on a threshold of VIF <3. We list detailed information on temporal resolution and source(s) for each included covariate (11) in Supplementary Table 5. In addition, calendar year was utilized as a covariate in our model. Supplementary Figure 8 provides maps of spatial covariates. Supplementary Table 6 lists the final covariates selected for each region based on VIF analysis.

Supplementary Table 5. Sources for covariates used in mapping.

Covariate	Temporal resolution	Source	Reference
Educational attainment in women of reproductive age (15–49 years old)[TV]	Annual	Institute for Health Metrics and Evaluation (IHME), University of Washington	Graetz, N. *et al*. Local variation in educational attainment in low- and middle-income countries, 2000–2017. *Nature* **577,** 235–238 (2020).
Night-time lights[TV]	Annual	NOAA DMSP	National Oceanic and Atmospheric Administration (NOAA) (United States), United States Air Force (USAF). DMSP-OLS Nighttime Lights Time Series, V4. United States of America: National Oceanic and Atmospheric Administration (NOAA) (United States).
Population[TV]	Annual	WorldPop	Lloyd, C. T., Sorichetta, A. & Tatem, A. J. High resolution global gridded data for use in population studies. *Sci. Data* **4**, (2017). World Pop. Get data. Available at: http://www.worldpop.org.uk/data/get_data/. (Accessed: 25th July 2017)
Travel time to nearest settlement >50,000 inhabitants	Static	Malaria Atlas Project, Big Data Institute, Nuffield Department of Medicine, University of Oxford	Weiss, D. J. *et al*. A global map of travel time to cities to assess inequalities in accessibility in 2015. *Nature* **533**, 333–336 (2018).
Urban proportion of the location (landcover)[TV]	Annual	MODIS	Friedl, M. & Sulla-Menashe, D. MCD12Q1v006.MODIS/Terra+Aqua Land Cover Type Yearly L3 Global 500m SIN Grid https://doi.org/10.5067/MODIS/MCD12Q1.006 (NASA EOSDIS Land Processes DAAC, 2019).
Number of people whose daily vitamin A needs could be met (nutrient yield)	Static	Herrero et al (modelled)	Herrero, M. et al. Farming and the geography of nutrient production for human use: a transdisciplinary analysis. *Lancet Planet. Health* **1**, e33–e42 (2017).
Human Development Index (HDI) [TV]	Annual	Kummu et al (modelled)	Kummu, M. et al. Gridded global datasets for Gross Domestic Product and Human Development Index over 1990–2015. Scientific data, 5:180004 (2018)
Human Immunodeficiency Virus (HIV)[TV]	Annual	Institute for Health Metrics and Evaluation (IHME), University of Washington	Dwyer-Lindgren, L. et al. Mapping HIV prevalence in sub-Saharan Africa between 2000 and 2017. *Nature* **570**, 189–193 (2019).

Covariate	Temporal resolution	Source	Reference
Number of children under 5 per woman of childbearing age (fertility)[TV]	Annual	WorldPop (derived)	Lloyd, C. T., Sorichetta, A. & Tatem, A. J. High resolution global gridded data for use in population studies. *Sci. Data* **4**, (2017).
Healthcare Access and Quality Index (HAQI)[TV]	Annual	Institute for Health Metrics and Evaluation (IHME), University of Washington	Fullman, N. et al. Measuring performance on the Healthcare Access and Quality Index for 195 countries and territories and selected subnational locations: a systematic analysis from the Global Burden of Disease Study 2016. The Lancet 391, 2236–2271 (2018).
Proportion of pregnant women who received four or more antenatal care visits[TV]	Annual	Institute for Health Metrics and Evaluation (IHME), University of Washington	Lozano, R. et al. Measuring progress from 1990 to 2017 and projecting attainment to 2030 of the health-related Sustainable Development Goals for 195 countries and territories: a systematic analysis for the Global Burden of Disease Study 2017. The Lancet 392, 2091–2138 (2018).<br><br>*Note: SDI numbers were updated based on the GBD 2019 studies*

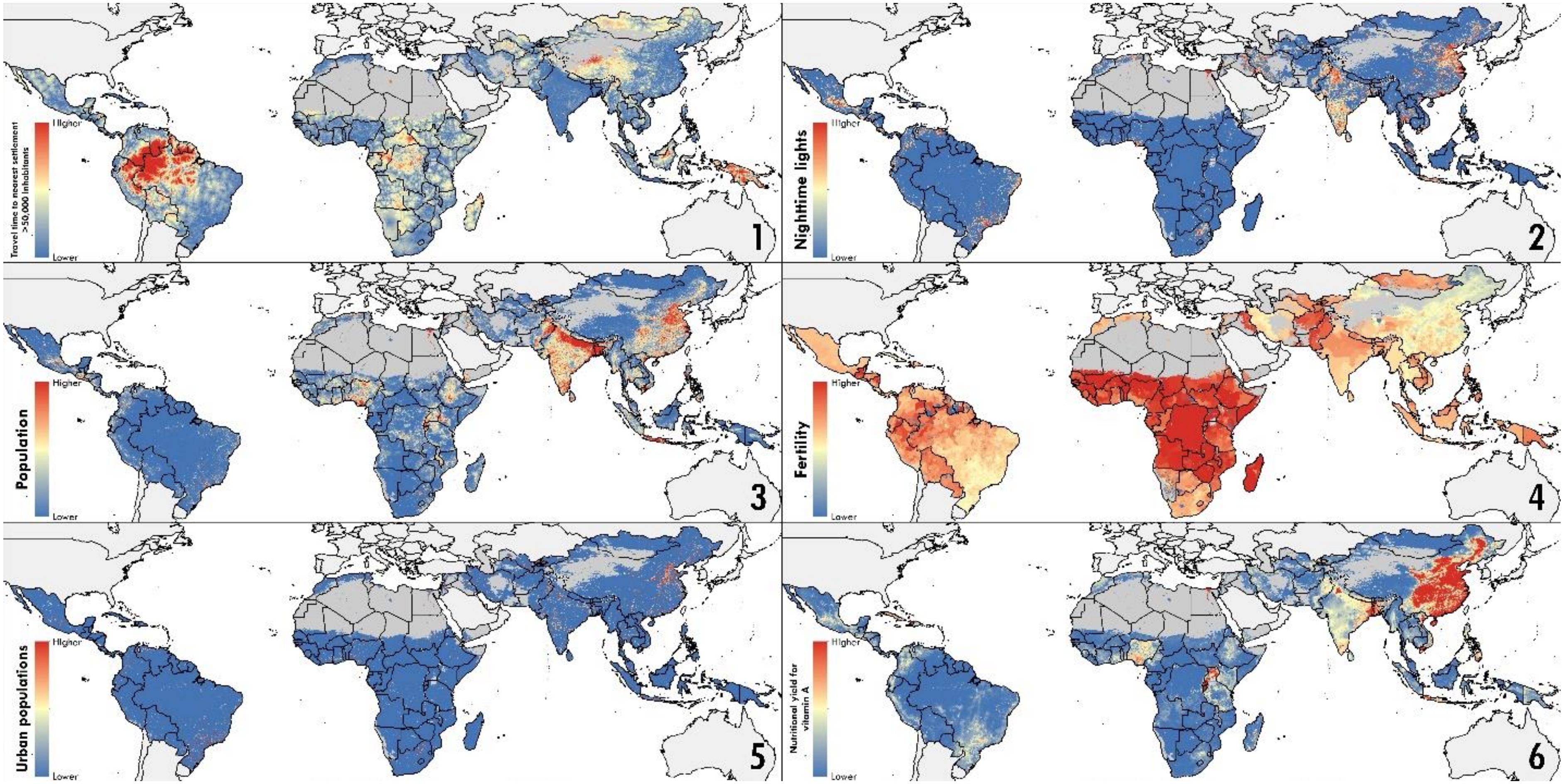
Travel time to nearest settlement >50,000 inhabitants
Higher
Lower
1
Nighttime lights
Higher
Lower
2
Population
Higher
Lower
3
Fertility
Higher
Lower
4
Urban populations
Higher
Lower
5
Nutritional yield for vitamin A
Higher
Lower
6

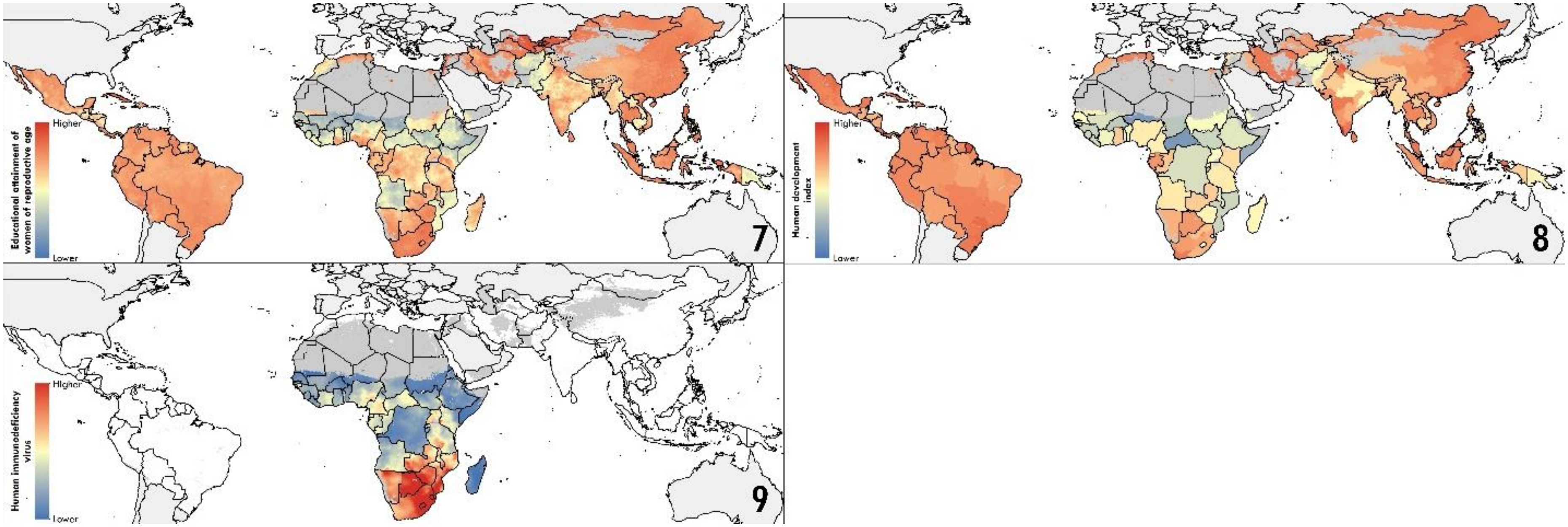

Supplementary Figure 8. Map of spatial covariates

Covariate raster layers of possible socioeconomic, environmental, and health-related covariates used as inputs for the stacking modelling process: (1) travel time to the nearest settlement >50,000 inhabitants, (2) nighttime lights^{TV}, (3) population^{TV}, (4) number of children under 5 per woman of childbearing age^{TV}, (5) urban proportion of the location^{TV}, (6) number of people whose daily vitamin A needs could be met, (7) educational attainment in women of reproductive age (15–49 years-old)^{TV}, (8) Human Development Index (HDI)^{TV}, and (9) human immunodeficiency virus (HIV) prevalence^{TV} (^{TV}=time-varying covariates). The two other covariates in the model were (10) Healthcare Access and Quality Index^{TV} and (11) the proportion of pregnant women who received four or more antenatal care visits^{TV}, but these were indexed at the national level and thus not shown in this figure. Time-varying covariates are presented for the most recent year. For the year of production of non-time-varying covariates, please refer to the individual covariate citation in Supplementary Table 5 for additional detail. Maps reflect administrative boundaries, land cover, lakes, and population; grey-colored grid cells had fewer than ten people per 1 × 1-km grid cell and were classified as "barren or sparsely vegetated", or were not included in this analysis[26–31].

Supplementary Table 6. Covariates used in ensemble covariate modelling via stacked generalization, stratified by modeling region

Region	Educational attainment^{TV}	Night-time lights^{TV}	Popula-tion^{TV}	Travel time	Urban proportion of the location^{TV}	Nutrient yield	HDI^{TV}	HIV^{TV}	Fertility ^{TV}	HAQI^{TV}	Antenatal care^{TV}
Andean South America	TRUE	FALSE	TRUE	TRUE	TRUE	TRUE	FALSE	FALSE	TRUE	TRUE	FALSE
Central America and the Caribbean	TRUE	TRUE	TRUE	TRUE	FALSE	TRUE	FALSE	FALSE	TRUE	FALSE	FALSE
Central Asia	TRUE	FALSE	TRUE	TRUE	TRUE	TRUE	FALSE	FALSE	TRUE	FALSE	FALSE
Central sub-Saharan Africa	TRUE	TRUE	TRUE	TRUE	TRUE	TRUE	FALSE	TRUE	TRUE	FALSE	TRUE
East Asia	TRUE	FALSE	TRUE	TRUE	TRUE	TRUE	FALSE	FALSE	TRUE	FALSE	FALSE
Eastern sub-Saharan Africa	TRUE	FALSE	TRUE	TRUE	TRUE	TRUE	FALSE	TRUE	TRUE	TRUE	TRUE
Middle East	TRUE	TRUE	TRUE	TRUE	FALSE	TRUE	FALSE	FALSE	TRUE	FALSE	FALSE
North Africa	TRUE	FALSE	TRUE	TRUE	TRUE	TRUE	FALSE	TRUE	TRUE	FALSE	FALSE
Oceania	TRUE	TRUE	TRUE	TRUE	FALSE	TRUE	TRUE	FALSE	TRUE	TRUE	FALSE
South Asia	TRUE	TRUE	TRUE	TRUE	FALSE	TRUE	TRUE	FALSE	TRUE	TRUE	TRUE
Southern sub-Saharan Africa	TRUE	FALSE	TRUE	TRUE	TRUE	TRUE	FALSE	TRUE	TRUE	TRUE	TRUE
Tropical South America	TRUE	TRUE	TRUE	TRUE	FALSE	TRUE	TRUE	FALSE	TRUE	FALSE	TRUE
Western sub-Saharan Africa	TRUE	FALSE	TRUE	TRUE	TRUE	TRUE	FALSE	TRUE	TRUE	FALSE	TRUE

4.0. Statistical model

The technical descriptions of methods for the underlying geostatistical model are consistent with those previously used in the geospatial modelling of EBF across Africa[2].

4.1. Ensemble covariate modelling process

We implemented an ensemble covariate modelling method to select covariates and capture possible non-linear effects and complex interactions between them[32]. Our methods largely follow the approach described by Bhatt and colleagues[33] and that was previously applied to mapping child growth failure, educational attainment, under-5 mortality, diarrhoea, and lower respiratory infections[34–39].

We fit three sub-models (a generalised additive model, boosted regression trees, and lasso regression) to the exclusive breastfeeding data covariates described above. We selected these three sub-models based on ease of implementation through existing software packages, the fundamental differences in their approaches, and a proven track record in predictive accuracy[33]. Sub-models were fit in R using the mgcv, xgboost, glmnet, and caret packages.

Each sub-model was fit using five-fold cross-validation to avoid overfitting, and hyper-parameter fitting was performed to maximise predictive power. For each sub-model, we produced two sets of predictions: out-of-sample and in-sample. Out-of-sample predictions for each model were generated by compiling the predictions from the five holdouts from each cross-validation fold, and in-sample predictions were generated by re-fitting the sub-models using all available data. The out-of-sample sub-model predictions were used as explanatory covariates when fitting the geostatistical model described below, and the in-sample predictions were used when generating predictions from the geostatistical model in order to maximise data use. In both cases, the logit-transformation of the predictions was used to put these predictions on the same scale as the linear predictor in the geostatistical model.

4.2. Geostatistical model

We fit the geostatistical model below for 14 regions as defined in GBD (see Supplementary Figure 7)[40]. For each region, we write the hierarchy that defines our Bayesian model as follows:

$$ebf_i | p_i, N_i \sim \text{Binomial}(p_i, N_i)$$

$$\text{logit}(p_i) = \beta_0 + \boldsymbol{X_i \beta} + \gamma_{ci} + \epsilon_{GPi} + \epsilon_i$$

$$\sum \boldsymbol{\beta} = 1$$

$$\gamma_{ci} \sim \text{N}(0, \sigma^2_{country})$$

$$\epsilon_i \sim \text{N}(0, \sigma^2_{nug})$$

$$\epsilon_{GP} | \Sigma_{space,} \Sigma_{time} \sim \text{GP}(0, \Sigma_{space} \otimes \Sigma_{time})$$

We modelled the number of children who were categorized as "exclusive breastfed" (ebf_i) among a sample size (N_i) at space-time location i as a binomial random variable. The logit-transformed prevalence of exclusive breastfeeding (p_i) was specified as a linear combination of a regional intercept (β_0), a weighted combination of the logit-transformed predictions from the three sub-models ($\boldsymbol{X_i \beta}$), country-level random effects (γ_{ci}), a correlated spatiotemporal error term (ϵ_{GPi}), and an independent nugget (unstructured residual error) effect (ϵ_i). Weighting coefficients ($\boldsymbol{\beta}$) are constrained to sum to 1[33]. The spatial covariance, Σ_{space}, is modelled using an isotropic and stationary Matérn function[41]. The temporal covariance, Σ_{time}, is an annual autoregressive function over the 18 years represented in the model.

The intercept captures the overall mean level of EBF prevalence while the covariate effects capture the spatial and temporal variation in EBF prevalence that can be described as a function of spatial and temporal variation in the included covariates. The country random effects capture additional variation between countries, while the spatially and temporally correlated random effects capture additional variation by location (within and between countries) and time that varies smoothly in space and time. Finally, the uncorrelated error term (or nugget effect) captures any additional, non-structured variation by location and time.

The Matérn covariance function is associated with two hyperparameters, κ and τ (ν is fixed at 1), while the AR1 covariance function is associated with one hyperparameter, ρ. The following hyper-priors were set for each these parameters:

$$\theta_1 = \log(\tau) \sim \text{Normal}\left(\mu_{\theta_1}, \sigma^2_{\theta_1}\right)$$
$$\theta_2 = \log(\kappa) \sim \text{Normal}\left(\mu_{\theta_2}, \sigma^2_{\theta_2}\right)$$
$$\log((1+\rho)/(1-\rho)) \sim \text{Normal}(4, 1.2^2)$$

The prior for the temporal correlation parameter, ρ, corresponds to a mean of 0.96 and a distribution that is wide enough to include approximately 0.2 to 1 within three standard deviations of the mean. This relatively informative prior was chosen because temporal

correlation was expected to be high. μ_{θ_1}, σ_{θ_1}, μ_{θ_2}, and σ_{θ_2} were automatically determined by INLA. Priors for fixed effects and hyper-priors for other random effects were set as:

$$\beta_0 \sim \text{Normal}(0, 3^2)$$

$$1/\sigma^2_{country} \sim \text{gamma}(\text{rate} = 1, \text{shape} = 0.00005)$$
$$1/\sigma^2_{nugget} \sim \text{gamma}(\text{rate} = 1, \text{shape} = 0.00005)$$

This model was fit in R-INLA[42] using the stochastic partial differential equations (SPDE)[43] approach to approximate the continuous spatiotemporal Gaussian random fields (ϵ_{GPi}). We constructed a finite elements mesh for the SPDE approximation to the Gaussian process regression using a simplified polygon boundary (Supplementary Figure 9).

After fitting each model based on regional classification, we generated 1,000 draws of all model parameters from the approximated joint posterior distribution using the inla.posterior.sample() function in R-INLA. For each draw s of the model parameters we constructed a draw of $p_i^{(s)}$ as:

$$p_i^{(s)} = logit^{-1}\left(\beta_0^{(s)} + \boldsymbol{X_i\beta}^{(s)} + \gamma_{ci}^{(s)} + \epsilon_{GPi}^{(s)} + \epsilon_i^{(s)}\right)$$

Additional processing of the output from inla.posterior.sample() is required for the correlated spatiotemporal error term ($\epsilon_{GPi}^{(s)}$) and the nugget effect ($\epsilon_i^{(s)}$) prior to constructing $p_i^{(s)}$ according to the equation above. Specifically, for $\epsilon_{GPi}^{(s)}$, draws are generated initially only at the vertices of the finite element mesh, so we project from this mesh to each location i desired for prediction (i.e., the centroid of each grid cell on a 5 × 5-km grid) as well as years from 2000 to 2018. For the nugget effect, we generate $\epsilon_i^{(s)}$ for each i by sampling from Normal $\left(0, {\sigma_{nug}^2}^{(s)}\right)$. At the end of this process, we have 1,000 draws of p_i for each grid cell and year. Supplementary Figure 10-12 present posterior means and 95% uncertainty intervals maps for EBF in 2018 by aggregation levels (5 × 5-km, and first- and second-administrative levels).

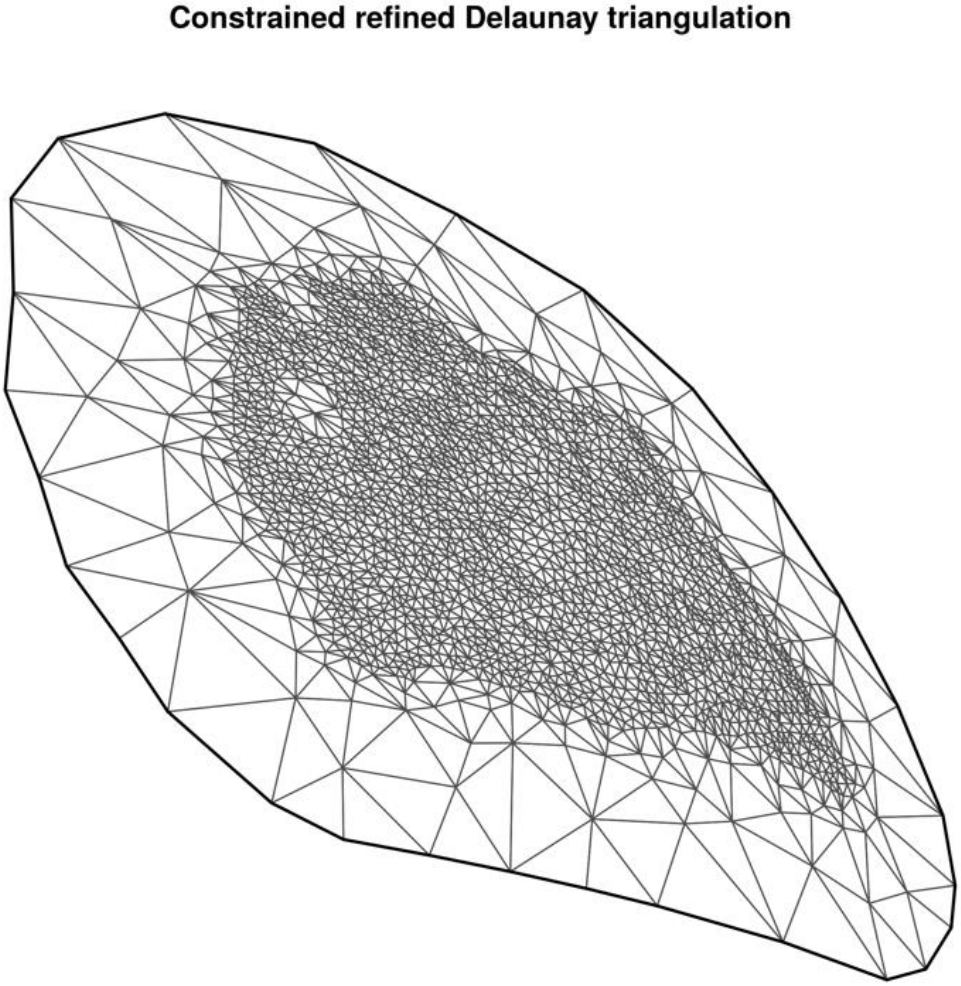

Supplementary Figure 9. Example of finite elements mesh for geostatistical models

The finite elements mesh used to fit the space-time correlated error for the eastern sub-Saharan Africa (ESSA) region overlaid on the countries in ESSA. Both the fine-scale mesh over land in the modelling region and the coarser buffer region mesh are shown.

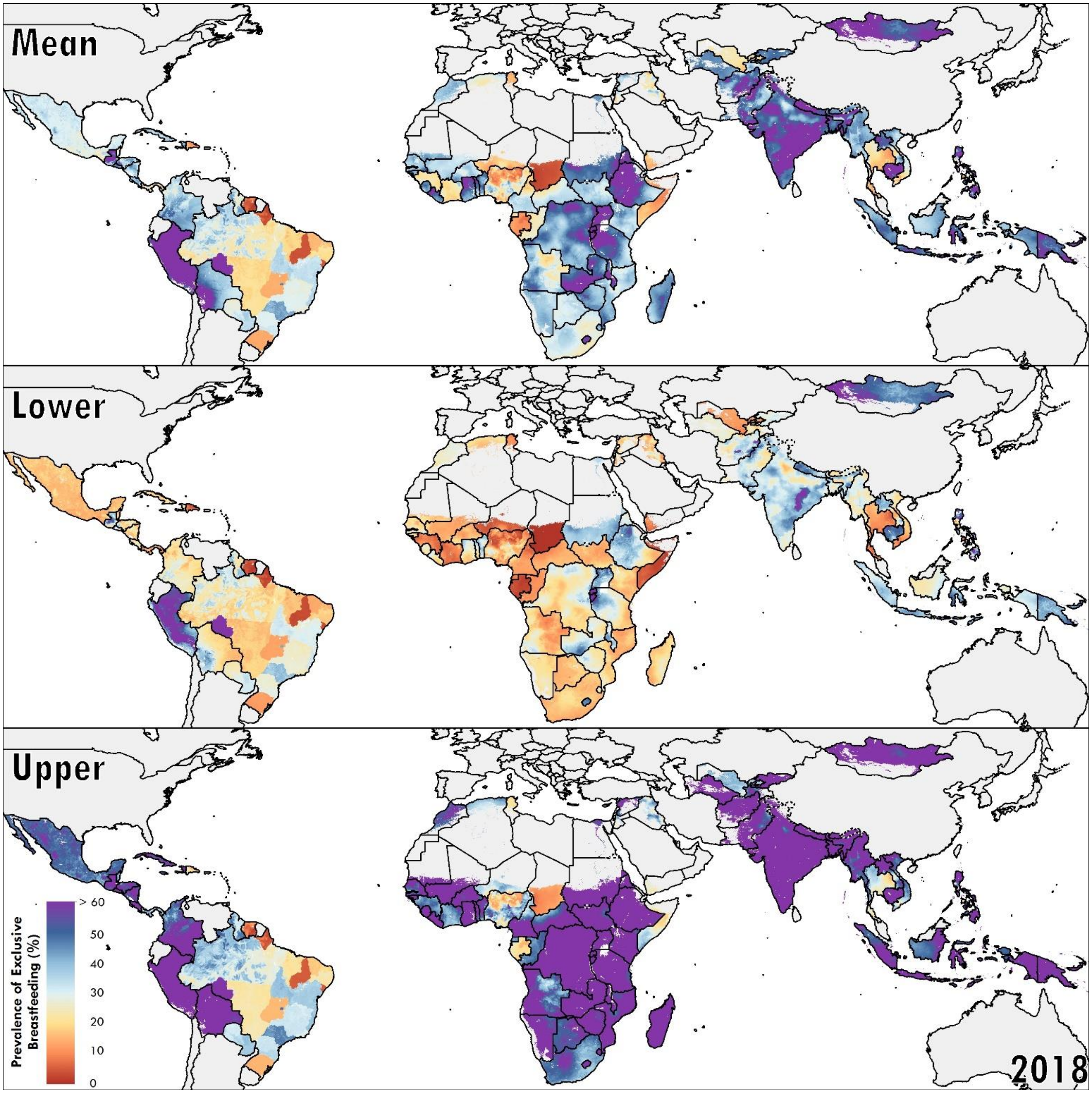

Supplementary Figure 10. Posterior means and 95% uncertainty intervals for EBF prevalence by 5 × 5-km level in 2018.

Maps reflect administrative boundaries, land cover, lakes, and population; grey-colored grid cells had fewer than ten people per 1 × 1-km grid cell and were classified as "barren or sparsely vegetated", or were not included his analysis[26–31].

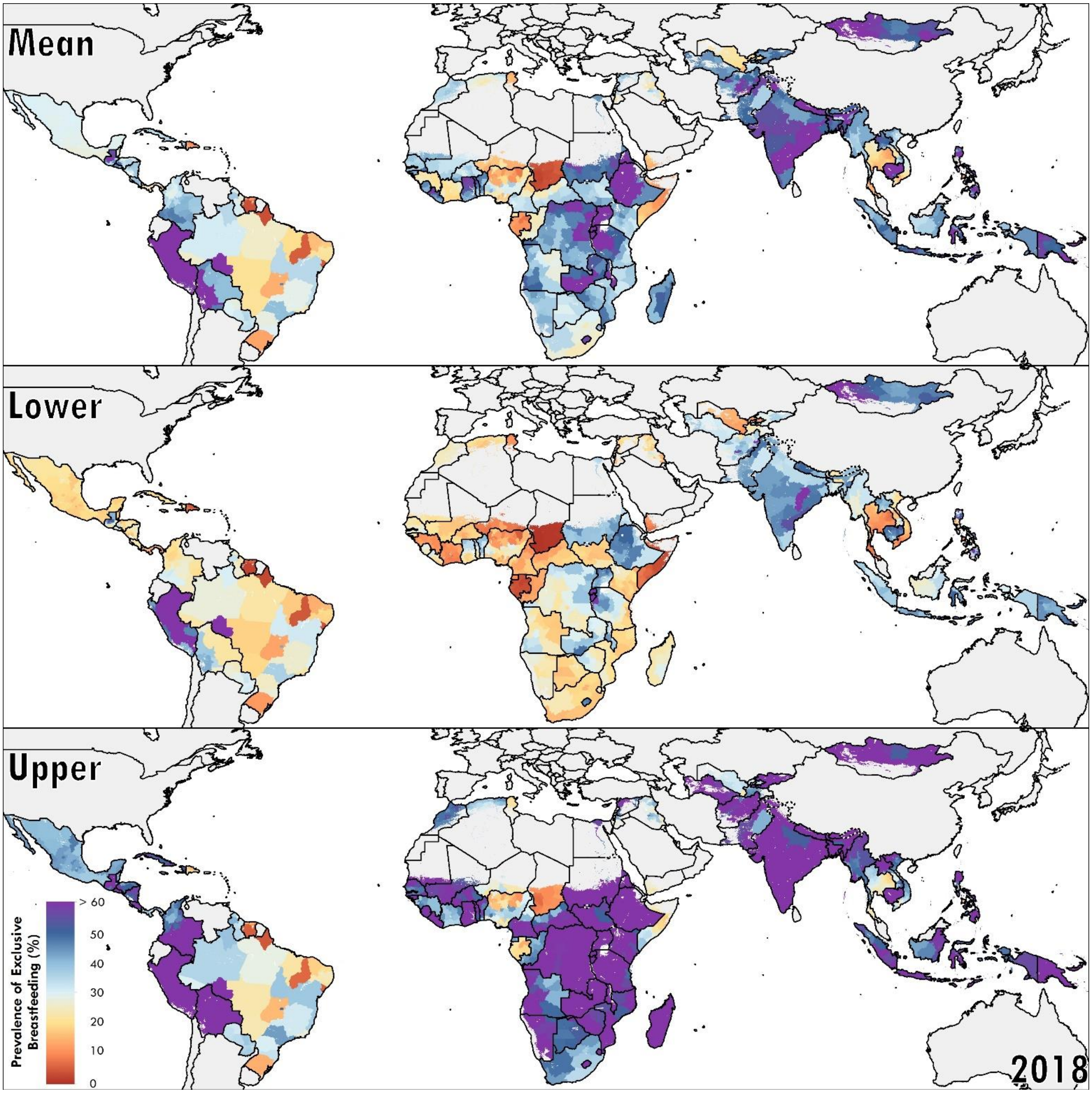

Supplementary Figure 11. Posterior means and 95% uncertainty intervals for EBF prevalence by the first administrative level in 2018.

Maps reflect administrative boundaries, land cover, lakes, and population; grey-colored grid cells had fewer than ten people per 1 × 1-km grid cell and were classified as “barren or sparsely vegetated”, or were not included in this analysis[26–31].

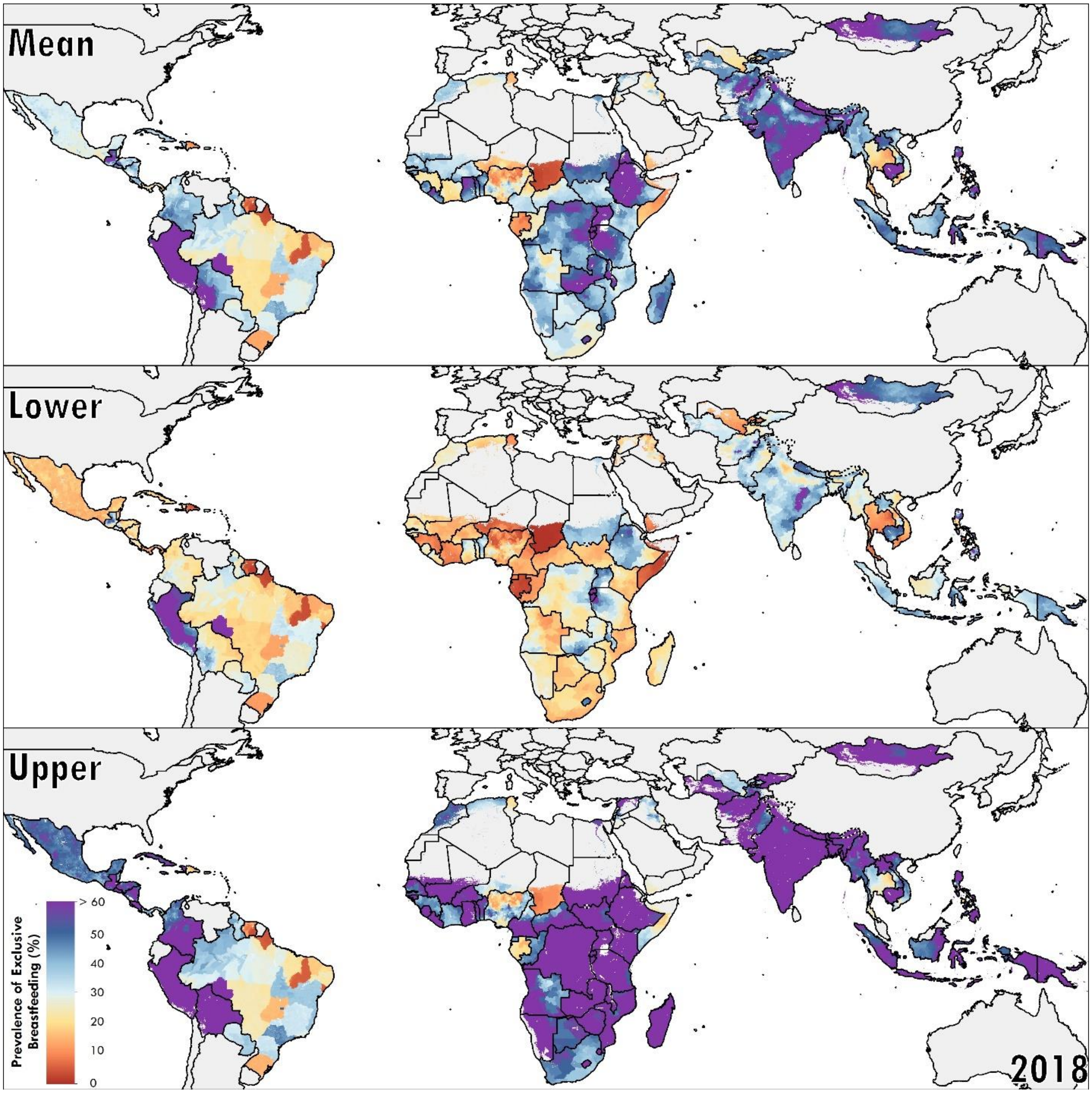

Supplementary Figure 12. Posterior means and 95% uncertainty intervals for EBF prevalence by the second administrative level in 2018.

Maps reflect administrative boundaries, land cover, lakes, and population; grey-colored grid cells had fewer than ten people per 1 × 1-km grid cell and were classified as "barren or sparsely vegetated", or were not included in this analysis[26–31].

4.3. Model validation

The technical descriptions of methods for model validation are consistent with those previously used in the geospatial modelling of EBF across Africa[2].

We utilized five-fold cross-validation in order to assess the performance of the modelling framework described above. To do so, we first split all survey data into five groups by randomly sorting a list of unique identifiers for each survey, calculating the cumulative effective sample size represented by the surveys in this list, and then dividing the list into five parts at the point where this cumulative sample size was closest to 20%, 40%, 60%, and 80% of the total. This results in five groups that are approximately equal in terms of the total effective sample size and which contain entire surveys (i.e., all of the data points derived from each survey are contained exclusively within only one fold). We then fit the model described above five times, excluding each of the five groups of data in turn.

After fitting the model five times, the data withheld from each model were matched with predictions from that model, and then these data-prediction pairs were compiled across all five models, resulting in a complete dataset of out-of-sample predictions corresponding to all survey data included in the analysis. EBF prevalence estimates based on single survey clusters are generally quite noisy due to very small sample sizes, and are consequently insufficient as a "gold standard" for evaluating the model predictions[4]. To address this issue, we aggregated both the observed data and the corresponding out-of-sample predictions within countries and within first- and second-administrative units, by calculating a weighted mean of each using the effective sample sizes as the weights. Then, across all data-estimate pairs, we calculated the following measures: the mean error (ME: a measure of bias), the mean absolute error (MAE: measure of total variation in the errors), the correlation, and the root-mean-square error (RMSE: a measure of total variance). In addition, for each data-estimate pair, we constructed 95% prediction intervals from the 2.5th and 97.5th percentiles of 1,000 draws from a binomial distribution corresponding to each of the 1,000 posterior draws of EBF prevalence with p equal to EBF prevalence in a given posterior draw and N equal to the effective sample size for the data point type. We then calculated coverage as the percentage of data-estimate pairs where the data point was contained within this 95% prediction interval. Supplementary Figures 13–15 compare in-sample EBF data and predictions aggregated to the national and subnational levels, with 95% uncertainty intervals. Supplementary Table 7 provides a summary of in-sample and out-of-sample predictive validity metrics for EBF across national, and first- and second-administrative levels.

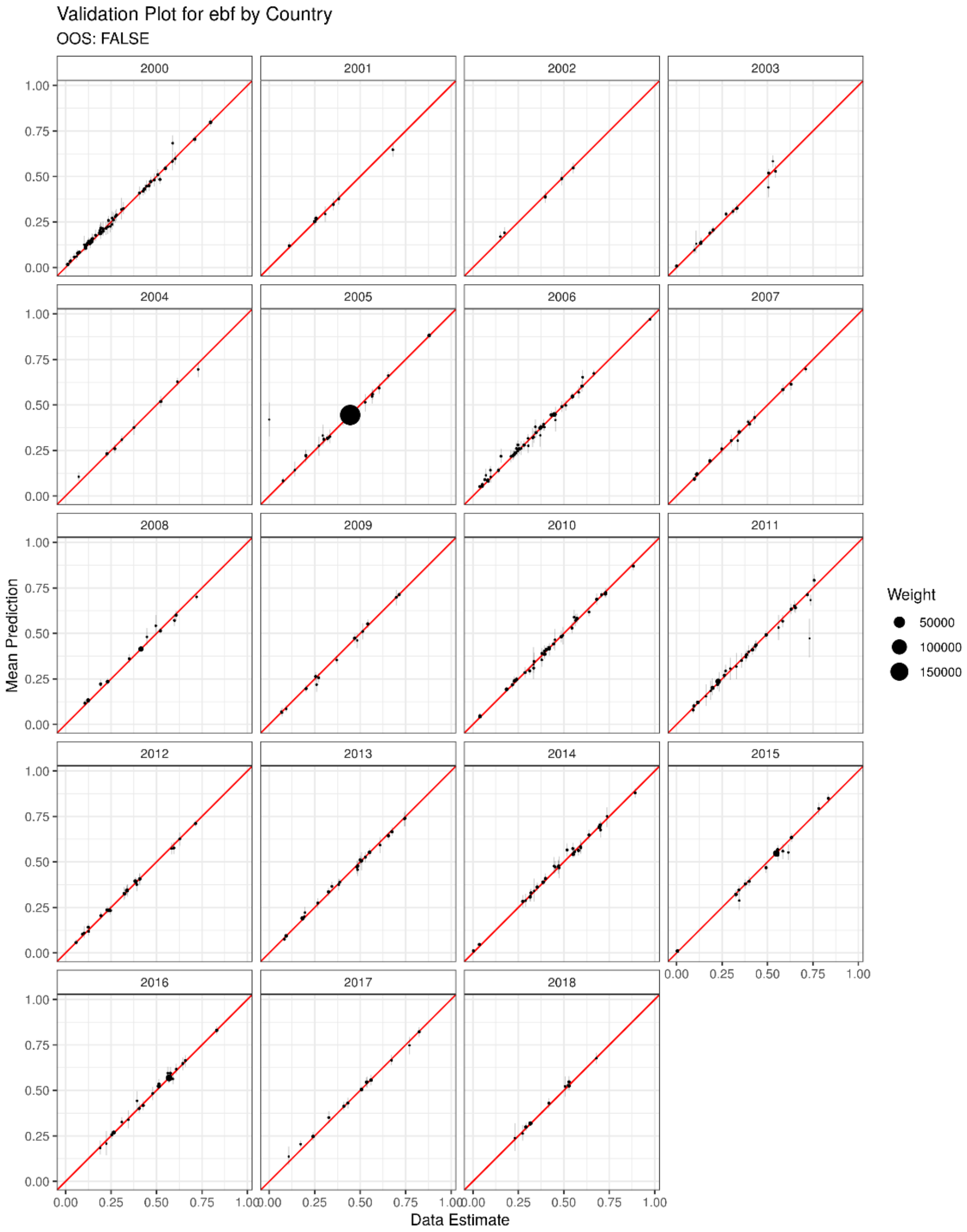

Supplementary Figure 13. In-sample comparison of data and estimates, aggregated to the national level and year

Comparison of in-sample EBF data and predictions aggregated to the national level and year, with 95% uncertainty intervals.

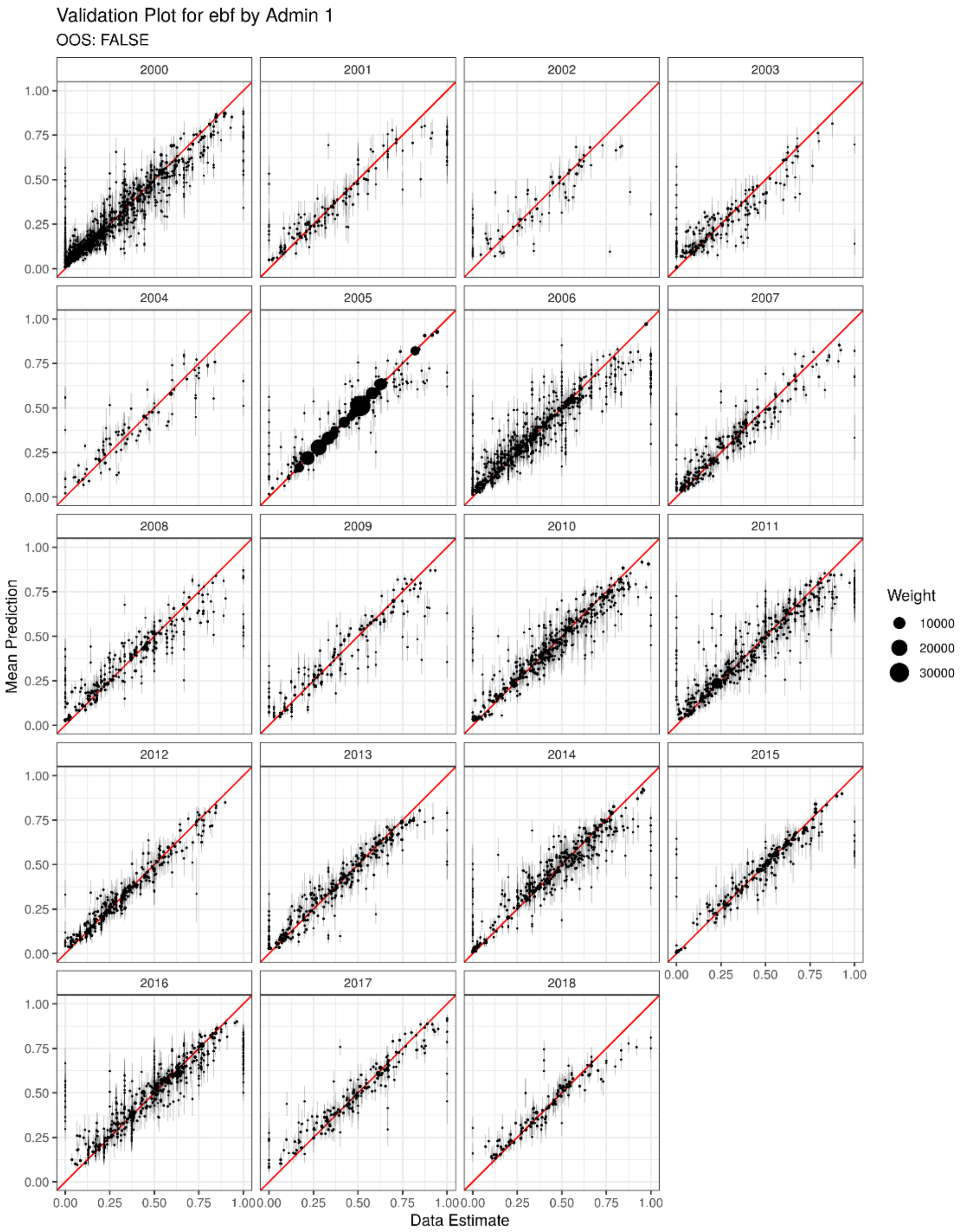

Supplementary Figure 14. In-sample comparison of data and estimates, aggregated to the first administrative level and year

Comparison of in-sample EBF data and predictions aggregated to the first administrative level and year, with 95% uncertainty intervals.

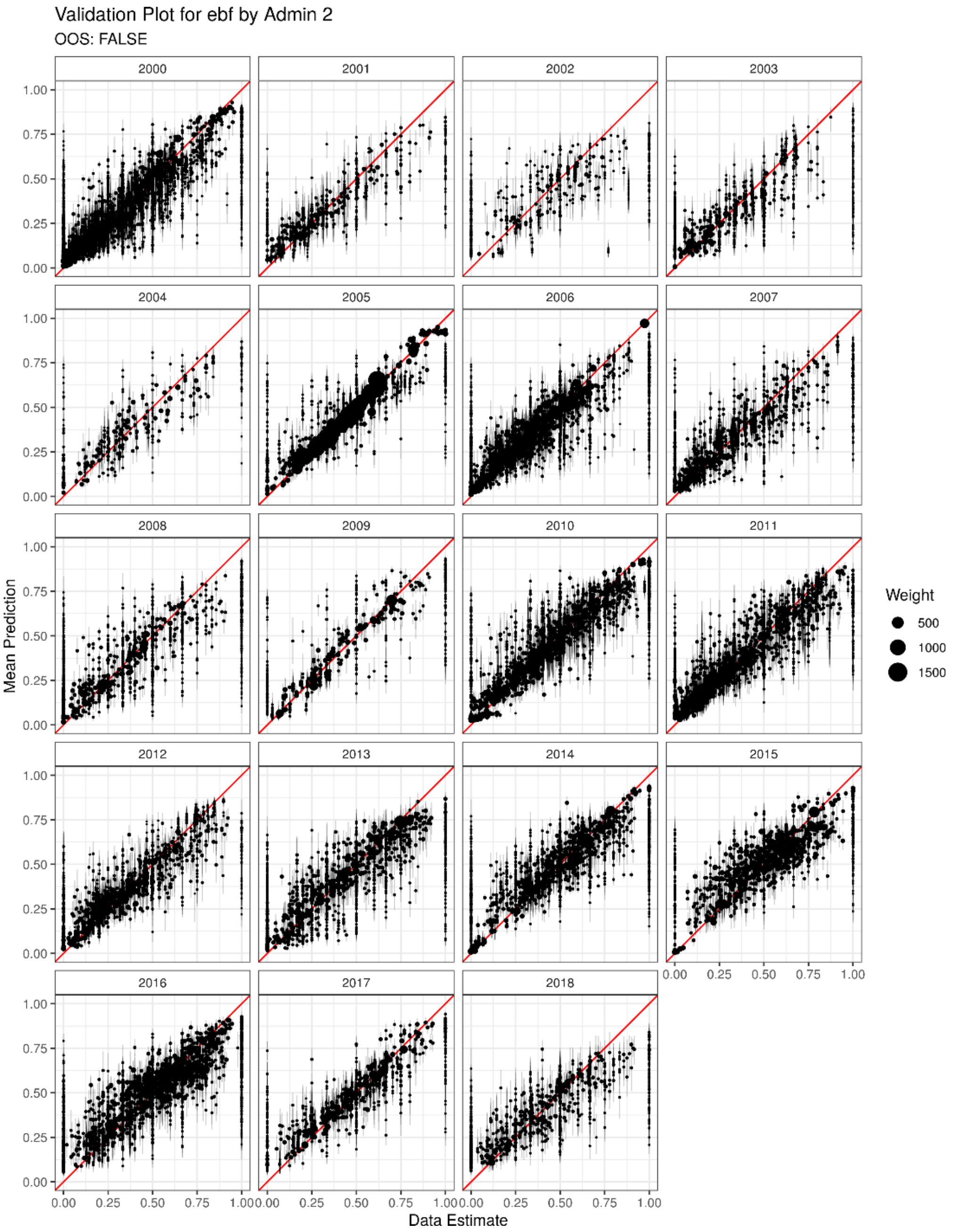

Supplementary Figure 15. In-sample comparison of data and estimates, aggregated to the second administrative level and year

Comparison of in-sample EBF data and predictions aggregated to the second administrative level and year, with 95% uncertainty intervals.

Supplementary Table 7. Validation metrics by level of aggregation

Aggregation level	IS/OOS	ME	RMSE	Correlation	Coverage (%)
National level	IS	-0.0640	1.0942	0.9985	97.6060
	OOS	-0.1730	11.4750	0.9313	91.8937
First administrative level	IS	-0.0640	6.1229	0.9639	97.6060
	OOS	-0.1730	15.4702	0.9056	91.8937
Second administrative level	IS	-0.0640	12.0638	0.8831	97.6060
	OOS	-0.1730	20.1755	0.8116	91.8937

4.4. Post-estimation

The technical descriptions of methods for post-estimation are consistent with those previously used in the geospatial modelling of EBF across Africa[2].

4.4.1. Calibration to Global Burden of Disease 2019

To take advantage of the extensive data-gathering and analysis of GBD 2019[1], which included, in some cases, data sources outside of the scope of our geospatial modelling framework, we preformed post-hoc calibration of our estimates to the GBD estimates.

First, each grid cell in our 5 × 5-km grid was assigned to a GBD geography based on the location of the grid-cell centroid. Then, for each country and year, we defined a raking factor that was the ratio of the GBD estimate for this geography and year to the population-weighted posterior mean EBF prevalence in all grid cells within this geography and year. Finally, this raking factor was used to scale each draw of EBF prevalence for each grid cell within the GBD geography and year. National time series plots of the post-GBD calibration final estimates (including uncertainty ranges) are presented along with the aggregated input data (classified by survey series, data type, and sample size) in Extended Data Figure 2.

Point estimates for each 5 × 5-km grid cell were calculated as the mean of the scaled draws, and 95% uncertainty intervals were calculated as the 2.5th and 97.5th percentiles of the scaled draws. An example of relative uncertainty in EBF estimates map for 2018 is given in Extended Data Figure 3.

4.4.2. Aggregation to first- and second-level administrative units

In addition to estimates of EBF prevalence on a 5 × 5-km grid, we also constructed estimates of EBF prevalence for first- (admin 1) and second-level (admin 2) administrative units. These estimates were derived by calculating population-weighted averages of EBF prevalence of estimates for each grid cell within a given first- or second-level administrative unit. This was carried out for each of the 1,000 posterior draws (after calibration to GBD 2019[1] as described above), and then point estimates and uncertainty intervals were derived from the mean, 2.5th percentile, and 97.5th percentile of these draws, respectively. In cases where an administrative unit did not contain the centroid of any grid cell, the nearest grid cell to it was assigned as its proxy prevalence.

4.4.3. Geographic Inequality

We calculated national-level geographic inequality using the Gini coefficient. The Gini coefficient assesses the magnitude of disparity between the richest and poorest individuals (ref). The Gini coefficient can be calculated directly from the Lorenz curve, which sorts individuals by their income and plots cumulative percentages of individuals against their corresponding fraction of wealth. For the purposes of this study, "wealth" is defined as EBF prevalence in each second administrative unit. The Gini coefficient is then calculated as one minus twice the area under the Lorenz curve. An alternative formulation of the Gini coefficient, which gives the same result, calculates the relative mean absolute difference in wealth, and then observes that the Gini coefficient is half the resulting quantity. If x_i is the wealth of the ith individual (out of n individuals), the Gini coefficient, G, is given as:

$$G = \frac{\sum_{i=1}^{n} \sum_{j=1}^{n} |x_i - x_j|}{2n \sum_{i=1}^{n} x_i}$$

Absolute inequality is calculated as absolute differences between districts with the highest and lowest EBF prevalence in each country:

$$Absolute\ inequality = \max(prev_{unit}) - \min(prev_{unit})$$

Relative inequality is calculated as absolute difference between each unit and country mean relative to the mean:

$$Relative\ inequality = \frac{prev_{unit} - prev_{country}}{prev_{country}}$$

4.4.4. Projections

We compared our estimated rates of improvement in EBF prevalence over the last 18 years with the improvements needed between 2018 and 2025 to meet the WHO GNT by performing a simple projection calculation. We first calculated log-additive annual rates of change at each grid cell (*i*) by logit-transforming our 18 years of posterior mean prevalence estimates, $prev_{i,yr}^{l}$ and calculating the annual rate of change between each pair of adjacent years starting with 2001:

$$AROC_{i,yr}^{l} = prev_{i,yr}^{l} - prev_{i,yr-1}^{l}.$$

We then calculated a weighted AROC for each grid cell by taking a weighted average across the years, where more recent AROCs are given more weight in the average. We defined the weights to be:

$$w_{yr} = \frac{(yr - 2000)^{\gamma}}{\sum_{2001}^{2018}(yr - 2000)^{\gamma}},$$

where γ may be chosen to give varying amounts of weight across the years. For this set of projections we selected $\gamma = 1$, resulting in a linear weighting scheme that has been tested and vetted for use in projecting the health-related Sustainable Development Goal (SDG)[6]. For any grid cell, we then calculated the weighted AROC to be:

$$AROC_i = \sum_{2001}^{2018} w_{yr} AROC_{i,yr}^{l}$$

Finally, we calculated the projections by applying the weighted AROC at each grid cell to our mean 2018 mean prevalence estimates:

$$Proj_{i,2025} = logit^{-1}(prev_{i,2018}^{l} + AROC_{i,j} \times 8).$$

We use the same process to project country-level and admin-level AROCs.

5.0. Supplementary Results

5.1. National differences in rates of change from 2000 to 2018

Supplementary Table 8. Countries with annualized increases and decreases in all districts

(a) Increases

Countries with annualised increases in all districts *(28 LMICs)*	Countries with annualised increases >2.5% in all districts *(25 LMICs)*	Countries with annualised increases >5% in all districts *(14 LMICs)*
Angola, Bangladesh, Belize, Botswana, Burkina Faso, Burundi, Cambodia, Côte d'Ivoire, Eritrea, Eswatini, Guinea-Bissau, Jamaica, Kenya, Lesotho, Liberia, Mauritania, Morocco, Myanmar, Namibia, Nepal, Palestine, São Tomé and Príncipe, Sierra Leone, South Africa, Sudan, Timor-Leste, Turkmenistan, Zimbabwe	Angola, Bangladesh, Belize, Botswana, Burkina Faso, Burundi, Cambodia, Côte d'Ivoire, Eritrea, Eswatini, Guinea-Bissau, Kenya, Lesotho, Liberia, Mauritania, Morocco, Myanmar, Namibia, Palestine, São Tomé and Príncipe, Sierra Leone, South Africa, Sudan, Turkmenistan, Zimbabwe	Angola, Burkina Faso, Côte d'Ivoire, Eritrea, Guinea-Bissau, Kenya, Mauritania, Myanmar, Namibia, Sierra Leone, South Africa, Sudan, Turkmenistan, Zimbabwe

(b) Decreases **(c) Decreases and Increases**

Countries with annualised decreases in the majority of districts *(13 LMICs)*	Countries with annualised decreases in all districts *(1 LMIC)*	Countries with districts with annualised decreases (≤-2.5%) and annualised increases (>5%) *(7 LMICs)*
Afghanistan, Bolivia, Brazil, Chad, Colombia, Comoros, Dominican Republic, Equatorial Guinea, Haiti, Jordan, Madagascar, Pakistan, Uzbekistan	Chad	India, Mozambique, Niger, Nigeria, Philippines, Somalia, Thailand

5.2. Achievement of the original WHO GNT (50% EBF) by 2018 and 2025

We mapped probabilities of LMICs meeting the original WHO GNT of ≥50% EBF by 2018 and 2025 (Supplementary Figures 16–18 and Figure 6). By 2018, more than half of LMICs (53.2% (50 of 94)) had low probability (<5%) of having already met the original WHO GNT of ≥50% EBF at a national level, while 27.7% (26 of 94) and 19.1% (18 of 94) of LMICs had low probability of having met this target in all their first-administrative level (e.g., provinces) and second-administrative-level units (e.g., districts), respectively. Overall, 11 LMICs had a high probability (>95%) of having already met the 50% EBF WHO GNT in 2018 at the national level: Burundi, Ethiopia, India, Cambodia, Lesotho, Mongolia, Nepal, Peru, Papua New Guinea, Rwanda and Uganda. Only 2 LMICs (Burundi and Rwanda), however, had a high probability of having met the 50% target in 2018 in all their provinces and districts. Across LMICs, 58.2% (14,286 of 24,556) of districts located in 63 LMICs had a low probability, and 5.7% (1,396 of 24,556) of districts in 25 LMICs had a high probability of having met the 50% EBF prevalence target in 2018. Ten LMICs had district-level units with both high and low probability of having met the 50% target in 2018: Afghanistan, Brazil, Guatemala, Indonesia, India, Laos, Pakistan, the Philippines, Sudan, and Tanzania.

Estimating probability of achieving the original WHO GNT of 50% EBF prevalence, 35.1% (33 of 94), 12.3% (12 of 94), and 8.5% (8 of 94) of LMICs are expected to have a low probability (<5%) of meeting this target by 2025 (the original target year) nationally, and in all of their province- and district-level units, respectively. On the other hand, 10 LMICs had a high probability (>95%) of achieving the 50% target in 2025 at the national level: Burundi, Indonesia, India, Cambodia, Lesotho, Mongolia, Nepal, Rwanda, Sudan. Subnationally, only 3 LMICs (Burundi, Lesotho, Rwanda) have a high probability of meeting the 50% target by 2025 in all provinces, as well as in all districts. Across the 94 LMICs in our analysis, 4.2% (1,042 of 24,556) of districts located in 23 LMICs have a high probability, while 40.2% (9,861 of 24,556) of districts in 42 LMICs have a low probability of meeting the ≥50% target by 2025. Broad inequalities are expected to continue and eight LMICs are expected to have both high and low probabilities of achieving the 50% target by 2025 in their district-level units: Afghanistan, Benin, Brazil, Indonesia, India, Laos, Pakistan, and the Philippines.

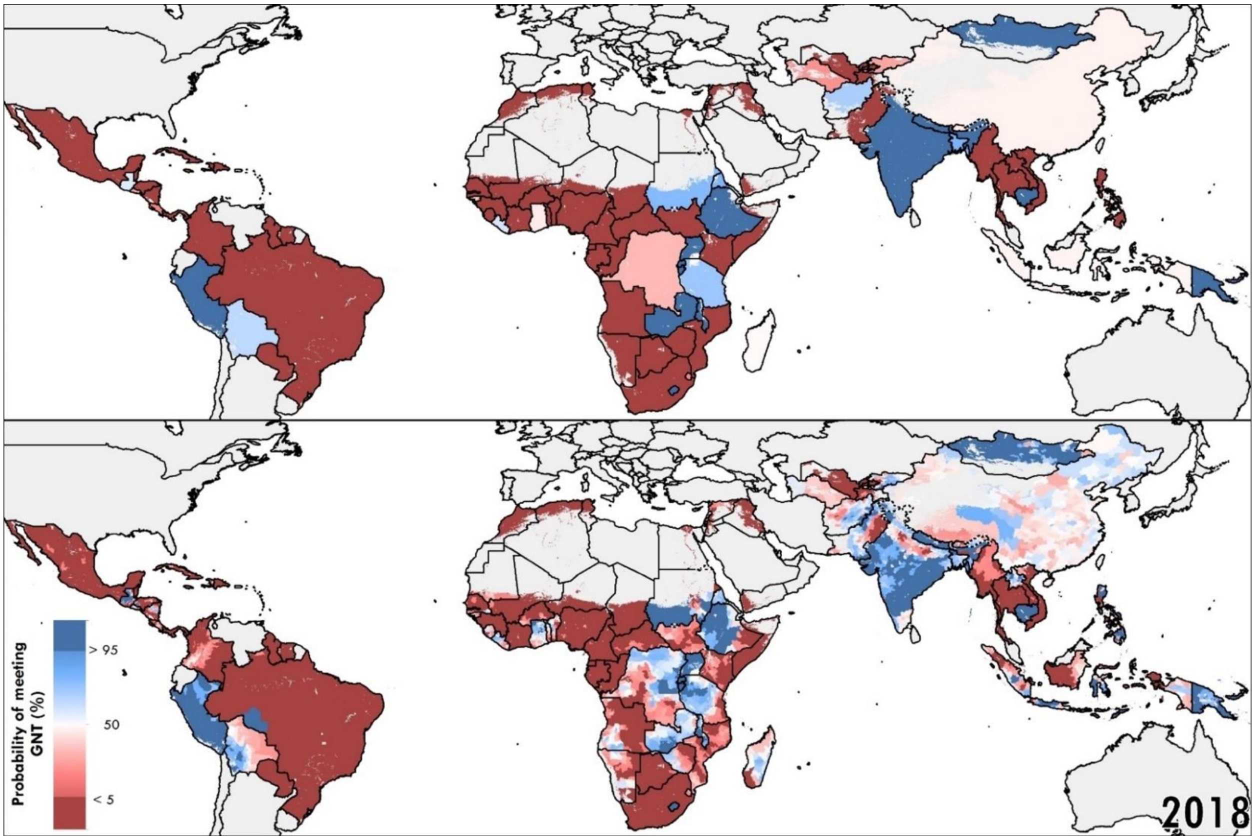

Supplementary Figure 16. Probability of meeting the ≥50% WHO GNT for EBF in 2018

a–**b**, Probability of having met the WHO GNT of at least 50% exclusive breastfeeding prevalence in 2018 at the national level (**a**) and second administrative level (**b**). Dark blue indicates a high probability (>95% posterior probability) and dark red indicates a low probability (<5% posterior probability) of having met the WHO GNT by 2018. Maps reflect administrative boundaries, land cover, lakes, and population; grey-colored grid cells had fewer than ten people per 1 × 1-km grid cell and were classified as "barren or sparsely vegetated", or were not included in this analysis[26–31].

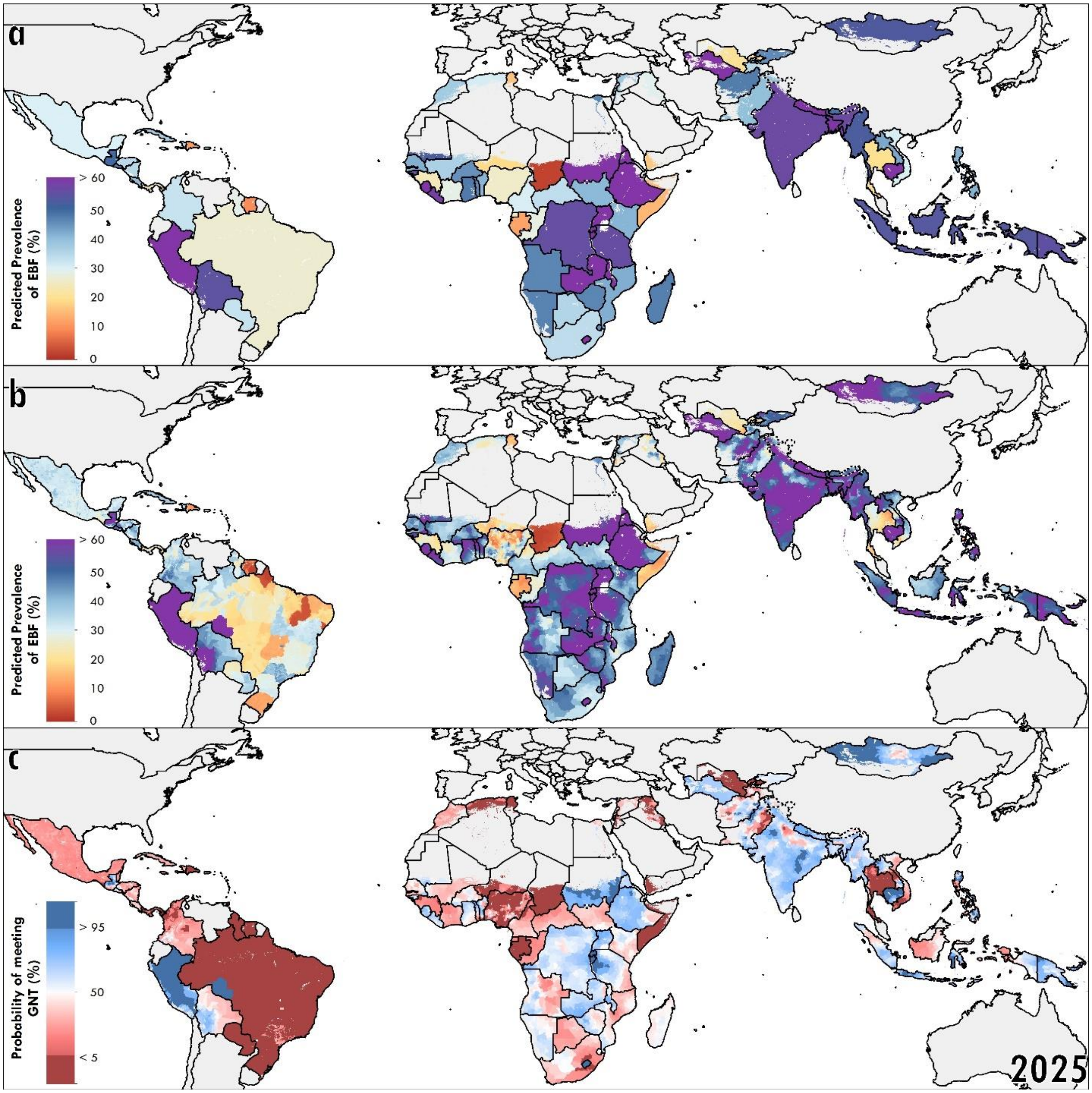

Supplementary Figure 17. Projected prevalence for exclusive breastfeeding for 2025 and probability of meeting the WHO GNT by 2025

a–**b**, Projected exclusive breastfeeding prevalence for 2030 at the national (**a**) and second administrative level (**b**). **c**, Probability of meeting the WHO GNT of at least 70% exclusive breastfeeding prevalence by 2030 at the second administrative level. Dark blue indicates a high probability (>95% posterior probability) and dark red indicates a low probability (<5% posterior probability) of meeting the WHO GNT by 2030. Maps reflect administrative boundaries, land cover, lakes, and population; grey-colored grid cells had fewer than ten people per 1 × 1-km grid cell and were classified as "barren or sparsely vegetated", or were not included in this analysis[26–31].

Supplementary Table 9. Countries and administrative units achieving the original WHO GNT of 50% prevalence of EBF with high and low probabilities

(a) High probability (>95% posterior probability) of achieving the original WHO GNT of 50% prevalence of EBF

Region	High probability in meeting the WHO GNT at National or in all Administrative units by 2018			High probability in meeting the WHO GNT at National or in all Administrative units by 2025		
	National	All Admin 1 Units	All Admin 2 Units	National	All Admin 1 Units	All Admin 2 Units
**Andean South America**	Peru			Peru		
**Eastern sub-Saharan Africa**	Burundi, Ethiopia, Rwanda, Uganda,	Burundi, Rwanda	Burundi, Rwanda	Burundi, Rwanda,	Burundi, Rwanda	Burundi, Rwanda
**Southern sub-Saharan Africa**	Lesotho			Lesotho	Lesotho	Lesotho
**North Africa**				Sudan		
**South Asia**	India, Nepal			India, Nepal		
**Southeast Asia**	Cambodia			Cambodia		
**East Asia**	Mongolia			Mongolia		
**Oceania**	Papua New Guinea			Indonesia		
The regions of Western sub-Saharan Africa, Central sub-Saharan Africa, and Central Asia did not have any countries that had a high probability of achieving the original WHO GNT of 50% EBF in 2018 or 2025 at the national level or in all first or all second administrative-level units.						

(b) Low probability (<5% posterior probability) of achieving the original WHO GNT of 50% prevalence of EBF

Region	Low probability in meeting the WHO GNT at National or in all Administrative units by 2018			Low probability in meeting the WHO GNT at National or in all Administrative units by 2025		
	**National**	**All Admin 1 Units**	**All Admin 2 Units**	**National**	**All Admin 1 Units**	**All Admin 2 Units**
**Central America and the Caribbean**	Belize, Cuba, Dominican Republic, Honduras, Haiti, Jamaica, Mexico, Nicaragua, Panama	Belize, Dominican Republic, Mexico, Panama	Belize, Dominican Republic, Panama	Belize, Dominican Republic, Mexico, Panama	Dominican Republic	Dominican Republic
**Andean South America**	Colombia, Trinidad and Tobago	Trinidad and Tobago	Trinidad and Tobago	Colombia, Trinidad and Tobago	Trinidad and Tobago	Trinidad and Tobago
**Tropical South America**	Brazil, Guyana, Paraguay, Suriname	Guyana, Paraguay, Suriname	Guyana, Paraguay, Suriname	Brazil, Guyana, Paraguay, Suriname	Guyana , Paraguay, Suriname	Paraguay, Suriname
**Western sub-Saharan Africa**	Benin, Chad, Côte d'Ivoire, Cameroon, Guinea, Gambia, Mali, Niger, Nigeria, Senegal	Chad, Côte d'Ivoire, Niger,	Chad, Niger	Benin, Cameroon, Chad, Guinea, Niger, Nigeria	Chad	Chad
**Central sub-Saharan Africa**	Angola, Central African Republic, Comoros, Equatorial Guinea, Gabon, Republic of the Congo, Somalia, Yemen	Comoros, Equatorial Guinea, Gabon, Republic of the Congo, Somalia, Yemen	Comoros, Equatorial Guinea, Gabon, Somalia	Comoros, Equatorial Guinea, Gabon, Republic of the Congo, Somalia, Yemen	Comoros, Gabon, Somalia, Yemen	Comoros, Gabon
**Southern sub-Saharan Africa**	Botswana, South Africa,	South Africa		South Africa		

**North Africa**	Algeria, Morocco, Tunisia	Algeria, Tunisia	Algeria, Tunisia	Algeria, Tunisia	Tunisia	Tunisia
**Middle East**	Iraq, Jordan, Palestine, Syria	Iraq, Jordan, Palestine	Jordan, Palestine	Iraq, Jordan, Palestine		
**Central Asia**	Pakistan, Tajikistan, Uzbekistan	Tajikistan, Uzbekistan	Uzbekistan	Pakistan, Tajikistan, Uzbekistan	Uzbekistan	Uzbekistan
**Southeast Asia**	Laos, Myanmar, Thailand, Vietnam	Thailand		Thailand, Vietnam		
**Oceania**	The Philippines					
The regions of East Asia and South Asia did not have any countries that had a low probability of achieving the original WHO GNT of 50% EBF in 2018 or 2025 at the national level or in all first or all second administrative-level units.						

5.3. Achievement of the updated WHO GNT (70% EBF) by 2030

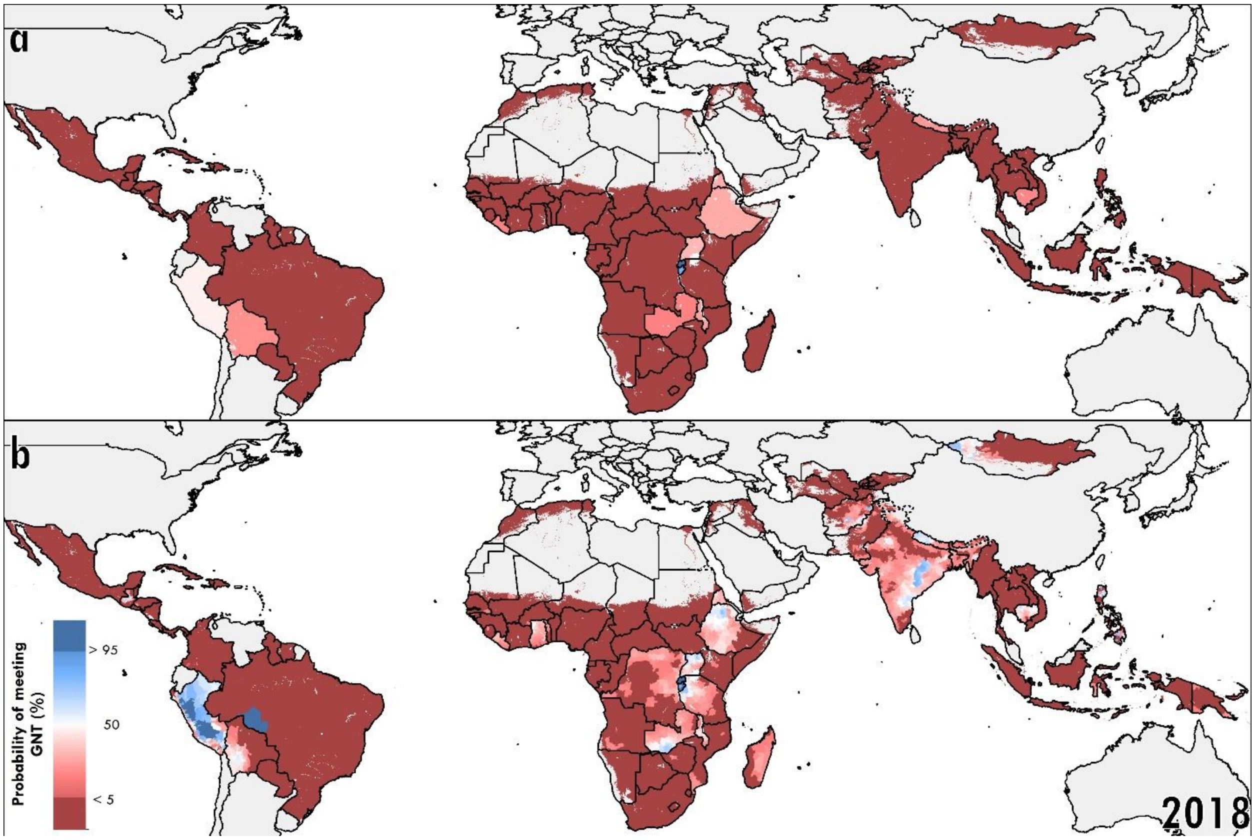

Supplementary Figure 18. Probability of meeting the ≥70% WHO GNT for EBF in 2018

a–**b**, Probability of having met the WHO GNT of at least 70% exclusive breastfeeding prevalence in 2018 at the national level (**a**) and second administrative level (**b**). Dark blue indicates a high probability (>95% posterior probability) and dark red indicates a low probability (<5% posterior probability) of having met the WHO GNT by 2018. Maps reflect administrative boundaries, land cover, lakes, and population; grey-colored grid cells had fewer than ten people per 1 × 1-km grid cell and were classified as “barren or sparsely vegetated”, or were not included in this analysis[26–31].

Supplementary Table 10. Countries and administrative units achieving the updated WHO GNT of 70% prevalence of EBF with high and low probabilities

(a) High probability (>95% posterior probability) of achieving the updated WHO GNT of 70% prevalence of EBF

**Region**	**High probability in meeting the WHO GNT at National or in all Administrative units by 2018**			**High probability in meeting the WHO GNT at National or in all Administrative units by 2030**		
	**National**	**All Admin 1 Units**	**All Admin 2 Units**	**National**	**All Admin 1 Units**	**All Admin 2 Units**
**Eastern sub-Saharan Africa**	Rwanda	Rwanda				
The regions of Andean South America, Central America and the Caribbean, Tropical South America, Western sub-Saharan Africa, Central sub-Saharan Africa, Southern sub-Saharan Africa, North Africa, Middle East, Central Asia, South Asia, Southeast Asia, East Asia, and Oceania did not have any countries that had a high probability of achieving the updated WHO GNT of 70% in 2018 or 2030 at the national level or in all first or all second administrative-level units.						

(b) Low probability (<5% posterior probability) of achieving the updated WHO GNT of 70% prevalence of EBF

Region	Low probability in meeting the WHO GNT at National or in all Administrative units by 2018			Low probability in meeting the WHO GNT at National or in all Administrative units by 2030		
	**National**	**All Admin 1 Units**	**All Admin 2 Units**	**National**	**All Admin 1 Units**	**All Admin 2 Units**
**Central America and the Caribbean**	Belize, Costa Rica, Cuba, Dominican Republic, Guatemala, Honduras, Haiti, Jamaica, Mexico, Nicaragua, Panama, El Salvador	Belize，Costa Rica，Cuba，Dominican Republic，Honduras，Haiti，Jamaica，Mexico，Nicaragua，Panama，El Salvador	Belize, Costa Rica, Cuba, Dominican Republic, Haiti, Jamaica, Mexico, Nicaragua, Panama	Belize, Cuba, Dominican Republic, Honduras, Haiti, Jamaica, Mexico, Nicaragua, Panama	Belize, Dominica n Republic, Mexico, Panama	Belize, Dominica n Republic
**Andean South America**	Colombia, Trinidad and Tobago	Colombia，Trinidad and Tobago	Trinidad and Tobago	Colombia, Trinidad and Tobago	Trinidad and Tobago	Trinidad and Tobago
**Tropical South America**	Brazil, Guyana, Paraguay, Suriname	Guyana，Paraguay, Suriname	Guyana, Paraguay, Suriname	Brazil, Guyana, Paraguay, Suriname	Guyana, Paraguay, Suriname	Guyana, Paraguay, Suriname
**Eastern sub-Saharan Africa**	Comoros, Kenya, Madagascar, Mozambique, Somalia, South Sudan, Tanzania, Yemen	Comoros, Somalia, South Sudan, Yemen	Comoros, Somalia, Yemen	Comoros, Somalia, Yemen	Comoros, Somalia, Yemen	Comoros
**Western sub-Saharan Africa**	Benin, Burkina Faso, Côte d'Ivoire, Cameroon, Ghana, Guinea, Gambia, Guinea-Bissau,	Benin, Burkina Faso, Côte d'Ivoire, Cameroon, Guinea, Gambia, Mali, Mauritania, Niger,	Benin, Côte d'Ivoire, Cameroon, Guinea, Gambia, Mali, Niger, Nigeria, Chad	Benin, Côte d'Ivoire, Cameroon, Guinea, Gambia, Niger, Nigeria, Senegal, Chad	Nigeria, Chad	Chad

	Mali, Mauritania, Niger, Nigeria, Senegal, Sierra Leone, Chad, Togo	Nigeria, Sierra Leone, Chad				
**Central sub-Saharan Africa**	Angola, Central African Republic, Democratic Republic of the Congo, Republic of Congo, Gabon, Equatorial Guinea	Central African Republic, Republic of Congo, Gabon, Equatorial Guinea	Central African Republic, Republic of Congo, Gabon, Equatorial Guinea	Central African Republic, Republic of Congo, Gabon, Equatorial Guinea	Gabon	Gabon
**Southern sub-Saharan Africa**	Botswana, Eswatini, Lesotho, Namibia, South Africa, Zimbabwe	Botswana, Eswatini, Namibia, South Africa, Zimbabwe	Botswana, Eswatini, Namibia, South Africa	Botswana, South Africa		
**North Africa**	Algeria, Egypt, Morocco, Sudan, Tunisia	Algeria, Egypt, Morocco, Sudan, Tunisia	Algeria, Egypt, Morocco, Sudan, Tunisia	Algeria, Egypt, Morocco, Tunisia	Algeria, Morocco, Tunisia	Tunisia
**Middle East**	Iraq, Jordan, Palestine, Syria	Iraq, Jordan, Palestine, Syria	Iraq, Jordan, Palestine, Syria	Iraq, Jordan, Palestine, Syria	Iraq, Jordan, Palestine	Jordan, Palestine
**Central Asia**	Afghanistan, Kyrgyzstan, Pakistan, Tajikistan, Turkmenistan, Uzbekistan	Kyrgyzstan, Pakistan, Tajikistan, Turkmenistan, Uzbekistan	Tajikistan, Turkmenistan, Uzbekistan	Afghanistan, Pakistan, Tajikistan, Uzbekistan	Uzbeki-stan	Uzbeki-stan
**South Asia**	Bangladesh, Bhutan, India			India		

**Southeast Asia**	Laos, Myanmar, Thailand, Vietnam	Laos, Myanmar, Thailand, Vietnam	Myanmar, Thailand, Vietnam	Laos, Thailand, Vietnam	Thailand	
**East Asia**	Mongolia			Mongolia		
**Oceania**	Indonesia, The Philippines, Papua New Guinea, Timor-Leste	Timor-Leste	Timor-Leste	Indonesia, the Philippines, Papua New Guinea		
All regions had at least one country that had a low probability of achieving the updated WHO GNT of 70% EBF in 2018 or 2030 at the national level or in all first or all second administrative-level units.						

5.4. Global Breastfeeding Scorecard (GBS) Exemplars

Supplementary Table 11. Countries meeting and not meeting GBS[44] criteria

(a) Meeting GBS Criteria

$4.70 per newborn on breastfeeding support programs *(6 LMICs)*	Comprehensive Code Legislation *(24 LMICs)*	Met minimum recommendations of 14 weeks paid maternity leave and appropriate workplace nursing areas *(6 LMICs)*	Individual breastfeeding counselling in all primary healthcare facilities *(28 LMICs)*	≥50% births in Baby Friendly hospitals and maternities *(6 LMICs)*	Implemented community programs in all districts *(29 LMICs)*
Guinea-Bissau, Haiti, Nepal, Rwanda, Somalia, Timor-Leste	Afghanistan, Bangladesh, Benin, Bolivia, Botswana, Brazil, Dominican Republic, Gabon, Gambia, Ghana, India, Madagascar, Mozambique, Nepal, Pakistan, Panama, Peru, Philippines, South Africa, Uganda, Tanzania, Vietnam, Yemen, Zimbabwe	Colombia, Cuba, India, Paraguay, Tajikistan, Vietnam	Bhutan, Burkina Faso, Comoros, Côte d'Ivoire, El Salvador, Eritrea, Gambia, Ghana, Indonesia, Jamaica, Lesotho, Liberia, Malawi, Mexico, Morocco, Mozambique, Namibia, Nepal, Rwanda, São Tomé and Príncipe, South Africa, Suriname, Timor-Leste, Turkmenistan, Uganda, Uzbekistan, Vietnam, Zambia	Costa Rica, Cuba, Eswatini, Tajikistan, Thailand, Turkmenistan	Bhutan, Brazil, Côte d'Ivoire, Cuba, Eritrea, Eswatini, Ethiopia, Guatemala, Guyana, Honduras, Jamaica, Kyrgyzstan, Lesotho, Liberia, Madagascar, Malawi, Morocco, Mozambique, Nepal, the Philippines, Rwanda, São Tomé and Príncipe, Sierra Leone, South Africa, Suriname, Timor-Leste, Uganda, Vietnam, Zambia

(b) Not meeting GBS Criteria[44]

<$1 per newborn on breastfeeding support programs *(50 LMICs)*	No legal measures protecting against BMS marketing *(25 LMICs)*
Algeria, Angola, Belize, Benin, Botswana, Brazil, Cameroon, Central African Republic, Chad, Colombia, Costa Rica, Côte d'Ivoire, Cuba, Democratic Republic of the Congo, Dominican Republic, Egypt, El Salvador, Eritrea, Eswatini, Gabon, Guatemala, Guinea, India, Indonesia, Iraq, Jamaica, Jordan, Kenya, Lesotho, Madagascar, Mexico, Morocco, Myanmar, Nigeria, Pakistan, Panama, Papua New Guinea, Paraguay, the Philippines, South Africa, Sudan, Suriname, Syria, Tajikistan, Thailand, Togo, Tunisia, Turkmenistan, Uzbekistan, Vietnam	Angola, Belize, Bhutan, Central African Republic, Chad, Republic of Congo, Equatorial Guinea, Eritrea, Eswatini, Guinea, Guyana, Haiti, Jamaica, Lesotho, Liberia, Mauritania, Morocco, Namibia, São Tomé and Príncipe, Sierra Leone, Somalia, South Sudan, Suriname, Timor-Leste, Togo

5.5. Comparison of EBF with respect to other key indicators

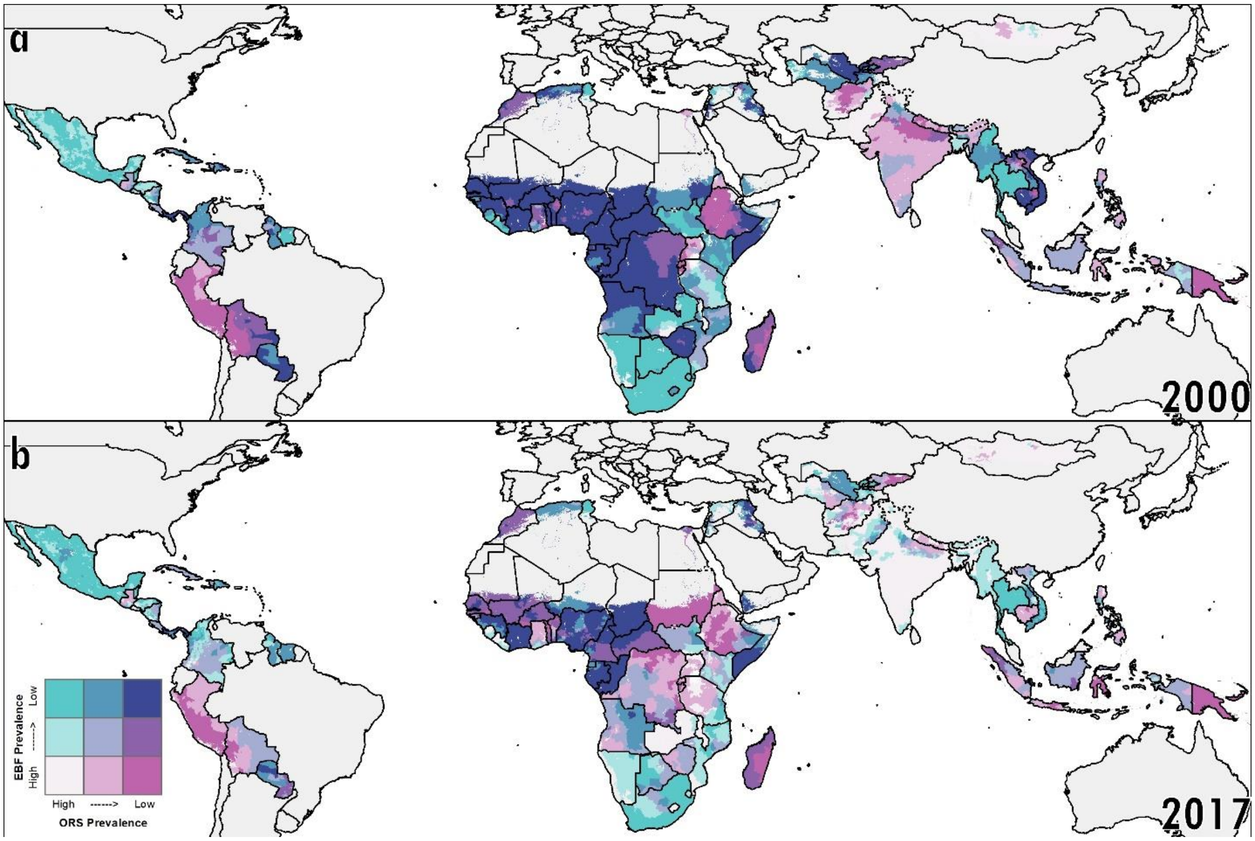

Supplementary Figure 19. Comparison of ORS (oral rehydration solution) prevalence among children under 5 years and EBF prevalence by area

Overlapping population-weighted tertiles of ORS prevalence (in children under 5 years)[45] and EBF prevalence (in children under 6 months) in 2000 and 2017. Cut-offs for the tertiles were 32.5% and 48.2% for the EBF prevalence axis, and 31.7% and 48.3% for the ORS prevalence axis. Maps reflect administrative boundaries, land cover, lakes, and population; grey-colored grid cells had fewer than ten people per 1 × 1-km grid cell and were classified as "barren or sparsely vegetated", or were not included in this analysis[26–31].

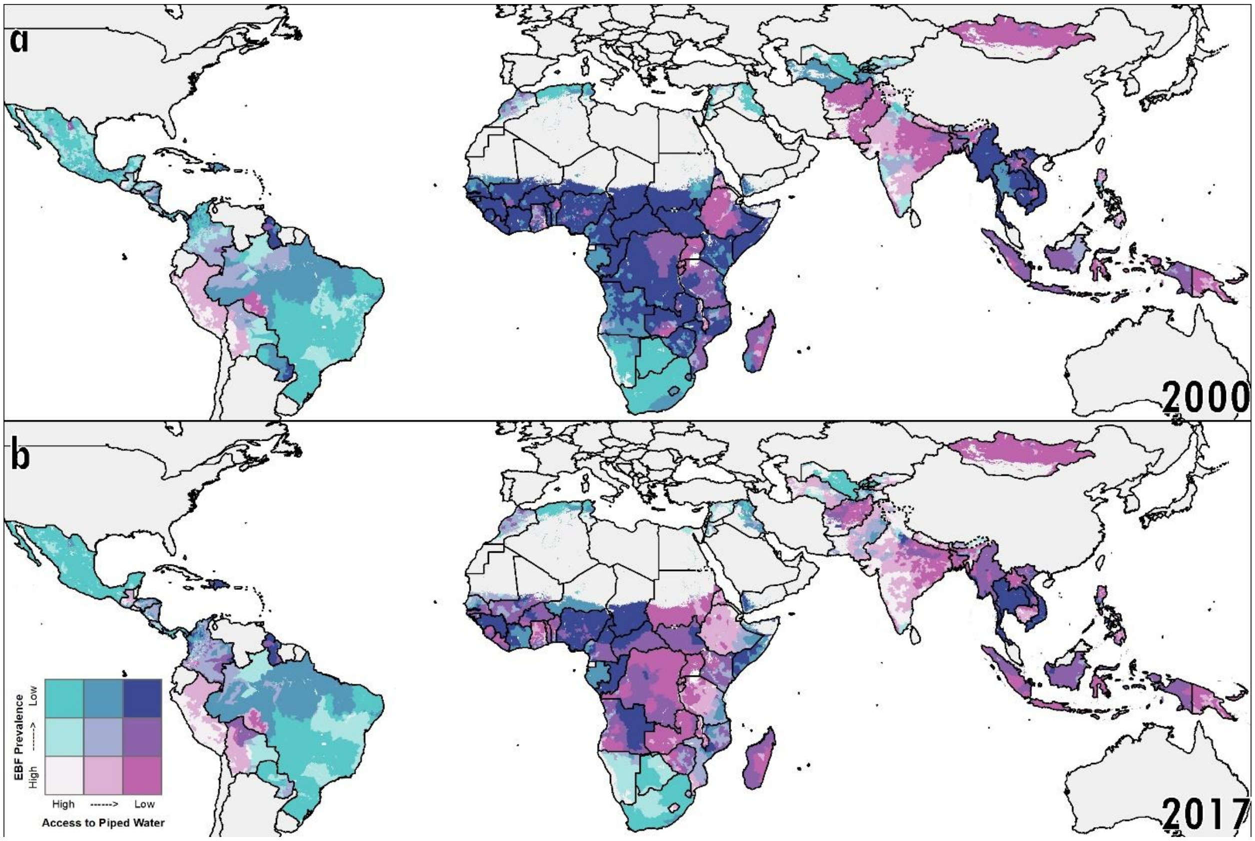

Supplementary Figure 20. Comparison of access to piped water and EBF prevalence by area
Overlapping population-weighted tertiles of access to piped (improved) waster[46] and EBF prevalence (in children under 6 months) in 2000 and 2017. Cut-offs for the tertiles were 32.5% and 48.2% for the EBF prevalence axis, and 31.0% and 64.6% for the access to piped (improved) water axis. Maps reflect administrative boundaries, land cover, lakes, and population; grey-colored grid cells had fewer than ten people per 1 × 1-km grid cell and were classified as "barren or sparsely vegetated", or were not included in this analysis[26–31].

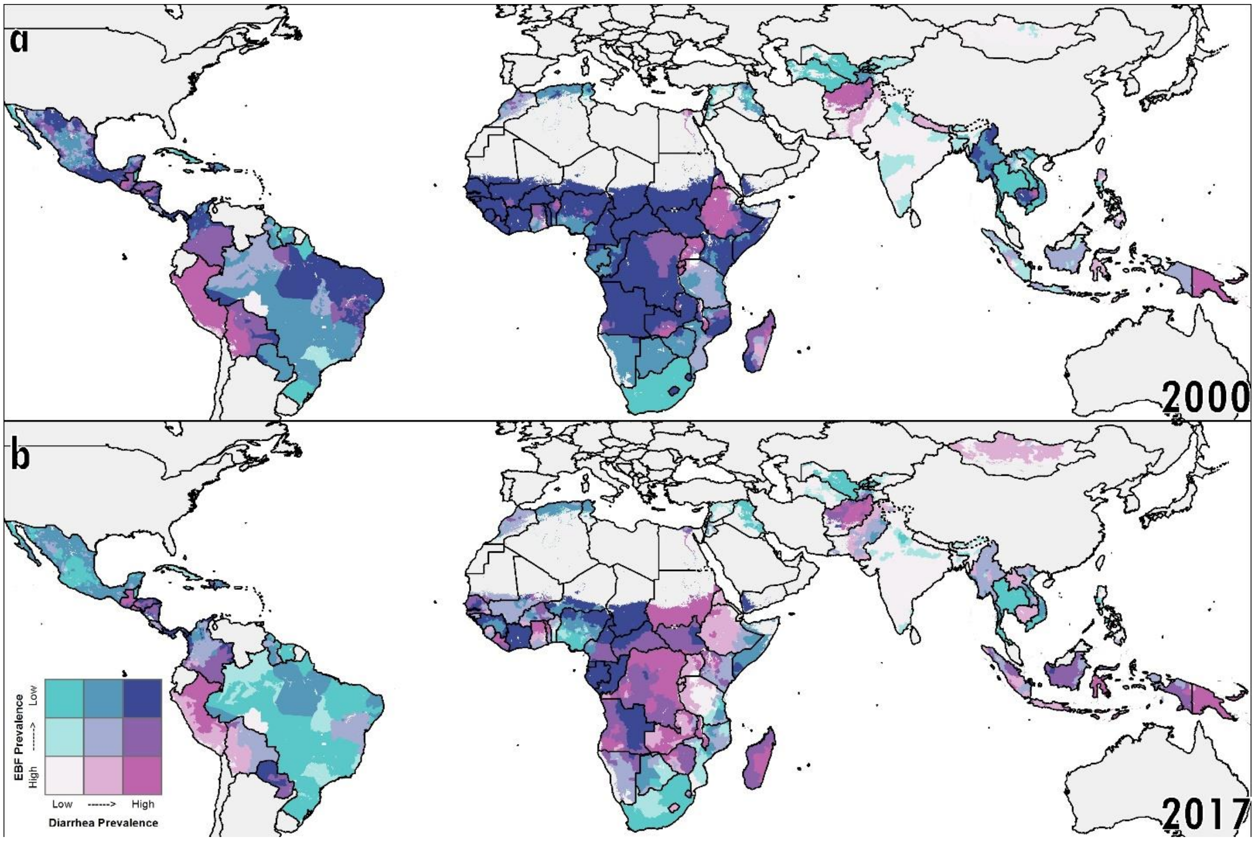

Supplementary Figure 21. Comparison of diarrhea prevalence among children under 5 years and EBF prevalence by area

Overlapping population-weighted tertiles of diarrhea prevalence (in children under 5 years)[38] and EBF prevalence (in children under 6 months) in 2000 and 2017. Cut-offs for the tertiles were 32.5% and 48.2% for the EBF prevalence axis, and 2.4% and 3.8% for the diarrhea prevalence axis. Maps reflect administrative boundaries, land cover, lakes, and population; grey-colored grid cells had fewer than ten people per 1 × 1-km grid cell and were classified as "barren or sparsely vegetated", or were not included in this analysis[26–31].

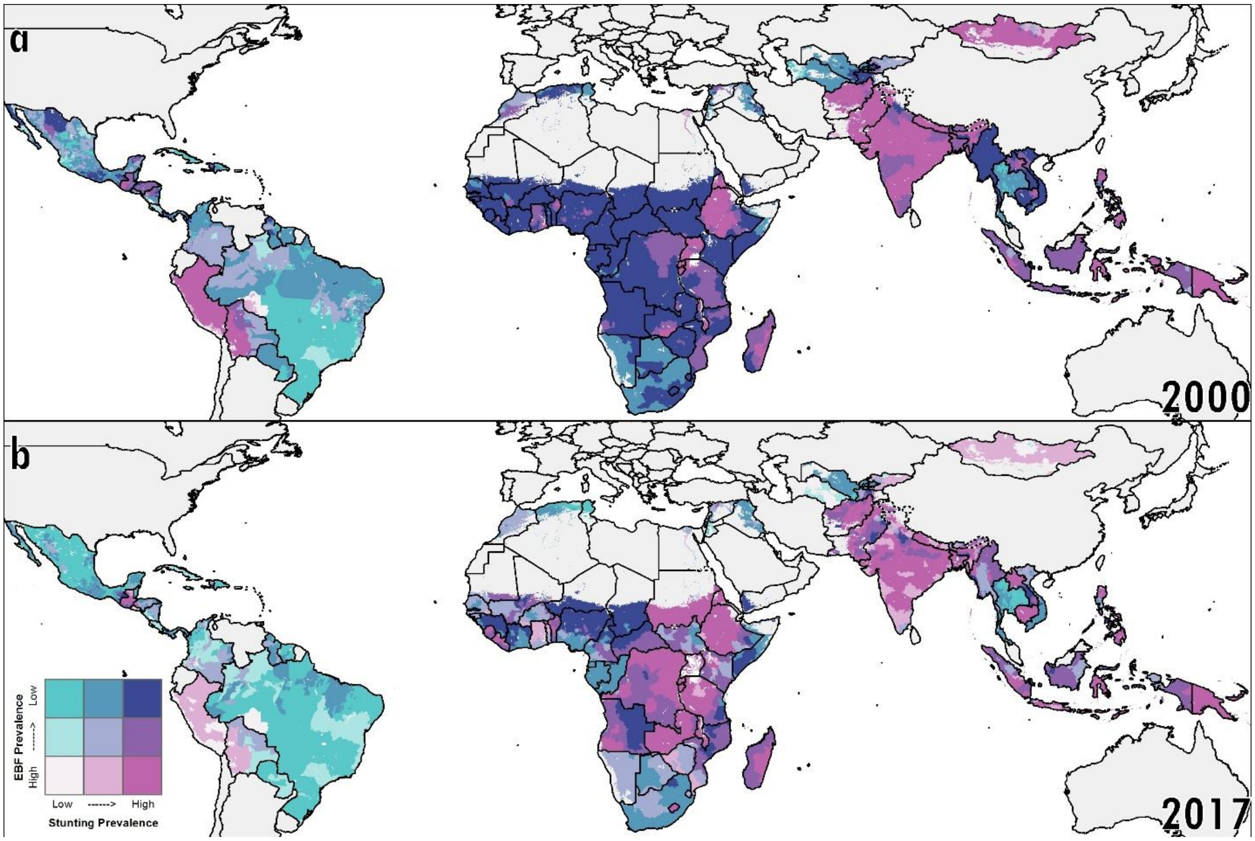

Supplementary Figure 22. Comparison of stunting prevalence among children under 5 years and EBF prevalence by area

Overlapping population-weighted tertiles of stunting prevalence (in children under 5 years)[34] and EBF prevalence (in children under 6 months) in 2000 and 2017. Cut-offs for the tertiles were 32.5% and 48.2% for the EBF prevalence axis, and 15.4% and 33.1% for the stunting prevalence axis. Maps reflect administrative boundaries, land cover, lakes, and population; grey-colored grid cells had fewer than ten people per 1 × 1-km grid cell and were classified as "barren or sparsely vegetated", or were not included in this analysis[26–31].

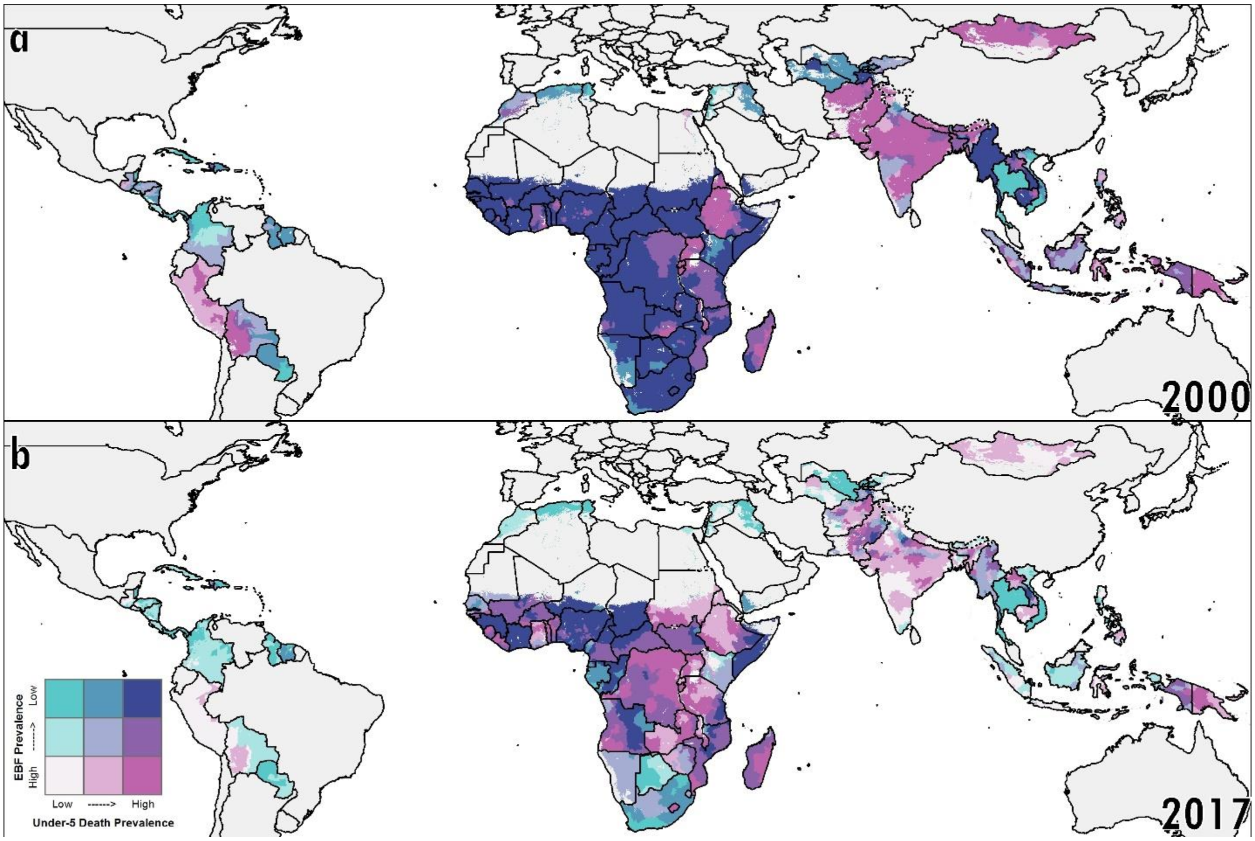

Supplementary Figure 23. Comparison of mortality rate of children under 5 years (U5MR) and EBF prevalence by area

Overlapping population-weighted tertiles of U5MR[37] and EBF prevalence (in children under 6 months) in 2000 and 2017. Cut-offs for the tertiles were 32.5% and 48.2% for the EBF prevalence axis, and 3.2% and 6.1% for the U5MR axis. Maps reflect administrative boundaries, land cover, lakes, and population; grey-colored grid cells had fewer than ten people per 1 × 1-km grid cell and were classified as “barren or sparsely vegetated”, or were not included in this analysis[26–31].

Supplementary Table 12. First administrative-level units with the lowest decile of EBF prevalence, as well as either the lowest decile of oral rehydration solution (ORS) coverage, highest prevalence of child diarrheal disease, highest decile of child stunting prevalence, or highest under-5 mortality rates, for year 2017.

		Low ORS	High Diarrheal Disease	High Stunting	High Under-5 Mortality	Low access to piped water
**Region**	**Country**	**Admin 1 Units**	**Admin 1 Units**	**Admin 1 Units**	**Admin 1 Units**	**Admin 1 Units**
**WSSA**	**Chad**	Mayo-Kebbi Ouest, Mayo-Kebbi Est, Logone Occidental, Mandoul, Tandjilé, Logone Oriental, Hadjer-Lamis, Salamat, Chari-Baguirmi, Batha, Moyen-Chari, Lac, Barh el Ghazel, Guéra, Borkou, Ennedi Ouest, Kanem, Ennedi Est, Tibesti, Ouaddaï, Sila, Wadi Fira, Ville de N'Djamena	Mayo-Kebbi Ouest, Mayo-Kebbi Est, Logone Occidental, Mandoul, Tandjilé, Logone Oriental, Hadjer-Lamis, Salamat, Chari-Baguirmi, Batha, Moyen-Chari, Lac, Barh el Ghazel, Guéra, Borkou, Ennedi Ouest, Kanem, Tibesti, Ouaddaï, Sila, Wadi Fira, Ville de N'Djamena	Mayo-Kebbi Ouest, Hadjer-Lamis, Batha, Lac, Barh el Ghazel, Borkou, Ennedi Ouest, Kanem, Ennedi Est, Tibesti, Wadi Fira	Mayo-Kebbi Ouest, Mayo-Kebbi Est, Logone Occidental, Mandoul, Tandjilé, Logone Oriental, Hadjer-Lamis, Salamat, Chari-Baguirmi, Batha, Moyen-Chari, Lac, Barh el Ghazel, Guéra, Borkou, Ennedi Ouest, Kanem, Tibesti, Ouaddaï, Sila, Ville de N'Djamena	
	**Nigeria**	Yobe, Bauchi, Kebbi		Yobe, Bauchi, Kebbi, Katsina, Jigawa, Kano	Yobe, Bauchi, Kebbi, Katsina, Jigawa, Kano, Niger	

	**Niger**			Zinder,<br>Diffa	Zinder,<br><br>Dosso	
**ESSA**	**Yemen**		Shabwah,<br>Abyan,<br>`Adan,<br>Ma'rib,<br>Lahij,<br>Ta`izz,<br>Al Bayda',<br>Al Dali',<br>Al Hudaydah,<br>Ibb	Al Dali',<br><br>Ibb		
	**Somalia**				Nugaal,<br>Sool,<br>Galguduud,<br>Mudug,<br>Shabeellaha Dhexe,<br>Bari,<br>Sanaag,<br>Shabeellaha Hoose,<br>Togdheer	
	**Comoros**					Mwali
**CSSA**	**Gabon**		Woleu-Ntem			
**SEAS**	**Thailand**					Sakon Nakhon,<br>Roi Et,<br>Bangkok Metropolis,<br>Maha Sarakham,<br>Songkhla,<br>Mwali,<br>Udon Thani,<br>Nakhon Si Thammarat,<br>Surat Thani,<br>Khon Kaen

WSSA=Western sub-Saharan Africa, ESSA=Eastern sub-Saharan Africa, CSSA=Central sub-Saharan Africa, SEAS=Southeast Asia

6.0. Limitations

Data Availability
This work should be assessed in full acknowledgement of the data and methodological limitations. Most importantly, the accuracy of our estimates is critically dependent on the quantity and quality of the underlying data. Availability of relevant data varies both spatially and temporally across LMICs (Supplementary Figures 1–5). For example, temporal data gaps are observed in South Sudan (for the 2000–2002 period) and in Namibia (for the 2008–2012 period), whereas spatial data gaps are seen in Botswana (for the 2003–2007 period) and in South Africa (for the 2013–2018 period). We have constructed a large database of geo-located EBF prevalence data for the purposes of this analysis; nonetheless, important gaps in data coverage, both spatial and temporal, remain (Supplementary Figures 1–5), and these gaps are main sources of uncertainty around our estimates (as seen in Extended Data Figure 3).

More local data are necessary to monitor health outcomes and guide quality improvement efforts and increase certainty of our results. Collecting local data from all communities every year would be an insurmountable task for most countries; this study aids in filling the current knowledge gap by producing estimates for areas without data collection based on learned patterns from well-surveyed areas, using the same estimation methods for all areas for comparable results across communities.

Data Accuracy
In addition, there are several factors related to data quality that should be acknowledged. Data in our analyses were obtained from caregivers of infants at any time point between birth and 6 months of age. Though an infant's EBF status was based on a single time point (the 24 hours preceding the survey interview), which is known to over-estimate EBF practice for the full six-month period as infants may be fed other foods and liquids either before or after the survey, this estimation is standard practice[47–51]. Following the standard approach for estimating EBF based on international guidelines, the proportion of infants who are exclusively breastfed for the full six months is calculated by estimating prevalence of EBF for all children under 6 months of age (though EBF is known to decline with age). Due to the age range (0–5 months-old infants) relevant to the purpose of estimating EBF prevalence, our sample sizes are relatively smaller than previous efforts mapping localised estimates for health conditions, outcomes, and socioeconomic indicators[34,36–38], further contributing to the relatively large degree of uncertainty associated with our estimates.

The location information associated with the data compiled for these analyses is subject to some error. In order to protect respondents' confidentiality, most surveys that collect GPS coordinates perform some type of random displacement on those coordinates prior to releasing data for secondary analyses. For example, GPS coordinates for Demographic and Health Surveys (DHS) are displaced by up to 2 km for urban clusters, up to 5 km for most rural clusters, and up to 10 km in a random 1% of rural clusters[52]. Furthermore, data associated with polygons rather than GPS coordinates were resampled so that they could be included in the geostatistical model, but this process essentially assumes that EBF prevalence is constant over the polygon. Research on scalable methods for better integration of polygon data in geostatistical models similar to those used in this analysis is currently ongoing.

Modelling Limitations
With respect to the modelling strategy, the primary limitation is the difficulty in assessing model performance at the grid-cell level. We used cross-validation to assess model performance but due to the substantial impact of sampling error on estimates derived from single survey clusters, it was necessary to aggregate both the data and predictions when assessing error. Additionally, while we have attempted to propagate uncertainty from various sources through the different modelling stages, there are some sources of uncertainty that have not been propagated. In particular, it was not computationally feasible to propagate uncertainty from the sub-models in stacking through the geostatistical model. Similarly, although the WorldPop population raster is also composed of estimates associated with some uncertainty, this uncertainty is difficult to quantify and not currently reported, and so we were unable to propagate this uncertainty into our estimates of EBF prevalence for administrative units that were created using population-weighted averages of grid-cell estimates.

Model fitting was carried out using an integrated nested Laplace approximation to the posterior distribution, as implemented in the R-INLA package[42]. Prediction from fitted models was subsequently carried out using the inla.posterior.sample() function, which generates samples from the approximated posterior of the fitted model. Both model fitting and prediction thus require approximations, and these approximations may introduce error. While it is difficult to assess the impact of these approximations in this particular use case, our validation analysis found that our final model has low bias and good coverage of the 95% prediction intervals which provides some reassurance that the approximation method used—as well as other potential sources of error—are not resulting in appreciable bias or poorly described uncertainty in our reported estimates.

Furthermore, our projection methods are derived from the previous spatiotemporal historical trends and based on the assumption that recent trends will continue; thus, we are not projecting underlying drivers (such as increasing urbanisation or changes in population)[7,53,54].

7.0. Supplementary Discussion

Additional barriers to EBF include cultural perceptions and generational feeding practices, which can be highly variable across communities. Mothers who perceive their breast milk to be insufficient or nutritionally inadequate are more likely to discontinue practice of EBF[55]. Infant cues when feeding (such as fussiness and crying) and problems when breastfeeding (such as breast pain or engorgement, or problems latching) are commonly cited barriers to EBF[55]. A common misconception and practice is the discarding of mothers' early breast milk (colostrum), which has important protective properties for infants, as it is perceived to be sour and difficult to digest[56–58]. This instead is replaced by prelacteal feeding of water, formula, or animal milk, and makes establishing breastfeeding difficult[55,57,58]. Some cultural practices involve feeding newborns water, sugar water, tea, honey, butter, animal milk, or porridges before they are fed at the breast, or during their first few months of life[56,57]. Breastfeeding counselling to increase maternal knowledge on the importance of EBF and provide lactation support can help counteract these barriers[56,55]. Fathers and grandparents can influence a woman's decision to breastfeed[56,57,59], whereas positive encouragement from family and sharing of household responsibilities increases the likelihood mothers will continue breastfeeding for the newborn's first six months[55,56].

The subnational maps in this study highlight where further efforts are needed to reduce barriers to breastfeeding so more infants can receive the health benefits of EBF. Furthermore, when combined with maps of other health conditions and interventions, these estimates provide policy makers with quantitative tools for evaluating subnational health disparities and needs and identifying sub-populations that could benefit most from targeted investments. Lessons learned from countries that have made progress towards the WHO GNT could also be adapted and applied in other contexts, where appropriate. The WHO-UNICEF Global Breastfeeding Collective (GBC) provides government leaders with key policy actions to provide a supportive environment to encourage breastfeeding. Further local investigations of the underlying drivers of these subnational inequalities, including on local customs and perceptions of breastfeeding, is important for planning and implementing effective strategies and behaviour change interventions to increase EBF practice.

8.0. Collaborators and Affiliations

LBD EBF Stage II Collaborators

Natalia V Bhattacharjee,[1] Lauren E Schaeffer,[2,3] Dan Lu,[1] Megan F Schipp,[1] Alice Lazzar-Atwood,[1] Katie M Donkers,[1] Gdiom Gebreheat Abady,[4] Prof. Foad Abd-Allah,[5] Prof. Ahmed Abdelalim,[5] Zeleke Hailemariam Abebo,[6] Ayenew Negesse Abejie,[7] Akine Eshete Abosetugn,[8] Prof. Lucas Guimarães Abreu,[9] Michael R M Abrigo,[10] Eman Abu-Gharbieh,[11] Abdelrahman I Abushouk,[12,13] Aishatu L Adamu,[14,15] Isaac Akinkunmi Adedeji,[16] Adeyinka Emmanuel Adegbosin,[17] Victor Adekanmbi,[18] Olatunji O Adetokunboh,[19,20] Prof. Marcela Agudelo-Botero,[21] Budi Aji,[22] Oluwaseun Oladapo Akinyemi,[23,24] Alehegn Aderaw Alamneh,[25] Fahad Mashhour Alanezi,[26] Turki M Alanzi,[27] James Albright,[1] Jacqueline Elizabeth Alcalde-Rabanal,[28] Biresaw Wassihun Alemu,[29,30] Robert Kaba Alhassan,[31] Beriwan Abdulqadir Ali,[32,33] Saqib Ali,[34] Cyrus Alinia,[35] Vahid Alipour,[36,37] Arianna Maever L Amit,[38,39] Dickson A Amugsi,[40] Etsay Woldu Anbesu,[41] Prof. Robert Ancuceanu,[42] Mina Anjomshoa,[43] Fereshteh Ansari,[44,45] Carl Abelardo T Antonio,[46,47] Davood Anvari,[48,49] Jalal Arabloo,[36] Amit Arora,[50,51] Kurnia Dwi Artanti,[52] Mulusew A Asemahagn,[53] Wondwossen Niguse Asmare,[54] Maha Moh'd Wahbi Atout,[55] Prof. Marcel Ausloos,[56,57] Nefsu Awoke,[58] Beatriz Paulina Ayala Quintanilla,[59] Martin Amogre Ayanore,[60] Yared Asmare Aynalem,[61] Muluken Altaye Ayza,[62] Zelalem Nigussie Azene,[63] Darshan B B,[64] Ashish D Badiye,[65] Atif Amin Baig,[66] Shankar M Bakkannavar,[67] Prof. Maciej Banach,[68,69] Palash Chandra Banik,[70] Prof. Till Winfried Bärnighausen,[71,72] Prof. Huda Basaleem,[73] Mohsen Bayati,[74] Bayisa Abdissa Baye,[75] Prof. Neeraj Bedi,[76,77] Sefealem Assefa Belay,[78] Akshaya Srikanth Bhagavathula,[79,80] Dinesh Bhandari,[81,82] Prof. Nikha Bhardwaj,[83] Pankaj Bhardwaj,[84,85] Prof. Zulfiqar A Bhutta,[86,87] Ali Bijani,[88] Tsegaye Adane Birhan,[89] Binyam Minuye Birihane,[90,91] Zebenay Workneh Bitew,[92,93] Somayeh Bohlouli,[94] Mahdi Bohluli,[95,96] Hunduma Amensisa Bojia,[97] Archith Boloor,[98] Oliver J Brady,[99] Nicola Luigi Bragazzi,[100] Prof. Andre R Brunoni,[101,102] Shyam S Budhathoki,[103] Prof. Sharath Burugina Nagaraja,[104] Zahid A Butt,[105,106] Prof. Rosario Cárdenas,[107] Prof. Joao Mauricio Castaldelli-Maia,[108] Franz Castro,[109] Achille Cernigliaro,[110] Jaykaran Charan,[111] Pranab Chatterjee,[112] Souranshu Chatterjee,[113] Vijay Kumar Chattu,[114,115] Sarika Chaturvedi,[116] Mohiuddin Ahsanul Kabir Chowdhury,[117,118] Dinh-Toi Chu,[119] Michael L Collison,[1] Aubrey J Cook,[1] Michael A Cork,[1] Rosa A S Couto,[120] Baye Dagnew,[121] Haijiang Dai,[122,123] Prof. Lalit Dandona,[124,1,125] Prof. Rakhi Dandona,[124,1,126] Parnaz Daneshpajouhnejad,[127,128] Aso Mohammad Darwesh,[129] Amira Hamed Darwish,[130] Prof. Ahmad Daryani,[131] Jai K Das,[132] Rajat Das Gupta,[118,133] Claudio Alberto Dávila-Cervantes,[134] Prof. Adrian Charles Davis,[135,136] Nicole Davis Weaver,[1] Edgar Denova-Gutiérrez,[137] Kebede Deribe,[138,139] Assefa Desalew,[140] Aniruddha Deshpande,[141] Awrajaw Dessie,[89] Keshab Deuba,[142,143] Prof. Samath Dhamminda Dharmaratne,[144,126,1] Meghnath Dhimal,[145] Govinda Prasad Dhungana,[146] Prof. Daniel Diaz,[147,148] Alireza Didarloo,[149] Isaac Oluwafemi Dipeolu,[150] Linh Phuong Doan,[151] Bereket Duko,[152,153] Prof. Andre Rodrigues Duraes,[154,155] Laura Dwyer-Lindgren,[1,126] Lucas Earl,[1] Prof. Maysaa El Sayed Zaki,[156] Prof. Maha El Tantawi,[157] Teshome Bekele Elema,[158,159] Prof. Hala Rashad Elhabashy,[160] Shaimaa I El-Jaafary,[5] Pawan Sirwan Faris,[161,162] Prof. Andre Faro,[163] Prof. Farshad Farzadfar,[164] Prof. Valery L Feigin,[165,1,166] Berhanu Elfu Feleke,[167] Tomas Y Ferede,[168] Florian Fischer,[169] Nataliya A Foigt,[170] Prof. Morenike Oluwatoyin Folayan,[171] Richard Charles Franklin,[172] Mohamed M Gad,[173,174] Shilpa Gaidhane,[175] William M Gardner,[1] Biniyam Sahiledengle Geberemariyam,[176] Birhan Gebresillassie Gebregiorgis,[61] Ketema Bizuwork Gebremedhin,[177] Berhe Gebremichael,[178] Fariborz Ghaffarpasand,[179] Prof. Syed Amir

Gilani,[180,181] Themba G Ginindza,[182] Mustefa Glagn,[183] Mahaveer Golechha,[184] Dr Kebebe Bekele Gonfa,[185] Prof. Bárbara Niegia Garcia Goulart,[186] Nachiket Gudi,[187] Davide Guido,[188] Rashid Abdi Guled,[189] Prof. Yuming Guo,[190,191] Prof. Samer Hamidi,[192] Demelash Woldeyohannes Handiso,[193] Ahmed I Hasaballah,[194] Amr Hassan,[5] Khezar Hayat,[195,196] Mohamed I Hegazy,[5] Behnam Heidari,[197] Nathaniel J Henry,[198] Prof. Claudiu Herteliu,[57,199] Hagos Degefa de Hidru,[200] Hung Chak Ho,[201] Chi Linh Hoang,[202] Ramesh Holla,[64] Julia Hon,[1] Prof. Mostafa Hosseini,[203,204] Mehdi Hosseinzadeh,[36] Prof. Mowafa Househ,[205] Prof. Mohamed Hsairi,[206] Prof. Guoqing Hu,[207] Tanvir M Huda,[208,117] Prof. Bing-Fang Hwang,[209] Segun Emmanuel Ibitoye,[150] Olayinka Stephen Ilesanmi,[210,211] Irena M Ilic,[212] Prof. Milena D Ilic,[213] Leeberk Raja Inbaraj,[214] Usman Iqbal,[215] Seyed Sina Naghibi Irvani,[216] M Mofizul Islam,[217] Chidozie C D Iwu,[218] Chinwe Juliana Iwu,[219,20] Prof. Animesh Jain,[220] Prof. Manthan Dilipkumar Janodia,[221] Tahereh Javaheri,[222] Yetunde O John-Akinola,[150] Kimberly B Johnson,[1] Farahnaz Joukar,[223,224] Jacek Jerzy Jozwiak,[225] Ali Kabir,[226] Leila R Kalankesh,[227] Rohollah Kalhor,[228,229] Ashwin Kamath,[64,230] Naser Kamyari,[231] Other Tanuj Kanchan,[232] Neeti Kapoor,[65] Prof. Behzad Karami Matin,[233] Salah Eddin Karimi,[234] Habtamu Kebebe Kasaye,[235] Getinet Kassahun,[236] Nicholas J Kassebaum,[237,1,126] Gbenga A Kayode,[238,239] Ali Kazemi Karyani,[233] Prof. Peter Njenga Keiyoro,[240] Bayew Kelkay,[241] Nauman Khalid,[242] Md Nuruzzaman Khan,[243] Khaled Khatab,[244,245] Amir M Khater,[246] Mona M Khater,[247] Prof. Mahalaqua Nazli Khatib,[248] Yun Jin Kim,[249] Ruth W Kimokoti,[250] Damaris K Kinyoki,[1,126] Prof. Adnan Kisa,[251,252] Sezer Kisa,[253] Soewarta Kosen,[254] Kewal Krishan,[255] Vaman Kulkarni,[220] G Anil Kumar,[124] Manasi Kumar,[256,257] Nithin Kumar,[220] Pushpendra Kumar,[258] Om P Kurmi,[259,260] Dian Kusuma,[261,262] Prof. Carlo La Vecchia,[263] Sheetal D Lad,[264] Faris Hasan Lami,[265] Prof. Iván Landires,[266,267] Prof. Van Charles Lansingh,[268,269] Savita Lasrado,[270] Paul H Lee,[271] Kate E LeGrand,[1] Ian D Letourneau,[1] Sonia Lewycka,[272,273] Bingyu Li,[274] Ming-Chieh Li,[275] Shanshan Li,[276] Xuefeng Liu,[277] Prof. Rakesh Lodha,[278] Jaifred Christian F Lopez,[279,280] Celia Louie,[1] Daiane Borges Machado,[281,282] Prof. Venkatesh Maled,[283,284] Shokofeh Maleki,[285] Prof. Deborah Carvalho Malta,[286] Abdullah A Mamun,[287] Navid Manafi,[288,289] Mohammad Ali Mansournia,[203] Chabila Christopher Mapoma,[290] Laurie B Marczak,[1] Francisco Rogerlândio Martins-Melo,[291] Prof. Man Mohan Mehndiratta,[292,293] Fabiola Mejia-Rodriguez,[294] Tefera Chane Mekonnen,[295] Walter Mendoza,[296] Prof. Ritesh G Menezes,[297] Endalkachew Worku Mengesha,[298] Abera M Mersha,[299] Ted R Miller,[300,153] Prof. GK Mini,[301,302] Prof. Erkin M Mirrakhimov,[303,304] Prof. Sanjeev Misra,[305] Masoud Moghadaszadeh,[306,307] Dara K Mohammad,[308,309] Abdollah Mohammadian-Hafshejani,[310] Jemal Abdu Mohammed,[311] Shafiu Mohammed,[312,71] Prof. Ali H Mokdad,[1,126] Pablo A Montero-Zamora,[313,314] Masoud Moradi,[233] Rahmatollah Moradzadeh,[315] Paula Moraga,[316] Jonathan F Mosser,[1] Seyyed Meysam Mousavi,[317] Prof. Amin Mousavi Khaneghah,[318] Sandra B Munro,[1] Moses K Muriithi,[319] Prof. Ghulam Mustafa,[320,321] Saravanan Muthupandian,[322] Ahamarshan Jayaraman Nagarajan,[323,324] Gurudatta Naik,[325] Mukhammad David Naimzada,[326,327] Vinay Nangia,[328] Prof. Bruno Ramos Nascimento,[329,330] Prof. Vinod C Nayak,[67] Rawlance Ndejjo,[331] Duduzile Edith Ndwandwe,[332] Ionut Negoi,[333,334] Georges Nguefack-Tsague,[335] Josephine W Ngunjiri,[336] Cuong Tat Nguyen,[337] Diep Ngoc Nguyen,[151,338] Huong Lan Thi Nguyen,[337] Samuel Negash Nigussie,[339] Tadesse T N Nigussie,[339] Rajan Nikbakhsh,[340] Chukwudi A Nnaji,[219,341] Virginia Nunez-Samudio,[342,343] Prof. Bogdan Oancea,[344] Onome Bright Oghenetega,[345] Andrew T Olagunju,[346,347] Bolajoko Olubukunola Olusanya,[348] Jacob Olusegun Olusanya,[348] Muktar Omer Omer,[349] Prof. Obinna E Onwujekwe,[350] Doris V Ortega-Altamirano,[351] Aaron E Osgood-Zimmerman,[1] Nikita Otstavnov,[326] Stanislav S Otstavnov,[326,352] Prof. Mayowa O Owolabi,[353,354] Prof. Mahesh P A,[355] Jagadish Rao Padubidri,[64]

Adrian Pana,[57,356] Anamika Pandey,[357] Seithikurippu R Pandi-Perumal,[358] Helena Ullyartha Pangaribuan,[359] Shradha S Parsekar,[360] Deepak Kumar Pasupula,[361] Urvish K Patel,[362] Prof. Ashish Pathak,[363,364] Mona Pathak,[365] Sanjay M Pattanshetty,[187] Prof. George C Patton,[366,367] Kebreab Paulos,[368] Veincent Christian Filipino Pepito,[369] Brandon V Pickering,[1] Marina Pinheiro,[370] Ellen G Piwoz,[371] Khem Narayan Pokhrel,[372] Hadi Pourjafar,[373,374] Sergio I Prada,[375,376] Dimas Ria Angga Pribadi,[377] Prof. Zahiruddin Quazi Syed,[378] Prof. Mohammad Rabiee,[379] Navid Rabiee,[380] Fakher Rahim,[381,382] Shadi Rahimzadeh,[383,164] Azizur Rahman,[384] Mohammad Hifz Ur Rahman,[385] Amir Masoud Rahmani,[386,387] Rajesh Kumar Rai,[388,389] Chhabi Lal Ranabhat,[390,391] Sowmya J Rao,[392] Prof. Prateek Rastogi,[393] Priya Rathi,[64] David Laith Rawaf,[394,395] Prof. Salman Rawaf,[396,397] Reza Rawassizadeh,[398] Rahul Rawat,[399] Ramu Rawat,[400] Lemma Demissie Regassa,[178] Prof. Maria Albertina Santiago Rego,[401] Robert C Reiner Jr.,[1,126] Bhageerathy Reshmi,[402,403] Aziz Rezapour,[36] Ana Isabel Ribeiro,[404] Jennifer Rickard,[405,406] Leonardo Roever,[407] Susan Fred Rumisha,[408,409] Godfrey M Rwegerera,[410] Prof. Rajesh Sagar,[411] Prof. S. Mohammad Sajadi,[412,413] Marwa Rashad Salem,[414] Abdallah M Samy,[415] Prof. Milena M Santric-Milicevic,[212,416] Sivan Yegnanarayana Iyer Saraswathy,[417,418] Abdur Razzaque Sarker,[419] Benn Sartorius,[420,421,126] Brijesh Sathian,[422,423] Prof. Deepak Saxena,[424,378] Alyssa N Sbarra,[1] Debarka Sengupta,[425] Subramanian Senthilkumaran,[426] Feng Sha,[427] Omid Shafaat,[428,429] Amira A Shaheen,[430] Masood Ali Shaikh,[431] Prof. Ali S Shalash,[432] Mohammed Shannawaz,[433] Prof. Aziz Sheikh,[434,435] Prof. B Suresh Kumar Shetty,[393] Ranjitha S Shetty,[436] Prof. Kenji Shibuya,[437] Wondimeneh Shibabaw Shiferaw,[61] Prof. Jae Il Shin,[438] Prof. Diego Augusto Santos Silva,[439] Prof. Narinder Pal Singh,[440] Pushpendra Singh,[441] Surya Singh,[442] Yitagesu Sintayehu,[443] Valentin Yurievich Skryabin,[444] Anna Aleksandrovna Skryabina,[445] Amin Soheili,[446] Shahin Soltani,[233] Muluken Bekele Sorrie,[183] Emma Elizabeth Spurlock,[1] Krista M Steuben,[1] Agus Sudaryanto,[447] Mu'awiyyah Babale Sufiyan,[448] Scott J Swartz,[449,450] Eyayou Girma Tadesse,[451] Animut Tagele Tamiru,[241] Leili Tapak,[231,452] Prof. Md Ismail Tareque,[453] Ingan Ukur Tarigan,[454] Getayeneh Antehunegn Tesema,[455] Fisaha Haile Tesfay,[456,457] Abinet Teshome,[451] Zemenu Tadesse Tessema,[458] Prof. Kavumpurathu Raman Thankappan,[459] Rekha Thapar,[220] Prof. Nihal Thomas,[460] Roman Topor-Madry,[461,462] Marcos Roberto Tovani-Palone,[463,464] Eugenio Traini,[465] Bach Xuan Tran,[466] Phuong N Truong,[467] Berhan Tsegaye BT Tsegaye,[236] Irfan Ullah,[468] Chukwuma David Umeokonkwo,[469] Prof. Bhaskaran Unnikrishnan,[470] Era Upadhyay,[471] Prof. Benjamin S Chudi Uzochukwu,[472] John David VanderHeide,[473] Prof. Francesco S Violante,[474,475] Bay Vo,[476] Yohannes Dibaba Wado,[477] Prof. Yasir Waheed,[478] Richard G Wamai,[479,480] Fang Wang,[481] Yafeng Wang,[482] Yuan-Pang Wang,[102] Nuwan Darshana Wickramasinghe,[483] Kirsten E Wiens,[484] Prof. Charles Shey Wiysonge,[219,341] Lauren Woyczynski,[1] Prof. Ai-Min Wu,[485] Chenkai Wu,[486,487] Tomohide Yamada,[488] Prof. Sanni Yaya,[489,490] Alex Yeshaneh,[491] Yigizie Yeshaw,[458] Yordanos Gizachew Yeshitila,[299] Mekdes Tigistu Yilma,[492] Prof. Paul Yip,[493,494] Naohiro Yonemoto,[495,496] Tewodros Yosef,[339] Prof. Mustafa Z Younis,[497,498] Abdilahi Yousuf Yousuf,[349] Prof. Chuanhua Yu,[482] Yong Yu,[499] Deniz Yuce,[500] Prof. Shamsa Zafar,[501,502] Syed Saoud Zaidi,[503] Leila Zaki,[504] Prof. Josefina Zakzuk,[505] Maryam Zamanian,[315] Prof. Heather J Zar,[506,507] Prof. Mikhail Sergeevich Zastrozhin,[508,509] Anasthasia Zastrozhina,[510] Desalege Amare Zelellw,[511] Yunquan Zhang,[512,513] Zhi-Jiang Zhang,[514] Xiu-Ju George Zhao,[515,481] Prof. Sanjay Zodpey,[516] Yves Miel H Zuniga,[517,518] and Prof. Simon I Hay.[1,126]

Affiliations

[1]Institute for Health Metrics and Evaluation, University of Washington, Seattle, WA, USA; [2]Medical Teams International, Seattle, WA, USA; [3]Department of Pediatric Newborn Medicine,

Brigham and Women's Hospital, Boston, MA, USA; [4]Department of Nursing, Adigrat University, Adigrat, Ethiopia; [5]Department of Neurology, Cairo University, Cairo, Egypt; [6]Department of Public health, Arba Minch University, Arba Minch, Ethiopia; [7]Debre Markos University, Debre Markos, Ethiopia; [8]Department of Public Health, Debre Berhan University, Debre Brehan, Ethiopia; [9]Department of Pediatric Dentistry, Federal University of Minas Gerais, Belo Horizonte, Brazil; [10]Department of Research, Philippine Institute for Development Studies, Quezon City, Philippines; [11]Department of Clinical Sciences, University of Sharjah, Sharjah, United Arab Emirates; [12]Harvard Medical School, Harvard University, Boston, MA, USA; [13]Department of Medicine, Ain Shams University, Cairo, Egypt; [14]Community Medicine Department, Bayero University Kano, Kano, Nigeria; [15]Infectious Diseases Epidemiology, London School of Hygiene & Tropical Medicine, London, UK; [16]Department of Sociology, Olabisi Onabanjo University, Ago-Iwoye, Nigeria; [17]School of Medicine, Griffith University, Gold coast, QLD, Australia; [18]Population Health Sciences, King's College London, London, England; [19]Centre of Excellence for Epidemiological Modelling and Analysis, Stellenbosch University, Stellenbosch, South Africa; [20]Department of Global Health, Stellenbosch University, Cape Town, South Africa; [21]Center for Policy, Population & Health Research, National Autonomous University of Mexico, Mexico City, Mexico; [22]Faculty of Medicine and Public Health, Jenderal Soedirman University, Purwokerto, Indonesia; [23]Department of Health Policy and Management, University of Ibadan, Ibadan, Nigeria; [24]Department of Health Policy and Management, University College Hospital, Ibadan, Ibadan, Nigeria; [25]Department of Human Nutrition and Food Sciences, Debre Markos University, Debre Markos, Ethiopia; [26]Imam Abdulrahman Bin Faisal University, Dammam, Saudi Arabia; [27]Health Information Management and Technology Department, Imam Abdulrahman Bin Faisal University, Dammam, Saudi Arabia; [28]Center for Health System Research, National Institute of Public Health, Cuernavaca, Mexico; [29]College of Medicine and Health Science, Arba Minch University, Arba Minch, Ethiopia; [30]Department of Midwifery, Arba Minch University, Injbara, Ethiopia; [31]Institute of Health Research, University of Health and Allied Sciences, Ho, Ghana; [32]Erbil Technical Health College, Erbil Polytechnic University, Erbil, Iraq; [33]School of Pharmacy, Tishk International University, Erbil, Iraq; [34]Department of Information Systems, College of Economics and Political Science, Sultan Qaboos University, Muscat, Oman; [35]Department of Health Care Management and Economics, Urmia University of Medical Science, Urmia, Iran; [36]Health Management and Economics Research Center, Iran University of Medical Sciences, Tehran, Iran; [37]Health Economics Department, Iran University of Medical Sciences, Tehran, Iran; [38]School of Medicine and Public Health, Ateneo De Manila University, Manila, Philippines; [39]College of Medicine, University of the Philippines Manila, Manila, Philippines; [40]Maternal and Child Wellbeing, African Population and Health Research Center, Nairobi, Kenya; [41]Department of Public Health, Samara University, Samara, Ethiopia; [42]Pharmacy Department, Carol Davila University of Medicine and Pharmacy, Bucharest, Romania; [43]Social Determinants of Health Research Center, Rafsanjan University of Medical Sciences, Rafsanjan, Iran; [44]Research Center for Evidence Based Medicine, Tabriz University of Medical Sciences, Tabriz, Iran; [45]Razi Vaccine and Serum Research Institute, Agricultural Research, Education, and Extension Organization (AREEO), Tehran, Iran; [46]Department of Health Policy and Administration, University of the Philippines Manila, Manila, Philippines; [47]Department of Applied Social Sciences, Hong Kong Polytechnic University, Hong Kong, China; [48]Department of Parasitology, Mazandaran University of Medical Sciences, Sari, Iran; [49]Department of Parasitology, Iranshahr University of Medical Sciences, Iranshahr, Iran; [50]School of Health Sciences, Western Sydney

University, Campbelltown, NSW, Australia; [51]Disciple of Child and Adolescent Health, University of Sydney, Westmead, NSW, Australia; [52]Department of Epidemiology, Airlangga University, Surabaya, Indonesia; [53]School of Public Health, Bahir Dar University, Bahir Dar, Ethiopia; [54]Department of Nursing, Mizan-Tepi University, Mizan Teferi, Ethiopia; [55]Faculty of Nursing, Philadelphia University, Amman, Jordan; [56]School of Business, University of Leicester, Leicester, UK; [57]Department of Statistics and Econometrics, Bucharest University of Economic Studies, Bucharest, Romania; [58]Department of Nursing, Wolaita Sodo University, Wolaita Sodo, Ethiopia; [59]The Judith Lumley Centre, La Trobe University, Melbourne, VIC, Australia; [60]Department of Health Policy Planning and Management, University of Health and Allied Sciences, Ho, Ghana; [61]Department of Nursing, Debre Berhan University, Debre Berhan, Ethiopia; [62]Department of Pharmacology and Toxicology, Mekelle University, Mekelle, Ethiopia; [63]Department of Reproductive Health, University of Gondar, Gondar, Ethiopia; [64]Kasturba Medical College, Mangalore, Manipal Academy of Higher Education, Manipal, India; [65]Department of Forensic Science, Government Institute of Forensic Science, Nagpur, India; [66]Unit of Biochemistry, Universiti Sultan Zainal Abidin (Sultan Zainal Abidin University), Kuala Terengganu, Malaysia; [67]Department of Forensic Medicine and Toxicology, Manipal Academy of Higher Education, Manipal, India; [68]Department of Hypertension, Medical University of Lodz, Lodz, Poland; [69]Polish Mothers' Memorial Hospital Research Institute, Lodz, Poland; [70]Department of Non-communicable Diseases, Bangladesh University of Health Sciences, Dhaka, Bangladesh; [71]Heidelberg Institute of Global Health (HIGH), Heidelberg University, Heidelberg, Germany; [72]T.H. Chan School of Public Health, Harvard University, Boston, MA, USA; [73]School of Public Health and Community Medicine, Aden College, Aden, Yemen; [74]Health Human Resources Research Center, Shiraz University of Medical Sciences, Shiraz, Iran; [75]Department of Public Health, Ambo University, Ambo, Ethiopia; [76]Department of Community Medicine, Gandhi Medical College Bhopal, Bhopal, India; [77]Jazan University, Jazan, Saudi Arabia; [78]Department of Biomedical Science, Bahir Dar University, Bahir Dar, Ethiopia; [79]Department of Social and Clinical Pharmacy, Charles University, Hradec Kralova, Czech Republic; [80]Institute of Public Health, United Arab Emirates University, Al Ain, United Arab Emirates; [81]School of Public Health, University of Adelaide, Adelaide, SA, Australia; [82]Public Health Research Laboratory, Tribhuvan University, Kathmandu, Nepal; [83]Department of Anatomy, Government Medical College Pali, Pali, India; [84]Department of Community Medicine and Family Medicine, All India Institute of Medical Sciences, Jodhpur, India; [85]School of Public Health, All India Institute of Medical Sciences, Jodhpur, India; [86]Centre for Global Child Health, University of Toronto, Toronto, ON, Canada; [87]Centre of Excellence in Women & Child Health, Aga Khan University, Karachi, Pakistan; [88]Social Determinants of Health Research Center, Babol University of Medical Sciences, Babol, Iran; [89]Department of Environmental and Occupational Health and Safety, University of Gondar, Gondar, Ethiopia; [90]Ethiopian Public Health Institute, Addis Ababa, Ethiopia; [91]Department of Nursing, Debre Tabor University, Debretabor, Ethiopia; [92]Nutrition Department, St. Paul's Hospital Millennium Medical College, Addis Ababa, Ethiopia; [93]St. Paul's Hospital Millennium Medical College, Addis Ababa, Ethiopia; [94]Department of Veterinary Medicine, Islamic Azad University, Kermanshah, Iran; [95]Department of Computer Science and Information Technology, Institute for Advanced Studies in Basic Sciences, Zanjan, Iran; [96]Department of Research and Innovation, Petanux Research GmBH, Bonn, Germany; [97]School of Pharmacy, Haramaya University, Harar, Ethiopia; [98]Department of Internal Medicine, Manipal Academy of Higher Education, Mangalore, India; [99]Department of Infectious Disease Epidemiology, London School of Hygiene & Tropical

Medicine, London, UK; [100]University of Genoa, Genoa, Italy; [101]Department of Internal Medicine, University of São Paulo, São Paulo, Brazil; [102]Department of Psychiatry, University of São Paulo, São Paulo, Brazil; [103]Research Division, Golden Community, Kathmandu, Nepal; [104]Department of Community Medicine, Employee State Insurance Post Graduate Institute of Medical Sciences and Research, Bangalore, India; [105]School of Public Health and Health Systems, University of Waterloo, Waterloo, ON, Canada; [106]Al Shifa School of Public Health, Al Shifa Trust Eye Hospital, Rawalpindi, Pakistan; [107]Department of Health Care, Metropolitan Autonomous University, Mexico City, Mexico; [108]Department of Psychiatry, University of São Paulo, Sao Paulo, Brazil; [109]Gorgas Memorial Institute for Health Studies, Panama City, Panama; [110]Regional Epidemiological Observatory Department, Sicilian Regional Health Authority, Palermo, Italy; [111]Department of Pharmacology, All India Institute of Medical Sciences, Jodhpur, India; [112]Department of International Health, Johns Hopkins Bloomberg School of Public Health, Baltimore, MD, USA; [113]Department of Microbiology & Infection Control, Medanta Medicity, Gurugram, India; [114]Department of Medicine, University of Toronto, Toronto, ON, Canada; [115]Global Institute of Public Health (GIPH), Thiruvananthapuram, India; [116]Research Department, Dr. D. Y. Patil University, Pune, India; [117]Maternal and Child Health Division, International Centre for Diarrhoeal Disease Research, Bangladesh, Dhaka, Bangladesh; [118]Department of Epidemiology and Biostatistics, University of South Carolina, Columbia, SC, USA; [119]Center for Biomedicine and Community Health, VNU International School, Hanoi, Vietnam; [120]Department of Chemical Sciences, University of Porto, Porto, Portugal; [121]Department of Human Physiology, University of Gondar, Gondar, Ethiopia; [122]Department of Cardiology, Central South University, Changsha, China; [123]Department of Mathematics and Statistics, York University, Toronto, ON, Canada; [124]Public Health Foundation of India, Gurugram, India; [125]Indian Council of Medical Research, New Delhi, India; [126]Department of Health Metrics Sciences, School of Medicine, University of Washington, Seattle, WA, USA; [127]Department of Pathology, Johns Hopkins University School of Medicine, Baltimore, MD, USA; [128]Department of Pathology, Isfahan University of Medical Sciences, Isfahan, Iran; [129]Department of Information Technology, University of Human Development, Sulaymaniyah, Iraq; [130]Department of Pediatrics, Tanta University, Tanta, Egypt; [131]Toxoplasmosis Research Center, Mazandaran University of Medical Sciences, Sari, Iran; [132]Division of Women and Child Health, Aga Khan University, Karachi, Pakistan; [133]James P Grant School of Public Health, BRAC University, Dhaka, Bangladesh; [134]Department of Population and Development, Latin American Faculty of Social Sciences Mexico, Mexico City, Mexico; [135]Department of Surgery and Cancer, Imperial College London, London, UK; [136]Ear Institute, University College London, London, UK; [137]Center for Nutrition and Health Research, National Institute of Public Health, Cuernavaca, Mexico; [138]Wellcome Trust Brighton and Sussex Centre for Global Health Research, Brighton and Sussex Medical School, Brighton, UK; [139]School of Public Health, Addis Ababa University, Addis Ababa, Ethiopia; [140]School of Nursing and Midwifery, Haramaya University, Harar, Ethiopia; [141]Department of Epidemiology, Emory University, Atlanta, GA, USA; [142]National Centre for AIDS and STD Control, Save the Children, Kathmandu, Nepal; [143]Department of Global Public Health, Karolinska Institute, Stockholm, Sweden; [144]Department of Community Medicine, University of Peradeniya, Peradeniya, Sri Lanka; [145]Health Research Section, Nepal Health Research Council, Kathmandu, Nepal; [146]Department of Microbiology, Far Western University, Mahendranagar, Nepal; [147]Center of Complexity Sciences, National Autonomous University of Mexico, Mexico City, Mexico; [148]Faculty of Veterinary Medicine and Zootechnics, Autonomous University of Sinaloa, Culiacán Rosales, Mexico; [149]Department of

Public Health, Urmia University of Medical Science, Urmia, Iran; [150]Department of Health Promotion and Education, University of Ibadan, Ibadan, Nigeria; [151]Institute for Global Health Innovations, Duy Tan University, Da Nang, Vietnam; [152]School of Public Health, Hawassa University, Hawassa, Ethiopia; [153]School of Public Health, Curtin University, Perth, WA, Australia; [154]School of Medicine, Federal University of Bahia, Salvador, Brazil; [155]Department of Internal Medicine, Escola Bahiana de Medicina e Saúde Pública (Bahiana School of Medicine and Public Health), Salvador, Brazil; [156]Clinical Pathology Department, Mansoura Faculty of Medicine, Mansoura, Egypt; [157]Pediatric Dentistry and Dental Public Health Department, Alexandria University, Alexandria, Egypt; [158]Department of Food Science and Nutrition, Arsi University, Asella, Ethiopia; [159]Center for Food Science and Nutrition, Addis Ababa University, Addis Ababa, Ethiopia; [160]Neurophysiology Department, Cairo University, Cairo, Egypt; [161]Department of Biology and Biotechnology "Lazzaro Spallanzani", University of Pavia, Pavia, Italy; [162]Department of Biology, Cihan University-Erbil, Erbil, Iraq; [163]Department of Psychology, Federal University of Sergipe, São Cristóvão, Brazil; [164]Non-communicable Diseases Research Center, Tehran University of Medical Sciences, Tehran, Iran; [165]National Institute for Stroke and Applied Neurosciences, Auckland University of Technology, Auckland, New Zealand; [166]Research Center of Neurology, Moscow, Russia; [167]Department of Epidemiology and Biostatistics, Bahir Dar University, Bahir Dar, Ethiopia; [168]School of Nursing, Hawassa University, Hawassa, Ethiopia; [169]Institute of Gerontological Health Services and Nursing Research, Ravensburg-Weingarten University of Applied Sciences, Weingarten, Germany; [170]Institute of Gerontology, National Academy of Medical Sciences of Ukraine, Kyiv, Ukraine; [171]Department of Child Dental Health, Obafemi Awolowo University, Ile-Ife, Nigeria; [172]School of Public Health, Medical, and Veterinary Sciences, James Cook University, Douglas, QLD, Australia; [173]Department of Cardiovascular Medicine, Cleveland Clinic, Cleveland, OH, USA; [174]Gillings School of Global Public Health, University of North Carolina Chapel Hill, Chapel Hill, NC, USA; [175]Department of Medicine, Datta Meghe Institute of Medical Science, Wardha, India; [176]Department of Public Health, Madda Walabu University, Bale Robe, Ethiopia; [177]Department of Nursing and Midwifery, Addis Ababa University, Addis Ababa, Ethiopia; [178]School of Public Health, Haramaya University, Harar, Ethiopia; [179]Department of Neurosurgery, Shiraz University of Medical Sciences, Shiraz, Iran; [180]Faculty of Allied Health Sciences, The University of Lahore, Lahore, Pakistan; [181]Afro-Asian Institute, Lahore, Pakistan; [182]Discipline of Public Health Medicine, University of KwaZulu-Natal, Durban, South Africa; [183]Department of Public Health, Arba Minch University, Arba Minch, Ethiopia; [184]Health Systems and Policy Research, Indian Institute of Public Health Gandhinagar, Gandhinagar, India; [185]Department of Surgery, Madda Walabu University, Bale Robe, Ethiopia; [186]Postgraduate Program in Epidemiology, Federal University of Rio Grande do Sul, Porto Alegre, Brazil; [187]Department of Health Policy, Manipal Academy of Higher Education, Manipal, India; [188]UO Neurologia, Salute Pubblica e Disabilità, Fondazione IRCCS Istituto Neurologico Carlo Besta (Neurology, Public Health and Disability Unit, Carlo Besta Neurological Institute), Milan, Italy; [189]College of Medicine and Health Science, Jigjiga University, Jijiga, Ethiopia; [190]Department of Epidemiology and Preventive Medicine, Monash University, Melbourne, VIC, Australia; [191]Department of Epidemiology, Binzhou Medical University, Yantai City, China; [192]School of Health and Environmental Studies, Hamdan Bin Mohammed Smart University, Dubai, United Arab Emirates; [193]Department of Public Health, Wachemo University, Hossana, Ethiopia; [194]Department of Zoology and Entomology, Al Azhar University, Cairo, Egypt; [195]Institute of Pharmaceutical Sciences, University of Veterinary and

Animal Sciences, Lahore, Pakistan; [196]Department of Pharmacy Administration and Clinical Pharmacy, Xian Jiaotong University, Xian, China; [197]Endocrinology and Metabolism Research Center, Tehran University of Medical Sciences, Tehran, Iran; [198]Nuffield Department of Clinical Medicine, University of Oxford, Oxford, UK; [199]School of Business, London South Bank University, London, UK; [200]Department of Public Health, Adigrat University, Adigrat, Ethiopia; [201]Department of Urban Planning and Design, University of Hong Kong, Hong Kong, China; [202]Center of Excellence in Behavioral Medicine, Nguyen Tat Thanh University, Ho Chi Minh City, Vietnam; [203]Department of Epidemiology and Biostatistics, Tehran University of Medical Sciences, Tehran, Iran; [204]Pediatric Chronic Kidney Disease Research Center, Tehran University of Medical Sciences, Tehran, Iran; [205]College of Science and Engineering, Hamad Bin Khalifa University, Doha, Qatar; [206]Faculty of Medicine of Tunis, University Tunis El Manar, Tunis, Tunisia; [207]Department of Epidemiology and Health Statistics, Central South University, Changsha, China; [208]School of Public Health, University of Sydney, Sydney, NSW, Australia; [209]Department of Occupational Safety and Health, China Medical University, Taichung, Taiwan; [210]Department of Community Medicine, University of Ibadan, Ibadan, Nigeria; [211]Department of Community Medicine, University College Hospital, Ibadan, Ibadan, Nigeria; [212]Faculty of Medicine, University of Belgrade, Belgrade, Serbia; [213]Department of Epidemiology, University of Kragujevac, Kragujevac, Serbia; [214]Division of Community Health and Family Medicine, Bangalore Baptist Hospital, Bangalore, India; [215]College of Public Health, Taipei Medical University, Taipei, Taiwan; [216]Research Institute for Endocrine Sciences, Shahid Beheshti University of Medical Sciences, Tehran, Iran; [217]School of Psychology and Public Health, La Trobe University, Melbourne, VIC, Australia; [218]School of Health Systems and Public Health, University of Pretoria, Pretoria, South Africa; [219]South African Medical Research Council, Cape Town, South Africa; [220]Department of Community Medicine, Manipal Academy of Higher Education, Mangalore, India; [221]Manipal College of Pharmaceutical Sciences, Manipal Academy of Higher Education, Manipal, India; [222]Health Informatic Lab, Boston University, Boston, MA, USA; [223]Gastrointestinal and Liver Diseases Research Center, Guilan University of Medical Sciences, Rasht, Iran; [224]Caspian Digestive Disease Research Center, Guilan University of Medical Sciences, Rasht, Iran; [225]Department of Family Medicine and Public Health, University of Opole, Opole, Poland; [226]Minimally Invasive Surgery Research Center, Iran University of Medical Sciences, Tehran, Iran; [227]School of Management and Medical Informatics, Tabriz University of Medical Sciences, Tabriz, Iran; [228]Institute for Prevention of Non-communicable Diseases, Qazvin University of Medical Sciences, Qazvin, Iran; [229]Health Services Management Department, Qazvin University of Medical Sciences, Qazvin, Iran; [230]Manipal Academy of Higher Education, India; [231]Department of Biostatistics, Hamadan University of Medical Sciences, Hamadan, Iran; [232]Department of Forensic Medicine and Toxicology, All India Institute of Medical Sciences, Jodhpur, India; [233]Research Center for Environmental Determinants of Health, Kermanshah University of Medical Sciences, Kermanshah, Iran; [234]Social Determinants of Health Research Center, Tabriz University of Medical Sciences, Tabriz, Iran; [235]School of Nursing and Midwifery, Wollega University, Nekemte, Ethiopia; [236]School of Midwifery, Hawassa University, Hawassa, Ethiopia; [237]Department of Anesthesiology & Pain Medicine, University of Washington, Seattle, WA, USA; [238]International Research Center of Excellence, Institute of Human Virology Nigeria, Abuja, Nigeria; [239]Julius Centre for Health Sciences and Primary Care, Utrecht University, Utrecht, Netherlands; [240]Open, Distance and eLearning Campus, University of Nairobi, Nairobi, Kenya; [241]Department of Midwifery, University of Gondar, Gondar, Ethiopia; [242]School of Food and Agricultural

Sciences, University of Management and Technology, Lahore, Pakistan; [243]Department of Population Science, Jatiya Kabi Kazi Nazrul Islam University, Mymensingh, Bangladesh; [244]Faculty of Health and Wellbeing, Sheffield Hallam University, Sheffield, UK; [245]College of Arts and Sciences, Ohio University, Zanesville, OH, USA; [246]National Hepatology and Tropical Medicine Research Institute, Cairo University, Cairo, Egypt; [247]Department of Medical Parasitology, Cairo University, Cairo, Egypt; [248]Global Evidence Synthesis Initiative, Datta Meghe Institute of Medical Sciences, Wardha, India; [249]School of Traditional Chinese Medicine, Xiamen University Malaysia, Sepang, Malaysia; [250]Department of Nutrition, Simmons University, Boston, MA, USA; [251]School of Health Sciences, Kristiania University College, Oslo, Norway; [252]Global Community Health and Behavioral Sciences, Tulane University, New Orleans, LA, USA; [253]Department of Nursing and Health Promotion, Oslo Metropolitan University, Oslo, Norway; [254]Independent Consultant, Jakarta, Indonesia; [255]Department of Anthropology, Panjab University, Chandigarh, India; [256]Department of Psychiatry, University of Nairobi, Nairobi, Kenya; [257]Division of Psychology and Language Sciences, University College London, London, UK; [258]International Institute for Population Sciences, Mumbai, India; [259]Faculty of Health and Life Sciences, Coventry University, Coventry, UK; [260]Department of Medicine, McMaster University, Hamilton, ON, Canada; [261]Imperial College Business School, Imperial College London, London, UK; [262]Faculty of Public Health, University of Indonesia, Depok, Indonesia; [263]Department of Clinical Sciences and Community Health, University of Milan, Milan, Italy; [264]Department of Pediatrics, Post Graduate Institute of Medical Education and Research, Chandigarh, India; [265]Department of Community and Family Medicine, University of Baghdad, Baghdad, Iraq; [266]Unit of Genetics and Public Health, Institute of Medical Sciences, Las Tablas, Panama; [267]Ministry of Health, Herrera, Panama; [268]HelpMeSee, New York, NY, USA; [269]Mexican Institute of Ophthalmology, Queretaro, Mexico; [270]Department of Otorhinolaryngology, Father Muller Medical College, Mangalore, India; [271]School of Nursing, Hong Kong Polytechnic University, Hong Kong, China; [272]Centre for Tropical Medicine and Global Health, University of Oxford, Oxford, UK; [273]Oxford University Clinical Research Unit, Wellcome Trust Asia Programme, Hanoi, Vietnam; [274]Department of Sociology, Shenzhen University, Shenzhen, China; [275]Department of Public Health, China Medical University, Taichung, Taiwan; [276]School of Public Health and Preventive Medicine, Monash University, Melbourne, VIC, Australia; [277]Department of Systems, Populations, and Leadership, University of Michigan, Ann Arbor, MI, USA; [278]Department of Paediatrics, All India Institute of Medical Sciences, New Delhi, India; [279]Department of Nutrition, University of the Philippines Manila, Manila, Philippines; [280]Alliance for Improving Health Outcomes, Inc., Quezon City, Philippines; [281]Center for Integration of Data and Health Knowledge, Oswald Cruz Foundation (FIOCRUZ), Salvador, Brazil; [282]Centre for Global Mental Health (CGMH), London School of Hygiene & Tropical Medicine, London, England; [283]Department of Forensic Medicine, Shri Dharmasthala Manjunatheshwara University, Dharwad, India; [284]Department of Forensic Medicine, Rajiv Gandhi University of Health Sciences, Bangalore, India; [285]Clinical Research Development Center, Kermanshah University of Medical Sciences, Kermanshah, Iran; [286]Department of Maternal and Child Nursing and Public Health, Federal University of Minas Gerais, Belo Horizonte, Brazil; [287]Institute for Social Science Research, The University of Queensland, Indooroopilly, QLD, Australia; [288]School of Medicine, Iran University of Medical Sciences, Tehran, Iran; [289]School of Medicine, University of Manitoba, Winnipeg, MB, Canada; [290]Department of Population Studies, University of Zambia, Lusaka, Zambia; [291]Campus Caucaia, Federal Institute of Education, Science and Technology of Ceará, Caucaia, Brazil;

[292]Neurology Department, Janakpuri Super Specialty Hospital Society, New Delhi, India; [293]Department of Neurology, Govind Ballabh Institute of Medical Education and Research, New Delhi, India; [294]Research in Nutrition and Health, National Institute of Public Health, Cuernavaca, Mexico; [295]Department of Public Health, Wollo University, Dessie, Ethiopia; [296]Peru Country Office, United Nations Population Fund (UNFPA), Lima, Peru; [297]Forensic Medicine Division, Imam Abdulrahman Bin Faisal University, Dammam, Saudi Arabia; [298]Department of Reproductive Health and Population Studies, Bahir Dar University, Bahir Dar, Ethiopia; [299]Department of Nursing, Arba Minch University, Arba Minch, Ethiopia; [300]Pacific Institute for Research & Evaluation, Calverton, MD, USA; [301]Global Institute of Public Health, Ananthapuri Hospitals and Research Institute, Trivandrum, India; [302]Women's Social and Health Studies Foundation, Trivandrum, India; [303]Internal Medicine Programme, Kyrgyz State Medical Academy, Bishkek, Kyrgyzstan; [304]Department of Atherosclerosis and Coronary Heart Disease, National Center of Cardiology and Internal Disease, Bishkek, Kyrgyzstan; [305]Department of Surgical Oncology, All India Institute of Medical Sciences, Jodhpur, India; [306]Biotechnology Research Center, Tabriz University of Medical Sciences, Tabriz, Iran; [307]Molecular Medicine Research Center, Tabriz University of Medical Sciences, Tabriz, Iran; [308]Department of Forestry, Salahaddin University-Erbil, Erbil, Iraq; [309]Department of Medicine-Huddinge, Karolinska Institute, Stockholm, Sweden; [310]Department of Epidemiology and Biostatistics, Shahrekord University of Medical Sciences, Shahrekord, Iran; [311]Department of Public Health, Samara University, Semera, Ethiopia; [312]Health Systems and Policy Research Unit, Ahmadu Bello University, Zaria, Nigeria; [313]Department of Public Health Sciences, University of Miami, Miami, FL, USA; [314]Center for Health Systems Research, National Institute of Public Health, Cuernavaca, Mexico; [315]Department of Epidemiology, Arak University of Medical Sciences, Arak, Iran; [316]Computer, Electrical, and Mathematical Sciences and Engineering Division, King Abdullah University of Science and Technology, Thuwal, Saudi Arabia; [317]Management and Leadership in Medical Education Research Center, Kerman University of Medical Sciences, Kerman, Iran; [318]Department of Food Science, University of Campinas (Unicamp), Campinas, Brazil; [319]School of Economics, University of Nairobi, Nairobi, Kenya; [320]Department of Pediatric Medicine, The Children's Hospital & The Institute of Child Health, Multan, Pakistan; [321]Department of Pediatrics & Pediatric Pulmonology, Institute of Mother & Child Care, Multan, Pakistan; [322]Department of Microbiology and Immunology, Mekelle University, Mekelle, Ethiopia; [323]Research and Analytics Department, Initiative for Financing Health and Human Development, Chennai, India; [324]Department of Research and Analytics, Bioinsilico Technologies, Chennai, India; [325]Comprehensive Cancer Center, University of Alabama at Birmingham, Birmingham, AL, USA; [326]Laboratory of Public Health Indicators Analysis and Health Digitalization, Moscow Institute of Physics and Technology, Dolgoprudny, Russia; [327]Experimental Surgery and Oncology Laboratory, Kursk State Medical University, Kursk, Russia; [328]Suraj Eye Institute, Nagpur, India; [329]Department of Clinical Medicine, Federal University of Minas Gerais, Belo Horizonte, Brazil; [330]Clinical Hospital, Federal University of Minas Gerais, Belo Horizonte, Brazil; [331]Disease Control and Environmental Health, Makerere University, Kampala, Uganda; [332]Cochrane South Africa, South African Medical Research Council, Cape Town, South Africa; [333]Department of General Surgery, Carol Davila University of Medicine and Pharmacy, Bucharest, Romania; [334]Department of General Surgery, Emergency Hospital of Bucharest, Bucharest, Romania; [335]Department of Public Health, University of Yaoundé I, Yaoundé, Cameroon; [336]Department of Biological Sciences, University of Embu, Embu, Kenya; [337]Institute for Global Health Innovations, Duy Tan University, Hanoi, Vietnam;

[338]Faculty of Pharmacy, Duy Tan University, Da Nang, Vietnam; [339]Department of Public Health, Mizan-Tepi University, Mizan Teferi, Ethiopia; [340]Obesity Research Center, Shahid Beheshti University of Medical Sciences, Tehran, Iran; [341]School of Public Health and Family Medicine, University of Cape Town, Cape Town, South Africa; [342]Unit of Microbiology and Public Health, Institute of Medical Sciences, Las Tablas, Panama; [343]Department of Public Health, Ministry of Health, Herrera, Panama; [344]Administrative and Economic Sciences Department, University of Bucharest, Bucharest, Romania; [345]Department of Obstetrics and Gynecology, University of Ibadan, Ibadan, Nigeria; [346]Department of Psychiatry and Behavioural Neurosciences, McMaster University, Hamilton, ON, Canada; [347]Department of Psychiatry, University of Lagos, Lagos, Nigeria; [348]Centre for Healthy Start Initiative, Lagos, Nigeria; [349]Department of Public Health, Jigjiga University, Jijiga, Ethiopia; [350]Department of Pharmacology and Therapeutics, University of Nigeria Nsukka, Enugu, Nigeria; [351]Health Systems Research Center, National Institute of Public Health, Cuernavaca, Mexico; [352]Department of Project Management, National Research University Higher School of Economics, Moscow, Russia; [353]Department of Medicine, University of Ibadan, Ibadan, Nigeria; [354]Department of Medicine, University College Hospital, Ibadan, Ibadan, Nigeria; [355]Department of Respiratory Medicine, Jagadguru Sri Shivarathreeswara Academy of Health Education and Research, Mysore, India; [356]Department of Health Metrics, Center for Health Outcomes & Evaluation, Bucharest, Romania; [357]Department of Research, Public Health Foundation of India, Gurugram, India; [358]Corporate, Somnogen Canada Inc, Toronto, ON, Canada; [359]National Institute of Health Research and Development, Ministry of Health, Jakarta, Indonesia; [360]Public Health Evidence South Asia, Manipal Academy of Higher Education, Manipal, India; [361]Division of General Internal Medicine, University of Pittsburgh Medical Center, Pittsburgh, PA, USA; [362]Department of Neurology and Public Health, Icahn School of Medicine at Mount Sinai, New York, NY, USA; [363]Department of Pediatrics, RD Gardi Medical College, Ujjain, India; [364]Global Public Health-Health Systems and Policy (HSP): Medicines Focusing Antibiotics, Karolinska Institute, Stockholm, Sweden; [365]Research & Development Department, Kalinga Institute of Medical Sciences, Bhubaneswar, India; [366]Department of Pediatrics, University of Melbourne, Melbourne, VIC, Australia; [367]Population Health Theme, Murdoch Childrens Research Institute, Melbourne, VIC, Australia; [368]Department of Midwifery, Wolaita Sodo University, Wolaita Sodo, Ethiopia; [369]Center for Research and Innovation, Ateneo De Manila University, Pasig City, Philippines; [370]Department of Chemistry, University of Porto, Porto, Portugal; [371]Global Development Program, Bill & Melinda Gates Foundation, Seattle, WA, USA; [372]HIV and Mental Health Department, Integrated Development Foundation Nepal, Kathmandu, Nepal; [373]Department of Nutrition and Food Sciences, Maragheh University of Medical Sciences, Maragheh, Iran; [374]Dietary Supplements and Probiotic Research Center, Alborz University of Medical Sciences, Karaj, Iran; [375]Centro de Investigaciones Clinicas, Fundación Valle del Lili, (Clinical Research Center, Valle del Lili Foundation), Cali, Colombia; [376]Centro PROESA, Universidad ICESI, (PROESA, ICESI University), Cali, Colombia; [377]Health Sciences Department, Muhammadiyah University of Surakarta, Sukoharjo, Indonesia; [378]Department of Community Medicine, Datta Meghe Institute of Medical Sciences, Wardha, India; [379]Biomedical Engineering Department, Amirkabir University of Technology, Tehran, Iran; [380]Department of Chemistry, Sharif University of Technology, Tehran, Iran; [381]Thalassemia and Hemoglobinopathy Research Center, Ahvaz Jundishapur University of Medical Sciences, Ahvaz, Iran; [382]Metabolomics and Genomics Research Center, Tehran University of Medical Sciences, Tehran, Iran; [383]Department of Natural Science, Middlesex University, London, UK;

[384]Data Mining Research Unit (DaMRA), Charles Sturt University, Wagga Wagga, NSW, Australia; [385]Department of Community Medicine, Maharishi Markandeshwar Medical College & Hospital, Solan, India; [386]Future Technology Research Center, National Yunlin University of Science and Technology, Yunlin, Taiwan; [387]Institute of Research and Development, Duy Tan University, Da Nang, Vietnam; [388]Society for Health and Demographic Surveillance, Suri, India; [389]Department of Economics, University of Göttingen, Göttingen, Germany; [390]Research Department, Policy Research Institute, Kathmandu, Nepal; [391]Health and Public Policy Department, Global Center for Research and Development, Kathmandu, Nepal; [392]Department of Oral Pathology, Srinivas Institute of Dental Sciences, Mangalore, India; [393]Department of Forensic Medicine and Toxicology, Manipal Academy of Higher Education, Mangalore, India; [394]WHO Collaborating Centre for Public Health Education and Training, Imperial College London, London, UK; [395]University College London Hospitals, London, UK; [396]Department of Primary Care and Public Health, Imperial College London, London, UK; [397]Academic Public Health England, Public Health England, London, UK; [398]Department of Computer Science, Boston University, Boston, MA, USA; [399]Maternal, Newborn, and Child Health Program, Bill & Melinda Gates Foundation, Seattle, WA, USA; [400]Department of Mathematical Demography & Statistics, International Institute for Population Sciences, Mumbai, India; [401]Department of Pediatrics, Federal University of Minas Gerais, Belo Horizonte, Brazil; [402]Department of Health Information Management, Manipal Academy of Higher Education, Manipal, India; [403]Manipal Academy of Higher Education, Manipal, India; [404]Epidemiology Research Unit Institute of Public Health (EPIUnit-ISPUP), University of Porto, Porto, Portugal; [405]Department of Surgery, University of Minnesota, Minneapolis, MN, USA; [406]Department of Surgery, University Teaching Hospital of Kigali, Kigali, Rwanda; [407]Department of Clinical Research, Federal University of Uberlândia, Uberlândia, Brazil; [408]Malaria Atlas Project, University of Oxford, Oxford, UK; [409]Department of Health Statistics, National Institute for Medical Research, Dar es Salaam, Tanzania; [410]Department of Internal Medicine, University of Botswana, Gaborone, Botswana; [411]Department of Psychiatry, All India Institute of Medical Sciences, New Delhi, India; [412]Department of Phytochemistry, Soran University, Soran, Iraq; [413]Department of Nutrition, Cihan University-Erbil, Erbil, Iraq; [414]Public Health and Community Medicine Department, Cairo University, Giza, Egypt; [415]Department of Entomology, Ain Shams University, Cairo, Egypt; [416]School of Public Health and Health Management, University of Belgrade, Belgrade, Serbia; [417]Department of Community Medicine, PSG Institute of Medical Sciences and Research, Coimbatore, India; [418]PSG-FAIMER South Asia Regional Institute, Coimbatore, India; [419]Health Economics Department, Bangladesh Institute of Development Studies (BIDS), Dhaka, Bangladesh; [420]Centre for Tropical Medicine and Global Health, University of Oxford, Oxford, UK; [421]Nuffield Department of Medicine, University of Oxford, Oxford, ; [422]Department of Geriatrics and Long Term Care, Hamad Medical Corporation, Doha, Qatar; [423]Faculty of Health & Social Sciences, Bournemouth University, Bournemouth, UK; [424]Department of Epidemiology, Indian Institute of Public Health, Gandhinagar, India; [425]Department of Computational Biology, Indraprastha Institute of Information Technology, Delhi, India; [426]Emergency Department, Manian Medical Centre, Erode, India; [427]Center for Biomedical Information Technology, Shenzhen Institutes of Advanced Technology, Shenzhen, China; [428]Department of Radiology and Radiological Science, Johns Hopkins University, Baltimore, MD, USA; [429]Department of Radiology and Interventional Neuroradiology, Isfahan University of Medical Sciences, Isfahan, Iran; [430]Public Health Division, An-Najah National University, Nablus, Palestine; [431]Independent Consultant, Karachi, Pakistan; [432]Neurology

Department, Ain Shams University, Cairo, Egypt; [433]Department of Community Medicine, BLDE University, Vijayapur, India; [434]Centre for Medical Informatics, University of Edinburgh, Edinburgh, UK; [435]Division of General Internal Medicine, Harvard University, Boston, MA, USA; [436]Department of Community Medicine, Manipal Academy of Higher Education, Manipal, India; [437]Institute for Population Health, King's College London, London, UK; [438]College of Medicine, Yonsei University, Seoul, South Korea; [439]Department of Physical Education, Federal University of Santa Catarina, Florianópolis, Brazil; [440]Faculty of Medicine and Health Sciences, Shree Guru Gobind Singh Tricentenary University, Gurugram, India; [441]Department of Humanities and Social Sciences, Indian Institute of Technology, Roorkee, Roorkee, India; [442]Division of Environmental Monitoring & Exposure Assessment (Water & Soil), National Institute for Research in Environmental Health, Bhopal, India; [443]Department of Midwifery, Haramaya University, Harar, Ethiopia; [444]Department No.16, Moscow Research and Practical Centre on Addictions, Moscow, Russia; [445]Therapeutic Department, Balashiha Central Hospital, Balashikha, Russia; [446]Nursing Care Research Center, Semnan University of Medical Sciences, Semnan, Iran; [447]Department of Nursing, Muhammadiyah University of Surakarta, Surakarta, Indonesia; [448]Department of Community Medicine, Ahmadu Bello University, Zaria, Nigeria; [449]School of Medicine, University of California San Francisco, San Francisco, CA, USA; [450]Joint Medical Program, University of California Berkeley, Berkeley, CA, USA; [451]Department of Biomedical Sciences, Arba Minch University, Arba Minch, Ethiopia; [452]Non-communicable Diseases Research Center, Hamadan University of Medical Sciences, Hamadan, Iran; [453]Department of Population Science and Human Resource Development, University of Rajshahi, Rajshahi, Bangladesh; [454]Research and Development Center for Humanities and Health Management, National Institute of Health Research & Development, Jakarta, Indonesia; [455]Department of Epidemiology and Biostatistsics, University of Gondar, Gondar, Ethiopia; [456]School of Public Health, Mekelle University, Mekelle, Ethiopia; [457]Southgate Institute for Health and Society, Flinders University, Adelaide, SA, Australia; [458]Department of Epidemiology and Biostatistics, University of Gondar, Gondar, Ethiopia; [459]Department of Public Health and Community Medicine, Central University of Kerala, Kasaragod, India; [460]Department of Endocrinology, Diabetes and Metabolism, Christian Medical College and Hospital (CMC), Vellore, India; [461]Institute of Public Health, Jagiellonian University Medical College, Kraków, Poland; [462]Agency for Health Technology Assessment and Tariff System, Warsaw, Poland; [463]Department of Pathology and Legal Medicine, University of São Paulo, Ribeirão Preto, Brazil; [464]Modestum LTD, London, UK; [465]Institute for Risk Assessment Sciences (IRAS), Utrecht University, Utrecht, Netherlands; [466]Department of Health Economics, Hanoi Medical University, Hanoi, Vietnam; [467]Faculty of Geo-Information Science and Earth Observation, University of Twente, Enschede, Netherlands; [468]Department of Allied Health Sciences, Iqra National University, Peshawar, Pakistan; [469]Department of Community Medicine, Alex Ekwueme Federal University Teaching Hospital Abakaliki, Abakaliki, Nigeria; [470]Kasturba Medical College, Manipal Academy of Higher Education, Mangalore, India; [471]Amity Institute of Biotechnology, Amity University Rajasthan, Jaipur, India; [472]Department of Community Medicine, University of Nigeria Nsukka, Enugu, Nigeria; [473]Insights Program, Bill & Melinda Gates Foundation, Seatte, WA, USA; [474]Department of Medical and Surgical Sciences, University of Bologna, Bologna, Italy; [475]Occupational Health Unit, Sant'Orsola Malpighi Hospital, Bologna, Italy; [476]Faculty of Information Technology, Ho Chi Minh City University of Technology (HUTECH), Ho Chi Minh City, Vietnam; [477]Population Dynamics and Sexual and Reproductive Health, African Population and Health Research Center, Nairobi, Kenya;

[478]Foundation University Medical College, Foundation University Islamabad, Islamabad, Pakistan; [479]Cultures, Societies and Global Studies, & Integrated Initiative for Global Health, Northeastern University, Boston, MA, USA; [480]School of Public Health, University of Nairobi, Nairobi, Kenya; [481]School of Health Sciences, Wuhan University, Wuhan, China; [482]Department of Epidemiology and Biostatistics, Wuhan University, Wuhan, China; [483]Department of Community Medicine, Rajarata University of Sri Lanka, Anuradhapura, Sri Lanka; [484]Department of Epidemiology, Johns Hopkins University, Baltimore, MD, USA; [485]Department of Orthopaedics, Wenzhou Medical University, Wenzhou, China; [486]Global Health Research Center, Duke Kunshan University, Kunshan, China; [487]Duke Global Health Institute, Duke University, Durham, NC, USA; [488]Department of Diabetes and Metabolic Diseases, University of Tokyo, Tokyo, Japan; [489]School of International Development and Global Studies, University of Ottawa, Ottawa, ON, Canada; [490]The George Institute for Global Health, University of Oxford, Oxford, UK; [491]Department of Midwifery, Wolkite University, Wolkite, Ethiopia; [492]Department of Public Health, Wollega University, Nekemte, Ethiopia; [493]Centre for Suicide Research and Prevention, University of Hong Kong, Hong Kong, China; [494]Department of Social Work and Social Administration, University of Hong Kong, Hong Kong, China; [495]Department of Neuropsychopharmacology, National Center of Neurology and Psychiatry, Kodaira, Japan; [496]Department of Public Health, Juntendo University, Tokyo, Japan; [497]Department of Health Policy and Management, Jackson State University, Jackson, MS, USA; [498]School of Medicine, Tsinghua University, Beijing, China; [499]School of Public Health and Management, Hubei University of Medicine, Shiyan, China; [500]Cancer Institute, Hacettepe University, Ankara, Turkey; [501]Department of Obstetrics and Gynaecology, Fazaia Medical College, Islamabad, Pakistan; [502]Department of Obstetrics and Gynaecology, Air University, Islamabad, Pakistan; [503]Department of Pharmaceutics, Dow University of Health Sciences, Karachi, Pakistan; [504]Department of Parasitology and Entomology, Tarbiat Modares University, Tehran, Iran; [505]Institute for Immunological Research, University of Cartagena, Cartagena, Colombia; [506]Department of Paediatrics & Child Health, University of Cape Town, Cape Town, South Africa; [507]Unit on Child & Adolescent Health, Medical Research Council South Africa, Cape Town, South Africa; [508]Laboratory of Genetics and Genomics, Moscow Research and Practical Centre on Addictions, Moscow, Russia; [509]Addictology Department, Russian Medical Academy of Continuous Professional Education, Moscow, Russia; [510]Pediatrics Department, Russian Medical Academy of Continuous Professional Education, Moscow, Russia; [511]Department of Pediatrics and Child Health Nursing, Bahir Dar University, Bahir Dar, Ethiopia; [512]School of Public Health, Wuhan University of Science and Technology, Wuhan, China; [513]Hubei Province Key Laboratory of Occupational Hazard Identification and Control, Wuhan University of Science and Technology, Wuhan, China; [514]School of Medicine, Wuhan University, Wuhan, China; [515]School of Biology and Pharmaceutical Engineering, Wuhan Polytechnic University, Wuhan, China; [516]Indian Institute of Public Health, Public Health Foundation of India, Gurugram, India; [517]Health Technology Assessment Unit, Department of Health Philippines, Manila, Philippines; [518]#MentalHealthPH, Inc., Quezon City, Philippines.

9.0. Author Contributions

Providing data or critical feedback on data sources

Natalia V Bhattacharjee, Dan Lu, Megan F Schipp, Gdiom Gebreheat Abady, Akine Eshete Abosetugn, Michael R M Abrigo, Isaac Akinkunmi Adedeji, Victor Adekanmbi, Olatunji O Adetokunboh, Budi Aji, Fahad Mashhour Alanezi, Turki M Alanzi, Jacqueline Elizabeth Alcalde-Rabanal, Beriwan Abdulqadir Ali, Saqib Ali, Mina Anjomshoa, Fereshteh Ansari, Davood Anvari, Jalal Arabloo, Amit Arora, Kurnia Dwi Artanti, Mulusew A Asemahagn, Wondwossen Niguse Asmare, Maha Moh'd Wahbi Atout, Marcel Ausloos, Nefsu Awoke, Beatriz Paulina Ayala Quintanilla, Yared Asmare Aynalem, Muluken Altaye Ayza, Maciej Banach, Palash Chandra Banik, Till Winfried Bärnighausen, Sefealem Assefa Belay, Akshaya Srikanth Bhagavathula, Binyam Minuye Birihane, Zebenay Workneh Bitew, Somayeh Bohlouli, Mahdi Bohluli, Archith Boloor, Nicola Luigi Bragazzi, Shyam S Budhathoki, Sharath Burugina Nagaraja, Joao Mauricio Castaldelli-Maia, Franz Castro, Vijay Kumar Chattu, Dinh-Toi Chu, Rosa A S Couto, Lalit Dandona, Rakhi Dandona, Aso Mohammad Darwesh, Amira Hamed Darwish, Ahmad Daryani, Rajat Das Gupta, Awrajaw Dessie, Samath Dhamminda Dharmaratne, Meghnath Dhimal, Govinda Prasad Dhungana, Linh Phuong Doan, Lucas Earl, Maysaa El Sayed Zaki, Teshome Bekele Elema, Andre Faro, Farshad Farzadfar, Berhanu Elfu Feleke, Richard Charles Franklin, Mohamed M Gad, Shilpa Gaidhane, William M Gardner, Birhan Gebresillassie Gebregiorgis, Ketema Bizuwork Gebremedhin, Themba G Ginindza, Mahaveer Golechha, Dr Kebebe Bekele Gonfa, Bárbara Niegia Garcia Goulart, Yuming Guo, Ahmed I Hasaballah, Mohamed I Hegazy, Behnam Heidari, Claudiu Herteliu, Hung Chak Ho, Chi Linh Hoang, Julia Hon, Mehdi Hosseinzadeh, Mowafa Househ, Mohamed Hsairi, Guoqing Hu, Segun Emmanuel Ibitoye, Usman Iqbal, Seyed Sina Naghibi Irvani, Chidozie C D Iwu, Animesh Jain, Tahereh Javaheri, Yetunde O John-Akinola, Kimberly B Johnson, Farahnaz Joukar, Jacek Jerzy Jozwiak, Ashwin Kamath, Naser Kamyari, Salah Eddin Karimi, Nicholas J Kassebaum, Gbenga A Kayode, Nauman Khalid, Md Nuruzzaman Khan, Khaled Khatab, Amir M Khater, Mona M Khater, Mahalaqua Nazli Khatib, Damaris K Kinyoki, Adnan Kisa, Sezer Kisa, Soewarta Kosen, Kewal Krishan, G Anil Kumar, Pushpendra Kumar, Dian Kusuma, Van Charles Lansingh, Savita Lasrado, Shanshan Li, Xuefeng Liu, Jaifred Christian F Lopez, Daiane Borges Machado, Deborah Carvalho Malta, Navid Manafi, Francisco Rogerlândio Martins-Melo, Man Mohan Mehndiratta, Walter Mendoza, Endalkachew Worku Mengesha, Erkin M Mirrakhimov, Masoud Moghadaszadeh, Abdollah Mohammadian-Hafshejani, Jemal Abdu Mohammed, Shafiu Mohammed, Ali H Mokdad, Pablo A Montero-Zamora, Masoud Moradi, Seyyed Meysam Mousavi, Ahamarshan Jayaraman Nagarajan, Bruno Ramos Nascimento, Ionut Negoi, Georges Nguefack-Tsague, Josephine W Ngunjiri, Cuong Tat Nguyen, Huong Lan Thi Nguyen, Samuel Negash Nigussie, Chukwudi A Nnaji, Bogdan Oancea, Onome Bright Oghenetega, Andrew T Olagunju, Bolajoko Olubukunola Olusanya, Jacob Olusegun Olusanya, Obinna E Onwujekwe, Mayowa O Owolabi, Mahesh P A, Jagadish Rao Padubidri, Adrian Pana, Anamika Pandey, Urvish K Patel, Ashish Pathak, Sanjay M Pattanshetty, Kebreab Paulos, Veincent Christian Filipino Pepito, Brandon V Pickering, Marina Pinheiro, Hadi Pourjafar, Sergio I Prada, Dimas Ria Angga Pribadi, Zahiruddin Quazi Syed, Fakher Rahim, Azizur Rahman, Mohammad Hifz Ur Rahman, Amir Masoud Rahmani, Chhabi Lal Ranabhat, Sowmya J Rao, Priya Rathi, David Laith Rawaf, Salman Rawaf, Reza Rawassizadeh, Ramu Rawat, Lemma Demissie Regassa, Robert C Reiner Jr., Bhageerathy Reshmi, Leonardo Roever, Rajesh Sagar, S. Mohammad Sajadi, Abdallah M Samy, Milena M Santric-Milicevic, Sivan Yegnanarayana Iyer Saraswathy,

Abdur Razzaque Sarker, Benn Sartorius, Brijesh Sathian, Deepak Saxena, Debarka Sengupta, Feng Sha, Masood Ali Shaikh, Mohammed Shannawaz, B Suresh Kumar Shetty, Wondimeneh Shibabaw Shiferaw, Jae Il Shin, Valentin Yurievich Skryabin, Anna Aleksandrovna Skryabina, Amin Soheili, Emma Elizabeth Spurlock, Agus Sudaryanto, Scott J Swartz, Animut Tagele Tamiru, Marcos Roberto Tovani-Palone, Bach Xuan Tran, Phuong N Truong, Berhan Tsegaye BT Tsegaye, Irfan Ullah, Bhaskaran Unnikrishnan, Era Upadhyay, John David VanderHeide, Bay Vo, Yasir Waheed, Yafeng Wang, Charles Shey Wiysonge, Sanni Yaya, Alex Yeshaneh, Yordanos Gizachew Yeshitila, Naohiro Yonemoto, Mustafa Z Younis, Abdilahi Yousuf Yousuf, Chuanhua Yu, Syed Saoud Zaidi, Josefina Zakzuk, Mikhail Sergeevich Zastrozhin, Desalege Amare Zelellw, Zhi-Jiang Zhang, Yves Miel H Zuniga, and Simon I Hay

Developing methods or computational machinery

Natalia V Bhattacharjee, Saqib Ali, Nefsu Awoke, Yared Asmare Aynalem, Palash Chandra Banik, Somayeh Bohlouli, Mahdi Bohluli, Michael L Collison, Aso Mohammad Darwesh, Ahmad Daryani, Aniruddha Deshpande, Meghnath Dhimal, Maysaa El Sayed Zaki, William M Gardner, Yuming Guo, Nathaniel J Henry, Mowafa Househ, Tahereh Javaheri, Leila R Kalankesh, Amir M Khater, Adnan Kisa, Sezer Kisa, Shanshan Li, Masoud Moghadaszadeh, Shafiu Mohammed, Ali H Mokdad, Saravanan Muthupandian, Josephine W Ngunjiri, Aaron E Osgood-Zimmerman, Chhabi Lal Ranabhat, Ramu Rawat, Robert C Reiner Jr., Abdallah M Samy, Abdur Razzaque Sarker, Deepak Saxena, Alyssa N Sbarra, Emma Elizabeth Spurlock, Krista M Steuben, Scott J Swartz, Animut Tagele Tamiru, John David VanderHeide, Kirsten E Wiens, Desalege Amare Zelellw, and Simon I Hay

Providing critical feedback on methods or results

Natalia V Bhattacharjee, Lauren E Schaeffer, Alice Lazzar-Atwood, Gdiom Gebreheat Abady, Foad Abd-Allah, Ahmed Abdelalim, Ayenew Negesse Abejie, Akine Eshete Abosetugn, Lucas Guimarães Abreu, Michael R M Abrigo, Eman Abu-Gharbieh, Abdelrahman I Abushouk, Isaac Akinkunmi Adedeji, Adeyinka Emmanuel Adegbosin, Victor Adekanmbi, Olatunji O Adetokunboh, Marcela Agudelo-Botero, Budi Aji, Oluwaseun Oladapo Akinyemi, Alehegn Aderaw Alamneh, Fahad Mashhour Alanezi, Turki M Alanzi, Jacqueline Elizabeth Alcalde-Rabanal, Biresaw Wassihun Alemu, Robert Kaba Alhassan, Beriwan Abdulqadir Ali, Saqib Ali, Cyrus Alinia, Vahid Alipour, Arianna Maever L Amit, Dickson A Amugsi, Etsay Woldu Anbesu, Robert Ancuceanu, Fereshteh Ansari, Carl Abelardo T Antonio, Davood Anvari, Jalal Arabloo, Amit Arora, Kurnia Dwi Artanti, Mulusew A Asemahagn, Wondwossen Niguse Asmare, Maha Moh'd Wahbi Atout, Marcel Ausloos, Nefsu Awoke, Beatriz Paulina Ayala Quintanilla, Martin Amogre Ayanore, Muluken Altaye Ayza, Zelalem Nigussie Azene, Darshan B B, Ashish D Badiye, Maciej Banach, Palash Chandra Banik, Till Winfried Bärnighausen, Bayisa Abdissa Baye, Sefealem Assefa Belay, Akshaya Srikanth Bhagavathula, Dinesh Bhandari, Nikha Bhardwaj, Pankaj Bhardwaj, Zulfiqar A Bhutta, Ali Bijani, Tsegaye Adane Birhan, Binyam Minuye Birihane, Zebenay Workneh Bitew, Somayeh Bohlouli, Mahdi Bohluli, Hunduma Amensisa Bojia, Archith Boloor, Oliver J Brady, Nicola Luigi Bragazzi, Andre R Brunoni, Shyam S Budhathoki, Sharath Burugina Nagaraja, Zahid A Butt, Rosario Cárdenas, Joao Mauricio Castaldelli-Maia, Franz Castro, Achille Cernigliaro, Jaykaran Charan, Pranab Chatterjee, Souranshu Chatterjee, Vijay Kumar Chattu, Sarika Chaturvedi, Mohiuddin Ahsanul Kabir Chowdhury, Dinh-Toi Chu, Michael A Cork, Baye Dagnew, Haijiang Dai, Lalit Dandona, Rakhi Dandona, Parnaz Daneshpajouhnejad, Aso Mohammad Darwesh, Amira Hamed Darwish,

Ahmad Daryani, Jai K Das, Rajat Das Gupta, Kebede Deribe, Assefa Desalew, Awrajaw Dessie, Keshab Deuba, Meghnath Dhimal, Govinda Prasad Dhungana, Daniel Diaz, Alireza Didarloo, Isaac Oluwafemi Dipeolu, Linh Phuong Doan, Bereket Duko, Laura Dwyer-Lindgren, Maysaa El Sayed Zaki, Teshome Bekele Elema, Hala Rashad Elhabashy, Pawan Sirwan Faris, Andre Faro, Farshad Farzadfar, Valery L Feigin, Berhanu Elfu Feleke, Florian Fischer, Nataliya A Foigt, Morenike Oluwatoyin Folayan, Richard Charles Franklin, Mohamed M Gad, Shilpa Gaidhane, William M Gardner, Birhan Gebresillassie Gebregiorgis, Ketema Bizuwork Gebremedhin, Berhe Gebremichael, Fariborz Ghaffarpasand, Syed Amir Gilani, Themba G Ginindza, Mustefa Glagn, Mahaveer Golechha, Dr Kebebe Bekele Gonfa, Bárbara Niegia Garcia Goulart, Nachiket Gudi, Davide Guido, Rashid Abdi Guled, Yuming Guo, Ahmed I Hasaballah, Behnam Heidari, Nathaniel J Henry, Claudiu Herteliu, Hagos Degefa de Hidru, Hung Chak Ho, Chi Linh Hoang, Ramesh Holla, Mehdi Hosseinzadeh, Mowafa Househ, Tanvir M Huda, Bing-Fang Hwang, Segun Emmanuel Ibitoye, Olayinka Stephen Ilesanmi, Irena M Ilic, Milena D Ilic, Leeberk Raja Inbaraj, Usman Iqbal, Seyed Sina Naghibi Irvani, M Mofizul Islam, Chidozie C D Iwu, Chinwe Juliana Iwu, Animesh Jain, Manthan Dilipkumar Janodia, Tahereh Javaheri, Yetunde O John-Akinola, Farahnaz Joukar, Jacek Jerzy Jozwiak, Ali Kabir, Leila R Kalankesh, Rohollah Kalhor, Ashwin Kamath, Naser Kamyari, Tanuj Kanchan, Neeti Kapoor, Behzad Karami Matin, Salah Eddin Karimi, Habtamu Kebebe Kasaye, Getinet Kassahun, Nicholas J Kassebaum, Gbenga A Kayode, Ali Kazemi Karyani, Peter Njenga Keiyoro, Bayew Kelkay, Nauman Khalid, Md Nuruzzaman Khan, Khaled Khatab, Amir M Khater, Mona M Khater, Mahalaqua Nazli Khatib, Yun Jin Kim, Ruth W Kimokoti, Damaris K Kinyoki, Adnan Kisa, Sezer Kisa, Kewal Krishan, Vaman Kulkarni, G Anil Kumar, Manasi Kumar, Nithin Kumar, Pushpendra Kumar, Om P Kurmi, Dian Kusuma, Carlo La Vecchia, Sheetal D Lad, Faris Hasan Lami, Savita Lasrado, Kate E LeGrand, Sonia Lewycka, Bingyu Li, Ming-Chieh Li, Shanshan Li, Xuefeng Liu, Rakesh Lodha, Jaifred Christian F Lopez, Daiane Borges Machado, Venkatesh Maled, Abdullah A Mamun, Navid Manafi, Mohammad Ali Mansournia, Laurie B Marczak, Francisco Rogerlândio Martins-Melo, Fabiola Mejia-Rodriguez, Tefera Chane Mekonnen, Walter Mendoza, Ritesh G Menezes, Endalkachew Worku Mengesha, Abera M Mersha, Ted R Miller, Erkin M Mirrakhimov, Sanjeev Misra, Masoud Moghadaszadeh, Dara K Mohammad, Abdollah Mohammadian-Hafshejani, Jemal Abdu Mohammed, Shafiu Mohammed, Ali H Mokdad, Pablo A Montero-Zamora, Rahmatollah Moradzadeh, Jonathan F Mosser, Amin Mousavi Khaneghah, Sandra B Munro, Moses K Muriithi, Ghulam Mustafa, Saravanan Muthupandian, Ahamarshan Jayaraman Nagarajan, Gurudatta Naik, Mukhammad David Naimzada, Vinay Nangia, Bruno Ramos Nascimento, Rawlance Ndejjo, Duduzile Edith Ndwandwe, Ionut Negoi, Georges Nguefack-Tsague, Josephine W Ngunjiri, Cuong Tat Nguyen, Diep Ngoc Nguyen, Huong Lan Thi Nguyen, Samuel Negash Nigussie, Tadesse T N Nigussie, Rajan Nikbakhsh, Chukwudi A Nnaji, Bogdan Oancea, Onome Bright Oghenetega, Andrew T Olagunju, Bolajoko Olubukunola Olusanya, Jacob Olusegun Olusanya, Muktar Omer Omer, Obinna E Onwujekwe, Doris V Ortega-Altamirano, Aaron E Osgood-Zimmerman, Nikita Otstavnov, Stanislav S Otstavnov, Mayowa O Owolabi, Mahesh P A, Jagadish Rao Padubidri, Adrian Pana, Anamika Pandey, Seithikurippu R Pandi-Perumal, Helena Ullyartha Pangaribuan, Shradha S Parsekar, Deepak Kumar Pasupula, Urvish K Patel, Ashish Pathak, Mona Pathak, Sanjay M Pattanshetty, Kebreab Paulos, Veincent Christian Filipino Pepito, Ellen G Piwoz, Khem Narayan Pokhrel, Hadi Pourjafar, Sergio I Prada, Zahiruddin Quazi Syed, Mohammad Rabiee, Navid Rabiee, Fakher Rahim, Shadi Rahimzadeh, Azizur Rahman, Mohammad Hifz Ur Rahman, Amir Masoud Rahmani, Rajesh Kumar Rai, Chhabi Lal Ranabhat, Sowmya J Rao,

Prateek Rastogi, Priya Rathi, David Laith Rawaf, Salman Rawaf, Reza Rawassizadeh, Rahul Rawat, Ramu Rawat, Lemma Demissie Regassa, Maria Albertina Santiago Rego, Robert C Reiner Jr., Bhageerathy Reshmi, Aziz Rezapour, Ana Isabel Ribeiro, Jennifer Rickard, Leonardo Roever, Godfrey M Rwegerera, Rajesh Sagar, S. Mohammad Sajadi, Marwa Rashad Salem, Abdallah M Samy, Milena M Santric-Milicevic, Sivan Yegnanarayana Iyer Saraswathy, Abdur Razzaque Sarker, Benn Sartorius, Brijesh Sathian, Alyssa N Sbarra, Debarka Sengupta, Subramanian Senthilkumaran, Omid Shafaat, Amira A Shaheen, Masood Ali Shaikh, Ali S Shalash, Mohammed Shannawaz, Aziz Sheikh, Ranjitha S Shetty, Kenji Shibuya, Wondimeneh Shibabaw Shiferaw, Jae Il Shin, Diego Augusto Santos Silva, Narinder Pal Singh, Yitagesu Sintayehu, Valentin Yurievich Skryabin, Anna Aleksandrovna Skryabina, Shahin Soltani, Muluken Bekele Sorrie, Emma Elizabeth Spurlock, Agus Sudaryanto, Mu'awiyyah Babale Sufiyan, Eyayou Girma Tadesse, Animut Tagele Tamiru, Md Ismail Tareque, Ingan Ukur Tarigan, Getayeneh Antehunegn Tesema, Abinet Teshome, Zemenu Tadesse Tessema, Kavumpurathu Raman Thankappan, Rekha Thapar, Nihal Thomas, Roman Topor-Madry, Marcos Roberto Tovani-Palone, Eugenio Traini, Bach Xuan Tran, Phuong N Truong, Berhan Tsegaye BT Tsegaye, Irfan Ullah, Chukwuma David Umeokonkwo, Bhaskaran Unnikrishnan, Era Upadhyay, Francesco S Violante, Bay Vo, Yohannes Dibaba Wado, Yasir Waheed, Richard G Wamai, Yafeng Wang, Yuan-Pang Wang, Nuwan Darshana Wickramasinghe, Kirsten E Wiens, Charles Shey Wiysonge, Ai-Min Wu, Chenkai Wu, Tomohide Yamada, Alex Yeshaneh, Yordanos Gizachew Yeshitila, Mekdes Tigistu Yilma, Paul Yip, Naohiro Yonemoto, Tewodros Yosef, Mustafa Z Younis, Abdilahi Yousuf Yousuf, Chuanhua Yu, Yong Yu, Deniz Yuce, Shamsa Zafar, Leila Zaki, Maryam Zamanian, Mikhail Sergeevich Zastrozhin, Anasthasia Zastrozhina, Desalege Amare Zelellw, Yunquan Zhang, Zhi-Jiang Zhang, Sanjay Zodpey, Yves Miel H Zuniga, and Simon I Hay

Drafting the work or revising is critically for important intellectual content

Natalia V Bhattacharjee, Lauren E Schaeffer, Megan F Schipp, Katie M Donkers, Gdiom Gebreheat Abady, Foad Abd-Allah, Ahmed Abdelalim, Zeleke Hailemariam Abebo, Ayenew Negesse Abejie, Lucas Guimarães Abreu, Eman Abu-Gharbieh, Abdelrahman I Abushouk, Aishatu L Adamu, Isaac Akinkunmi Adedeji, Victor Adekanmbi, Olatunji O Adetokunboh, Marcela Agudelo-Botero, Budi Aji, Oluwaseun Oladapo Akinyemi, Jacqueline Elizabeth Alcalde-Rabanal, Robert Kaba Alhassan, Cyrus Alinia, Dickson A Amugsi, Robert Ancuceanu, Mina Anjomshoa, Carl Abelardo T Antonio, Jalal Arabloo, Amit Arora, Marcel Ausloos, Nefsu Awoke, Martin Amogre Ayanore, Darshan B B, Atif Amin Baig, Shankar M Bakkannavar, Maciej Banach, Till Winfried Bärnighausen, Huda Basaleem, Mohsen Bayati, Neeraj Bedi, Akshaya Srikanth Bhagavathula, Dinesh Bhandari, Oliver J Brady, Nicola Luigi Bragazzi, Andre R Brunoni, Shyam S Budhathoki, Sharath Burugina Nagaraja, Joao Mauricio Castaldelli-Maia, Franz Castro, Achille Cernigliaro, Pranab Chatterjee, Vijay Kumar Chattu, Sarika Chaturvedi, Dinh-Toi Chu, Baye Dagnew, Ahmad Daryani, Rajat Das Gupta, Claudio Alberto Dávila-Cervantes, Nicole Davis Weaver, Edgar Denova-Gutiérrez, Kebede Deribe, Samath Dhamminda Dharmaratne, Meghnath Dhimal, Govinda Prasad Dhungana, Daniel Diaz, Alireza Didarloo, Isaac Oluwafemi Dipeolu, Linh Phuong Doan, Andre Rodrigues Duraes, Laura Dwyer-Lindgren, Maysaa El Sayed Zaki, Maha El Tantawi, Shaimaa I El-Jaafary, Andre Faro, Berhanu Elfu Feleke, Tomas Y Ferede, Florian Fischer, Nataliya A Foigt, Morenike Oluwatoyin Folayan, Mohamed M Gad, Shilpa Gaidhane, Biniyam Sahiledengle Geberemariyam, Berhe Gebremichael, Themba G Ginindza, Mustefa Glagn, Bárbara Niegia Garcia Goulart, Nachiket

Gudi, Yuming Guo, Samer Hamidi, Demelash Woldeyohannes Handiso, Ahmed I Hasaballah, Amr Hassan, Khezar Hayat, Claudiu Herteliu, Hagos Degefa de Hidru, Chi Linh Hoang, Ramesh Holla, Mostafa Hosseini, Mehdi Hosseinzadeh, Mowafa Househ, Guoqing Hu, Segun Emmanuel Ibitoye, Olayinka Stephen Ilesanmi, Irena M Ilic, Milena D Ilic, Usman Iqbal, Seyed Sina Naghibi Irvani, M Mofizul Islam, Chidozie C D Iwu, Chinwe Juliana Iwu, Animesh Jain, Manthan Dilipkumar Janodia, Yetunde O John-Akinola, Kimberly B Johnson, Jacek Jerzy Jozwiak, Ali Kabir, Habtamu Kebebe Kasaye, Getinet Kassahun, Nicholas J Kassebaum, Gbenga A Kayode, Bayew Kelkay, Md Nuruzzaman Khan, Khaled Khatab, Mona M Khater, Mahalaqua Nazli Khatib, Yun Jin Kim, Adnan Kisa, Sezer Kisa, Kewal Krishan, Vaman Kulkarni, Manasi Kumar, Om P Kurmi, Dian Kusuma, Carlo La Vecchia, Iván Landires, Savita Lasrado, Paul H Lee, Kate E LeGrand, Shanshan Li, Jaifred Christian F Lopez, Daiane Borges Machado, Venkatesh Maled, Shokofeh Maleki, Abdullah A Mamun, Navid Manafi, Mohammad Ali Mansournia, Chabila Christopher Mapoma, Laurie B Marczak, Francisco Rogerlândio Martins-Melo, Tefera Chane Mekonnen, Ted R Miller, GK Mini, Masoud Moghadaszadeh, Dara K Mohammad, Jemal Abdu Mohammed, Shafiu Mohammed, Ali H Mokdad, Pablo A Montero-Zamora, Paula Moraga, Jonathan F Mosser, Seyyed Meysam Mousavi, Amin Mousavi Khaneghah, Ghulam Mustafa, Ahamarshan Jayaraman Nagarajan, Mukhammad David Naimzada, Vinay Nangia, Bruno Ramos Nascimento, Vinod C Nayak, Ionut Negoi, Georges Nguefack-Tsague, Josephine W Ngunjiri, Cuong Tat Nguyen, Huong Lan Thi Nguyen, Samuel Negash Nigussie, Tadesse T N Nigussie, Rajan Nikbakhsh, Virginia Nunez-Samudio, Onome Bright Oghenetega, Andrew T Olagunju, Bolajoko Olubukunola Olusanya, Jacob Olusegun Olusanya, Muktar Omer Omer, Obinna E Onwujekwe, Doris V Ortega-Altamirano, Nikita Otstavnov, Stanislav S Otstavnov, Mayowa O Owolabi, Mahesh P A, Jagadish Rao Padubidri, Adrian Pana, Seithikurippu R Pandi-Perumal, Mona Pathak, George C Patton, Veincent Christian Filipino Pepito, Khem Narayan Pokhrel, Sergio I Prada, Zahiruddin Quazi Syed, Fakher Rahim, Azizur Rahman, Mohammad Hifz Ur Rahman, Amir Masoud Rahmani, Chhabi Lal Ranabhat, Sowmya J Rao, Priya Rathi, David Laith Rawaf, Salman Rawaf, Reza Rawassizadeh, Lemma Demissie Regassa, Robert C Reiner Jr., Bhageerathy Reshmi, Ana Isabel Ribeiro, Jennifer Rickard, Leonardo Roever, Susan Fred Rumisha, Godfrey M Rwegerera, Rajesh Sagar, Marwa Rashad Salem, Abdallah M Samy, Milena M Santric-Milicevic, Deepak Saxena, Masood Ali Shaikh, Mohammed Shannawaz, Diego Augusto Santos Silva, Pushpendra Singh, Surya Singh, Yitagesu Sintayehu, Muluken Bekele Sorrie, Mu'awiyyah Babale Sufiyan, Eyayou Girma Tadesse, Animut Tagele Tamiru, Leili Tapak, Fisaha Haile Tesfay, Nihal Thomas, Marcos Roberto Tovani-Palone, Bach Xuan Tran, Berhan Tsegaye BT Tsegaye, Irfan Ullah, Chukwuma David Umeokonkwo, Bhaskaran Unnikrishnan, Era Upadhyay, Benjamin S Chudi Uzochukwu, Francesco S Violante, Bay Vo, Yasir Waheed, Richard G Wamai, Fang Wang, Yuan-Pang Wang, Nuwan Darshana Wickramasinghe, Kirsten E Wiens, Charles Shey Wiysonge, Ai-Min Wu, Sanni Yaya, Alex Yeshaneh, Yigizie Yeshaw, Yordanos Gizachew Yeshitila, Mekdes Tigistu Yilma, Naohiro Yonemoto, Deniz Yuce, Maryam Zamanian, Heather J Zar, Mikhail Sergeevich Zastrozhin, Anasthasia Zastrozhina, Xiu-Ju George Zhao, and Simon I Hay

Managing the overall research enterprise

Aubrey J Cook, Lalit Dandona, Deborah Carvalho Malta, Ali H Mokdad, George C Patton, Robert C Reiner Jr., Benn Sartorius, Roman Topor-Madry, and Simon I Hay.

10.0. Supplementary References

1. Murray, C. J. L. *et al.* Global burden of 87 risk factors in 204 countries and territories, 1990–2019: a systematic analysis for the Global Burden of Disease Study 2019. *The Lancet* **396**, 1223–1249 (2020).
2. Bhattacharjee, N. V. *et al.* Mapping exclusive breastfeeding in Africa between 2000 and 2017. *Nature Medicine* **25**, 1205–1212 (2019).
3. Wiegand, H. Kish, L.: Survey Sampling. John Wiley & Sons, Inc., New York, London 1965, IX + 643 S., 31 Abb., 56 Tab., Preis 83 s. *Biometrische Zeitschrift* **10**, 88–89 (1968).
4. Golding, N. *et al.* Mapping under-5 and neonatal mortality in Africa, 2000-15: a baseline analysis for the Sustainable Development Goals. *The Lancet* **390**, 2171–2182 (2017).
5. Fullman, N. *et al.* Measuring performance on the Healthcare Access and Quality Index for 195 countries and territories and selected subnational locations: a systematic analysis from the Global Burden of Disease Study 2016. *The Lancet* **391**, 2236–2271 (2018).
6. Lozano, R. *et al.* Measuring progress from 1990 to 2017 and projecting attainment to 2030 of the health-related Sustainable Development Goals for 195 countries and territories: a systematic analysis for the Global Burden of Disease Study 2017. *The Lancet* **392**, 2091–2138 (2018).
7. Development Initiatives. *2018 Global Nutrition Report: Shining a light to spur action on nutrition*. https://globalnutritionreport.org/reports/global-nutrition-report-2018/ (2018).
8. UNICEF. Exclusive breastfeeding (<6 months) dataset. *Infant and young child feeding (IYCF) data* https://data.unicef.org/resources/dataset/infant-young-child-feeding/.
9. Perez-Escamilla, R. Update on the breastfeeding situation in Africa. *Nutrition Research* **13**, 597–609 (1993).
10. Shirima, R., Greiner, T., Kylberg, E. & Gebre-Medhin, M. Exclusive breast-feeding is rarely practised in rural and urban Morogoro, Tanzania. *Public Health Nutrition* **4**, 147–154 (2001).
11. Shirima, R., Gebre-Medhin, M. & Greiner, T. Information and socioeconomic factors associated with early breastfeeding practices in rural and urban Morogoro, Tanzania. *Acta Paediatrica* **90**, 936–942 (2001).
12. Victora, C. G. *et al.* Breastfeeding in the 21st century: epidemiology, mechanisms, and lifelong effect. *The Lancet* **387**, 475–490 (2016).
13. Cernadas, J. M. C., Noceda, G., Barrera, L., Martinez, A. M. & Garsd, A. Maternal and Perinatal Factors Influencing the Duration of Exclusive Breastfeeding During the First 6 Months of Life. *J Hum Lact* **19**, 136–144 (2003).
14. Coutinho, S. B., de Lira, P. I. C., de Carvalho Lima, M. & Ashworth, A. Comparison of the effect of two systems for the promotion of exclusive breastfeeding. *The Lancet* **366**, 1094–1100 (2005).
15. Lartey, A. Maternal and child nutrition in Sub-Saharan Africa: challenges and interventions. *Proceedings of the Nutrition Society* **67**, 105–108 (2008).
16. Wray, J. D. Maternal Nutrition, Breast-Feeding and Infant Survival. in *Nutrition and Human Reproduction* (ed. Mosley, W. H.) 197–229 (Springer US, 1978). doi:10.1007/978-1-4684-0790-7_12.

17. Hossain, M., Islam, A., Kamarul, T. & Hossain, G. Exclusive breastfeeding practice during first six months of an infant's life in Bangladesh: a country based cross-sectional study. *BMC Pediatrics* **18**, 93 (2018).
18. ODPHP. Maternal, Infant, and Child Health | Healthy People 2020. https://www.healthypeople.gov/2020/topics-objectives/topic/maternal-infant-and-child-health.
19. HHS, Office of the Surgeon General. Breastfeeding Reports And Publications. *HHS.gov* https://www.hhs.gov/surgeongeneral/reports-and-publications/breastfeeding/index.html (2019).
20. Biks, G. A., Tariku, A. & Tessema, G. A. Effects of antenatal care and institutional delivery on exclusive breastfeeding practice in northwest Ethiopia: a nested case–control study. *Int Breastfeed J* **10**, (2015).
21. Doherty, T. *et al.* Effectiveness of the WHO/UNICEF guidelines on infant feeding for HIV-positive women: results from a prospective cohort study in South Africa. *AIDS* **21**, 1791 (2007).
22. Thairu, L. N., Pelto, G. H., Rollins, N. C., Bland, R. M. & Ntshangase, N. Sociocultural influences on infant feeding decisions among HIV-infected women in rural Kwa-Zulu Natal, South Africa. *Maternal & Child Nutrition* **1**, 2–10 (2005).
23. Bland, R. M. *et al.* Intervention to promote exclusive breast-feeding for the first 6 months of life in a high HIV prevalence area. *AIDS* **22**, 883 (2008).
24. Bland, R. M., Rollins, N. C., Coutsoudis, A. & Coovadia, H. M. Breastfeeding practices in an area of high HIV prevalence in rural South Africa. *Acta Paediatrica* **91**, 704–711 (2002).
25. Faraway, J. J. Chapter 4: Problems with the predictors. in *Linear Models with R* (CRC Press, 2004).
26. WorldPop. WorldPop dataset. http://www.worldpop.org.uk/data/get_data/.
27. Tatem, A. J. WorldPop, open data for spatial demography. *Scientific Data* **4**, 170004 (2017).
28. Global Administrative Areas (GADM). GADM Database of Global Adminstrative Areas. http://www.gadm.org (2018).
29. Land Processes Distributed Active Archive Center. Combined MODIS 5.1. *MCD12Q1 | LP DAAC :: NASA Land Data Products and Services*.
30. World Wildlife Fund. Global Lakes and Wetlands Database, Level 3. https://www.worldwildlife.org/pages/global-lakes-and-wetlands-database (2004).
31. Lehner, B. & Döll, P. Development and validation of a global database of lakes, reservoirs and wetlands. *Journal of Hydrology* **296**, 1–22 (2004).
32. Breiman, L. Stacked regressions. *Mach Learn* **24**, 49–64 (1996).
33. Bhatt, S. *et al.* Improved prediction accuracy for disease risk mapping using Gaussian process stacked generalization. *Journal of The Royal Society Interface* **14**, 1–10 (2017).
34. Kinyoki, D. K. *et al.* Mapping child growth failure across low- and middle-income countries. *Nature* **577**, 231–234 (2020).
35. Kinyoki, D. K. *et al.* Mapping local patterns of childhood overweight and wasting in low- and middle-income countries between 2000 and 2017. *Nature Medicine* **26**, 750–759 (2020).
36. Graetz, N. *et al.* Mapping disparities in education across low- and middle-income countries. *Nature* **577**, 235–238 (2020).
37. Burstein, R. *et al.* Mapping 123 million neonatal, infant and child deaths between 2000 and 2017. *Nature* **574**, 353–358 (2019).

38. Reiner, Jr., R. C. *et al.* Mapping geographical inequalities in childhood diarrhoeal morbidity and mortality in low-income and middle-income countries, 2000–17: analysis for the Global Burden of Disease Study 2017. *The Lancet* **395**, 1779–1801 (2020).
39. Bhutta, Z. A. *et al.* Interventions to address deaths from childhood pneumonia and diarrhoea equitably: what works and at what cost? *Lancet* **381**, 1417–1429 (2013).
40. Murray, C. J. *et al.* GBD 2010: design, definitions, and metrics. *The Lancet* **380**, 2063–2066 (2012).
41. Stein, M. L. *Interpolation of spatial data: some theory for kriging.* (Springer, 1999).
42. Rue, H., Martino, S. & Chopin, N. Approximate Bayesian inference for latent Gaussian models by using integrated nested Laplace approximations (with discussion). *J Roy Stat Soc B* **71**, 319–392 (2009).
43. Lindgren, F., Rue, H. & Lindström, J. An explicit link between Gaussian fields and Gaussian Markov random fields: the stochastic partial differential equation approach. *Journal of the Royal Statistical Society: Series B (Statistical Methodology)* **73**, 423–498 (2011).
44. WHO & UNICEF. Global Breastfeeding Collective Scorecard Data. *Tableau Software* https://public.tableau.com/profile/gbc2364#!/vizhome/Tables2/Dashboard1?publish=yes (2018).
45. Wiens, K. E. *et al.* Mapping geographical inequalities in oral rehydration therapy coverage in low-income and middle-income countries, 2000–17. *The Lancet Global Health* **8**, e1038–e1060 (2020).
46. Deshpande, A. *et al.* Mapping geographical inequalities in access to drinking water and sanitation facilities in low-income and middle-income countries, 2000–17. *The Lancet Global Health* **8**, e1162–e1185 (2020).
47. Pullum, T. W. Exclusive breastfeeding: aligning the indicator with the goal. *Glob Health Sci Pract* **2**, 355–356 (2014).
48. Piwoz, E. G. *et al.* Potential for Misclassification of Infants' Usual Feeding Practices using 24-Hour Dietary Assessment Methods. *J Nutr* **125**, 57–65 (1995).
49. Aarts, C. *et al.* How exclusive is exclusive breastfeeding? A comparison of data since birth with current status data. *Int J Epidemiol* **29**, 1041–1046 (2000).
50. Gibson, R., Charrondiere, U. & Bell, W. Measurement errors in dietary assessment using self-reported 24-hour recalls in low-income countries and strategies for their prevention. *Advances in Nutrition* **8**, 980–991 (2017).
51. Bauchner, H., Leventhal, J. M. & Shapiro, E. D. Studies of breast-feeding and infections. How good is the evidence? *JAMA* **256**, 887–892 (1986).
52. Burgert, C. R., Colston, J., Roy, T. & Zachary, B. *Geographic displacement procedure and georeferenced data release policy for the Demographic and Health Surveys*. https://www.dhsprogram.com/publications/publication-SAR7-Spatial-Analysis-Reports.cfm (2013).
53. Perez-Escamilla, R. Breastfeeding in Africa and the Latin American and Caribbean Region: the Potential Role of Urbanization. *J Trop Pediatr* **40**, 137–143 (1994).
54. Lugina, H. I. Breastfeeding commitments and challenges in Africa. *African Journal of Midwifery and Women's Health* **5**, 4–4 (2011).
55. Kavle, J., LaCroix, E., Dau, H. & Engmann, C. Addressing barriers to exclusive breast-feeding in low- and middle-income countries: a systematic review and programmatic implications. *Public Health Nutrition* **20**, 3120–3134 (2017).

56. UNICEF. *From the First Hour of Life: Making the case for improved infant and young child feeding everywhere*. https://www.unicef.org/publications/index_93027.html (2016).
57. UNICEF. *A successful start in life: Improving breastfeeding in West and Central Africa*. https://www.unicef.org/health/files/wcaro_improving_breastfeeding_en.pdf (2010).
58. Rollins, N. C. *et al.* Why invest, and what it will take to improve breastfeeding practices? *The Lancet* **387**, 491–504 (2016).
59. Sinha, B. *et al.* Interventions to improve breastfeeding outcomes: a systematic review and meta-analysis. *Acta Paediatrica* **104**, 114–134 (2015).
