# Supplementary material for: Mapping inequalities in exclusive breastfeeding in low- and middle-income countries, 2000–2018
Source: Nat Hum Behav. 2021 Jun 3;5(8):1027–45. doi: 10.1038/s41562-021-01108-6 (PMC8373614; doi:10.1038/s41562-021-01108-6)
Supplement: Supplementary file 3 — Peer review information [file 41562_2021_1108_MOESM3_ESM.pdf]

Peer Review Information

Journal: Nature Human Behaviour
Manuscript Title: Mapping inequalities in exclusive breastfeeding in low- and middle- income countries, 2000–2018
Corresponding author name(s): Simon I. Hay

Editorial Notes:

Reviewer Comments & Decisions:

Decision Letter, initial version:

12th October 2020

Dear Professor Hay,

Thank you once again for your manuscript, entitled "Mapping geographic inequalities in exclusive breastfeeding prevalence in low- and middle-income countries between 2000 and 2018." Please accept my sincere apologies once again for the extraordinary delay in contacting you with a decision on your manuscript - unfortunately, your manuscript underwent multiple reviewer replacements, which significantly delayed the decision process.

Your manuscript has now been evaluated by 3 reviewers, whose comments are included at the end of this letter. As you can see, all reviewers are positive towards your work, while at the same time making important suggestions on how to further strengthen it for publication. We are very interested in the possibility of publishing your study in Nature Human Behaviour, but would like to consider your response to the reviewers' feedback in the form of a revised manuscript before we make a decision on publication.

Before resubmission, please make sure that your revised manuscript complies with our editorial policies and formatting requirements. To assist you in this process, I have attached a checklist that lists all of our requirements. If you have any questions about any of our policies or formatting, please don't hesitate to contact me.

In sum, we invite you to revise your manuscript taking into account all reviewer comments. We are committed to providing a fair and constructive peer-review process. Do not hesitate to contact us if

there are specific requests from the reviewers that you believe are technically impossible or unlikely to yield a meaningful outcome.

We hope to receive your revised manuscript within three months. We understand that the COVID-19 pandemic is causing significant disruption for many of our authors and reviewers. If you cannot send your revised manuscript within this time, please let us know - we will be happy to extend the submission date to enable you to complete your work on the revision.

With your revision, please:

• Include a “Response to the editors and reviewers” document detailing, point-by-point, how you addressed each referee comment. If no action was taken to address a point, you must provide a compelling argument. This response will be used by the editors to evaluate your revision and sent back to the reviewers along with the revised manuscript.

• Highlight all changes made to your manuscript or provide us with a version that tracks changes.

Please use the link below to submit your revised manuscript and related files:

[REDACTED]

Note: This URL links to your confidential home page and associated information about manuscripts you may have submitted, or that you are reviewing for us. If you wish to forward this email to co-authors, please delete the link to your homepage.

We look forward to seeing the revised manuscript and thank you for the opportunity to review your work. Please do not hesitate to contact me if you have any questions or would like to discuss these revisions further.

Sincerely,

Stavroula Kousta

Stavroula Kousta, PhD
Chief Editor
Nature Human Behaviour

Reviewer expertise:

Reviewer #1: breastfeeding in LMICs

Reviewer #2: breastfeeding in LMICs

Reviewer #3: Bayesian geospatial modelling

REVIEWER COMMENTS:

Reviewer #1:
Remarks to the Author:
The authors conducted a geospatial analysis of exclusive breastfeeding prevalence estimates from 2000 to 2018 across 94 LMICs mapped to policy-relevant administrative units (e.g., districts), quantify subnational inequalities and their changes over time, and estimate probabilities of meeting the World Health Organization′s Global Nutrition Target (WHO GNT) of ≥70% EBF prevalence by 2030. This is an extensive analysis. However, I have the following comments:
It is critical to know the subnational inequalities of EBF rates in LMICs. However, the possible reasons for these subnational inequalities are equally important to know for the policymakers. EBF is a culture that different communities perceive in different ways. Unless we know the possible reasons, it will be difficult for policymakers to implement effective interventions to increase EBF. For example, few common barriers of sub-optimal breastfeeding practices in LMICs are baby too weak to suck, mothers' perceived inadequacy of breast milk, and breast problems. How do these barriers vary in different subnational levels; is an important question that policymakers would be interested in. The information generated from this analysis will definitely help the policymakers but I would suggest the authors should discuss the implications of the findings from a public health perspective.

Reviewer #2:
Remarks to the Author:
The paper brings significant results to one of the most important habits that can promote well-being and lifelong effects for child health, especially in LMICs. The authors used sophisticated statistical methods that allowed a detailed geographical analysis at country level. Additionally, potential uncertainties with the models you dealt with were well acknowledged.

I have minor comments to add. Despite its strengths in mapping EBF rates in LMICs, the paper has a tremendous amount of supplemental material that readers will hardly access entirely, in my opinion. Moreover, there are so many results provided that it is easy to one get confused amid all that material. I think the readers will find it difficult to understand about the methods and analysis executed based on so many results.

I was wondering why you did not use standard regional classifications of the world, like the UNICEF's or WHO's ones, in order to best place readers on the global perspective you proposed in this work.

Although you were very fortunate in showing district- and province-level estimates, I find myself confused with the results of declines for some countries, like Brazil. I recently had the opportunity to read a paper by Boccolini et al. (Rev Saude Publ, 2017) that showed increases in EBF prevalence at the national level in Brazil. What I think you've found was the reverse, right? Did I get the message wrongly, or are there some issues with the data?

One limitation of your work in predicting EBF trajectories by 2025/2030 is that you relied on older

findings from some countries. Some of them do not have a recent survey conducted from 2010 onwards, for instance, which affects your estimates significantly. I think this kind of limitation should be placed as a limitation.

Another issue, in my opinion, is with the spatial covariates used in your models. Some of them did not seem well suited for this kind of analysis, like the (6) number of people whose daily vitamin A needs could be met. Many countries do not follow a nationwide vitamin A supplementation scheme, and the supplementation is recommended only for children older than 6 months of age. How do these spatial covariates correlate with EBF rates over the period?

One last concern is that EBF is strongly influenced by policies and programs towards improvements in BF rates. Have you considered including this kind of variables in your models?

Reviewer #3:
Remarks to the Author:
This is an impressive work from Hay's group and collaborators, and as usual rich in details and open information. The methods are coherent with the project objectives. Many congratulations to the authors for this massive work.

I have only few points that the authors are invited to consider in reviewing the manuscript.

The main one is around uncertainty. The uncertainty is not played well, but actually there is a lot of uncertainty in the results (Extended data Figure 3). I will suggest to add a section in the manuscript describing the different uncertainties and ideally linking it with Supplementary Figure 1 to 5 where some of the countries are really lacking in data. In addition, a section on uncertainty could clear the results, where in my view some statements does not take into account uncertainty (for example when describing EBF in South Africa).

Other minor points.

1) I like the model validation approach. Please can you provide more details on the out-of-sample. Was it balanced, e.g. showing a good proportion of both low and high values?

2) In post estimation (page 17) the authors stated "We used absolute differences between lowest and highest units and relative differences between a country's average and each unit in that country to quantify geographic inequality." I assume these are the modelled ones, aren't they?

3) Modelling limitations section. I think in reality you can include a certain amount of uncertainty (unstructured) just by working on your priors (for example comparing with non-informative priors and setting them with large boundaries).

4) In extended data figure 2 it is not clear how the uncertainty reflects the data heterogeneity. For example, in BFA the uncertainty is very low when you have an "other survey" polygon. I would have expected a large uncertainty there because the other survey is almost an outlier. The same for BRA, YEM etc… Or may be my interpretation is wrong. Please clarify.

5) In Extended Data Figure 3, I can understand with low prevalence and sparse data you will have a large uncertainty. I wonder if the results need to be described in terms of what is accurate and what is not. May be this can be simply covered in a new section of uncertainty (as advised above).

6)Not sure what “not extracted” means in Supplementary Table 1. Why is not extracted?

7)The resampled data matched to polygons need more explanations. It is not clear how a reduced set of pseudo-location is generated. In particular on the final number of pseudo-locations. In fact, in geostatistics it is more important the number and geometry of the points instead of their value.

Author Rebuttal to Initial comments

24 December, 2020

Nature Human Behaviour

Dear Reviewers,

Thank you for the opportunity to revise our manuscript “Mapping geographic inequalities in exclusive breastfeeding prevalence in low- and middle-income countries between 2000 and 2018” (MS ID: 200510750) for *Nature Human Behaviour*. We are thankful for the reviewers’ comments and believe that the revised manuscript is stronger as a result of this feedback.

In response to the suggestions provided by reviewers, we have revised our manuscript and supplementary materials.

Apologies for the slight delay in the return of these materials; this year has been a challenging one for us all in so many ways.

We hope these revisions are satisfactory and look forward to your assessment.

With very best wishes,

Natalia V. Bhattacharjee, PhD
Lauren E. Schaeffer, MSc
Simon I. Hay, DPhil, DSc, FMedSci

natureresearch

Institute for Health Metrics and Evaluation
2301 5th Ave, Suite 600
Seattle, WA 98121

Referees' comments:

Referee #1 (expertise – breastfeeding in LMICs):

Remarks to the Author:
The authors conducted a geospatial analysis of exclusive breastfeeding prevalence estimates from 2000 to 2018 across 94 LMICs mapped to policy-relevant administrative units (e.g., districts), quantify subnational inequalities and their changes over time, and estimate probabilities of meeting the World Health Organization's Global Nutrition Target (WHO GNT) of ≥70% EBF prevalence by 2030. This is an extensive analysis. However, I have the following comments:

It is critical to know the subnational inequalities of EBF rates in LMICs. However, the possible reasons for these subnational inequalities are equally important to know for the policymakers. EBF is a culture that different communities perceive in different ways. Unless we know the possible reasons, it will be difficult for policymakers to implement effective interventions to increase EBF. For example, few common barriers of sub-optimal breastfeeding practices in LMICs are baby too weak to suck, mothers' perceived inadequacy of breast milk, and breast problems. How do these barriers vary in different subnational levels; is an important question that policymakers would be interested in. The information generated from this analysis will definitely help the policymakers but I would suggest the authors should discuss the implications of the findings from a public health perspective.

Response:
Thank you for your comment. We have added paragraphs to the Supplementary Discussion to include examples of cultural perceptions and customs as barriers to EBF, and note that these underlying drivers of subnational inequalities of EBF need to be further investigated to plan effective intervention strategies locally.

> *"Additional barriers to EBF include cultural perceptions and generational feeding practices, which can be highly variable across communities. Mothers who perceive their breast milk to be insufficient or nutritionally inadequate are more likely to discontinue practice of EBF[1]. Infant cues when feeding (such as fussiness and crying) and problems when breastfeeding (such as breast pain or engorgement, or problems latching) are commonly cited*

barriers to EBF[1]. A common misconception and practice is the discarding of mothers' early breast milk (colostrum), which has important protective properties for infants, as it is perceived to be sour and difficult to digest[2–4]. This instead is replaced by prelacteal feeding of water, formula, or animal milk, and makes establishing breastfeeding difficult[1,3,4]. Some cultural practices involve feeding newborns water, sugar water, tea, honey, butter, animal milk, or porridges before they are fed at the breast, or during their first few months of life[2,3]. Breastfeeding counselling to increase maternal knowledge on the importance of EBF and provide lactation support can help counteract these barriers[2,1]. Fathers and grandparents can influence a woman's decision to breastfeed[2,3,5], whereas positive encouragement from family and sharing of household responsibilities increases the likelihood mothers will continue breastfeeding for the newborn's first six months[1,2]."

Referee #2 (expertise – breastfeeding in LMICs):

Remarks to the Author:

The paper brings significant results to one of the most important habits that can promote well-being and lifelong effects for child health, especially in LMICs. The authors used sophisticated statistical methods that allowed a detailed geographical analysis at country level. Additionally, potential uncertainties with the models you dealt with were well acknowledged.

I have minor comments to add. Despite its strengths in mapping EBF rates in LMICs, the paper has a tremendous amount of supplemental material that readers will hardly access entirely, in my opinion. Moreover, there are so many results provided that it is easy to one get confused amid all that material. I think the readers will find it difficult to understand about the methods and analysis executed based on so many results.

Response:

Thank you for your comment. Given the complicated nature of our modelling efforts, we wanted to include a more thorough explanation and defense of our methods in the Supplemental material. We acknowledge that most readers will not read the Supplemental material in its entirety, but we intend for it to be read by those who wish for a more thorough explanation of the methods, and to provide

additional transparency of our data sources, data availability, covariates, and uncertainties. These will be of particular value it is hoped for those that may wish to reproduce and improve on this work. We have provided a more succinct version in the online Methods of the main paper.

As for our results, we wanted to present a comprehensive study that included not only exclusive breastfeeding (EBF) rates by year, but also annualised change in these rates over time (2000-2018), within-country inequalities of EBF rates, estimates of number of infants who are not exclusively breastfed, and projections of likelihood to meet the WHO Global Nutrition Target set for EBF by the year 2030. We also wanted to present the same kind of results as we have across our body of work mapping conditions and risk factors at the administrative-unit level—including our studies estimating infant mortality[6], diarrhea[7], oral rehydration solution usage[8], and child growth failure indicators[9]. This would allow for policy makers and relevant stakeholders to analyze subnational prevalence and trends across conditions to determine which administrative areas are most in need of additional resources, interventions, and/or policy change, especially in resource-strapped countries.

I was wondering why you did not use standard regional classifications of the world, like the UNICEF's or WHO's ones, in order to best place readers on the global perspective you proposed in this work.

Response:
For this study, we used 14 geographically distinct modelling regions as determined by the Global Burden of Disease (GBD) study[10], which were based on epidemiological homogeneity and geographical contiguity. This study is part of a body of work we call Local Burden of Disease (LBD) results, wherein we mapped diseases, conditions, and risk factors at a 5 x 5-km level using geolocated data, and then aggregated resulting estimates to policy-relevant administrative-level units for public health decision makers. We use the same modelling regions across the Institute for Health Metrics and Evaluation (IHME) studies (including the GBD and LBD studies) in order to have comparable results, wherein policy makers, public health program planners, and relevant stakeholders could compare across our results across our modelling regions.

We have edited the following sentence in the Main Text introduction:
"We used 14 geographically distinct modelling regions which were determined based on epidemiological homogeneity and geographical contiguity by the Global Burden of Disease study[10] (Supplementary Table 4 and Supplementary Figure 7)."

Although you were very fortunate in showing district- and province-level estimates, I find myself confused with the results of declines for some countries, like Brazil. I recently had the opportunity to read a paper by Boccolini et al. (Rev Saude Publ, 2017) that showed increases in EBF prevalence at the national level in Brazil. What I think you've found was the reverse, right? Did I get the message wrongly, or are there some issues with the data?

<u>Response:</u>
Thank you for bringing this reference to our attention. Please note that our study period is 2000–2018 wherein Boccolini *et al*. 2017 looked at a 1986–2013 period, wherein a slight increase of EBF in Brazil occurred from 1986 to 2006 and then became steady after 2006. Therefore, our national estimates are not different from EBF trends per Boccolini *et al*. 2017. Our time series show a slight increase in national levels of EBF in Brazil during the 2000–2006 period and afterwards became steady. Data and our model also suggest, however, that Brazil had both provinces with increases and decreases in EBF levels, demonstrating inequalities in EBF practice within the country. Western provinces had the highest and eastern provinces had the lowest EBF prevalence levels between 2000 and 2018.

One limitation of your work in predicting EBF trajectories by 2025/2030 is that you relied on older findings from some countries. Some of them do not have a recent survey conducted from 2010 onwards, for instance, which affects your estimates significantly. I think this kind of limitation should be placed as a limitation.

<u>Response:</u>
Thank you for your comment. While we had included an explanation of this limitation in the Supplementary Information and a brief statement in the online Methods, we have added to this explanation in the 'Modelling Limitations' section of the online Methods:

"To estimate projections of EBF prevalence levels in 2025 and 2030, we used previous historical trends and the assumption that recent trends will

continue. These assumptions in turn lend to modelling limitations, as we were not able to project underlying drivers of changes in EBF, such as increasing urbanization or changes in population[11–13]*, and the certainty of our estimates and projections were critically dependent on data quality and availability. Availability of relevant data varies both spatially and temporally across LMICs (Supplementary Figures 1–5), and lack of relevant data is one of the main sources of uncertainty around our estimates (as seen in Extended Data Figure 3). We have mapped EBF prevalence levels against the relative uncertainty of our estimates in Extended Data Figure 3."*

Another issue, in my opinion, is with the spatial covariates used in your models. Some of them did not seem well suited for this kind of analysis, like the (6) number of people whose daily vitamin A needs could be met. Many countries do not follow a nationwide vitamin A supplementation scheme, and the supplementation is recommended only for children older than 6 months of age. How do these spatial covariates correlate with EBF rates over the period?

Response:

Thank you for your comment. The model maximizes its prediction by utilizing covariates that are related in some way to EBF; thus, it provides information where direct data are limited. In other words, the spatial covariates were selected because they are factors or proxies for factors that previous literature has identified to be associated (not necessarily causally) with exclusive breastfeeding prevalence. Our model is fairly flexible and we do not pre-specify the strength/direction of the relationship between covariates and EBF. "Number of people whose daily vitamin A needs could be met" was chosen as a proxy for maternal nutrition while breastfeeding[14,15]. If there is no association between the covariate of "number of people whose daily vitamin A needs could be met" and EBF prevalence in a particular area of a country in a given year, then the model would account for it. Therefore, we do not expect to impose any relationship that does not already exist in the data.

One last concern is that EBF is strongly influenced by policies and programs towards improvements in BF rates. Have you considered including this kind of variables in your models?

Response:

One of the goals of this study was to provide estimates of exclusive breastfeeding (EBF) prevalence that could inform where changes in policies or programs, or additional resources, should be focused. By providing these EBF prevalence estimates at

administrative-unit levels, we hoped that these estimates could later be compared against the history of EBF-related policies and programs by area to determine which were most and least effective. Since future policy analysis using our estimates was a goal of this study, we could not use policy or program influence as a covariate in our model.

The household survey data we used, and in turn our estimates of EBF prevalence and trends, should already capture the resulting impact of any relevant policies and programs in place. If the goal were to determine which policies/ interventions were most or least effective, additional studies would be required, and measurement of the level of enforcement of these policies/ interventions and local cultural beliefs on breastfeeding would also need to be considered for this kind of analysis. We did not have the resources available to thoroughly conduct such an analysis on a global level.

Referee #3 (expertise – Bayesian geospatial modeling):

Remarks to the Author:
This is an impressive work from Hay's group and collaborators, and as usual rich in details and open information. The methods are coherent with the project objectives. Many congratulations to the authors for this massive work.

I have only few points that the authors are invited to consider in reviewing the manuscript.

The main one is around uncertainty. The uncertainty is not played well, but actually there is a lot of uncertainty in the results (Extended data Figure 3). I will suggest to add a section in the manuscript describing the different uncertainties and ideally linking it with Supplementary Figure 1 to 5 where some of the countries are really lacking in data. In addition, a section on uncertainty could clear the results, where in my view some statements does not take into account uncertainty (for example when describing EBF in South Africa).

Response:
Thank you for your comment. We added statements regarding uncertainties in the 'Limitations' section in the online methods, and link it with Supplementary Figures 1 to 5:

"To estimate projections of EBF prevalence levels in 2025 and 2030, we used previous historical trends and the assumption that recent trends will

continue. These assumptions in turn lend to modelling limitations, as we were not able to project underlying drivers of changes in EBF, such as increasing urbanization or changes in population[11–13]*, and the certainty of our estimates and projections were critically dependent on data quality and availability. Availability of relevant data varies both spatially and temporally across LMICs (Supplementary Figures 1–5), and lack of relevant data is one of the main sources of uncertainty around our estimates (as seen in Extended Data Figure 3). We have mapped EBF prevalence levels against the relative uncertainty of our estimates in Extended Data Figure 3."*

We discuss this more in the Supplemental materials (Section 6.0., Data Availability Section):

"Most importantly, the accuracy of our estimates is critically dependent on the quantity and quality of the underlying data. Availability of relevant data varies both spatially and temporally across LMICs (Supplementary Figures 1–5). For example, temporal data gaps are observed in South Sudan (for the 2000–2002 period) and in Namibia (for the 2008–2012 period), wheras spatial data gaps are seen in Botswana (for the 2003–2007 period) and in South Africa (for the 2013–2018 period). We have constructed a large database of geo-located EBF prevalence data for the purposes of this analysis; nonetheless, important gaps in data coverage, both spatial and temporal, remain (Supplementary Figures 1–5), and these gaps are main sources of uncertainty around our estimates (as seen in Extended Data Figure 3)."

Other minor points.
1) I like the model validation approach. Please can you provide more details on the out-of-sample. Was it balanced, e.g. showing a good proportion of both low and high values?

Response:
Thank you for your comment. We used spatially stratified out-of-sample cross-validation. To do so, we first split all survey data into five groups by randomly sorting a list of unique identifiers for each survey, calculating the cumulative number of spatial points represented by the surveys in this list, and then dividing the list into five parts at the point where this number of spatial points was closest to 20%, 40%, 60%, and 80% of the

total. This resulted in five groups that were approximately equal in terms of the total number of spatial points and contain entire surveys (i.e., all the data points derived from each survey were contained exclusively within only one fold).

2) In post estimation (page 17) the authors stated “We used absolute differences between lowest and highest units and relative differences between a country’s average and each unit in that country to quantify geographic inequality.” I assume these are the modelled ones, aren't they?

Response:

Yes, this is correct. The post-estimation process involves an analysis of the estimates produced by the geostatistical model. We modified this sentence as the following:

> *“Based on the estimates, we calculated absolute differences between lowest and highest administrative units and relative differences between a country’s average and each administrative unit in that country to quantify geographic inequality.”*

3) Modelling limitations section. I think in reality you can include a certain amount of uncertainty (unstructured) just by working on your priors (for example comparing with non-informative priors and setting them with large boundaries).

Response:

Thank you for your comment. In previous study, we ran a series of sensitivity tests comparing our predictions between the default INLA gamma priors and the informative and less informative priors. Due to close concordance in predictions (above 0.98) and no meaningful difference identified in the fit statistics, we have decided to maintain the default priors. This suggests that the predictions are relatively robust to different hyper-prior specifications[16].

4) In extended data figure 2 it is not clear how the uncertainty reflects the data heterogeneity. For example, in BFA the uncertainty is very low when you have an “other survey” polygon. I would have expected a large uncertainty there because the other survey is almost an outlier. The same for BRA, YEM etc… Or may be my interpretation is wrong. Please clarify.

Response:

Thank you for your comment. We performed a thorough data validation and excluded any survey outliers before modeling (Supplementary Figure 6). For Burkina Faso (BFA), “other survey” is BFA National Nutrition Survey 2016 and is not considered to be an

outlier. In fact, it is also cited in Global Nutrition Report 2018[11] and Infant and Young Child Feeding (IYCF) database)[17]. Similarly for other countries, only surveys that met inclusion criteria (described in Section 2.2 of Supplementary Information) were included in the Extended Data Figure 2. Therefore, we do not expect to have a large uncertainty for places with good temporal and spatial data coverage and/or large sample size.

5) In Extended Data Figure 3, I can understand with low prevalence and sparse data you will have a large uncertainty. I wonder if the results need to be described in terms of what is accurate and what is not. May be this can be simply covered in a new section of uncertainty (as advised above).

Response:
Thank you for your suggestion. Throughout the manuscript, we report our estimates along with uncertainty intervals (UIs). We also explain uncertainty of the results in "Methods" and "Limitation" sections (both main manuscript and Supplementary Information). Therefore, we are concerned that creating a new separate section on uncertainty will be redundant, but we have added sentences about uncertainty in the text per your comment above (under "Remarks to Author").

6) Not sure what "not extracted" means in Supplementary Table 1. Why is not extracted?

Response:
When we say that a survey was not extracted, we mean that it was not included in the data processing workflow either because of data restrictions or because it did not meet the inclusion criteria.

7) The resampled data matched to polygons need more explanations. It is not clear how a reduced set of pseudo-location is generated. In particular on the final number of pseudo-locations. In fact, in geostatistics it is more important the number and geometry of the points instead of their value.

Response:
Thank you for your comment. The pseudo-point data were generated based on k-mean clustering on the randomly sampled 10,000 locations (Figure 1B) across 5 × 5-km grid cells in the given polygon with probability proportional to grid-cell population (Figure 1A). Weights were assigned to each pseudo-point proportional to the number of sampled points contained in each of the k-means clusters (Figure 1C). In the example below, the

district of Makonde in Zimbabwe has 11 pseudo-point locations. This was illustrated in previous work by Golding et al., 2017[17] (Figure 1 below).

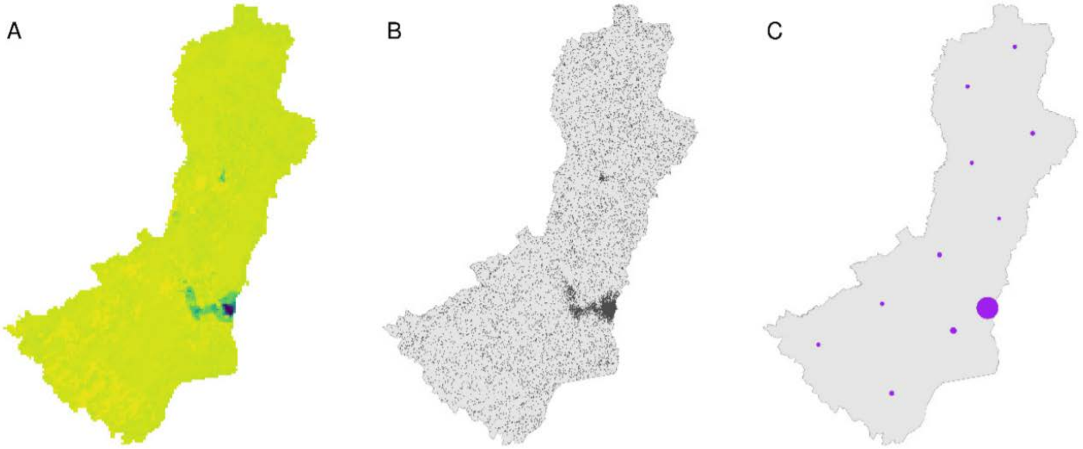

Figure 1. Illustration of k-means clustering for selecting pseudo-point data, applied to the district of Makonde, Zimbabwe.

References

1. Kavle, J., LaCroix, E., Dau, H. & Engmann, C. Addressing barriers to exclusive breast-feeding in low- and middle-income countries: a systematic review and programmatic implications. *Public Health Nutrition* **20**, 3120–3134 (2017).

2. UNICEF. *From the First Hour of Life: Making the case for improved infant and young child feeding everywhere*. https://www.unicef.org/publications/index_93027.html (2016).

3. UNICEF. *A successful start in life: Improving breastfeeding in West and Central Africa*. https://www.unicef.org/health/files/wcaro_improving_breastfeeding_en.pdf (2010).

4. Rollins, N. C. *et al.* Why invest, and what it will take to improve breastfeeding practices? *The Lancet* **387**, 491–504 (2016).

5. Sinha, B. *et al.* Interventions to improve breastfeeding outcomes: a systematic review and meta-analysis. *Acta Paediatrica* **104**, 114–134 (2015).

6. Burstein, R. *et al.* Mapping 123 million neonatal, infant and child deaths between 2000 and 2017. *Nature* **574**, 353–358 (2019).
7. Reiner, Jr., R. C. *et al.* Mapping geographical inequalities in childhood diarrhoeal morbidity and mortality in low-income and middle-income countries, 2000–17: analysis for the Global Burden of Disease Study 2017. *The Lancet* **395**, 1779–1801 (2020).
8. Wiens, K. E. *et al.* Mapping geographical inequalities in oral rehydration therapy coverage in low-income and middle-income countries, 2000–17. *The Lancet Global Health* **8**, e1038–e1060 (2020).
9. Kinyoki, D. K. *et al.* Mapping child growth failure across low- and middle-income countries. *Nature* **577**, 231–234 (2020).
10. Murray, C. J. *et al.* GBD 2010: design, definitions, and metrics. *The Lancet* **380**, 2063–2066 (2012).
11. Development Initiatives. *2018 Global Nutrition Report: Shining a light to spur action on nutrition*. https://globalnutritionreport.org/reports/global-nutrition-report-2018/ (2018).
12. Lugina, H. I. Breastfeeding commitments and challenges in Africa. *African Journal of Midwifery and Women's Health* **5**, 4–4 (2011).
13. Perez-Escamilla, R. Breastfeeding in Africa and the Latin American and Caribbean Region: the Potential Role of Urbanization. *J Trop Pediatr* **40**, 137–143 (1994).
14. Wray, J. D. Maternal Nutrition, Breast-Feeding and Infant Survival. in *Nutrition and Human Reproduction* (ed. Mosley, W. H.) 197–229 (Springer US, 1978). doi:10.1007/978-1-4684-0790-7_12.
15. Lartey, A. Maternal and child nutrition in Sub-Saharan Africa: challenges and interventions. *Proceedings of the Nutrition Society* **67**, 105–108 (2008).
16. UNICEF. Infant and young child feeding. *UNICEF DATA: Monitoring the situation of children and women* https://data.unicef.org/topic/nutrition/infant-and-young-child-feeding/.
17. Golding, N. *et al.* Mapping under-5 and neonatal mortality in Africa, 2000-15: a baseline analysis for the Sustainable Development Goals. *The Lancet* **390**, 2171–2182 (2017).

Decision Letter, first revision:

27th January 2021

Dear Simon,

Thank you for submitting your revised manuscript "Mapping inequalities in exclusive breastfeeding in low- and middle-income countries, 2000–2018" (NATHUMBEHAV-200510750A). Your manuscript has now been evaluated by the original referees and their comments are below. As you can see, all reviewers are satisfied with the revisions and responses to their previous feedback. I am therefore

pleased to let you know that we will be happy in principle to publish it in Nature Human Behaviour, pending minor revisions to comply with our editorial and formatting guidelines.

We are now performing detailed checks on your paper and will send you a checklist detailing our editorial and formatting requirements in about a week. Please do not upload the final materials and make any revisions until you receive this additional information from us.

Thank you again for offering us this work. Please do not hesitate to contact me if you have any questions.

With best wishes,

Stavroula

Stavroula Kousta, PhD
Chief Editor
Nature Human Behaviour

Reviewer #1 (Remarks to the Author):

The authors have addressed the comments appropriately.

Reviewer #2 (Remarks to the Author):

I want to congratulate the authors for the thorough work. Definitely, it will be an important evidence for policymakers to take some within-countries decisions in order to improve EBF rates. I don't have any further comment to place.

Reviewer #3 (Remarks to the Author):

I thanks the authors for reviewing the manuscript and scrupulously taking into account my comments. I am satisfied with the current version of the manuscript regarding its methodology.

Decision letter, final requests:

2nd February 2021

Dear Dr. Hay,

Thank you for your patience as we've prepared the guidelines for final submission of your Nature Human Behaviour manuscript, "Mapping inequalities in exclusive breastfeeding in low- and middle-income countries, 2000–2018" (NATHUMBEHAV-200510750A). Please carefully follow the step-by-step

instructions provided in the personalised checklist attached, to ensure that your revised manuscript can be swiftly handed over to our production team.

We hope to receive your revised paper, with all of the requested files and forms, within 10 days. If you anticipate delays, we would be grateful if you could contact us to provide us with an estimate regarding when you will submit these files.

When you upload your final materials, please include a point-by-point response to any remaining reviewer comments.

If you have not done so already, please alert us to any related manuscripts from your group that are under consideration or in press at other journals, or are being written up for submission to other journals (see: https://www.nature.com/nature-research/editorial-policies/plagiarism#policy-on-duplicate-publication for details).

Nature Human Behaviour offers a Transparent Peer Review option for new original research manuscripts submitted after December 1st, 2019. As part of this initiative, we encourage our authors to support increased transparency into the peer review process by agreeing to have the reviewer comments, author rebuttal letters, and editorial decision letters published as a Supplementary item. When you submit your final files please clearly state in your cover letter whether or not you would like to participate in this initiative. Please note that failure to state your preference will result in delays in accepting your manuscript for publication.

In recognition of the time and expertise our reviewers provide to Nature Human Behaviour's editorial process, we would like to formally acknowledge their contribution to the external peer review of your manuscript entitled "Mapping inequalities in exclusive breastfeeding in low- and middle-income countries, 2000–2018". On a trial basis for those reviewers who give their assent, we will be publishing their names alongside the published article.

Cover suggestions

As you prepare your final files we encourage you to consider whether you have any images or illustrations that may be appropriate for use on the cover of Nature Human Behaviour.

Covers should be both aesthetically appealing and scientifically relevant, and should be supplied at the best quality available. Due to the prominence of these images, we do not generally select images featuring faces, children, text, graphs, schematic drawings, or collages on our covers.

We accept TIFF, JPEG, PNG or PSD file formats (a layered PSD file would be ideal), and the image should be at least 300ppi resolution (preferably 600-1200 ppi), in CMYK colour mode.

If your image is selected, we may also use it on the journal website as a banner image, and may need to make artistic alterations to fit our journal style.

Please submit your suggestions, clearly labeled, along with your final files. We'll be in touch if more information is needed.

Nature Human Behaviour has now transitioned to a unified Rights Collection system which will allow our Author Services team to quickly and easily collect the rights and permissions required to publish your work. Approximately 10 days after your paper is formally accepted, you will receive an email in providing you with a link to complete the grant of rights. If your paper is eligible for Open Access, our Author Services team will also be in touch regarding any additional information that may be required to arrange payment for your article.

Please note that you will not receive your proofs until the publishing agreement has been received through our system.

For information regarding our different publishing models please see our Transformative Journals page. If you have any questions about costs, Open Access requirements, or our legal forms, please contact ASJournals@springernature.com.

Please use the following link for uploading these materials:
[REDACTED]

If you have any further questions, please feel free to contact me.

Best regards,

Stavroula Kousta

Stavroula Kousta, PhD
Chief Editor
Nature Human Behaviour

Reviewer #1:
Remarks to the Author:
The authors have addressed the comments appropriately.

Reviewer #2:
Remarks to the Author:
I want to congratulate the authors for the thorough work. Definitely, it will be an important evidence for policymakers to take some within-countries decisions in order to improve EBF rates. I don't have any further comment to place.

Reviewer #3:
Remarks to the Author:
I thanks the authors for reviewing the manuscript and scrupulously taking into account my comments. I am satisfied with the current version of the manuscript regarding its methodology.

Final Decision Letter:

Dear Simon,

I am pleased to inform you that your Article "Mapping inequalities in exclusive breastfeeding in low- and middle-income countries, 2000–2018", has now been accepted for publication in Nature Human Behaviour.

Before your manuscript is typeset, we will edit the text to ensure it is intelligible to our wide readership and conforms to house style. We look particularly carefully at the titles of all papers to ensure that they are relatively brief and understandable.

Once your manuscript is typeset and you have completed the appropriate grant of rights, you will receive a link to your electronic proof via email with a request to make any corrections within 48 hours. If, when you receive your proof, you cannot meet this deadline, please inform us at rjsproduction@springernature.com immediately. Once your paper has been scheduled for online publication, the Nature press office will be in touch to confirm the details.

Acceptance of your manuscript is conditional on all authors' agreement with our publication policies (see http://www.nature.com/nathumbehav/info/gta). In particular your manuscript must not be published elsewhere and there must be no announcement of the work to any media outlet until the publication date (the day on which it is uploaded onto our web site).

Please note that <i>Nature Human Behaviour</i> is a Transformative Journal (TJ). Authors may publish their research with us through the traditional subscription access route or make their paper immediately open access through payment of an article-processing charge (APC). Authors will not be required to make a final decision about access to their article until it has been accepted. Find out more about Transformative Journals

<B>Authors may need to take specific actions to achieve compliance with funder and institutional open access mandates. For submissions from January 2021, if your research is supported by a funder that requires immediate open access (e.g. according to Plan S principles) then you should select the gold OA route, and we will direct you to the compliant route where possible. For authors selecting the subscription publication route our standard licensing terms will need to be accepted, including our self-archiving policies. Those standard licensing terms will supersede any other terms that the author or any third party may assert apply to any version of the manuscript.

If you have posted a preprint on any preprint server, please ensure that the preprint details are updated with a publication reference, including the DOI and a URL to the published version of the article on the journal website.

An online order form for reprints of your paper is available at https://www.nature.com/reprints/author-reprints.html. All co-authors, authors' institutions and authors' funding agencies can order reprints using the form appropriate to their geographical region.

We welcome the submission of potential cover material (including a short caption of around 40 words) related to your manuscript; suggestions should be sent to Nature Human Behaviour as electronic files (the image should be 300 dpi at 210 x 297 mm in either TIFF or JPEG format). Please note that such pictures should be selected more for their aesthetic appeal than for their scientific content, and that colour images work better than black and white or grayscale images. Please do not try to design a cover with the Nature Human Behaviour logo etc., and please do not submit composites of images related to your work. I am sure you will understand that we cannot make any promise as to whether any of your suggestions might be selected for the cover of the journal.

You can now use a single sign-on for all your accounts, view the status of all your manuscript submissions and reviews, access usage statistics for your published articles and download a record of your refereeing activity for the Nature journals.

To assist our authors in disseminating their research to the broader community, our SharedIt initiative provides you with a unique shareable link that will allow anyone (with or without a subscription) to read the published article. Recipients of the link with a subscription will also be able to download and print the PDF.

As soon as your article is published, you will receive an automated email with your shareable link.

In approximately 10 business days you will receive an email with a link to choose the appropriate publishing options for your paper and our Author Services team will be in touch regarding any additional information that may be required.

You will not receive your proofs until the publishing agreement has been received through our system.

If you have any questions about our publishing options, costs, Open Access requirements, or our legal forms, please contact ASJournals@springernature.com

We look forward to publishing your paper.

With all best wishes,

Stavroula

Stavroula Kousta, PhD
Chief Editor
Nature Human Behaviour

P.S. Click on the following link if you would like to recommend Nature Human Behaviour to your librarian http://www.nature.com/subscriptions/recommend.html#forms

** Visit the Springer Nature Editorial and Publishing website at www.springernature.com/editorial-and-publishing-jobs for more information about our career opportunities. If you have any questions please click here.**
